# Synthesis of Non‐Symmetric Azoarenes by Palladium‐Catalyzed Cross‐Coupling of Silicon‐Masked Diazenyl Anions and (Hetero)Aryl Halides

**DOI:** 10.1002/anie.202210907

**Published:** 2022-08-29

**Authors:** Lucie Finck, Martin Oestreich

**Affiliations:** ^1^ Institut für Chemie Technische Universität Berlin Strasse des 17. Juni 115 10623 Berlin Germany

**Keywords:** Azo Compounds, Chemoselectivity, Cross-Coupling, Palladium, Silicon

## Abstract

The photoswitchable motif of azobenzenes is of great importance across the life and materials sciences. This maintains a constant demand for their efficient synthesis, especially that of non‐symmetric derivatives. We disclose here a general strategy for their synthesis through an unprecedented C(sp^2^)−N(sp^2^) cross‐coupling where functionalized aryl‐substituted diazenes masked with a silyl group are employed as diazenyl pronucleophiles. These equivalents of fragile diazenyl anions couple with a diverse set of (hetero)aryl bromides under palladium catalysis *with no loss of dinitrogen*. The competing denitrogenative biaryl formation is fully suppressed. The reaction requires only a minimal excess, that is 1.2 equivalents, of the diazenyl component. By this, a broad range of azoarenes decorated with two electron‐rich/deficient aryl groups can be accessed in a predictable way with superb functional‐group tolerance.

## Graphical abstract

A general strategy for the synthesis of non‐symmetric azobenzene derivatives by an unprecedented C(sp^2^)−N(sp^2^) cross‐coupling is presented. *N*‐Aryl‐*N′*‐silyldiazenes are employed as diazenyl anion equivalents that can be selectively coupled under palladium catalysis with a large array of (hetero)aryl bromides in high yields. The functional‐group tolerance on either (hetero)aryl ring is excellent.

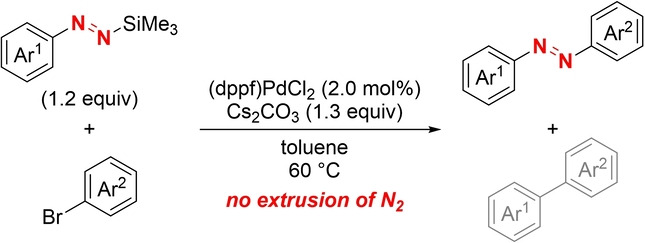

Since Mitscherlich's almost two‐hundred‐year‐old description of azobenzene in the literature,^[1]^ this class of aromatic azo compounds continues to find widespread application as organic dyes, molecular photoswitches, and therapeutic agents owing to its tunable chemical and physical properties.^[2]^ Viable synthetic approaches to symmetrically substituted azobenzene derivatives can be considered largely established but the preparation of their non‐symmetric counterparts remains challenging (Scheme 1, top).^[3]^ The azo coupling is a commonly used strategy involving an S_E_Ar reaction of a diazonium salt and an electron‐rich arene.[^3^, ^4^] Alternatively, the coupling of nitrosoarenes and aniline derivatives under acidic conditions, also known as the Baeyer‐Mills reaction, provides an access to non‐symmetric products.[^3^, ^5^] An enabling method to obviate the limited substrate scope of the aforementioned protocols is the coupling of those diazonium salts and metalated arenes.[^6^, ^7^] Feringa and co‐workers utilized this method for the preparation of difficult‐to‐access tetra‐*ortho*‐substituted red‐shifted azobenzenes from lithiated aryl nucleophiles.^[7]^ Azoarenes can also be obtained from readily available and less reactive precursors through dehydrogenative oxidative coupling of aniline derivatives[^8^, ^9^] or by reductive heterodimerization of nitroarenes.[^10^, ^11^] While these methods proved to be efficient per se, the distribution between homo‐ and heterodimerized products can often not be satisfactorily controlled. To achieve preferential heterodimerization, a large excess of either coupling partner is typically required. That limitation was recently addressed by Li and co‐workers with the development of a Chan‐Evans‐Lam‐type oxidative cross‐coupling of *N*‐aryl phthalic hydrazides and aryl boronic acids under copper catalysis (Scheme 1, bottom).^[12]^ This elegant solution furnished a subclass of azoarenes in moderate to good yields.

**Scheme 1:**
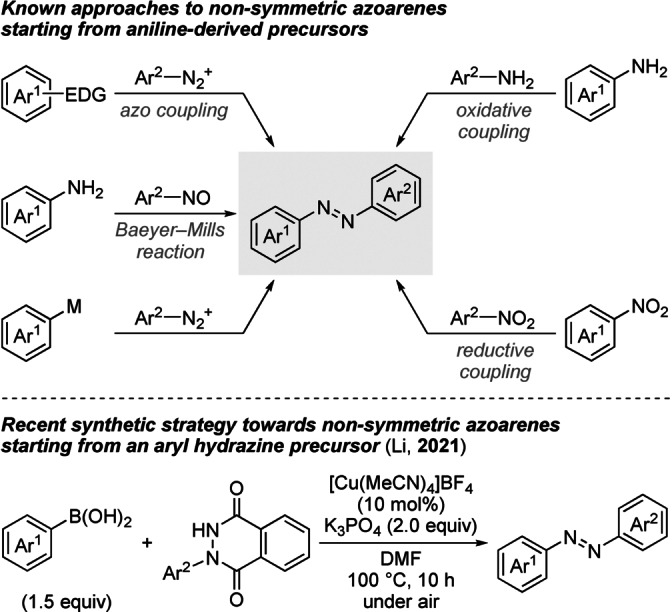
Synthetic routes to non‐symmetric azobenzene derivatives through C−N or N−N bond‐forming reactions. Ar=aryl group, EDG=electron‐donating group, M=metal.

Our research group recently rediscovered readily accessible aryl‐substituted diazenes with their terminus capped by a silyl residue.^[13]^ These *N*‐aryl‐*N′*‐silyldiazenes are kinetically stable and have already been employed *with* loss of dinitrogen as aryl pronucleophiles.[^14^, ^15^] We asked ourselves whether the same set of silylated diazenes could serve as precursors for diazenyl anions *without* extrusion of dinitrogen (Scheme 2, top). Diazenes are assumed intermediates in denitrogenative palladium‐catalyzed cross‐coupling reactions of arylhydrazine derivatives and aryl halides to form biaryls.^[16]^ In contrast, we anticipated the direct synthesis of non‐symmetric azobenzene derivatives starting from the aforementioned masked, aryl‐substituted diazenes and various (hetero)aryl halides as coupling partners (Scheme 2, bottom). This unknown cross‐coupling is different from the common detour where Buchwald–Hartwig‐type cross‐coupling of protected arylhydrazines is followed by an oxidation step.^[17]^ We aimed at a C(sp^2^)−N(sp^2^) cross‐coupling of in situ‐generated diazenyl anions and (hetero)aryl electrophiles under palladium catalysis. This type of rare transformation has only been applied with imines which act as ammonia surrogates.[^18^, ^19^]

**Scheme 2:**
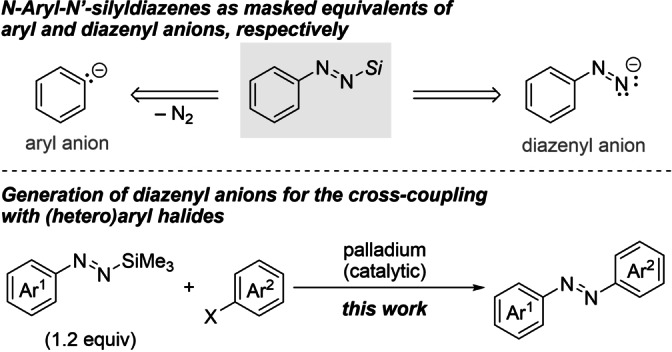
Silylated aryl diazenes as both aryl and diazenyl anion synthons (top) and planned strategy to access non‐symmetric azobenzene derivatives through C(sp^2^)−N(sp^2^) cross‐coupling (bottom). Ar=(hetero)aryl group, *Si*=triorganosilyl, X=(pseudo)halogen.

We began the catalyst identification starting from Cho's above‐mentioned palladium‐catalyzed arylation of hydrazine derivatives where Pd_2_dba_3_ and dppf with Cs_2_CO_3_ as the base in toluene have been employed.^[17]^ The choice of these reaction parameters was guided by our previous experience with the activation of the silylated diazenes; denitrogenation is more likely in polar solvents with the silaphilic Lewis base mostly in solution.^[14]^ A control experiment in THF as solvent revealed though that neither loss of dinitrogen (in traces) nor the targeted coupling occurred; defunctionalization of the aryl bromide was detected. Cs_2_CO_3_ in toluene seemed promising to us, and we set the reaction temperature to 60 °C rather than 110 °C not to facilitate loss of dinitrogen. Moreover, the wide bite angle of the dppf ligand would likely exert a similar effect by accelerating reductive elimination.^[20]^ The *para*‐tolyl‐substituted diazenyl pronucleophile **1 a** and the more challenging^[21]^ electron‐rich aryl bromide **2 a** were utilized as model compounds (Table 1; see the Supporting Information for the complete optimization of the reaction conditions). Those reaction conditions indeed afforded the desired azobenzene derivative **6 aa** in good yield with no formation of the biaryl **7 aa** (entry 1). Changing from the palladium(0) to a palladium(II) precatalyst, we performed the cross‐coupling with preformed (dppf)PdCl_2_. This modification brought about chemoselective formation of **6 aa** in 99 % yield after 15 h (entry 2). Aside from the shorter reaction time compared to that using Cho's modified procedure (60 °C instead of 110 °C), no byproducts did form, and there was also no need for chromatographic removal of dba. Other commercially available palladium(II) complexes such as (dtbpf)PdCl_2_, (dppe)PdCl_2_, and (Ph_3_P)_2_PdCl_2_ either provided a mixture of **6 aa** and **7 aa** or did not lead to any conversion (entries 3–5). We then turned our attention towards the influence of the leaving group on the aryl electrophile **3 a**–**5 a**. Both aryl triflate **3 a** and iodide **4 a** displayed comparable reactivity to bromide **2 a**, yet yields and chemoselectivities were inferior (entries 6 and 7). However, lowering the reaction temperature to 45 °C decreased the amount of denitrogenation fully for aryl triflate **3 a** and partially for aryl iodide **4 a** (not shown). The less reactive aryl chloride **5 a** did not react (entry 8). Several bases expected to promote the N−Si bond cleavage were examined.^[14]^ Owing to their poor solubility, K_2_CO_3_ and CsF resulted in low conversion of the starting materials while NaO*t*Bu afforded product **6 aa** in 63 % yield (entries 9–11). No reaction was seen in the absence of a base or a precatalyst (entries 12 and 13).

**Table 1:** Selected examples of the optimization of the palladium‐catalyzed cross‐coupling of a masked diazenyl anion and an electron‐rich aryl (pseudo)halide.^[a]^

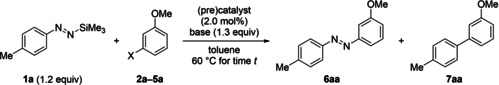						
Entry	X	(Pre)catalyst	Base	*t* [h]	Yield of **6 aa** [%]^[b]^	Yield of **7 aa** [%]^[b]^
1	Br (**2 a**)	Pd_2_dba_3_/dppf^[c]^	Cs_2_CO_3_	48	90	0
2	Br (**2 a**)	(dppf)PdCl_2_	Cs_2_CO_3_	15	99 (92)^[d]^	0
3^[e]^	Br (**2 a**)	(dtbpf)PdCl_2_	Cs_2_CO_3_	48	16	35
4^[e]^	Br (**2 a**)	(dppe)PdCl_2_	Cs_2_CO_3_	48	trace	0
5^[e]^	Br (**2 a**)	(Ph_3_P)_2_PdCl_2_	Cs_2_CO_3_	48	trace	0
6	OTf (**3 a**)	(dppf)PdCl_2_	Cs_2_CO_3_	15	76	9
7	I (**4 a**)	(dppf)PdCl_2_	Cs_2_CO_3_	15	59	28
8^[e]^	Cl (**5 a**)	(dppf)PdCl_2_	Cs_2_CO_3_	48	0	0
9^[e]^	Br (**2 a**)	(dppf)PdCl_2_	K_2_CO_3_	48	trace	0
10^[e]^	Br (**2 a**)	(dppf)PdCl_2_	CsF	24	26	trace
11^[e]^	Br (**2 a**)	(dppf)PdCl_2_	NaO*t*Bu	15	63	0
12	Br (**2 a**)	(dppf)PdCl_2_	–	48	0	0
13	Br (**2 a**)	–	Cs_2_CO_3_	48	0	0

[a] All reactions were performed on a 0.10 mmol scale in 0.2 mL of toluene (0.5 M). [b] Determined by calibrated GLC analysis with tetracosane as an internal standard. [c] 1.0 mol% of Pd_2_dba_3_ and 3.0 mol% of dppf. [d] Isolated yield on a 0.20 mmol scale after purification by flash chromatography on silica gel in parentheses. [e] Incomplete conversion of the aryl (pseudo)halide. dba=dibenzylideneacetone; dppf=1,1′‐bis(diphenylphosphino)ferrocene; dtbpf=1,1′‐bis(di‐*tert*‐butylphosphino)ferrocene; dppe=1,2‐bis(diphenylphosphino)ethane.

With suitable reaction conditions established (Table 1, entry 2), we turned towards the examination of the substrate scope (Schemes 3, 4, 5). A series of functionalized silyldiazenes **1 a**–**l** were successfully coupled with 1‐bromo‐3‐fluorobenzene (**2 b**) (Scheme 3). By this, the synthesis of the non‐symmetric, fluorinated aromatic azo compounds **6 ab**–**lb** known to be difficult to prepare by existing procedures was accomplished (Scheme 3; see Figure S1 in the Supporting Information for an overview of known and unknown substitution patterns). Coupling reactions of aryldiazenes substituted with an electron‐donating 4‐methyl (as in **1 a**) or 4‐methoxy group (as in **1 b**) provided the corresponding azobenzenes **6 ab** and **6 bb** in excellent yields, and the parent phenyldiazene **1 c** reacted equally well. Halogen atoms (F in **1 d** and Cl in **1 e**) as well as electron‐withdrawing substituents were also compatible, and the coupling products **6 db**–**hb** bearing two electron‐deficient aromatic rings were obtained in good yields. The competing denitrogenation and slower reaction rates were noted for nucleophiles decorated with strongly electron‐withdrawing groups such as trifluoromethyl (**1 f**) or cyano (**1 g**).^[21]^ In line with these findings, the *meta*‐nitro‐substituted diazene did not exhibit any reactivity under the standard reaction conditions (not shown). Additionally, *ortho*‐ and *meta*‐substituents were not detrimental, and **6 ib** and **6 jb** were isolated in high yields. Sterically congested **1 j** required a reduced reaction temperature (45 °C) to alleviate the loss of dinitrogen. A replacement of the aryl moiety with the β‐naphthyl unit was also tolerated to afford **6 kb** in 87 % yield. Finally, our method was applied to the 1,3‐bisdiazene **1 l** to give the bisazobenzene **6 lb** in 68 % yield.

**Scheme 3:**
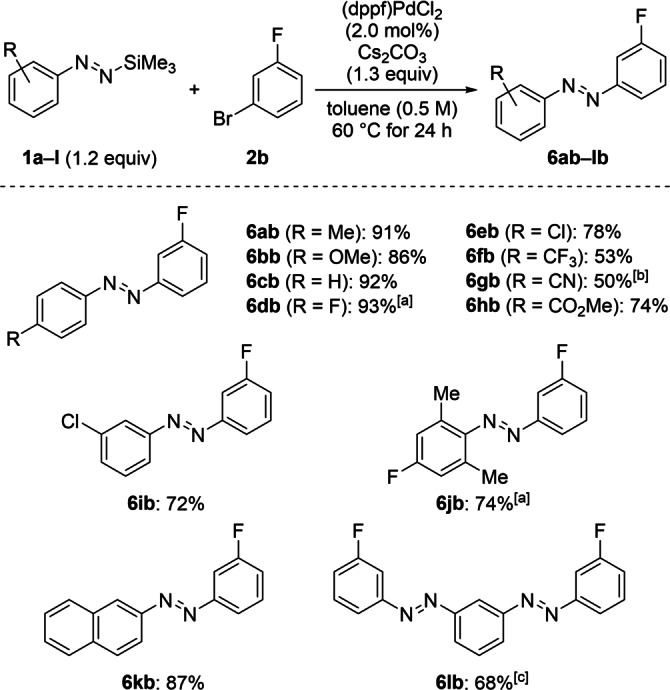
Scope I: Palladium‐catalyzed cross‐coupling of functionalized silylated aryldiazenes **1 a**–**l** and 1‐bromo‐3‐fluorobenzene (**2 b**). Unless otherwise noted, all reactions were performed on a 0.20 mmol scale. Yields are of isolated products after purification by flash chromatography on silica gel. [a] Run at 45 °C. [b] The reaction time was 48 h. [c] Reaction performed with 0.44 mmol of 1‐bromo‐3‐fluorobenzene (**2 b**) and 0.45 equiv of bisdiazene **1 l** using 2.0 mol% of (dppf)PdCl_2_ and 1.1 equiv of Cs_2_CO_3_ in 0.4 mL of toluene (see the Supporting Information for details).

**Scheme 4:**
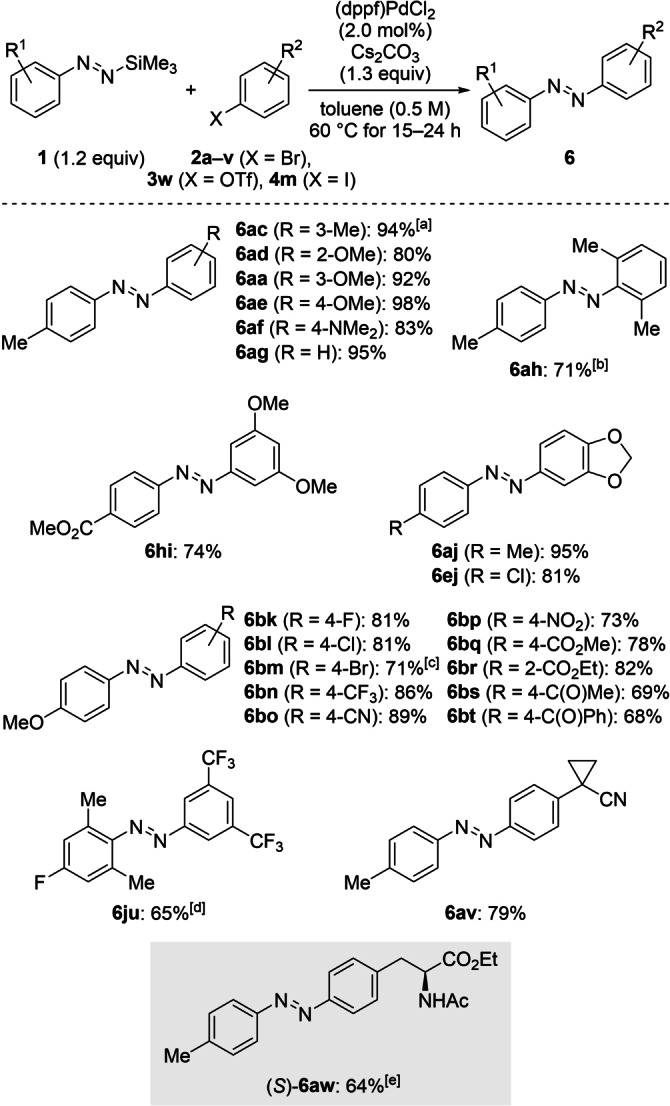
Scope II: Palladium‐catalyzed cross‐coupling of functionalized silylated aryldiazenes **1** and various aryl (pseudo)halides **2 a**–**v**, **3 w**, and **4 m**. Unless otherwise noted, all reactions were performed on a 0.20 mmol scale. Yields are of isolated products after purification by flash chromatography on silica gel. [a] 88 % were obtained on a 2.0 mmol scale. [b] Run at 80 °C with 4.0 mol% of (dppf)PdCl_2_. [c] 1‐Bromo‐4‐iodobenzene (**4 m**) was used. [d] Run at 45 °C. [e] Reaction was performed with the corresponding aryl triflate **3 w** on a 0.10 mmol scale.

**Scheme 5:**
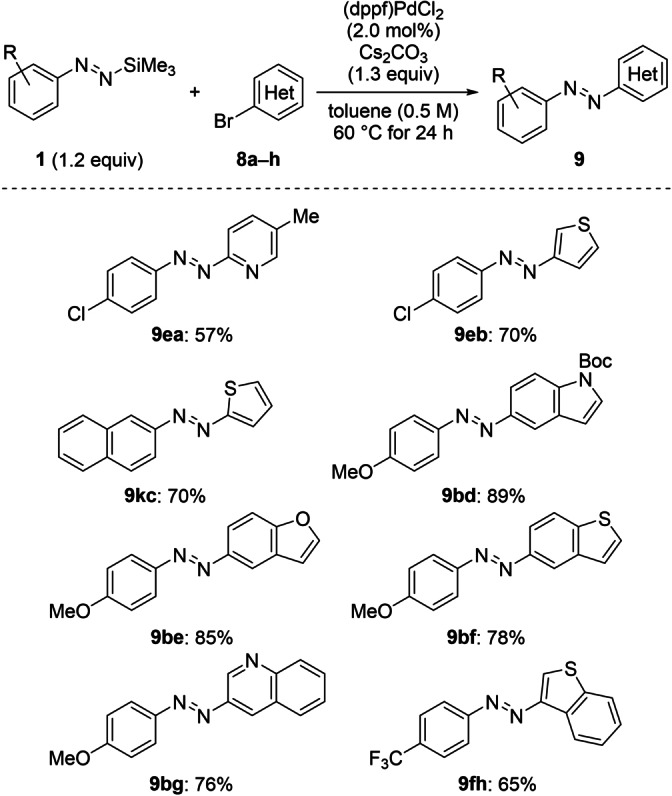
Scope III: Palladium‐catalyzed cross‐coupling of functionalized silylated aryldiazenes **1** and various heteroaryl bromides **8 a**–**h**. All reactions were performed on a 0.20 mmol scale. Yields are of isolated products after purification by flash chromatography on silica gel.

We next subjected a variety of aryl bromides to the optimized setup (Scheme 4). A large number of electronic and steric modifications was compatible with our method. We first tested electron‐rich coupling partners. (*E*)‐1‐(*m*‐Tolyl)‐2‐(*p*‐tolyl)diazene (**6 ac**) bearing a methyl group on each aromatic ring in distinct positions was isolated in 94 % yield. Additionally, methoxy substitution was tolerated in the *ortho*‐ (**2 d**), *meta*‐ (**2 a**), and *para*‐positions (**2 e**), respectively. Similarly, the dimethylamino‐substituted electrophile **2 f** led to the methyl yellow derivative **6 af**, and bromobenzene (**2 g**) was also a competent substrate. Disubstitution in the *ortho*‐ (**2 h**) or *meta*‐positions (**2 i**) furnished **6 ah** and **6 hi** in high yields; the same applied to catechol‐derived aryl bromide **2 j** to yield **6 aj** and **6 ej**. Of note, sterically hindered 2‐bromo‐1,3‐dimethylbenzene (**2 h**) did not show any reactivity under the standard reaction conditions but did convert into **6 ah** at a higher reaction temperature (80 °C) and catalyst loading (4.0 mol%). Halogens and electron‐withdrawing groups were also well tolerated, allowing for the preparation of push‐pull‐type azobenzene derivatives **6 bk**–**6 bt**. 1‐Bromo‐4‐iodobenzene (**4 m**) underwent exclusive coupling at the iodine‐substituted carbon atom with no detectable bromine displacement to yield **6 bm** while the corresponding dibromobenzene (**2 m**) led to a messy product mixture. Aryl bromides with sensitive functional groups such as nitro (as in **2 p**), alkoxycarbonyl (as in **2 q** and **2 r**), and even (non‐)enolizable ketones (as in **2 s** and **2 t**) reacted chemoselectively in high yields. The sterically congested azobenzene **6 ju** was obtained at 45 °C in 65 % yield. Substrate **2 v** containing a cyclopropyl ring furnished the desired coupling product **6 av** in 79 % yield. To illustrate the applicability of the method further, we eventually probed the aryl triflate **3 w** derived from the amino acid tyrosine, which yielded the corresponding azobenzene (*S*)‐**6 aw** in 64 % yield (gray box).

Given that the introduction of heteroaromatic motifs into azo compounds is not an easy task (see Figure S1), we tested a number of heteroaryl bromides (Scheme 5). Without the need for adapting the general procedure, a variety of heterocyclic bromoarenes **8 a**–**h** effectively underwent the coupling in high yields. Azo compounds bearing pyridyl (as in **9 ea**) and thienyl units (as in **9 eb** and **9 kc**) were successfully isolated in yields of 57 % and 70 %, respectively. The position of the heteroatom had no significant influence on the yield as shown with substrates **8 b** and **8 c**. Benzo‐fused heteroaromatics **8 d**–**h** such as a Boc‐protected indole (for **9 bd**), a benzofuran (for **9 be**), a benzothiophene (for **9 bf** and **9 fh**) as well as a quinoline (for **9 bg**) exhibited a high reactivity and furnished the corresponding heteroazobenzene derivatives in very good yields.

Based on generally accepted key steps of palladium‐catalyzed cross‐coupling reactions,^[22]^ a Pd^0^/Pd^II^ catalytic cycle is proposed (Scheme 6, top). The new arylation likely begins with oxidative addition of the aryl halide to the reduced precatalyst **I** to form the arylpalladium(II) halide **II**. Although Hünig as well as Kosower had in fact already shown half a century ago that the *tert*‐butyl‐substituted diazenyl anion can be added to carbonyl compounds,^[23]^ we think that the transmetalation does not involve a “free” diazenyl anion. Instead, palladium(II) intermediate **II** engages in a σ‐bond metathesis with the silylated and as such masked diazenyl anion. Transition state **III** then releases the aryl(diazenyl)palladium(II) complex **IV** which subsequently undergoes reductive elimination to afford the non‐symmetric azoarene. Extrusion of dinitrogen does neither occur at the stage of **IV** nor prior to the transmetalation, i.e. **III**. Both of these pathways would provide the unwanted biaryl (gray box) through the corresponding diarylpalladium(II) species **V** (Scheme 6, bottom).^[16]^ It must be emphasized that the presence of a base is crucial, and Cs_2_CO_3_ had turned out to be optimal (cf. Table 1).

**Scheme 6:**
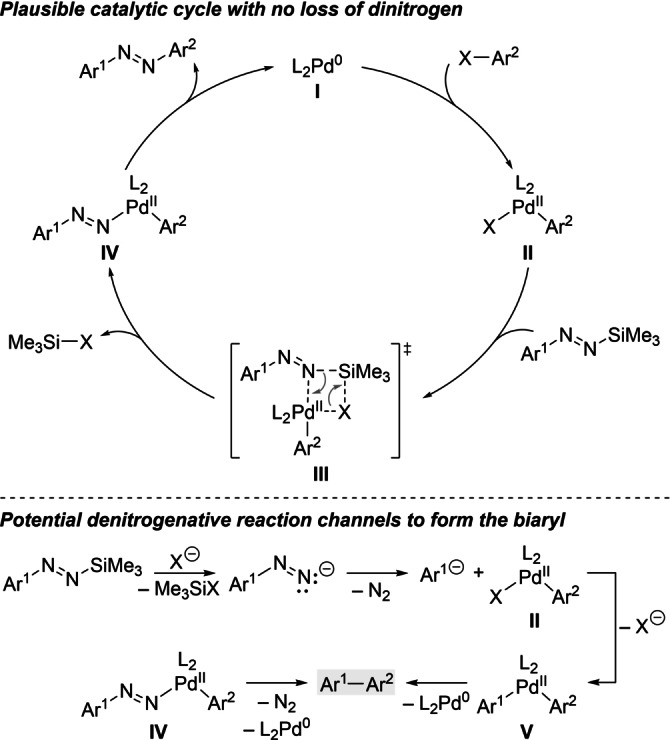
Proposed catalytic cycle (top) and competitive pathways with loss of dinitrogen (bottom). Ar=(hetero)aryl group, X=(pseudo)halogen.

In summary, we developed an efficient palladium‐catalyzed cross‐coupling of diazenyl‐anion equivalents and (hetero)aryl (pseudo)halides for the selective construction of non‐symmetric azobenzene derivatives. The new method does not require excess of either coupling partner, and reactions are routinely run with 1.2 equiv of the diazene pronucleophile. This is also possible because there is hardly any loss of dinitrogen under the optimized reaction conditions, and hence the formation of the undesired biaryl product is not competing. The functional‐group tolerance in both reactants is excellent, thereby enabling the synthesis of new azobenzene derivatives decorated with two totally different aryl groups.

## Conflict of interest

The authors declare no conflict of interest.

## Supporting information

As a service to our authors and readers, this journal provides supporting information supplied by the authors. Such materials are peer reviewed and may be re‐organized for online delivery, but are not copy‐edited or typeset. Technical support issues arising from supporting information (other than missing files) should be addressed to the authors.

Supporting Information

## Data Availability

The data that support the findings of this study are available from the corresponding author upon reasonable request.
