# Supplementary material for: Synthesis of Non‐Symmetric Azoarenes by Palladium‐Catalyzed Cross‐Coupling of Silicon‐Masked Diazenyl Anions and (Hetero)Aryl Halides
Source: Angew Chem Int Ed Engl. 2022 Aug 29;61(40):e202210907. doi: 10.1002/anie.202210907 (PMC9805057; doi:10.1002/anie.202210907)
Supplement: Supplementary file 1 — Supporting Information [file ANIE-61-0-s001.pdf]

Angewandte Chemie
Eine Zeitschrift der Gesellschaft Deutscher Chemiker

Supporting Information

Synthesis of Non-Symmetric Azoarenes by Palladium-Catalyzed Cross-Coupling of Silicon-Masked Diazenyl Anions and (Hetero)Aryl Halides

*L. Finck, M. Oestreich**

Table of Contents

**1**	**General Information**	**S3**
**2**	**Optimization Study**	**S6**
**3**	**Experimental Details for the Preparation of *N*-Aryl-*N'*-Silyldiazenes**	**S8**
3.1	Synthesis of 1-(3-chlorophenyl)-2-(trimethylsilyl)hydrazine (**S2**)	S8
3.2	Synthesis of (*E*)-1-(3-chlorophenyl)-2-(trimethylsilyl)diazene (**1i**)	S9
**4**	**General Procedure for the Coupling of Diazenes and Aryl Bromides (GP1)**	**S10**
**5**	**Characterization Data for the Azobenzene Derivatives**	**S11**
**6**	**Scale-Up Experiment**	**S41**
**7**	**NMR Spectra**	**S42**
**8**	**References**	**S148**

1 General Information

All reactions were performed using standard Schlenk techniques or in an *MBraun* glovebox, respectively under a static pressure of nitrogen or argon, unless otherwise stated. Glassware for reactions outside a glovebox was flame-dried under vacuum using a heat gun. Glassware for reactions performed inside a glovebox was either flame-dried under vacuum using a heat gun or dried overnight in a 120 °C oven before being transferred into the glovebox. Plastic syringes, needles and septa were dried overnight in a 60 °C oven before being transferred into the glovebox. All reactions were stirred with magnetic followers. All stated temperatures refer to external bath/heating aluminum block temperatures. For the addition of liquid reagents and solvents through silicon/rubber septa, argon- or nitrogen-flushed disposable syringes and needles were used. All glass syringes and stainless steel needles were used several times and stored at 120 °C. Solids were added in a countercurrent of inert atmosphere or in solution. Compound names were generated by the computer program *ChemDraw* according to the guidelines specified by the International Union of Pure and Applied Chemistry (IUPAC).

Reagents and solvents

Standard reagents and solvents were obtained from *ABCR*, *Acros Organics*, *Alfa Aesar*, *Carbolution*, *Merck*, *Sigma-Aldrich*, or *Tokyo Chemical Industry* (TCI) and used as received. Alkali metal salts (Cs_2CO_3, K_2CO_3, CsF, CsOPiv, KO*t*Bu, NaO*t*Bu, LiO*t*Bu) were grinded (*if applicable*), dried under high vacuum and stored in a glovebox prior to use. The *N*-aryl-*N'*-silyldiazenes were prepared according to previously reported procedures and stored in a glovebox.[S1] 3-Methoxyphenyl trifluoromethanesulfonate (**3a**), 3-methoxyphenyl 4-methyl-benzenesulfonate (**S1**) and ethyl (*S*)-2-acetamido-3-(4-(((trifluoromethyl)sulfonyl)oxy)-phenyl)-propanoate (**3w**) were synthesized according to reported procedures.[S2] Dichloromethane (CH_2Cl_2), *n*-pentane and triethylamine (Et_3N) were dried over CaH_2 and freshly distilled under nitrogen atmosphere prior to use. Tetrahydrofuran (THF), toluene and diethyl ether (Et_2O) were dried over sodium with benzophenone as indicator and freshly distilled under nitrogen atmosphere prior to use. For extraction and flash chromatography, technical grade solvents (*tert*-butyl methyl ether, *n*-pentane and dichloromethane) were distilled prior to use. All solvents and liquid reagents used in a glovebox were distilled, degassed by the freeze-pump-thaw method, and stored in a glovebox over thermally activated 4 Å molecular sieves.

Chromatography

Qualitative **thin-layer chromatography** (TLC) was performed on *Macherey-Nagel* Alugram® Xtra SIL G/UV_{254} silica gel 60 pre-coated aluminum-backed plates (200 µm layer thickness).

Product spots were visualized under UV light (λ_{max} = 254 nm), by staining with a ceric ammonium molybdate solution and/or by staining with a potassium permanganate solution.

Flash column chromatography was performed on silica gel Davisil LC60A (grain size 40–63 µm, pore size 60 Å, 230–400 mesh ASTM) from *Grace GmbH* according to the method reported by W.C. Still and coworkers.[S3]

Analytical **gas-liquid chromatography** (GLC) of the reaction mixtures and pure substances were performed using a *Varian* 430-GC gas chromatograph equipped with a *Varian* Factor Four Capillary column (30 m × 0.25 mm, 0.25 µm film thickness of the stationnary phase). All GLC analyses were performed by using the following conditions: carrier gas: N_2; injector temperature: 250 °C; detector temperature: 250 °C; flow rate: 4 mL/min; temperature program: starting temperature: 40 °C, heating rate: 10 °C/min, final temperature: 250 °C for 10 min.

Spectroscopy

Nuclear Magnetic Resonance (NMR)

^{1}H, ^{13}C, ^{19}F, and ^{29}Si NMR spectra were recorded in $CDCl_3$ or C_6D_6 on AV 400, AV 500 or AV 700 instruments from *Bruker* at *Institut für Chemie*, *Technische Universität Berlin*, with the deuterated solvent acting as an internal deuterium lock. ^{1}H NMR spectra were recorded at 400, 500 or 700 MHz, ^{13}C NMR spectra at 100, 126 or 176 MHz, ^{19}F NMR spectra at 471 or 659 MHz, and ^{29}Si NMR spectra at 99 MHz, using broadband proton decoupling when indicated. The ^{1}H and ^{13}C chemical shifts are reported in parts per million (ppm), and NMR spectra are referenced to the residual protic solvent resonances and the deuterated solvent carbon resonances, respectively ($CHCl_3$: δ = 7.26 ppm for ^{1}H NMR and $CDCl_3$: δ = 77.16 ppm for ^{13}C NMR, C_6D_5H: δ = 7.16 ppm for ^{1}H NMR and C_6D_6: δ = 128.1 ppm for ^{13}C NMR).[S4] For all other nuclei, the NMR resonance signals were internally calibrated according to the IUPAC recommendation, using a unified chemical shift scale based on the proton resonance of tetramethylsilane as primary reference.[S5] Chemical shifts are reported relative to tetramethylsilane to the 0.01 ppm for ^{1}H NMR spectra and to the 0.1 ppm for ^{13}C, ^{19}F and ^{29}Si NMR spectra. Coupling constants are quoted to the nearest 0.1 Hz for ^{1}H NMR spectra and to full Hz for ^{13}C and ^{19}F NMR spectra. Data are reported as follows: chemical shift, multiplicity (s = singlet, d = doublet, t = triplet, q = quartet, quint = quintet, m = multiplet, m_c = centrosymmetric multiplet, br = broad, app = apparent, and combinations thereof), coupling constants (Hz), and integration. Air- and moisture- sensitive samples were measured in J. Young NMR tubes.

Infrared (IR) spectra were recorded on a Cary 630 FT-IR spectrometer from *Agilent Techologies* equipped with a diamond ATR unit. Infrared spectra of silyl diazenes were

recorded in a glovebox using a *Thermo Nicolet Magna-IR* 750 spectrophotometer equipped with an ATR unit. Selected absorption maxima are reported in wavenumbers (cm^{-1}).

High Resolution Mass Spectrometry (HRMS) measurements were performed at the analytical facilities of the *Institut für Chemie, Technische Universität Berlin* with an LTQ Orbitrap XL using atmospheric-pressure chemical ionization (APCI), electrospray ionization (ESI) or liquid injection field desorption/ionization (LIFDI) methods. The in-detail fragmentation was omitted and only the molecular ion peak or characteristic molecular fragments are considered.

Physical Data

Melting Points (M.p.) were determined using a melting-point-determination apparatus *Leica* Galen III hot-stage microscope from *Wagner & Munz*. The values are not corrected.

Boiling Points (B.p.) were measured at the distillation head and are not corrected. Distillation under reduced pressure was determined directly by the connected pressure gauge (VAP 5, from *Vacuumbrand*).

2 Optimization Study

General procedure for the optimization reactions. In an argon-filled glovebox, an oven-dried 1.5-mL screw-caped vial equipped with a magnetic stirring bar was charged with the indicated palladium (pre)catalyst (2.0 µmol, 2.0 mol%) and the indicated base (0.130 mmol, 1.3 equiv). Toluene (0.1 mL) was added, and the resulting suspension was stirred at room temperature for 5 min. Then, a solution of aryl (pseudo)halide (**2a–5a**,**S1**, 0.100 mmol, 1.0 equiv), (*E*)-1-(*p*-tolyl)-2-(trimethyl-silyl)diazene (**1a**, 23.1 mg, 0.120 mmol, 1.2 equiv) and a known amount of tetracosane in toluene (0.1 mL) was added in one portion, and the parent vial was rinsed with toluene (0.01 mL). The reaction mixture was stirred at 60 °C for the indicated reaction time and subsequently analyzed by GLC analysis.

Note: The absence of aryl halide or diazene led to no reaction.

Table S1. Optimization of the palladium-catalyzed cross-coupling of a masked diazenyl anion and an electron-rich aryl (pseudo)halide.[a]

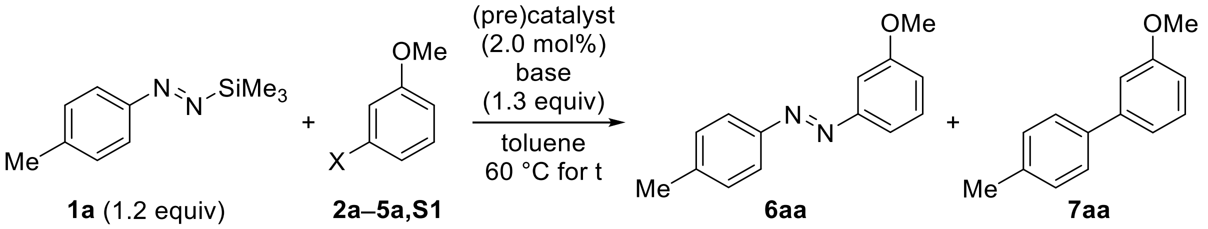

Entry	X	(Pre)catalyst	Base	*t* [h]	Yield [%] **6aa**	**7aa**
1	Br (**2a**)	Pd_2dba_3/dppf[b]	Cs_2CO_3	48	90	0
2	Br (**2a**)	(dppf)$PdCl_2$	Cs_2CO_3	15	99 (92)[c]	0
3[d]	Br (**2a**)	(dtbpf)$PdCl_2$	Cs_2CO_3	48	16	35
4[d]	Br (**2a**)	(dppe)$PdCl_2$	Cs_2CO_3	48	trace	0
5[d]	Br (**2a**)	$(PPh_3)_2PdCl_2$	Cs_2CO_3	48	trace	0
6	OTf (**3a**)	(dppf)$PdCl_2$	Cs_2CO_3	15	76	9
7	I (**4a**)	(dppf)$PdCl_2$	Cs_2CO_3	15	59	28
8[d]	Cl (**5a**)	(dppf)$PdCl_2$	Cs_2CO_3	48	0	0
9[d]	OTs (**S1**)	(dppf)$PdCl_2$	Cs_2CO_3	48	0	0
10[d]	Br (**2a**)	(dppf)$PdCl_2$	K_2CO_3	48	trace	0
11[d]	Br (**2a**)	(dppf)$PdCl_2$	CsF	24	26	trace
12	Br (**2a**)	(dppf)$PdCl_2$	CsOPiv	15	53	20
13[d]	Br (**2a**)	(dppf)$PdCl_2$	KO*t*Bu	48	0	0
14[d]	Br (**2a**)	(dppf)$PdCl_2$	NaO*t*Bu	15	63	0
15[d]	Br (**2a**)	(dppf)$PdCl_2$	LiO*t*Bu	48	0	0
16[d]	Br (**2a**)	(dppf)$PdCl_2$	Et_3N	48	0	0
17[d]	Br (**2a**)	(dppf)$PdCl_2$	—	48	0	0
18[d]	Br (**2a**)	—	Cs_2CO_3	48	0	0
19[e]	Br (**2a**)	(dppf)$PdCl_2$	Cs_2CO_3	24	99	0
20[f]	Br (**2a**)	(dppf)$PdCl_2$	Cs_2CO_3	15	87	12
21[d,g]	Br (**2a**)	(dppf)$PdCl_2$	Cs_2CO_3	48	52	0
22[h]	Br (**2a**)	(dppf)$PdCl_2$	Cs_2CO_3	15	94	0
23[i]	Br (**2a**)	(dppf)$PdCl_2$	Cs_2CO_3	48	89	5

[a] Unless otherwise noted, all reactions were performed on a 0.10 mmol scale in 0.2 mL (0.5 M) of toluene, and yields were determined by calibrated GLC analysis with tetracosane as an internal standard. [b] 1.0 mol% of Pd_2dba_3 and 3.0 mol% of dppf. [c] Yield of isolated product on a 0.20 mmol scale after flash chromatography on silica gel in parentheses. [d] Incomplete conversion of aryl (pseudo)halides **2a**–**5a**,**S1**. [e] Run at 45 °C. [f] Run at 80 °C. [g] 1.0 mol% of (dppf)$PdCl_2$. [h] 4.0 mol% of (dppf)$PdCl_2$. [i] 1.1 equiv of diazene **1a** and 1.2 equiv of Cs_2CO_3. dba = dibenzylideneacetone; dppf = 1,1'-bis(diphenylphosphino)ferrocene; dtbpf = 1,1'-bis-(di-*tert*-butylphosphino)-ferrocene; dppe = 1,2-bis(diphenylphosphino)ethane.

3 Experimental Details for the Preparation of *N*-Aryl-*N'*-Silyldiazenes

All additional *N*-aryl-*N'*-silyldiazenes were prepared according to previously reported procedures.[S1]

3.1 Synthesis of 1-(3-chlorophenyl)-2-(trimethylsilyl)hydrazine (**S2**)

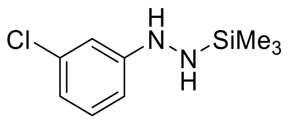

S2
$C_9H_{15}ClN_2Si$
M = 214.77 g/mol

In accordance with a reported literature procedure,[S1] a two-necked flask equipped with a magnetic stirring bar, a rubber septum, and a reflux condenser fitted with a nitrogen inlet was charged with the solid 3-chlorophenylhydrazine hydrochloride (7.00 g, 39.1 mmol, 1.0 equiv) followed by dry Et_2O (100 mL) at room temperature. The resulting suspension was vigorously stirred and Et_3N (12.7 mL, 93.8 mmol, 2.4 equiv) was added in one portion. Trimethyl-chlorosilane (Me_3SiCl, 6.0 mL, 47 mmol, 1.2 equiv) was then added neat over 10 min at room temperature. The resulting suspension was heated to reflux and vigorously stirred for 15 h (the conversion was monitored by ^{1}H NMR analysis) and then cooled to room temperature. The crude reaction mixture was then filtered over a fritted funnel, the filtrate was concentrated *in vacuo* and further evacuated under high vacuum (10^{-2} mbar) for 1 h to remove the remaining trace amount of $Et_3N{\cdot}HCl$ by sublimation. The title compound **S2** (6.57 g, 30.6 mmol, 78%) was obtained as a light yellow liquid and was used for the next step without further purification.

^{1}H NMR (500 MHz, $CDCl_3$): δ/ppm = 0.16 (s, 9H), 2.96 (bs, 1H), 5.20 (bs, 1H), 6.73 (m_c, 2H), 6.90 (app t, *J* = 2.2 Hz, 1H), 7.11 (app t, *J* = 8.0 Hz, 1H). **^{13}C{1H} NMR** (126 MHz, $CDCl_3$): δ/ppm = –1.03 (3C), 110.5, 112.1, 118.4, 130.0, 134.9, 153.3. **^{1}H/^{29}Si HMQC NMR** (500/99 MHz, $CDCl_3$, optimized for *J* = 7.0 Hz): δ/ppm = 0.16/6.84. **HRMS** (APCI) calculated for $C_9H_{15}ClN_2Si^+$ [M^+]: 214.0688; found: 214.0690. **IR** (ATR): $\tilde{\nu}$/ cm^{-1} = 3341, 2954, 1595, 1483, 1419, 1300, 1246, 1137, 1067, 988, 871, 832.

3.2 Synthesis of (*E*)-1-(3-chlorophenyl)-2-(trimethylsilyl)diazene (**1i**)

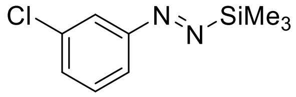

1i
$C_9H_{13}ClN_2Si$
M = 212.75 g/mol

In accordance with a reported literature procedure,[S1] in a glovebox, a 250-mL flask equipped with a magnetic stirring bar was charged with the silylated arylhydrazine (**S2**, 6.57 g, 30.6 mmol, 1.0 equiv) followed by dry *n*-pentane (60 mL). To the resulting homogeneous, vigorously stirred solution was added portion–wise the solid di-*tert*-butyl azodicarboxylate (DBAD, 6.69 g, 29.1 mmol, 0.95 equiv) at room temperature. An almost instantaneous color change from yellowish to dark blue was noted with the concomitant precipitation of the hydrazine $(BocNH)_2$. The resulting suspension was further stirred for 1 h at room temperature (the conversion was monitored by ^{1}H NMR analysis). The crude reaction mixture was then filtered in a glovebox over a fritted funnel, and the blue filtrate was concentrated *in vacuo* to afford the crude silylated aryldiazene, which was then distilled under reduced pressure *via* a short path distillation (**b.p.**: 58–60 °C at 2.8 × 10^{-1} mbar) affording the title compound **1i** (5.91 g, 27.8 mmol, 91%) as a deep blue liquid.

^{1}H NMR (500 MHz, $CDCl_3$): δ/ppm = 0.40 (s, 9H), 7.41–7.47 (m, 2H), 7.61 (m_c, 1H), 7.73 (m_c, 1H). **^{13}C{1H} NMR** (126 MHz, $CDCl_3$): δ/ppm = –2.58 (3C), 119.2, 121.2, 130.1, 130.7, 135.3, 156.8. **^{1}H/^{29}Si HMQC NMR** (500/99 MHz, $CDCl_3$, optimized for *J* = 7.0 Hz): δ/ppm = 0.40/13.9. **HRMS** (APCI) calculated for $C_9H_{14}ClN_2Si^+$ [$(M+H)^+$]: 213.0610; found: 213.0612. **IR** (ATR): $\tilde{\nu}$/cm^{-1} = 2960, 1587, 1499, 1463, 1410, 1247, 1162, 1139, 1060, 838.

4 General Procedure for the Coupling of Diazenes and Aryl Bromides (GP1)

In an argon-filled glovebox, an oven-dried 1.5-mL screw-caped vial equipped with a magnetic stirring bar was charged with (dppf)$PdCl_2$ (2.9 mg, 4.0 µmol, 2.0 mol%) and Cs_2CO_3 (84.7 mg, 0.260 mmol, 1.3 equiv). Toluene (0.2 mL) was added, and the resulting suspension was stirred at room temperature for 5 min. Then, a solution of the corresponding silylated aryldiazene (0.240 mmol, 1.2 equiv) and aryl bromide (0.200 mmol, 1.0 equiv) in toluene (0.2 mL) was added in one portion, and the parent vial was rinsed with toluene (0.02 mL). The reaction mixture was stirred at 60 °C. The conversion was monitored by GLC analysis, and unless otherwise noted, the aryl bromides were fully converted within 24 h. A color change from dark blue or dark purple to light orange was observed.

The reaction mixture was quenched by the addition of a saturated aqueous solution of NH_4Cl (5 mL), and further diluted with *tert*-butyl methyl ether (10 mL) and water (5 mL). The organic layer was separated, and the aqueous phase was extracted with *tert*-butyl methyl ether (3 x 10 mL). The combined organic layers were dried over anhydrous $MgSO_4$, filtered, and concentrated under reduced pressure. The resulting crude residue was washed thrice with *n*-pentane. Purification by flash column chromatography on silica gel using *n*-pentane: dichloromethane or *n*-pentane:*tert*-butyl methyl ether mixtures afforded the non-symmetric azobenzene derivatives.

Note: In a few cases, the *trans*-configured coupling product was isolated along with the corresponding *cis*-configured azobenzene. The latter is typically formed in trace amounts (less than 5%), notably for compounds **6gb** and **6ah**, and was not separable by column chromatography.

5 Characterization Data for the Azobenzene Derivatives

Figure S1. Overview of known and unknown azobenzene derivatives in the literature.

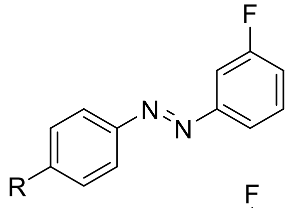

6ab (R = Me): *unknown*
6bb (R = OMe): *unknown*
6cb (R = H): *unknown*
6db (R = F): *unknown*
6eb (R = Cl): *unknown*
6fb (R = CF_3): *unknown*
6gb (R = CN): *unknown*
6hb (R = CO_2Me): *unknown*

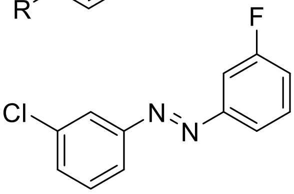

6ib: *unknown*

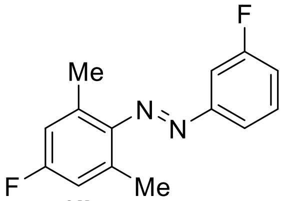

6jb: *unknown*

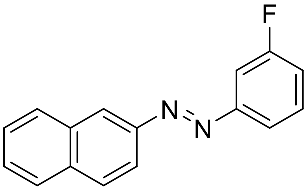

6kb: *unknown*

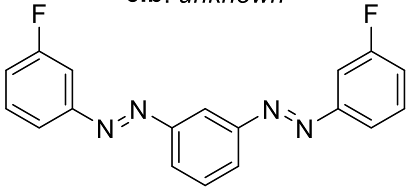

6lb: *unknown*

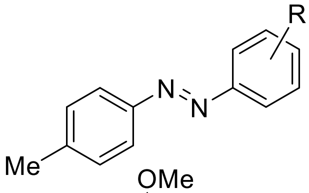

6ac (R = 3-Me): *known*
6ad (R = 2-OMe): *unknown*
6aa (R = 3-OMe): *unknown*
6ae (R = 4-OMe): *known*
6af (R = 4-NMe_2): *unknown*
6ag (R = H): *known*

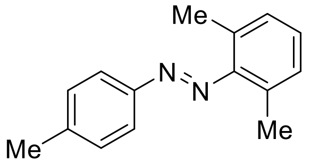

6ah: *unknown*

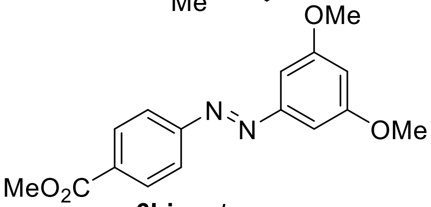

6hi: *unknown*

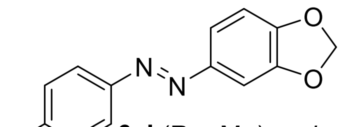

6aj (R = Me): *unknown*
6ej (R = Cl): *unknown*

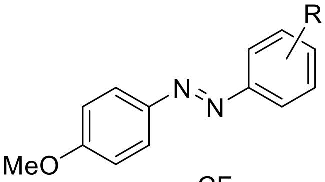

6bk (R = 4-F): *known*
6bl (R = 4-Cl): *known*
6bm (R = 4-Br): *known*
6bn (R = 4-CF_3): *unknown*
6bo (R = 4-CN): *unknown*
6bp (R = 4-NO_2): *unknown*
6bq (R = 4-CO_2Me): *unknown*
6br (R = 2-CO_2Et): *known*
6bs (R = 4-C(O)Me): *unknown*
6bt (R = 4-C(O)Ph): *unknown*

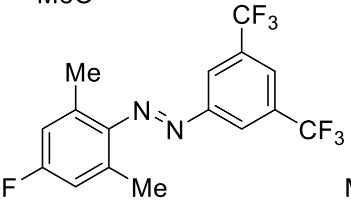

6ju: *unknown*

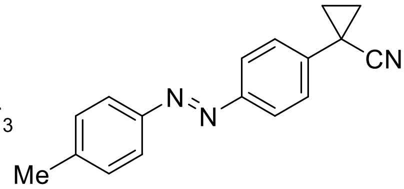

6av: *unknown*

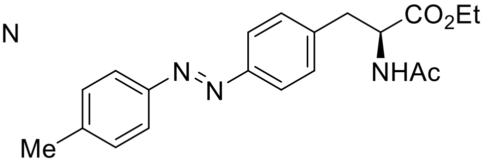

(*S*)-**6aw**: *unknown*

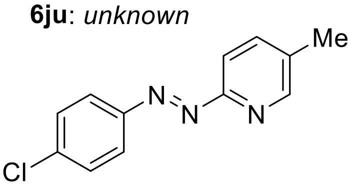

9ea: *unknown*

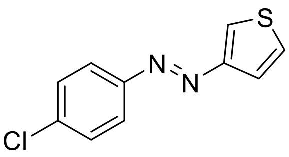

9eb: *unknown*

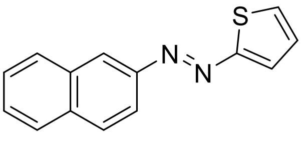

9kc: *unknown*

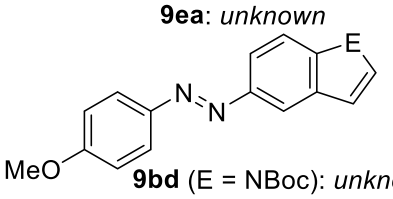

9bd (E = NBoc): *unknown*
9be (E = O): *unknown*
9bf (E = S): *unknown*

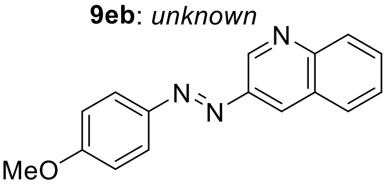

9bg: *unknown*

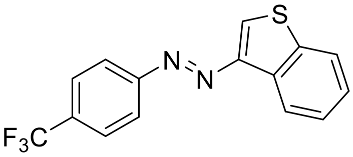

9fh: *unknown*

(*E*)-1-(3-Fluorophenyl)-2-(*p*-tolyl)diazene (6ab)

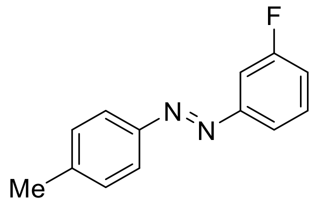

6ab
$C_{13}H_{11}FN_2$
M = 214.24 g/mol

Prepared according to **GP1** from the (*E*)-1-(*p*-tolyl)-2-(trimethylsilyl)diazene (**1a**, 46.2 mg, 0.240 mmol, 1.2 equiv) and 1-bromo-3-fluorobenzene (**2b**, 35.0 mg, 0.200 mmol, 1.0 equiv). Purification by flash column chromatography on silica gel using *n*-pentane as eluent afforded the title compound **6ab** (38.8 mg, 0.181 mmol, 91%) as a light orange solid.

R_f = 0.12 (*n*-pentane). **M.p.**: 85–87 °C (*n*-pentane). **^{1}H NMR** (500 MHz, $CDCl_3$): δ/ppm = 2.45 (s, 3H), 7.17 (m_c, 1H), 7.33 (d, *J* = 8.2 Hz, 2H), 7.49 (m_c, 1H), 7.60 (m_c, 1H), 7.74 (m_c, 1H), 7.84 (d, *J* = 8.2 Hz, 2H). **^{13}C{1H} NMR** (126 MHz, $CDCl_3$): δ/ppm = 21.7, 108.1 (d, *J* = 22.9 Hz), 117.5 (d, *J* = 22.3 Hz), 120.5 (d, *J* = 2.5 Hz), 123.2 (2C), 130.0 (2C), 130.3 (d, *J* = 8.3 Hz), 142.3, 150.7, 154.4 (d, *J* = 7.0 Hz), 163.5 (d, *J* = 247.2 Hz). **^{19}F NMR** (471 MHz, $CDCl_3$): δ/ppm = –112.2 (m_c, 1F). **HRMS** (APCI) calculated for $C_{13}H_{12}FN_2^+$ [$(M+H)^+$]: 215.0980; found: 215.0975. **IR** (ATR): $\tilde{\nu}$/cm^{-1} = 3071, 3026, 2920, 2856, 1591, 1501, 1469, 1435, 1306, 1243, 1141, 1102, 965, 873, 832.

(*E*)-1-(3-Fluorophenyl)-2-(4-methoxyphenyl)diazene (6bb)

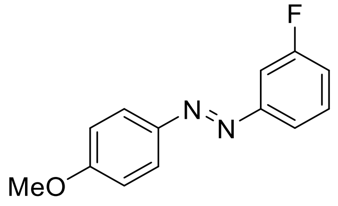

6bb
$C_{13}H_{11}FN_2O$
M = 230.24 g/mol

Prepared according to **GP1** from the (*E*)-1-(4-methoxyphenyl)-2-(trimethylsilyl)diazene (**1b**, 50.0 mg, 0.240 mmol, 1.2 equiv) and 1-bromo-3-fluorobenzene (**2b**, 35.0 mg, 0.200 mmol, 1.0 equiv). Purification by flash column chromatography on silica gel using *n*-pentane:*tert*-butyl

methyl ether (99:1) as eluent afforded the title compound **6bb** (39.5 mg, 0.172 mmol, 86%) as an orange solid.

R$_f$ = 0.65 (*n*-pentane:*tert*-butyl methyl ether 9:1). **M.p.**: 63–65 °C (*n*-pentane). **^{1}H NMR** (500 MHz, $CDCl_3$): δ/ppm = 3.90 (s, 3H), 7.02 (d, *J* = 8.6 Hz, 2H), 7.14 (m$_c$, 1H), 7.47 (m$_c$, 1H), 7.57 (m$_c$, 1H), 7.71 (m$_c$, 1H), 7.93 (d, *J* = 8.9 Hz, 2H). **^{13}C{1H} NMR** (126 MHz, $CDCl_3$): δ/ppm = 55.8, 108.0 (d, *J* = 22.5 Hz), 114.4 (2C), 117.2 (d, *J* = 21.9 Hz), 120.3 (d, *J* = 2.9 Hz), 125.2 (2C), 130.3 (d, *J* = 8.3 Hz), 146.9, 154.5 (d, *J* = 6.9 Hz), 162.6, 163.5 (d, *J* = 247.0 Hz). **^{19}F NMR** (471 MHz, $CDCl_3$): δ/ppm = –112.3 (m$_c$, 1F). **HRMS** (APCI) calculated for $C_{13}H_{12}FN_2O^+$ [(M+H)$^+$]: 231.0929; found: 231.0927. **IR** (ATR): $\tilde{\nu}$/cm^{-1} = 3067, 3013, 2965, 2841, 1579, 1495, 1472, 1440, 1315, 1236, 1181, 1143, 1103, 1024, 968, 879, 840.

(*E*)-1-(3-Fluorophenyl)-2-phenyldiazene (6cb)

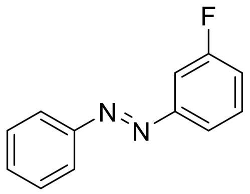

6cb
$C_{12}H_9FN_2$
M = 200.22 g/mol

Prepared according to **GP1** from the (*E*)-1-phenyl-2-(trimethylsilyl)diazene (**1c**, 42.8 mg, 0.240 mmol, 1.2 equiv) and 1-bromo-3-fluorobenzene (**2b**, 35.0 mg, 0.200 mmol, 1.0 equiv). Purification by flash column chromatography on silica gel using *n*-pentane as eluent afforded the title compound **6cb** (36.8 mg, 0.184 mmol, 92%) as an orange solid.

R$_f$ = 0.42 (*n*-pentane). **M.p.**: 52–54 °C (*n*-pentane). **^{1}H NMR** (500 MHz, $CDCl_3$): δ/ppm = 7.19 (m$_c$, 1H), 7.47–7.56 (m, 4H), 7.62 (m$_c$, 1H), 7.77 (m$_c$, 1H), 7.93 (m$_c$, 2H). **^{13}C{1H} NMR** (126 MHz, $CDCl_3$): δ/ppm = 108.2 (d, *J* = 22.9 Hz), 117.8 (d, *J* = 22.0 Hz), 120.6 (d, *J* = 2.6 Hz), 123.2 (2C), 129.3 (2C), 130.4 (d, *J* = 8.3 Hz), 131.6, 152.5, 154.3 (d, *J* = 7.1 Hz), 163.4 (d, *J* = 247.3 Hz). **^{19}F NMR** (471 MHz, $CDCl_3$): δ/ppm = –112.1 (m$_c$, 1F). **HRMS** (APCI) calculated for $C_{12}H_{10}FN_2^+$ [(M+H)$^+$]: 201.0823; found: 201.0822. **IR** (ATR): $\tilde{\nu}$/cm^{-1} = 3074, 2923, 2854, 1590, 1472, 1446, 1306, 1237, 1191, 1149, 1108, 1070, 971, 924, 886.

(*E*)-1-(3-Fluorophenyl)-2-(4-fluorophenyl)diazene (6db)

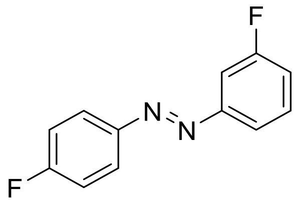

6db
$C_{12}H_8F_2N_2$
M = 218.21 g/mol

Prepared according to **GP1** from the (*E*)-1-(4-fluorophenyl)-2-(trimethylsilyl)diazene (**1d**, 47.1 mg, 0.240 mmol, 1.2 equiv) and 1-bromo-3-fluorobenzene (**2b**, 35.0 mg, 0.200 mmol, 1.0 equiv) at 45 °C. Purification by flash column chromatography on silica gel using *n*-pentane as eluent afforded the title compound **6db** (40.7 mg, 0.187 mmol, 93%) as a light orange solid.

R_f = 0.27 (*n*-pentane). **M.p.**: 96–98 °C (*n*-pentane). **^{1}H NMR** (500 MHz, $CDCl_3$): δ/ppm = 7.16–7.24 (m, 3H), 7.49 (m_c, 1H), 7.59 (m_c, 1H), 7.74 (m_c, 1H), 7.91–7.98 (m, 2H). **^{13}C{1H} NMR** (126 MHz, $CDCl_3$): δ/ppm = 108.2 (d, *J* = 22.9 Hz), 116.3 (d, *J* = 22.9 Hz, 2C), 117.9 (d, *J* = 22.1 Hz), 120.6 (d, *J* = 2.9 Hz), 125.3 (d, *J* = 9.2 Hz, 2C), 130.4 (d, *J* = 8.7 Hz), 149.1 (d, *J* = 2.9 Hz), 154.1 (d, *J* = 7.1 Hz), 163.5 (d, *J* = 247.9 Hz), 164.8 (d, *J* = 251.6 Hz). **^{19}F NMR** (471 MHz, $CDCl_3$): δ/ppm = –112.0 (m_c, 1F), –108.5 (m_c, 1F). **HRMS** (APCI) calculated for $C_{12}H_9F_2N_2^+$ [(M+H)$^+$]: 219.0729; found: 219.0727. **IR** (ATR): $\tilde{\nu}$/cm^{-1} = 3076, 2922, 2851, 1583, 1494, 1472, 1306, 1219, 1183, 1137, 1091, 972, 885, 843.

(*E*)-1-(4-Chlorophenyl)-2-(3-fluorophenyl)diazene (6eb)

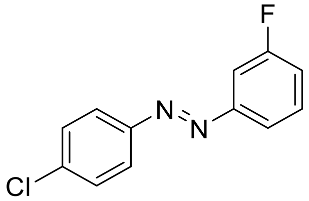

6eb
$C_{12}H_8ClFN_2$
M = 234.66 g/mol

Prepared according to **GP1** from the (*E*)-1-(4-chlorophenyl)-2-(trimethylsilyl)diazene (**1e**, 51.1 mg, 0.240 mmol, 1.2 equiv) and 1-bromo-3-fluorobenzene (**2b**, 35.0 mg, 0.200 mmol, 1.0

equiv) at 45 °C. Purification by flash column chromatography on silica gel using *n*-pentane as eluent afforded the title compound **6eb** (36.4 mg, 0.155 mmol, 78%) as a light orange solid.

R$_f$ = 0.21 (*n*-pentane). **M.p.**: 100–102 °C (*n*-pentane). **^{1}H NMR** (500 MHz, $CDCl_3$): δ/ppm = 7.20 (m$_c$, 1H), 7.47–7.52 (m, 3H), 7.60 (m$_c$, 1H), 7.75 (m$_c$, 1H), 7.88 (m$_c$, 2H). **^{13}C{1H} NMR** (126 MHz, $CDCl_3$): δ/ppm = 108.2 (app dd, *J* = 22.5 Hz, *J* = 9.1 Hz), 118.1 (app dd, *J* = 22.6 Hz, *J* = 6.3 Hz), 120.8 (d, *J* = 2.9 Hz), 124.4 (app d, *J* = 6.0 Hz, 2C), 129.6 (app d, *J* = 8.5 Hz, 2C), 130.4 (d, *J* = 8.7 Hz), 137.6, 150.8, 154.1 (d, *J* = 6.9 Hz), 162.4 (d, *J* = 248.1 Hz). **^{19}F NMR** (471 MHz, $CDCl_3$): δ/ppm = –111.9 (m$_c$, 1F). **HRMS** (APCI) calculated for $C_{12}H_9ClFN_2^+$ [(M+H)$^+$]: 235.0433; found: 235.0431. **IR** (ATR): $\tilde{\nu}$/cm^{-1} = 3083, 1590, 1473, 1431, 1398, 1235, 1083, 1002, 949, 895, 871, 839.

(*E*)-1-(3-Fluorophenyl)-2-(4-(trifluoromethyl)phenyl)diazene (6fb)

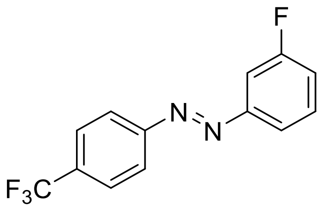

6fb
$C_{13}H_8F_4N_2$
M = 268.21 g/mol

Prepared according to **GP1** from the (*E*)-1-(4-(trifluoromethyl)phenyl)-2-(trimethylsilyl)diazene (**1f**, 59.1 mg, 0.240 mmol, 1.2 equiv) and 1-bromo-3-fluorobenzene (**2b**, 35.0 mg, 0.200 mmol, 1.0 equiv). Purification by flash column chromatography on silica gel using *n*-pentane as eluent afforded the title compound **6fb** (28.2 mg, 0.105 mmol, 53%) as an orange solid.

R$_f$ = 0.33 (*n*-pentane). **M.p.**: 66–68 °C (*n*-pentane). **^{1}H NMR** (500 MHz, $CDCl_3$): δ/ppm = 7.23 (m$_c$, 1H), 7.53 (m$_c$, 1H), 7.63 (m$_c$, 1H), 7.77–7.83 (m, 3H), 8.01 (d, *J* = 8.1 Hz, 2H). **^{13}C{1H} NMR** (126 MHz, $CDCl_3$): δ/ppm = 108.4 (d, *J* = 23.8 Hz), 118.7 (d, *J* = 22.6 Hz), 121.1 (d, *J* = 2.7 Hz), 123.3 (2C), 124.0 (q, *J* = 273.1 Hz), 126.5 (q, *J* = 3.8 Hz, 2C), 130.6 (d, *J* = 8.7 Hz), 132.8 (q, *J* = 32.4 Hz), 154.0 (d, *J* = 7.1 Hz), 154.2, 163.4 (d, *J* = 248.4 Hz). **^{19}F NMR** (471 MHz, $CDCl_3$): δ/ppm = –111.7 (m$_c$, 1F), –62.6 (s, 3F). **HRMS** (APCI) calculated for $C_{13}H_9F_4N_2^+$ [(M+H)$^+$]: 269.0697; found: 269.0690. **IR** (ATR): $\tilde{\nu}$/cm^{-1} = 3071, 2924, 1593, 1489, 1443, 1410, 1317, 1237, 1160, 1114, 1062, 1008, 952, 847.

(*E*)-4-((3-Fluorophenyl)diazenyl)benzonitrile (6gb)

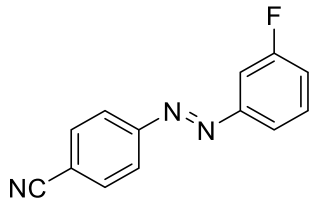

6gb
$C_{13}H_8FN_3$
M = 225.23 g/mol

Prepared according to **GP1** from the (*E*)-4-((trimethylsilyl)diazenyl)benzonitrile (**1g**, 54.2 mg, 0.240 mmol, 1.2 equiv) and 1-bromo-3-fluorobenzene (**2b**, 35.0 mg, 0.200 mmol, 1.0 equiv). The reaction time was 48 h. Purification by flash column chromatography on silica gel using *n*-pentane:*tert*-butyl methyl ether (99:1) as eluent afforded the title compound **6gb** (22.4 mg, 0.100 mmol, 50%) as a light orange solid (containing the *cis*-isomer in trace amounts).

R_f = 0.54 (*n*-pentane:*tert*-butyl methyl ether 9:1). **M.p.**: 141–143 °C (*n*-pentane). **^{1}H NMR** (500 MHz, $CDCl_3$): δ/ppm = 7.25 (m_c, 1H), 7.53 (m_c, 1H), 7.63 (m_c, 1H), 7.80 (m_c, 1H), 7.83 (d, *J* = 8.7 Hz, 2H), 8.00 (d, *J* = 8.7 Hz, 2H). **^{13}C{1H} NMR** (101 MHz, $CDCl_3$): δ/ppm = 108.4 (d, *J* = 23.1 Hz), 114.6, 118.5, 119.1 (d, *J* = 22.2 Hz), 121.3 (d, *J* = 2.8 Hz), 123.7 (2C), 130.6 (d, *J* = 8.6 Hz), 133.4 (2C), 153.9 (d, *J* = 7.1 Hz), 154.3, 163.4 (d, *J* = 248.7 Hz). **^{19}F NMR** (471 MHz, $CDCl_3$): δ/ppm = –111.5 (m_c, 1F). **HRMS** (APCI) calculated for $C_{13}H_9FN_3^+$ [$(M+H)^+$]: 226.0776; found: 226.0774. **IR** (ATR): $\tilde{\nu}$/cm^{-1} = 3086, 2922, 2850, 2230, 1587, 1468, 1423, 1404, 1289, 1241, 1193, 1151, 1104, 1069, 974, 876, 846.

Methyl (*E*)-4-((3-fluorophenyl)diazenyl)benzoate (6hb)

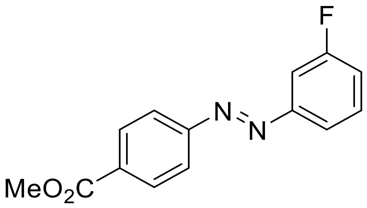

6hb
$C_{14}H_{11}FN_2O_2$
M = 258.25 g/mol

Prepared according to **GP1** from the (*E*)-4-((trimethylsilyl)diazenyl)benzoate (**1h**, 56.7 mg, 0.240 mmol, 1.2 equiv) and 1-bromo-3-fluorobenzene (**2b**, 35.0 mg, 0.200 mmol, 1.0 equiv).

Purification by flash column chromatography on silica gel using *n*-pentane:*tert*-butyl methyl ether (99:1) as eluent afforded the title compound **6hb** (38.4 mg, 0.149 mmol, 74%) as an orange solid.

R_f = 0.66 (*n*-pentane:*tert*-butyl methyl ether 9:1). **M.p.**: 137–139 °C (*n*-pentane). **^{1}H NMR** (500 MHz, $CDCl_3$): δ/ppm = 3.96 (s, 3H), 7.22 (m_c, 1H), 7.51 (m_c, 1H), 7.63 (m_c, 1H), 7.79 (m_c, 1H), 7.95 (d, *J* = 8.5 Hz, 2H), 8.02 (d, *J* = 8.5 Hz, 2H). **^{13}C{1H} NMR** (126 MHz, $CDCl_3$): δ/ppm = 52.5, 108.3 (d, *J* = 22.8 Hz), 108.6 (d, *J* = 22.1 Hz), 121.1 (d, *J* = 2.3 Hz), 123.0 (2C), 130.5 (d, *J* = 8.7 Hz), 130.8 (2C), 132.4, 154.1 (d, *J* = 6.9 Hz), 154.9, 163.4 (d, *J* = 247.8 Hz), 166.5. **^{19}F NMR** (471 MHz, $CDCl_3$): δ/ppm = –111.8 (m_c, 1F). **HRMS** (APCI) calculated for $C_{14}H_{12}FN_2O_2^+$ [(M+H)$^+$]: 259.0878; found: 259.0879. **IR** (ATR): $\tilde{\nu}$/cm^{-1} = 3080, 2960, 2852, 1722, 1588, 1472, 1437, 1314, 1276, 1236, 1192, 1152, 1099, 1004, 955, 880, 821.

(*E*)-1-(3-Chlorophenyl)-2-(3-fluorophenyl)diazene (6ib)

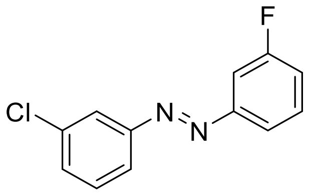

6ib
$C_{12}H_8ClFN_2$
M = 234.66 g/mol

Prepared according to **GP1** from the (*E*)-1-(3-chlorophenyl)-2-(trimethylsilyl)diazene (**1i**, 42.8 mg, 0.240 mmol, 1.2 equiv) and 1-bromo-3-fluorobenzene (**2b**, 35.0 mg, 0.200 mmol, 1.0 equiv). Purification by flash column chromatography on silica gel using *n*-pentane as eluent afforded the title compound **6ib** (33.8 mg, 0.144 mmol, 72%) as an orange solid.

R_f = 0.32 (*n*-pentane). **M.p.**: 87–89 °C (*n*-pentane). **^{1}H NMR** (500 MHz, $CDCl_3$): δ/ppm = 7.21 (m_c, 1H), 7.47 (m_c, 2H), 7.51 (m_c, 1H), 7.61 (m_c, 1H), 7.77 (m_c, 1H), 7.84 (m_c, 1H), 7.91 (m_c, 1H). **^{13}C{1H} NMR** (101 MHz, $CDCl_3$): δ/ppm = 108.3 (d, *J* = 23.1 Hz), 118.4 (d, *J* = 22.4 Hz), 120.9 (d, *J* = 2.8 Hz), 122.1 (C-2), 122.8 (C-6), 130.3 (C-4), 130.4 (d, *J* = 8.3 Hz), 131.3 (C-5), 135.4 (C-3), 153.3 (C-1), 153.9 (d, *J* = 7.1 Hz), 163.4 (d, *J* = 247.8 Hz). **^{19}F NMR** (471 MHz, $CDCl_3$): δ/ppm = –111.8 (m_c, 1F). **HRMS** (APCI) calculated for $C_{12}H_9ClFN_2^+$ [(M+H)$^+$]: 235.0433; found: 235.0434. **IR** (ATR): $\tilde{\nu}$/cm^{-1} = 3074, 2922, 2852, 1574, 1472, 1425, 1301, 1231, 1175, 1106, 1065, 996, 970, 913, 869.

(*E*)-1-(4-Fluoro-2,6-dimethylphenyl)-2-(3-fluorophenyl)diazene (6jb)

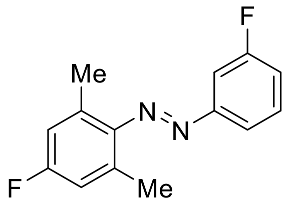

6jb
$C_{14}H_{12}F_2N_2$
M = 246.26 g/mol

Prepared according to **GP1** from the (*E*)-1-(4-fluoro-2,6-dimethylphenyl)-2-(trimethylsilyl)-diazene (**1j**, 53.8 mg, 0.240 mmol, 1.2 equiv) and 1-bromo-3-fluorobenzene (**2b**, 35.0 mg, 0.200 mmol, 1.0 equiv) at 45 °C. Purification by flash column chromatography on silica gel using *n*-pentane as eluent afforded the title compound **6jb** (36.3 mg, 0.147 mmol, 74%) as a red solid.

R_f = 0.35 (*n*-pentane). **M.p.**: 62–64 °C (*n*-pentane). **^{1}H NMR** (500 MHz, $CDCl_3$): δ/ppm = 2.43 (s, 6H), 6.85 (m_c, 2H), 7.19 (m_c, 1H), 7.50 (m_c, 1H), 7.54 (m_c, 1H), 7.72 (m_c, 1H). **^{13}C{1H} NMR** (126 MHz, $CDCl_3$): δ/ppm = 19.8 (app q, *J* = 8.2 Hz, 2C), 107.6 (app dd, *J* = 22.6 Hz, *J* = 10.0 Hz), 116.0 (d, *J* = 22.2 Hz, 2C), 117.8 (app dd, *J* = 22.3 Hz, *J* = 6.8 Hz), 120.8 (d, *J* = 6.7 Hz), 130.4, 135.0 (d, *J* = 9.1 Hz, 2C), 146.9, 154.4 (d, *J* = 6.7 Hz), 162.3 (d, *J* = 248.9 Hz), 163.5 (d, *J* = 247.7 Hz). **^{19}F NMR** (471 MHz, $CDCl_3$): δ/ppm = –113.1 (t, *J* = 10.1 Hz, 1F), –112.0 (m_c, 1F). **HRMS** (APCI) calculated for $C_{14}H_{13}F_2N_2^+$ [$(M+H)^+$]: 247.1042; found: 247.1041. **IR** (ATR): $\tilde{\nu}$/cm^{-1} = 3078, 2963, 2925, 2851, 1590, 1473, 1372, 1293, 1235, 1180, 1129, 1103, 1020, 946, 858.

(*E*)-1-(3-Fluorophenyl)-2-(naphthalen-2-yl)diazene (6kb)

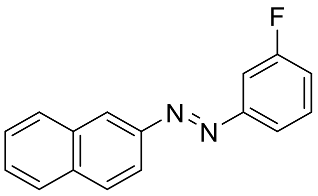

6kb
$C_{16}H_{11}FN_2$
M = 250.28 g/mol

Prepared according to **GP1** from the (*E*)-1-(naphthalen-2-yl)-2-(trimethylsilyl)diazene (**1k**, 54.8 mg, 0.240 mmol, 1.2 equiv) and 1-bromo-3-fluorobenzene (**2b**, 35.0 mg, 0.200 mmol, 1.0 equiv). Purification by flash column chromatography on silica gel using *n*-pentane:*tert*-butyl methyl ether (99:1) as eluent afforded the title compound **6kb** (43.4 mg, 0.173 mmol, 87%) as a light orange solid.

R_f = 0.15 (*n*-pentane). **M.p.**: 118–120 °C (*n*-pentane). **^{1}H NMR** (500 MHz, $CDCl_3$): δ/ppm = 7.20 (m_c, 1H), 7.52 (m_c, 1H), 7.58 (m_c, 2H), 7.68 (m_c, 1H), 7.82 (m_c, 1H), 7.90–7.94 (m, 2H), 8.03–8.08 (m, 2H), 8.49 (m_c, 1H). **^{13}C{1H} NMR** (126 MHz, $CDCl_3$): δ/ppm = 108.2 (d, *J* = 22.8 Hz), 117.0, 117.8 (d, *J* = 22.0 Hz), 120.7 (d, *J* = 2.9 Hz), 127.0, 128.0, 128.1, 128.8, 129.4, 129.6, 130.4 (d, *J* = 8.6 Hz), 133.6, 135.2, 150.1, 154.4 (d, *J* = 7.2 Hz), 163.5 (d, *J* = 246.8 Hz). **^{19}F NMR** (471 MHz, $CDCl_3$): δ/ppm = –112.1 (m_c, 1F). **HRMS** (APCI) calculated for $C_{16}H_{12}FN_2^+$ [$(M+H)^+$]: 251.0980; found: 251.0975. **IR** (ATR): $\tilde{\nu}$/cm^{-1} = 3057, 1584, 1471, 1416, 1349, 1309, 1231, 1159, 1106, 1072, 969, 903, 862, 820.

1,3-Bis((*E*)-(3-fluorophenyl)diazenyl)benzene (6lb)

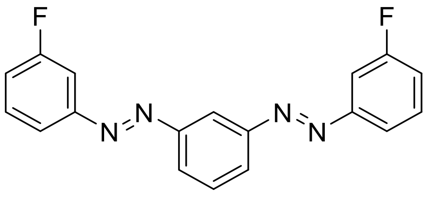

6lb
$C_{18}H_{12}F_2N_4$
M = 322.32 g/mol

Prepared according to **GP1**, in an argon-filled glovebox, an oven-dried 1.5-mL screw-caped vial equipped with a magnetic stirring bar was charged with (dppf)$PdCl_2$ (6.4 mg, 8.8 µmol, 2.0 mol%) and Cs_2CO_3 (158 mg, 0.484 mmol, 1.1 equiv). Toluene (0.2 mL) was added, and the resulting suspension was stirred at room temperature for 5 min. Then, a solution of the 1,3-bis((*E*)-(trimethylsilyl)diazenyl)benzene (**1l**, 55.1 mg, 0.200 mmol, 0.45 equiv) and 1-bromo-3-fluorobenzene (**2b**, 77.0 mg, 0.440 mmol, 1.0 equiv) in toluene (0.2 mL) was added in one portion, and the parent vial was rinsed with toluene (0.02 mL). Purification by flash column chromatography on silica gel using *n*-pentane:dichloromethane (9:1) as eluent afforded the title compound **6lb** (43.9 mg, 0.136 mmol, 68%) as a light orange solid.

R_f = 0.23 (*n*-pentane:dichloromethane 9:1). **M.p.**: 119–121 °C (*n*-pentane). **^{1}H NMR** (500 MHz, $CDCl_3$): δ/ppm = 7.22 (m_c, 2H), 7.53 (m_c, 2H), 7.66 (m_c, 2H), 7.70 (t, *J* = 7.9 Hz, 1H), 7.82 (m_c,

2H), 8.08 (dd, J = 7.9 Hz, J = 1.9 Hz, 2H), 8.45 (t, J = 1.9 Hz, 1H). **^{13}C{1H} NMR** (126 MHz, $CDCl_3$): δ/ppm = 108.4 (d, J = 23.4 Hz, 2C), 116.8, 118.3 (d, J = 22.3 Hz, 2C), 120.9 (d, J = 2.8 Hz, 2C), 126.1 (2C), 130.0, 130.5 (d, J = 8.5 Hz, 2C), 153.3 (2C), 154.1 (d, J = 6.3 Hz, 2C), 163.5 (d, J = 247.9 Hz, 2C). **^{19}F NMR** (471 MHz, $CDCl_3$): δ/ppm = –111.8 (m_c, 2F). **HRMS** (APCI) calculated for $C_{18}H_{13}F_2N_4^+$ [(M+H)$^+$]: 323.1103; found: 323.1103. **IR** (ATR): $\tilde{\nu}$/cm^{-1} = 3077, 1592, 1472, 1436, 1313, 1276, 1232, 1207, 1164, 1100, 1074, 999, 952, 901, 868.

(*E*)-1-(*m*-Tolyl)-2-(*p*-tolyl)diazene (6ac)

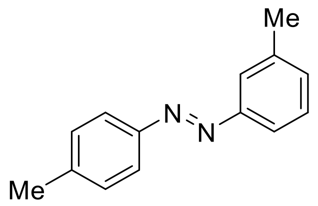

6ac
$C_{14}H_{14}N_2$
M = 210.28 g/mol

Prepared according to **GP1** from the (*E*)-1-(*p*-tolyl)-2-(trimethylsilyl)diazene (**1a**, 46.2 mg, 0.240 mmol, 1.2 equiv) and 1-bromo-3-methylbenzene (**2c**, 34.2 mg, 0.200 mmol, 1.0 equiv). Purification by flash column chromatography on silica gel using *n*-pentane:*tert*-butyl methyl ether (99:1) as eluent afforded the title compound **6ac** (39.4 mg, 0.187 mmol, 94%) as an orange solid.

R_f = 0.15 (*n*-pentane). **M.p.**: 57–59 °C (*n*-pentane). **^{1}H NMR** (400 MHz, $CDCl_3$): δ/ppm = 2.44 (s, 3H), 2.46 (s, 3H), 7.26–7.34 (m, 3H), 7.41 (m_c, 1H), 7.70–7.74 (m, 2H), 7.84 (d, J = 8.7 Hz, 2H). **^{13}C{1H} NMR** (101 MHz, $CDCl_3$): δ/ppm = 21.5, 21.6, 120.5, 122.9 (3C), 129.0, 129.9 (2C), 131.6, 139.1, 141.6, 151.0, 153.0. **HRMS** (APCI) calculated for $C_{14}H_{15}N_2^+$ [(M+H)$^+$]: 211.1230; found: 211.1230. **IR** (ATR): $\tilde{\nu}$/cm^{-1} = 3020, 2916, 2856, 1599, 1500, 1479, 1448, 1304, 1234, 1147, 1106, 1079, 1035, 1010, 912, 881, 818.

(*E*)-1-(2-Methoxyphenyl)-2-(*p*-tolyl)diazene (6ad)

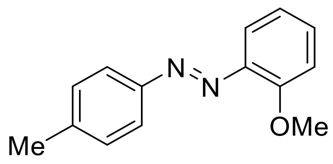

6ad
$C_{14}H_{14}N_2O$
M = 226.28 g/mol

Prepared according to **GP1** from the (*E*)-1-(*p*-tolyl)-2-(trimethylsilyl)diazene (**1a**, 46.2 mg, 0.240 mmol, 1.2 equiv) and 1-bromo-2-methoxybenzene (**2d**, 37.4 mg, 0.200 mmol, 1.0 equiv). The reaction time was 15 h. Purification by flash column chromatography on silica gel using *n*-pentane:*tert*-butyl methyl ether (99:1) as eluent afforded the title compound **6ad** (36.2 mg, 0.160 mmol, 80%) as a red solid.

R_f = 0.44 (*n*-pentane:*tert*-butyl methyl ether 9:1). **M.p.**: 65–67 °C (*n*-pentane). **^{1}H NMR** (500 MHz, $CDCl_3$): δ/ppm = 2.43 (s, 3H), 4.03 (s, 3H), 7.02 (app td, *J* = 7.5 Hz, *J* = 1.2 Hz, 1H), 7.09 (app d, *J* = 8.4 Hz, 1H), 7.30 (d, *J* = 8.2 Hz, 2H), 7.43 (app td, *J* = 7.5 Hz, *J* = 1.8 Hz, 1H), 7.66 (dd, *J* = 8.0 Hz, *J* = 1.7 Hz, 1H), 7.84 (d, *J* = 8.2 Hz, 2H). **^{13}C{1H} NMR** (126 MHz, $CDCl_3$): δ/ppm = 21.6, 56.5, 112.9, 117.1, 120.9, 123.1 (2C), 129.8 (2C), 132.2, 141.4, 142.6, 151.4, 157.0. **HRMS** (APCI) calculated for $C_{14}H_{15}N_2O^+$ $[(M+H)^+]$: 227.1179; found: 227.1179. **IR** (ATR): $\tilde{\nu}$/cm^{-1} = 2959, 2931, 2832, 1591, 1482, 1427, 1306, 1277, 1242, 1185, 1152, 1107, 1044, 1017, 943, 816.

(*E*)-1-(3-Methoxyphenyl)-2-(*p*-tolyl)diazene (6aa)

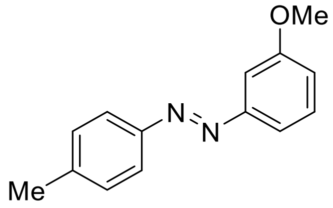

6aa
$C_{14}H_{14}N_2O$
M = 226.28 g/mol

Prepared according to **GP1** from the (*E*)-1-(*p*-tolyl)-2-(trimethylsilyl)diazene (**1a**, 46.2 mg, 0.240 mmol, 1.2 equiv) and 1-bromo-3-methoxybenzene (**2a**, 37.4 mg, 0.200 mmol, 1.0 equiv). The reaction time was 15 h. Purification by flash column chromatography on silica gel using *n*-

pentane:*tert*-butyl methyl ether (99:1) as eluent afforded the title compound **6aa** (41.5 mg, 0.183 mmol, 92%) as an orange solid.

R$_f$ = 0.71 (*n*-pentane:*tert*-butyl methyl ether 9:1). **M.p.**: 81–83 °C (*n*-pentane). **^{1}H NMR** (500 MHz, $CDCl_3$): δ/ppm = 2.45 (s, 3H), 3.91 (s, 3H), 7.04 (app dd, *J* = 8.2 Hz, *J* = 2.5 Hz, 1H), 7.33 (d, *J* = 8.3 Hz, 2H), 7.43 (app t, *J* = 7.9 Hz, 1H), 7.46 (app t, *J* = 2.1 Hz, 1H), 7.56 (app d, *J* = 7.9 Hz, 1H), 7.85 (d, *J* = 8.3 Hz, 2H). **^{13}C{1H} NMR** (126 MHz, $CDCl_3$): δ/ppm = 21.6, 55.6, 105.9, 117.1, 117.6, 123.0 (2C), 129.86, 129.88 (2C), 141.7, 150.9, 154.1, 160.5. **HRMS** (APCI) calculated for $C_{14}H_{15}N_2O^+$ [(M+H)$^+$]: 227.1179; found: 227.1180. **IR** (ATR): $\tilde{\nu}$/cm^{-1} = 3021, 2956, 2920, 2835, 1583, 1477, 1427, 1312, 1254, 1147, 1109, 1037, 951, 873, 819.

(*E*)-1-(4-Methoxyphenyl)-2-(*p*-tolyl)diazene (6ae)

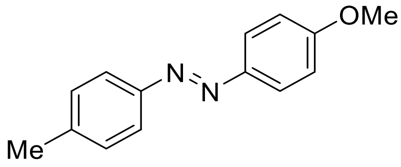

6ae
$C_{14}H_{14}N_2O$
M = 226.28 g/mol

Prepared according to **GP1** from the (*E*)-1-(*p*-tolyl)-2-(trimethylsilyl)diazene (**1a**, 46.2 mg, 0.240 mmol, 1.2 equiv) and 1-bromo-4-methoxybenzene (**2e**, 37.4 mg, 0.200 mmol, 1.0 equiv). The reaction time was 15 h. Purification by flash column chromatography on silica gel using *n*-pentane:*tert*-butyl methyl ether (99:1) as eluent afforded the title compound **6ae** (44.2 mg, 0.195 mmol, 98%) as a light orange solid.

R$_f$ = 0.61 (*n*-pentane:*tert*-butyl methyl ether 9:1). **M.p.**: 114–116 °C (*n*-pentane). **^{1}H NMR** (400 MHz, $CDCl_3$): δ/ppm = 2.44 (s, 3H), 3.89 (s, 3H), 7.02 (d, *J* = 8.9 Hz, 2H), 7.31 (d, *J* = 8.1 Hz, 2H), 7.80 (d, *J* = 8.1 Hz, 2H), 7.92 (d, *J* = 8.9 Hz, 2H). **^{13}C{1H} NMR** (101 MHz, $CDCl_3$): δ/ppm = 21.6, 55.7, 114.3 (2C), 122.7 (2C), 124.7 (2C), 129.8 (2C), 140.9, 147.2, 151.0, 162.0. **HRMS** (APCI) calculated for $C_{14}H_{15}N_2O^+$ [(M+H)$^+$]: 227.1179; found: 227.1179. **IR** (ATR): $\tilde{\nu}$/cm^{-1} = 2922, 2842, 1578, 1493, 1450, 1322, 1292, 1243, 1175, 1137, 1100, 1024, 836.

(*E*)-*N*,*N*-Dimethyl-4-(*p*-tolyldiazenyl)aniline (6af)

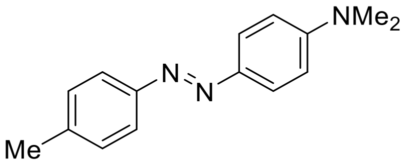

6af
$C_{15}H_{17}N_3$
M = 239.32 g/mol

Prepared according to **GP1** from the (*E*)-1-(*p*-tolyl)-2-(trimethylsilyl)diazene (**1a**, 46.2 mg, 0.240 mmol, 1.2 equiv) and 4-bromo-*N*,*N*-dimethylaniline (**2f**, 40.0 mg, 0.200 mmol, 1.0 equiv). Purification by flash column chromatography on silica gel using *n*-pentane:*tert*-butyl methyl ether (99:1) as eluent afforded the title compound **6af** (39.8 mg, 0.166 mmol, 83%) as an orange solid.

R_f = 0.44 (*n*-pentane:*tert*-butyl methyl ether 9:1). **M.p.**: 175–177 °C (*n*-pentane). **^{1}H NMR** (500 MHz, $CDCl_3$): δ/ppm = 2.41 (s, 3H), 3.08 (s, 6H), 6.76 (d, *J* = 9.2 Hz, 2H), 7.27 (d, *J* = 8.4 Hz, 2H), 7.75 (d, *J* = 8.4 Hz, 2H), 7.87 (d, *J* = 9.2 Hz, 2H). **^{13}C{1H} NMR** (126 MHz, $CDCl_3$): δ/ppm = 21.5, 40.5 (2C), 111.7 (2C), 122.3 (2C), 124.9 (2C), 129.7 (2C), 139.8, 143.9, 151.4, 152.4. **HRMS** (APCI) calculated for $C_{15}H_{18}N_3^+$ [$(M+H)^+$]: 240.1496; found: 240.1495. **IR** (ATR): $\tilde{\nu}$/cm^{-1} = 2911, 2853, 2816, 1595, 1516, 1442, 1395, 1364, 1229, 1136, 1065, 1035, 943, 811.

(*E*)-1-Phenyl-2-(*p*-tolyl)diazene (6ag)

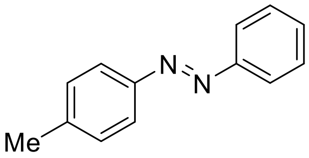

6ag
$C_{13}H_{12}N_2$
M = 196.25 g/mol

Prepared according to **GP1** from the (*E*)-1-(*p*-tolyl)-2-(trimethylsilyl)diazene (**1a**, 46.2 mg, 0.240 mmol, 1.2 equiv) and bromobenzene (**2g**, 31.4 mg, 0.200 mmol, 1.0 equiv). Purification by flash column chromatography on silica gel using *n*-pentane:*tert*-butyl methyl ether (99:1) as eluent afforded the title compound **6ag** (37.2 mg, 0.190 mmol, 95%) as an orange solid.

R$_f$ = 0.10 (*n*-pentane). **M.p.**: 81–83 °C (*n*-pentane). **^{1}H NMR** (400 MHz, $CDCl_3$): δ/ppm = 2.45 (s, 3H), 7.33 (d, *J* = 8.2 Hz, 2H), 7.44–7.55 (m, 3H), 7.85 (d, *J* = 8.2 Hz, 2H), 7.92 (app d, *J* = 8.4 Hz, 2H). **^{13}C{1H} NMR** (101 MHz, $CDCl_3$): δ/ppm = 21.6, 122.9 (2C), 123.0 (2C), 129.2 (2C), 129.9 (2C), 130.8, 141.7, 151.0, 152.9. **HRMS** (APCI) calculated for $C_{13}H_{13}N_2^+$ [(M+H)$^+$]: 197.1074; found: 197.1073. **IR** (ATR): $\tilde{\nu}$/cm^{-1} = 3033, 2919, 2854, 1599, 1500, 1440, 1407, 1298, 1218, 1149, 1106, 1068, 1036, 1013, 918, 820.

(*E*)-1-(2,6-Dimethylphenyl)-2-(*p*-tolyl)diazene (6ah)

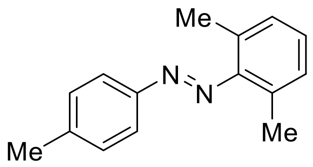

6ah
$C_{15}H_{16}N_2$
M = 224.31 g/mol

Prepared according to **GP1** from the (*E*)-1-(*p*-tolyl)-2-(trimethylsilyl)diazene (**1a**, 46.2 mg, 0.240 mmol, 1.2 equiv) and 2-bromo-1,3-dimethylbenzene (**2h**, 37.0 mg, 0.200 mmol, 1.0 equiv). The reaction was set up at 80 °C with (dppf)$PdCl_2$ (5.9 mg, 8.0 µmol, 4.0 mol%). Purification by flash column chromatography on silica gel using *n*-pentane:*tert*-butyl methyl ether (99:1) as eluent afforded the title compound **6ah** (31.7 mg, 0.141 mmol, 71%) as a red oil (containing the *cis*-isomer in trace amounts).

R$_f$ = 0.78 (*n*-pentane:*tert*-butyl methyl ether 9:1). **^{1}H NMR** (500 MHz, $CDCl_3$): δ/ppm = 2.33 (s, 6H), 2.45 (s, 3H), 7.10–7.16 (m, 3H), 7.33 (d, *J* = 8.4 Hz, 2H), 7.82 (d, *J* = 8.4 Hz, 2H). **^{13}C{1H} NMR** (101 MHz, $CDCl_3$): δ/ppm = 18.8 (2C), 21.6, 122.6 (2C), 128.0 (2C), 129.2 (2C), 129.9 (2C), 130.5, 141.7, 151.0, 151.8. **HRMS** (APCI) calculated for $C_{15}H_{17}N_2^+$ [(M+H)$^+$]: 225.1387; found: 225.1385. **IR** (ATR): $\tilde{\nu}$/cm^{-1} = 3024, 2955, 2920, 2853, 1601, 1502, 1461, 1376, 1146, 1103, 1033, 821.

Methyl (*E*)-4-((3,5-dimethoxyphenyl)diazenyl)benzoate (6hi)

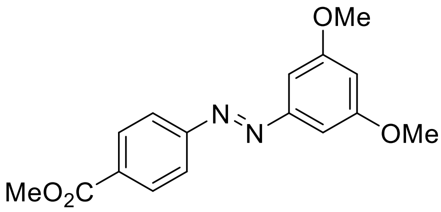

6hi
$C_{16}H_{16}N_2O_4$
M = 300.31 g/mol

Prepared according to **GP1** from the (*E*)-4-((trimethylsilyl)diazenyl)benzoate (**1h**, 56.7 mg, 0.240 mmol, 1.2 equiv) and 1-bromo-3,5-dimethoxybenzene (**2i**, 43.4 mg, 0.200 mmol, 1.0 equiv). Purification by flash column chromatography on silica gel using *n*-pentane:*tert*-butyl methyl ether (9:1) as eluent afforded the title compound **6hi** (44.4 mg, 0.148 mmol, 74%) as a red solid.

R_f = 0.35 (*n*-pentane:*tert*-butyl methyl ether 9:1). **M.p.**: 115–117 °C (*n*-pentane). **^{1}H NMR** (500 MHz, $CDCl_3$): δ/ppm = 3.88 (s, 6H), 3.96 (s, 3H), 6.64 (t, *J* = 2.3 Hz, 1H), 7.15 (d, *J* = 2.3 Hz, 2H), 7.94 (d, *J* = 8.2 Hz, 2H), 8.19 (d, *J* = 8.2 Hz, 2H). **^{13}C{1H} NMR** (126 MHz, $CDCl_3$): δ/ppm = 52.5, 55.8 (2C), 101.4 (2C), 104.8, 122.8 (2C), 130.8 (2C), 132.0, 154.5, 155.2, 161.3 (2C), 166.7. **HRMS** (APCI) calculated for $C_{16}H_{17}N_2O_4^+$ [$(M+H)^+$]: 301.1183; found: 301.1182. **IR** (ATR): $\tilde{\nu}$/cm^{-1} = 3078, 2989, 2945, 2838, 1717, 1600, 1454, 1424, 1333, 1304, 1269, 1192, 1148, 1109, 1060, 856.

(*E*)-1-(Benzo[*d*][1,3]dioxol-5-yl)-2-(*p*-tolyl)diazene (6aj)

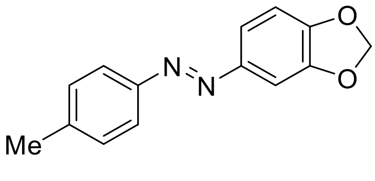

6aj
$C_{14}H_{12}N_2O_2$
M = 240.26 g/mol

Prepared according to **GP1** from the (*E*)-1-(*p*-tolyl)-2-(trimethylsilyl)diazene (**1a**, 46.2 mg, 0.240 mmol, 1.2 equiv) and 5-bromobenzo[*d*][1,3]dioxole (**2j**, 40.2 mg, 0.200 mmol, 1.0 equiv). Purification by flash column chromatography on silica gel using *n*-pentane:*tert*-butyl methyl

ether (99:1) as eluent afforded the title compound **6aj** (45.6 mg, 0.190 mmol, 95%) as a yellow solid.

R$_f$ = 0.67 (*n*-pentane:*tert*-butyl methyl ether 9:1). **M.p.**: 170–172 °C (*n*-pentane). **^{1}H NMR** (400 MHz, CDCl$_3$): δ/ppm = 2.43 (s, 3H), 6.06 (s, 2H), 6.95 (d, *J* = 8.3 Hz, 1H), 7.30 (d, *J* = 8.3 Hz, 2H), 7.47 (d, *J* = 2.0 Hz, 1H), 7.59 (dd, *J* = 8.3 Hz, *J* = 2.0 Hz, 1H), 7.80 (d, *J* = 8.3 Hz, 2H). **^{13}C{1H} NMR** (101 MHz, CDCl$_3$): δ/ppm = 21.6, 99.3, 102.0, 108.1, 122.8 (2C), 123.6, 129.9 (2C), 141.3, 148.6, 148.9, 150.3, 150.5. **HRMS** (APCI) calculated for $C_{14}H_{13}N_2O_2^+$ [(M+H)$^+$]: 241.0972; found: 241.0972. **IR** (ATR): $\tilde{\nu}$/cm^{-1} = 2905, 2789, 1598, 1498, 1461, 1413, 1371, 1236, 1076, 1027, 925.

(*E*)-1-(Benzo[*d*][1,3]dioxol-5-yl)-2-(4-chlorophenyl)diazene (6ej)

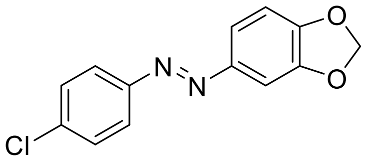

6ej
$C_{13}H_9ClN_2O_2$
M = 260.68 g/mol

Prepared according to **GP1** from the (*E*)-1-(4-chlorophenyl)-2-(trimethylsilyl)diazene (**1e**, 51.1 mg, 0.240 mmol, 1.2 equiv) and 5-bromobenzo[*d*][1,3]dioxole (**2j**, 40.2 mg, 0.200 mmol, 1.0 equiv). Purification by flash column chromatography on silica gel using *n*-pentane:*tert*-butyl methyl ether (99:1) as eluent afforded the title compound **6ej** (42.4 mg, 0.163 mmol, 81%) as a yellow solid.

R$_f$ = 0.68 (*n*-pentane:*tert*-butyl methyl ether 9:1). **M.p.**: 166–168 °C (*n*-pentane). **^{1}H NMR** (500 MHz, CDCl$_3$): δ/ppm = 6.07 (s, 2H), 6.95 (d, *J* = 8.2 Hz, 1H), 7.42 (d, *J* = 1.9 Hz, 1H), 7.46 (d, *J* = 8.7 Hz, 2H), 7.58 (dd, *J* = 8.2 Hz, *J* = 1.9 Hz, 1H), 7.82 (d, *J* = 8.7 Hz, 2H). **^{13}C{1H} NMR** (126 MHz, CDCl$_3$): δ/ppm = 99.1, 102.1, 108.2, 124.0 (2C), 124.2, 129.4 (2C), 136.5, 148.6, 149.0, 150.7, 151.1. **HRMS** (APCI) calculated for $C_{13}H_{10}ClN_2O_2^+$ [(M+H)$^+$]: 261.0426; found: 261.0425. **IR** (ATR): $\tilde{\nu}$/cm^{-1} = 2907, 1572, 1499, 1463, 1414, 1371, 1236, 1079, 1026, 925.

(*E*)-1-(4-Fluorophenyl)-2-(4-methoxyphenyl)diazene (6bk)

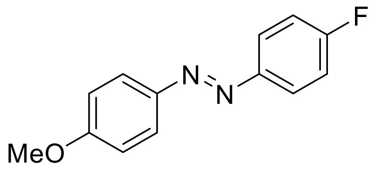

6bk
$C_{13}H_{11}FN_2O$
M = 230.24 g/mol

Prepared according to **GP1** from the (*E*)-1-(4-methoxyphenyl)-2-(trimethylsilyl)diazene (**1b**, 50.0 mg, 0.240 mmol, 1.2 equiv) and 1-bromo-4-fluorobenzene (**2k**, 35.0 mg, 0.200 mmol, 1.0 equiv). Purification by flash column chromatography on silica gel using *n*-pentane:*tert*-butyl methyl ether (99:1) as eluent afforded the title compound **6bk** (37.3 mg, 0.162 mmol, 81%) as an orange solid.

$\mathbf{R_f}$ = 0.60 (*n*-pentane:*tert*-butyl methyl ether 9:1). **M.p.**: 96–98 °C (*n*-pentane). ^{1}**H NMR** (500 MHz, $CDCl_3$): δ/ppm = 3.89 (s, 3H), 7.02 (m_c, 2H), 7.18 (m_c, 2H), 7.87–7.93 (m, 4H). ^{13}**C{1H} NMR** (126 MHz, $CDCl_3$): δ/ppm = 55.7, 114.4 (2C), 116.1 (d, *J* = 23.0 Hz, 2C), 124.6 (d, *J* = 8.9 Hz, 2C), 124.8 (2C), 147.0, 149.4 (d, *J* = 2.7 Hz), 162.2, 164.1 (d, *J* = 251.4 Hz). ^{19}**F NMR** (471 MHz, $CDCl_3$): δ/ppm = –110.5 (m_c, 1F). **HRMS** (APCI) calculated for $C_{13}H_{12}FN_2O^+$ [$(M+H)^+$]: 231.0929; found: 231.0928. **IR** (ATR): $\tilde{\nu}$/cm^{-1} = 3013, 2967, 2842, 1577, 1487, 1455, 1417, 1319, 1247, 1222, 1141, 1106, 1025, 946, 843.

(*E*)-1-(4-Chlorophenyl)-2-(4-methoxyphenyl)diazene (6bl)

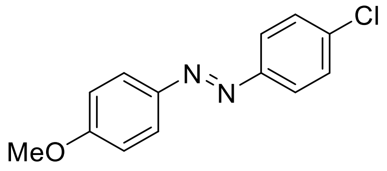

6bl
$C_{13}H_{11}ClN_2O$
M = 246.69 g/mol

Prepared according to **GP1** from the (*E*)-1-(4-methoxyphenyl)-2-(trimethylsilyl)diazene (**1b**, 50.0 mg, 0.240 mmol, 1.2 equiv) and 1-chloro-4-fluorobenzene (**2l**, 38.3 mg, 0.200 mmol, 1.0 equiv). Purification by flash column chromatography on silica gel using *n*-pentane:

dichloromethane (9:1) as eluent afforded the title compound **6bl** (40.0 mg, 0.162 mmol, 81%) as a light orange solid.

R_f = 0.15 (*n*-pentane:dichloromethane 9:1). **M.p.**: 127–129 °C (*n*-pentane). **^{1}H NMR** (400 MHz, $CDCl_3$): δ/ppm = 3.89 (s, 3H), 7.02 (d, *J* = 9.0 Hz, 2H), 7.46 (d, *J* = 8.7 Hz, 2H), 7.83 (d, *J* = 8.7 Hz, 2H), 7.92 (d, *J* = 9.0 Hz, 2H). **^{13}C{1H} NMR** (101 MHz, $CDCl_3$): δ/ppm = 55.7, 114.4 (2C), 124.0 (2C), 125.0 (2C), 129.4 (2C), 136.3, 147.0, 151.3, 162.4. **HRMS** (APCI) calculated for $C_{13}H_{12}ClN_2O^+$ [(M+H)$^+$]: 247.0633; found: 247.0632. **IR** (ATR): $\tilde{\nu}$/cm^{-1} = 3010, 2963, 2925, 2839, 1599, 1570, 1494, 1451, 1413, 1324, 1251, 1140, 1091, 1025, 841.

(*E*)-1-(4-Bromophenyl)-2-(4-methoxyphenyl)diazene (6bm)

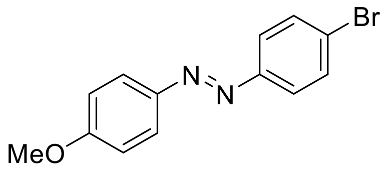

6bm
$C_{13}H_{11}BrN_2O$
M = 291.15 g/mol

Prepared according to **GP1** from the (*E*)-1-(4-methoxyphenyl)-2-(trimethylsilyl)diazene (**1b**, 50.0 mg, 0.240 mmol, 1.2 equiv) and 1-bromo-4-iodobenzene (**4m**, 56.6 mg, 0.200 mmol, 1.0 equiv). Purification by flash column chromatography on silica gel using *n*-pentane: dichloromethane (95:5) as eluent afforded the title compound **6bm** (41.1 mg, 0.141 mmol, 71%) as a light orange solid.

R_f = 0.13 (*n*-pentane:dichloromethane 9:1). **M.p.**: 154–156 °C (*n*-pentane). **^{1}H NMR** (500 MHz, $CDCl_3$): δ/ppm = 3.89 (s, 3H), 7.02 (d, *J* = 9.0 Hz, 2H), 7.63 (d, *J* = 8.7 Hz, 2H), 7.76 (d, *J* = 8.7 Hz, 2H), 7.92 (d, *J* = 9.0 Hz, 2H). **^{13}C{1H} NMR** (126 MHz, $CDCl_3$): δ/ppm = 55.8, 114.4 (2C), 124.2 (2C), 124.7, 125.1 (2C), 132.4 (2C), 147.0, 151.7, 162.5. **HRMS** (APCI) calculated for $C_{13}H_{12}BrN_2O^+$ [(M+H)$^+$]: 291.0128; found: 291.0127. **IR** (ATR): $\tilde{\nu}$/cm^{-1} = 3008, 2963, 2928, 2837, 1597, 1578, 1494, 1449, 1393, 1322, 1296, 1251, 1178, 1140, 1103, 1063, 1025, 841.

(*E*)-1-(4-Methoxyphenyl)-2-(4-(trifluoromethyl)phenyl)diazene (6bn)

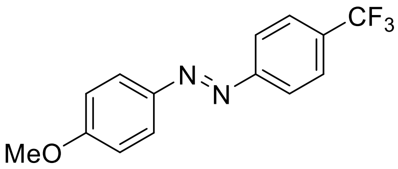

6bn
$C_{14}H_{11}F_3N_2O$
M = 280.25 g/mol

Prepared according to **GP1** from the (*E*)-1-(4-methoxyphenyl)-2-(trimethylsilyl)diazene (**1b**, 50.0 mg, 0.240 mmol, 1.2 equiv) and 1-bromo-4-(trifluoromethyl)benzene (**2n**, 45.0 mg, 0.200 mmol, 1.0 equiv). Purification by flash column chromatography on silica gel using *n*-pentane: dichloromethane (9:1) as eluent afforded the title compound **6bn** (48.1 mg, 0.172 mmol, 86%) as a light orange solid.

R_f = 0.10 (*n*-pentane:dichloromethane 9:1). **M.p.**: 133–135 °C (*n*-pentane). **^{1}H NMR** (400 MHz, $CDCl_3$): δ/ppm = 3.91 (s, 3H), 7.03 (d, *J* = 8.9 Hz, 2H), 7.76 (d, *J* = 8.4 Hz, 2H), 7.96 (app d, *J* = 8.9 Hz, 4H). **^{13}C{1H} NMR** (101 MHz, $CDCl_3$): δ/ppm = 55.8, 114.5 (2C), 122.9 (2C), 124.2 (q, *J* = 272.3 Hz), 125.4 (2C), 126.4 (q, *J* = 3.6 Hz, 2C), 131.7 (q, *J* = 32.5 Hz), 147.0, 154.8, 162.9. **^{19}F NMR** (471 MHz, $CDCl_3$): δ/ppm = –62.5 (s, 3F). **HRMS** (APCI) calculated for $C_{14}H_{12}F_3N_2O^+$ [$(M+H)^+$]: 281.0897; found: 281.0894. **IR** (ATR): $\tilde{\nu}$/cm^{-1} = 3033, 2967, 2844, 1598, 1578, 1498, 1457, 1405, 1319, 1253, 1164, 1120, 1097, 1062, 1025, 844.

(*E*)-4-((4-Methoxyphenyl)diazenyl)benzonitrile (6bo)

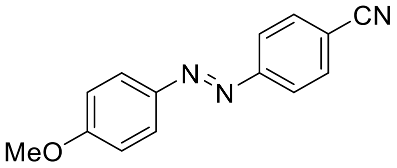

6bo
$C_{14}H_{11}N_3O$
M = 237.26 g/mol

Prepared according to **GP1** from the (*E*)-1-(4-methoxyphenyl)-2-(trimethylsilyl)diazene (**1b**, 50.0 mg, 0.240 mmol, 1.2 equiv) and 4-bromobenzonitrile (**2o**, 36.4 mg, 0.200 mmol, 1.0 equiv). Purification by flash column chromatography on silica gel using *n*-pentane:

dichloromethane (1:1) as eluent afforded the title compound **6bo** (42.2 mg, 0.178 mmol, 89%) as an orange solid.

R$_f$ = 0.37 (*n*-pentane:dichloromethane 1:1). **M.p.**: 153–155 °C (*n*-pentane). **^{1}H NMR** (400 MHz, $CDCl_3$): δ/ppm = 3.91 (s, 3H), 7.03 (d, *J* = 9.0 Hz, 2H), 7.78 (d, *J* = 8.6 Hz, 2H), 7.94 (d, *J* = 8.6 Hz, 2H), 7.95 (d, *J* = 9.0 Hz, 2H). **^{13}C{1H} NMR** (101 MHz, $CDCl_3$): δ/ppm = 55.8, 113.3, 114.6 (2C), 118.8, 123.2 (2C), 125.6 (2C), 133.3 (2C), 147.0, 154.9, 163.2. **HRMS** (APCI) calculated for $C_{14}H_{12}N_3O^+$ [$(M+H)^+$]: 238.0975; found: 238.0976. **IR** (ATR): $\tilde{\nu}$/cm^{-1} = 3054, 2945, 2919, 2841, 2218, 1579, 1494, 1445, 1396, 1291, 1245, 1184, 1133, 1107, 1027, 844.

(*E*)-1-(4-Methoxyphenyl)-2-(4-nitrophenyl)diazene (6bp)

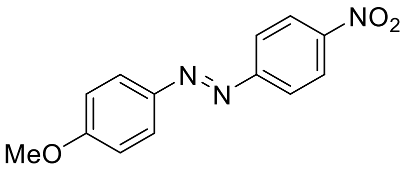

6bp
$C_{13}H_{11}N_3O_3$
M = 257.25 g/mol

Prepared according to **GP1** from the (*E*)-1-(4-methoxyphenyl)-2-(trimethylsilyl)diazene (**1b**, 50.0 mg, 0.240 mmol, 1.2 equiv) and methyl 4-bromobenzoate (**2p**, 40.4 mg, 0.200 mmol, 1.0 equiv). Purification by flash column chromatography on silica gel using *n*-pentane: dichloromethane (7:3) as eluent afforded the title compound **6bp** (37.5 mg, 0.146 mmol, 73%) as an orange solid.

R$_f$ = 0.15 (*n*-pentane:dichloromethane 7:3). **M.p.**: 166–168 °C (*n*-pentane). **^{1}H NMR** (400 MHz, $CDCl_3$): δ/ppm = 3.92 (s, 3H), 7.04 (d, *J* = 9.0 Hz, 2H), 7.98 (m_c, 4H), 8.36 (d, *J* = 9.0 Hz, 2H). **^{13}C{1H} NMR** (101 MHz, $CDCl_3$): δ/ppm = 55.8, 114.6 (2C), 123.3 (2C), 124.8 (2C), 125.8 (2C), 147.1, 148.4, 156.2, 163.4. **HRMS** (APCI) calculated for $C_{13}H_{12}N_3O_3^+$ [$(M+H)^+$]: 258.0874; found: 258.0871. **IR** (ATR): $\tilde{\nu}$/cm^{-1} = 3100, 2928, 2839, 1598, 1578, 1497, 1450, 1415, 1324, 1245, 1180, 1135, 1101, 1024, 857, 826.

Methyl (*E*)-4-((4-methoxyphenyl)diazenyl)benzoate (6bq)

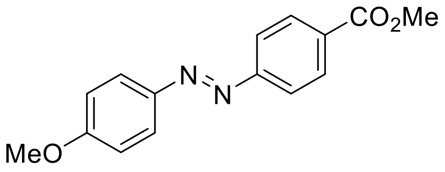

6bq
$C_{15}H_{14}N_2O_3$
M = 270.29 g/mol

Prepared according to **GP1** from the (*E*)-1-(4-methoxyphenyl)-2-(trimethylsilyl)diazene (**1b**, 50.0 mg, 0.240 mmol, 1.2 equiv) and 1-bromo-4-nitrobenzene (**2q**, 43.0 mg, 0.200 mmol, 1.0 equiv). Purification by flash column chromatography on silica gel using *n*-pentane: dichloromethane (1:1) as eluent afforded the title compound **6bq** (41.8 mg, 0.155 mmol, 78%) as an orange solid.

R_f = 0.16 (*n*-pentane:dichloromethane 1:1). **M.p.**: 174–176 °C (*n*-pentane). **^{1}H NMR** (400 MHz, $CDCl_3$): δ/ppm = 3.90 (s, 3H), 3.95 (s, 3H), 7.03 (d, *J* = 9.1 Hz, 2H), 7.91 (d, *J* = 8.4 Hz, 2H), 7.95 (d, *J* = 9.1 Hz, 2H), 8.17 (d, *J* = 8.4 Hz, 2H). **^{13}C{1H} NMR** (101 MHz, $CDCl_3$): δ/ppm = 52.4, 55.8, 114.5 (2C), 122.5 (2C), 125.3 (2C), 130.7 (2C), 131.1, 147.2, 155.5, 162.8, 166.8. **HRMS** (APCI) calculated for $C_{15}H_{15}N_2O_3^+$ [(M+H)$^+$]: 271.1078; found: 271.1078. **IR** (ATR): $\tilde{\nu}$/cm^{-1} = 3000, 2947, 2841, 1710, 1598, 1578, 1495, 1430, 1402, 1277, 1246, 1190, 1140, 1102, 1022, 958, 864, 838, 825.

Ethyl (*E*)-2-((4-methoxyphenyl)diazenyl)benzoate (6br)

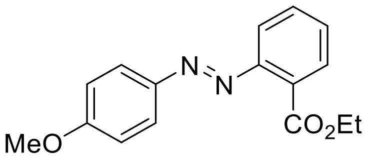

6br
$C_{16}H_{16}N_2O_3$
M = 284.32 g/mol

Prepared according to **GP1** from the (*E*)-1-(4-methoxyphenyl)-2-(trimethylsilyl)diazene (**1b**, 50.0 mg, 0.240 mmol, 1.2 equiv) and ethyl 2-bromobenzoate (**2r**, 45.8 mg, 0.200 mmol, 1.0 equiv). Purification by flash column chromatography on silica gel using *n*-pentane:*tert*-butyl methyl ether (98:2) as eluent afforded the title compound **6br** (46.4 mg, 0.163 mmol, 82%) as a red liquid.

R_f = 0.53 (*n*-pentane:*tert*-butyl methyl ether 9:1). **^{1}H NMR** (500 MHz, $CDCl_3$): δ/ppm = 1.31 (t, *J* = 7.3 Hz, 3H), 2.44 (s, 3H), 1.31 (q, *J* = 7.3 Hz, 2H), 7.32 (d, *J* = 8.2 Hz, 2H), 7.45–7.49 (m, 1H), 7.55–7.62 (m, 2H), 7.80–7.86 (m, 3H). **^{13}C{1H} NMR** (126 MHz, $CDCl_3$): δ/ppm = 14.5, 21.7, 61.5, 118.9, 123.4 (2C), 129.0, 129.6, 129.8, 129.9 (2C), 131.9, 142.2, 150.9, 152.2, 167.8. **HRMS** (APCI) calculated for $C_{16}H_{17}N_2O_3^+$ [(M+H)$^+$]: 285.1234; found: 285.1230. **IR** (ATR): $\tilde{\nu}$/cm^{-1} = 2978, 2923, 1718, 1597, 1501, 1473, 1439, 1364, 1287, 1247, 1149, 1120, 1076, 1036, 1013, 954, 823.

(*E*)-1-(4-((4-Methoxyphenyl)diazenyl)phenyl)ethan-1-one (6bs)

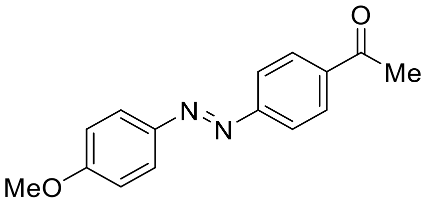

6bs
$C_{15}H_{14}N_2O_2$
M = 254.29 g/mol

Prepared according to **GP1** from the (*E*)-1-(4-methoxyphenyl)-2-(trimethylsilyl)diazene (**1b**, 50.0 mg, 0.240 mmol, 1.2 equiv) and 1-(4-bromophenyl)ethan-1-one (**2s**, 39.8 mg, 0.200 mmol, 1.0 equiv). Purification by flash column chromatography on silica gel using *n*-pentane:*tert*-butyl methyl ether (95:5) as eluent afforded the title compound **6bs** (35.3 mg, 0.139 mmol, 69%) as a light orange solid.

R_f = 0.23 (*n*-pentane:*tert*-butyl methyl ether 9:1). **M.p.**: 158–160 °C (*n*-pentane). **^{1}H NMR** (400 MHz, $CDCl_3$): δ/ppm = 2.66 (s, 3H), 3.91 (s, 3H), 7.03 (d, *J* = 9.0 Hz, 2H), 7.93 (d, *J* = 8.4 Hz, 2H), 7.96 (d, *J* = 9.1 Hz, 2H), 8.09 (d, *J* = 8.4 Hz, 2H). **^{13}C{1H} NMR** (101 MHz, $CDCl_3$): δ/ppm = 26.9, 55.8, 114.5 (2C), 122.7 (2C), 125.4 (2C), 129.5 (2C), 138.0, 147.2, 155.5, 162.9, 197.6. **HRMS** (APCI) calculated for $C_{15}H_{15}N_2O_2^+$ [(M+H)$^+$]: 255.1129; found: 255.1126. **IR** (ATR): $\tilde{\nu}$/cm^{-1} = 3329, 2960, 2919, 2841, 1667, 1598, 1579, 1497, 1402, 1355, 1248, 1141, 1105, 1024, 958, 836.

(*E*)-(4-((4-Methoxyphenyl)diazenyl)phenyl)(phenyl)methanone (6bt)

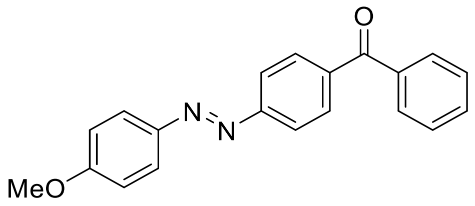

6bt
$C_{20}H_{16}N_2O_2$
M = 316.36 g/mol

Prepared according to **GP1** from the (*E*)-1-(4-methoxyphenyl)-2-(trimethylsilyl)diazene (**1b**, 50.0 mg, 0.240 mmol, 1.2 equiv) and (4-bromophenyl)(phenyl)methanone (**2t**, 52.2 mg, 0.200 mmol, 1.0 equiv). Purification by flash column chromatography on silica gel using *n*-pentane: dichloromethane (1:1) as eluent afforded the title compound **6bt** (42.9 mg, 0.136 mmol, 68%) as a light orange solid.

R_f = 0.19 (*n*-pentane:dichloromethane 1:1). **M.p.**: 154–156 °C (*n*-pentane). **^{1}H NMR** (400 MHz, $CDCl_3$): δ/ppm = 3.91 (s, 3H), 7.04 (d, *J* = 8.9 Hz, 2H), 7.51 (m_c, 2H), 7.62 (tt, *J* = 7.4 Hz, *J* = 2.0 Hz, 1H), 7.82–7.86 (m, 2H), 7.95 (app s, 4H), 7.97 (d, *J* = 8.9 Hz, 2H). **^{13}C{1H} NMR** (101 MHz, $CDCl_3$): δ/ppm = 55.8, 114.5 (2C), 122.5 (2C), 125.4 (2C), 128.5 (2C), 130.2 (2C), 131.2 (2C), 132.7, 137.7, 138.7, 147.2, 155.1, 162.9, 196.2. **HRMS** (APCI) calculated for $C_{20}H_{17}N_2O_2^+$ [(M+H)$^+$]: 317.1285; found: 317.1284. **IR** (ATR): $\tilde{\nu}$/cm^{-1} = 3280, 2959, 2910, 2837, 1641, 1595, 1577, 1491, 1448, 1404, 1300, 1276, 1239, 1179, 1136, 1104, 1027, 938, 858, 837.

(*E*)-1-(3,5-Bis(trifluoromethyl)phenyl)-2-(4-fluoro-2,6-dimethylphenyl)diazene (6ju)

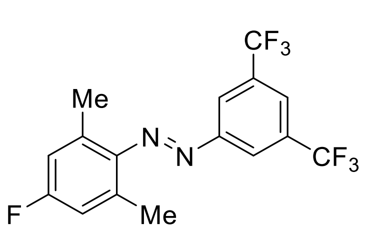

6ju
$C_{16}H_{11}F_7N_2$
M = 364.27 g/mol

Prepared according to **GP1** from the (*E*)-1-(4-fluoro-2,6-dimethylphenyl)-2-(trimethylsilyl)-diazene (**1j**, 53.8 mg, 0.240 mmol, 1.2 equiv) and 1-bromo-3,5-bis(trifluoromethyl)benzene

(**2u**, 58.6 mg, 0.200 mmol, 1.0 equiv) at 45 °C. Purification by flash column chromatography on silica gel using *n*-pentane as eluent afforded the title compound **6ju** (47.2 mg, 0.130 mmol, 65%) as an orange solid.

RR_f = 0.53 (*n*-pentane). **M.p.**: 160–162 °C (*n*-pentane). **^{1}H NMR** (700 MHz, $CDCl_3$): δ/ppm = 2.51 (s, 6H), 6.88 (d, *J* = 9.1 Hz, 2H), 7.98 (s, 1H), 8.28 (s, 2H). **^{13}C{1H} NMR** (176 MHz, $CDCl_3$): δ/ppm = 20.4 (2C), 116.4 (d, *J* = 22.0 Hz, 2C), 122.6 (m_c, 2C), 123.2 (q, *J* = 273.1 Hz, 2C), 123.8 (m_c), 132.9 (q, *J* = 34.0 Hz, 2C), 136.5 (d, *J* = 9.3 Hz, 2C), 146.2 (d, *J* = 2.7 Hz), 153.4, 163.0 (d, *J* = 251.5 Hz). **^{19}F NMR** (659 MHz, $CDCl_3$): δ/ppm = –110.7 (t, *J* = 9.1 Hz, 1F), –62.9 (s, 6F). **HRMS** (APCI) calculated for $C_{16}H_{12}F_7N_2^+$ [(M+H)$^+$]: 365.0884; found: 365.0883. **IR** (ATR): $\tilde{\nu}$/cm^{-1} = 3107, 2972, 2928, 2852, 1586, 1492, 1454, 1361, 1279, 1197, 1162, 1122, 1019, 901, 860.

(*E*)-1-(4-(*p*-Tolyldiazenyl)phenyl)cyclopropane-1-carbonitrile (6av)

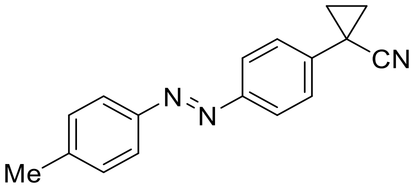

6av
$C_{17}H_{15}N_3$
M = 261.33 g/mol

Prepared according to **GP1** from the (*E*)-1-(*p*-tolyl)-2-(trimethylsilyl)diazene (**1a**, 46.2 mg, 0.240 mmol, 1.2 equiv) and 1-(4-bromophenyl)cyclopropane-1-carbonitrile (**2v**, 44.4 mg, 0.200 mmol, 1.0 equiv). Purification by flash column chromatography on silica gel using *n*-pentane:*tert*-butyl methyl ether (95:5) as eluent afforded the title compound **6av** (41.3 mg, 0.158 mmol, 79%) as an orange solid.

R_f = 0.29 (*n*-pentane:*tert*-butyl methyl ether 9:1). **M.p.**: 140–142 °C (*n*-pentane). **^{1}H NMR** (500 MHz, $CDCl_3$): δ/ppm = 1.49 (m_c, 2H), 1.81 (m_c, 2H), 2.44 (s, 3H), 7.32 (d, *J* = 8.1 Hz, 2H), 7.42 (d, *J* = 8.5 Hz, 2H), 7.83 (d, *J* = 8.1 Hz, 2H), 7.89 (d, *J* = 8.5 Hz, 2H). **^{13}C{1H} NMR** (126 MHz, $CDCl_3$): δ/ppm = 14.0, 19.0 (2C), 21.7, 122.3, 123.1 (2C), 123.4 (2C), 126.3 (2C), 129.9 (2C), 138.6, 142.0, 150.8, 152.1. **HRMS** (APCI) calculated for $C_{17}H_{16}N_3^+$ [(M+H)$^+$]: 262.1339; found: 262.1336. **IR** (ATR): $\tilde{\nu}$/cm^{-1} = 3101, 3020, 2919, 2853, 2236, 1600, 1496, 1434, 1412, 1302, 1154, 1098, 1077, 945, 851, 822.

Ethyl (*S*,*E*)-2-acetamido-3-(4-(*p*-tolyldiazenyl)phenyl)propanoate (6aw)

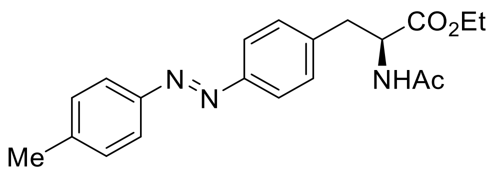

(*S*)-**6aw**
$C_{20}H_{23}N_3O_3$
M = 353.42 g/mol

Prepared according to **GP1** from the (*E*)-1-(*p*-tolyl)-2-(trimethylsilyl)diazene (**1a**, 23.1 mg, 0.120 mmol, 1.2 equiv) and ethyl (*S*)-2-acetamido-3-(4-(((trifluoromethyl)sulfonyl)oxy)phenyl)-propanoate (**3w**, 38.3 mg, 0.100 mmol, 1.0 equiv). Purification by flash column chromatography on silica gel using *n*-pentane:*tert*-butyl methyl ether (1:4) as eluent afforded the title compound (*S*)-**6aw** (22.6 mg, 64.0 µmol, 64%) as a light orange solid.

$\mathbf{R_f}$ = 0.23 (*n*-pentane:*tert*-butyl methyl ether 1:4). **M.p.**: 138–140 °C (*n*-pentane). ^{1}**H NMR** (400 MHz, $CDCl_3$): δ/ppm = 1.19 (t, *J* = 7.2 Hz, 3H), 1.94 (s, 3H), 2.37 (s, 3H), 3.14 (m_c, 2H), 4.13 (q, *J* = 7.2 Hz, 2H), 4.84 (m_c, 1H), 5.88 (d, *J* = 7.8 Hz, 1H), 7.18 (d, *J* = 8.3 Hz, 2H), 7.25 (d, *J* = 8.2 Hz, 2H), 7.74 (d, *J* = 8.2 Hz, 2H), 7.76 (d, *J* = 8.3 Hz, 2H). ^{13}**C{1H} NMR** (101 MHz, $CDCl_3$): δ/ppm = 14.3, 21.7, 23.4, 38.0, 53.2, 61.8, 122.99 (2C), 123.01 (2C), 129.9 (2C), 130.2 (2C), 139.0, 141.8, 150.9, 152.0, 169.7, 171.6. **HRMS** (APCI) calculated for $C_{20}H_{24}N_3O_3^+$ [$(M+H)^+$]: 354.1813; found: 354.1811. **IR** (ATR): $\tilde{\nu}$/cm^{-1} = 3311, 3026, 2971, 2921, 2852, 1728, 1639, 1527, 1442, 1374, 1345, 1259, 1196, 1129, 1098, 1063, 1011, 969, 857, 826.

(*E*)-2-((4-Chlorophenyl)diazenyl)-5-methylpyridine (9ea)

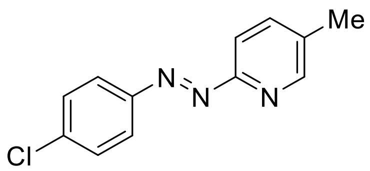

9ea
$C_{12}H_{10}ClN_3$
M = 231.68 g/mol

Prepared according to **GP1** from the (*E*)-1-(4-chlorophenyl)-2-(trimethylsilyl)diazene (**1e**, 51.1 mg, 0.240 mmol, 1.2 equiv) and 2-bromo-5-methylpyridine (**8a**, 34.4 mg, 0.200 mmol, 1.0 equiv). Purification by flash column chromatography on silica gel using *n*-pentane:*tert*-butyl

methyl ether (4:1) as eluent afforded the title compound **9ea** (26.4 mg, 0.114 mmol, 57%) as a light orange solid.

R$_f$ = 0.19 (*n*-pentane:*tert*-butyl methyl ether 4:1). **M.p.**: 170–172 °C (*n*-pentane). **^{1}H NMR** (400 MHz, $CDCl_3$): δ/ppm = 2.44 (s, 3H), 7.50 (d, *J* = 8.8 Hz, 2H), 7.68–7.78 (m, 2H), 7.99 (d, *J* = 8.8 Hz, 2H), 8.56 (m$_c$, 1H). **^{13}C{1H} NMR** (101 MHz, $CDCl_3$): δ/ppm = 18.6, 115.6, 124.9 (2C), 129.6 (2C), 136.0, 138.0, 138.9, 150.1, 151.0, 161.1. **HRMS** (APCI) calculated for $C_{12}H_{11}ClN_3^+$ [$(M+H)^+$]: 232.0637; found: 232.0633. **IR** (ATR): $\tilde{\nu}$/cm^{-1} = 3049, 2919, 2853, 1572, 1474, 1401, 1371, 1219, 1144, 1023, 1074, 832.

(*E*)-1-(4-Chlorophenyl)-2-(thiophen-3-yl)diazene (9eb)

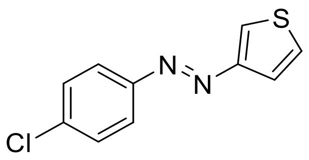

9eb
$C_{10}H_7ClN_2S$
M = 222.69 g/mol

Prepared according to **GP1** from the (*E*)-1-(4-chlorophenyl)-2-(trimethylsilyl)diazene (**1e**, 51.1 mg, 0.240 mmol, 1.2 equiv) and 3-bromothiophene (**8b**, 32.6 mg, 0.200 mmol, 1.0 equiv). Purification by flash column chromatography on silica gel using *n*-pentane:*tert*-butyl methyl ether (99:1) as eluent afforded the title compound **9eb** (31.2 mg, 0.140 mmol, 70%) as a yellow solid.

R$_f$ = 0.17 (*n*-pentane). **M.p.**: 121–123 °C (*n*-pentane). **^{1}H NMR** (400 MHz, $CDCl_3$): δ/ppm = 7.36 (dd, *J* = 5.3 Hz, *J* = 3.2 Hz, 1H), 7.47 (d, *J* = 8.7 Hz, 2H), 7.58 (dd, *J* = 5.3 Hz, *J* = 1.3 Hz, 1H), 7.81 (d, *J* = 8.7 Hz, 2H), 8.06 (dd, *J* = 3.2 Hz, *J* = 1.3 Hz, 1H). **^{13}C{1H} NMR** (101 MHz, $CDCl_3$): δ/ppm = 118.8, 124.0 (2C), 126.6, 127.5, 129.5 (2C), 136.7, 151.3, 157.0. **HRMS** (APCI) calculated for $C_{10}H_8ClN_2S^+$ [$(M+H)^+$]: 223.0092; found: 223.0090. **IR** (ATR): $\tilde{\nu}$/cm^{-1} = 3102, 2920, 2850, 1572, 1510, 1478, 1447, 1420, 1397, 1297, 1218, 1088, 1001, 947, 875.

(*E*)-1-(Naphthalen-2-yl)-2-(thiophen-2-yl)diazene (9kc)

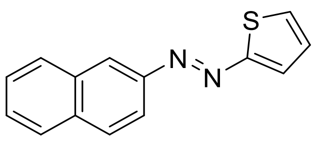

9kc
$C_{14}H_{10}N_2S$
M = 238.31 g/mol

Prepared according to **GP1** from the (*E*)-1-(naphthalen-2-yl)-2-(trimethylsilyl)diazene (**1k**, 54.8 mg, 0.240 mmol, 1.2 equiv) and 2-bromothiophene (**8c**, 32.6 mg, 0.200 mmol, 1.0 equiv). Purification by flash column chromatography on silica gel using *n*-pentane:dichloromethane (95:5) as eluent afforded the title compound **9kc** (33.2 mg, 0.139 mmol, 70%) as an orange solid.

R_f = 0.22 (*n*-pentane:dichloromethane 9:1). **M.p.**: 110–112 °C (*n*-pentane). **^{1}H NMR** (500 MHz, $CDCl_3$): δ/ppm = 7.19 (dd, *J* = 5.4 Hz, *J* = 3.8 Hz, 1H), 7.43 (dd, *J* = 5.4 Hz, *J* = 1.3 Hz, 1H), 7.53–7.57 (m, 2H), 7.84 (dd, *J* = 3.8 Hz, *J* = 1.3 Hz, 1H), 7.86–7.91 (m, 2H), 7.97–8.00 (m, 1H), 8.02 (dd, *J* = 8.9 Hz, *J* = 1.9 Hz, 1H), 8.38 (app d, *J* = 1.9 Hz, 1H). **^{13}C{1H} NMR** (126 MHz, $CDCl_3$): δ/ppm = 117.4, 126.9, 127.66, 127.68, 128.1 (2C), 128.6, 129.3, 129.4, 131.7, 133.7, 134.8, 149.9, 160.8. **HRMS** (APCI) calculated for $C_{14}H_{11}N_2S^+$ [(M+H)$^+$]: 239.0638; found: 239.0640. **IR** (ATR): $\tilde{\nu}$/cm^{-1} = 3057, 2920, 2851, 1379, 1230, 1203, 1033, 954, 907.

***tert*-Butyl (*E*)-5-((4-methoxyphenyl)diazenyl)-1*H*-indole-1-carboxylate (9bd)**

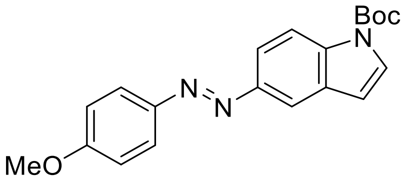

9bd
$C_{20}H_{21}N_3O_3$
M = 351.41 g/mol

Prepared according to **GP1** from the (*E*)-1-(4-methoxyphenyl)-2-(trimethylsilyl)diazene (**1b**, 50.0 mg, 0.240 mmol, 1.2 equiv) and *tert*-butyl 5-bromo-1*H*-indole-1-carboxylate (**8d**, 59.2 mg, 0.200 mmol, 1.0 equiv). Purification by flash column chromatography on silica gel using *n*-pentane:*tert*-butyl methyl ether (98:2) as eluent afforded the title compound **9bd** (62.2 mg, 0.177 mmol, 89%) as an orange solid.

R$_f$ = 0.49 (*n*-pentane:*tert*-butyl methyl ether 9:1). **M.p.**: 147–149 °C (*n*-pentane). **^{1}H NMR** (500 MHz, $CDCl_3$): δ/ppm = 1.70 (s, 9H), 3.89 (s, 3H), 6.68 (d, *J* = 3.7 Hz, 1H), 7.03 (d, *J* = 8.9 Hz, 2H), 7.65 (d, *J* = 3.7 Hz, 1H), 7.92–7.96 (m, 3H), 8.11 (d, *J* = 1.9 Hz, 1H), 8.25 (d, *J* = 8.8 Hz, 1H). **^{13}C{1H} NMR** (126 MHz, $CDCl_3$): δ/ppm = 28.3 (3C), 55.7, 84.2, 108.3, 114.3 (2C), 115.6, 116.4, 118.9, 124.6 (2C), 127.3, 131.2, 136.6, 147.3, 149.0, 149.7, 161.9. **HRMS** (APCI) calculated for $C_{20}H_{22}N_3O_3^+$ [(M+H)$^+$]: 352.1656; found: 352.1661. **IR** (ATR): $\tilde{\nu}$/cm^{-1} = 2974, 2927, 1729, 1598, 1578, 1499, 1456, 1369, 1330, 1281, 1249, 1148, 1104, 1025, 897.

(*E*)-1-(Benzofuran-5-yl)-2-(4-methoxyphenyl)diazene (9be)

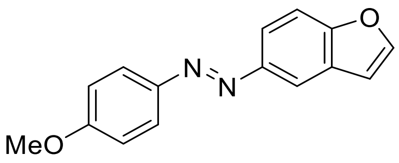

9be
$C_{15}H_{12}N_2O_2$
M = 252.27 g/mol

Prepared according to **GP1** from the (*E*)-1-(4-methoxyphenyl)-2-(trimethylsilyl)diazene (**1b**, 50.0 mg, 0.240 mmol, 1.2 equiv) and 5-bromobenzofuran (**8e**, 39.4 mg, 0.200 mmol, 1.0 equiv). Purification by flash column chromatography on silica gel using *n*-pentane:*tert*-butyl methyl ether (99:1) as eluent afforded the title compound **9be** (42.8 mg, 0.170 mmol, 85%) as a light orange solid.

R$_f$ = 0.47 (*n*-pentane:*tert*-butyl methyl ether 9:1). **M.p.**: 122–124 °C (*n*-pentane). **^{1}H NMR** (400 MHz, $CDCl_3$): δ/ppm = 3.90 (s, 3H), 6.88 (dd, *J* = 2.2 Hz, *J* = 1.0 Hz, 1H), 7.03 (d, *J* = 9.0 Hz, 2H), 7.59 (d, *J* = 8.8 Hz, 1H), 7.68 (d, *J* = 2.2 Hz, 1H), 7.91–7.96 (m, 3H), 8.15 (d, *J* = 1.9 Hz, 1H). **^{13}C{1H} NMR** (101 MHz, $CDCl_3$): δ/ppm = 55.7, 107.7, 111.9, 114.4 (2C), 116.6, 119.2, 124.7 (2C), 128.1, 146.4, 147.2, 149.3, 156.3, 162.0. **HRMS** (APCI) calculated for $C_{15}H_{13}N_2O_2^+$ [(M+H)$^+$]: 253.0972; found: 253.0970. **IR** (ATR): $\tilde{\nu}$/cm^{-1} = 2962, 2838, 1598, 1577, 1495, 1444, 1318, 1242, 1145, 1114, 1024, 905.

(*E*)-1-(Benzo[*b*]thiophen-5-yl)-2-(4-methoxyphenyl)diazene (9bf)

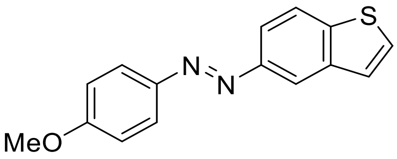

9bf
$C_{15}H_{12}N_2OS$
M = 268.33 g/mol

Prepared according to **GP1** from the (*E*)-1-(4-methoxyphenyl)-2-(trimethylsilyl)diazene (**1b**, 50.0 mg, 0.240 mmol, 1.2 equiv) and 5-bromobenzo[*b*]thiophene (**8f**, 42.6 mg, 0.200 mmol, 1.0 equiv). Purification by flash column chromatography on silica gel using *n*-pentane:*tert*-butyl methyl ether (99:1) as eluent afforded the title compound **9bf** (41.6 mg, 0.155 mmol, 78%) as a light orange solid.

R_f = 0.52 (*n*-pentane:*tert*-butyl methyl ether 9:1). **M.p.**: 122–124 °C (*n*-pentane). **^{1}H NMR** (400 MHz, $CDCl_3$): δ/ppm = 3.90 (s, 3H), 7.04 (d, *J* = 8.9 Hz, 2H), 7.47 (d, *J* = 5.5 Hz, 1H), 7.52 (d, *J* = 5.5 Hz, 1H), 7.93–7.98 (m, 4H), 8.35 (app t, *J* = 1.2 Hz, 1H). **^{13}C{1H} NMR** (101 MHz, $CDCl_3$): δ/ppm = 55.7, 114.4 (2C), 117.6, 119.9, 123.0, 124.8 (2C), 124.9, 127.9, 140.3, 141.8, 147.2, 150.4, 162.1. **HRMS** (APCI) calculated for $C_{15}H_{13}N_2OS^+$ [(M+H)$^+$]: 269.0744; found: 269.0741. **IR** (ATR): $\tilde{\nu}$/cm^{-1} = 2957, 2838, 1598, 1578, 1497, 1316, 1245, 1145, 1103, 1026, 904.

(*E*)-3-((4-Methoxyphenyl)diazenyl)quinoline (9bg)

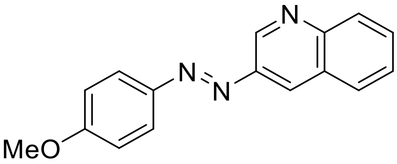

9bg
$C_{16}H_{13}N_3O$
M = 263.30 g/mol

Prepared according to **GP1** from the (*E*)-1-(4-methoxyphenyl)-2-(trimethylsilyl)diazene (**1b**, 50.0 mg, 0.240 mmol, 1.2 equiv) and 3-bromoquinoline (**8g**, 41.6 mg, 0.200 mmol, 1.0 equiv). Purification by flash column chromatography on silica gel using *n*-pentane:*tert*-butyl methyl ether (9:1) as eluent afforded the title compound **9bg** (39.9 mg, 0.152 mmol, 76%) as a red solid.

R_f = 0.15 (*n*-pentane:*tert*-butyl methyl ether 9:1). **M.p.**: 140–142 °C (*n*-pentane). **^{1}H NMR** (400 MHz, $CDCl_3$): δ/ppm = 3.90 (s, 3H), 7.04 (d, *J* = 9.0 Hz, 2H), 7.59 (m_c, 1H), 7.76 (m_c, 1H), 7.93–7.98 (m, 3H), 8.16 (d, *J* = 8.7 Hz, 1H), 8.53 (d, *J* = 2.3 Hz, 1H), 9.48 (d, *J* = 2.3 Hz, 1H). **^{13}C{1H} NMR** (101 MHz, $CDCl_3$): δ/ppm = 55.8, 114.5 (2C), 125.2 (2C), 127.4, 128.2, 128.9, 129.4, 129.6, 130.5, 145.1, 146.2, 147.3, 148.9, 162.8. **HRMS** (APCI) calculated for $C_{16}H_{14}N_3O^+$ [(M+H)$^+$]: 264.1132; found: 264.1128. **IR** (ATR): $\tilde{\nu}$/cm^{-1} = 3310, 2958, 2832, 1598, 1579, 1491, 1457, 1248, 1137, 1026, 918.

(*E*)-1-(Benzo[*b*]thiophen-3-yl)-2-(4-(trifluoromethyl)phenyl)diazene (9fh)

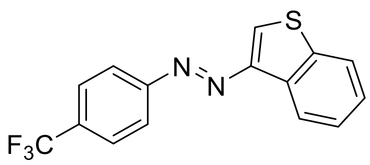

9fh
$C_{15}H_9F_3N_2S$
M = 306.31 g/mol

Prepared according to **GP1** from the (*E*)-1-(4-(trifluoromethyl)phenyl)-2-(trimethylsilyl)diazene (**1f**, 59.1 mg, 0.240 mmol, 1.2 equiv) and 3-bromobenzo[*b*]thiophene (**8h**, 42.6 mg, 0.200 mmol, 1.0 equiv). Purification by flash column chromatography on silica gel using *n*-pentane as eluent afforded the title compound **9fh** (39.7 mg, 0.130 mmol, 65%) as a red solid.

R_f = 0.16 (*n*-pentane). **M.p.**: 84–86 °C (*n*-pentane). **^{1}H NMR** (400 MHz, $CDCl_3$): δ/ppm = 7.46–7.57 (m, 2H), 7.79 (d, *J* = 8.4 Hz, 2H), 7.89 (m_c, 1H), 8.04 (d, *J* = 8.4 Hz, 2H), 8.32 (s, 1H), 8.76 (m_c, 1H). **^{13}C{1H} NMR** (101 MHz, $CDCl_3$): δ/ppm = 122.8 (2C), 122.9, 124.1 (q, *J* = 271.5 Hz), 124.9, 126.2, 126.3, 126.4 (q, *J* = 3.6 Hz, 2C), 131.0, 131.96, 131.99 (q, *J* = 31.9 Hz), 139.8, 149.3, 155.2. **^{19}F NMR** (471 MHz, $CDCl_3$): δ/ppm = –62.5 (s, 3F). **HRMS** (APCI) calculated for $C_{15}H_{10}F_3N_2S^+$ [(M+H)$^+$]: 307.0512; found: 307.0510. **IR** (ATR): $\tilde{\nu}$/cm^{-1} = 3081, 2923, 2850, 1607, 1499, 1435, 1399, 1318, 1157, 1114, 1062.

6 Scale-Up Experiment

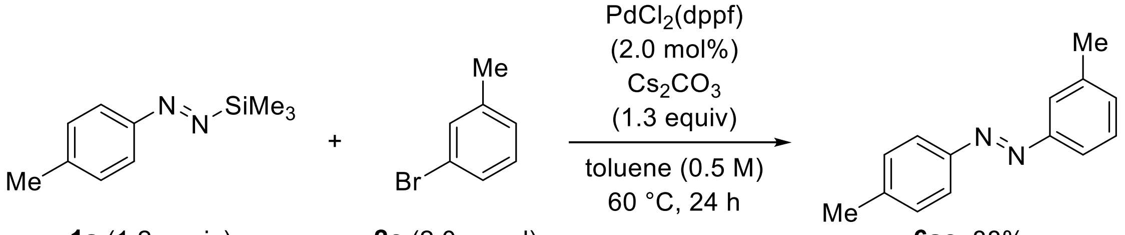

1a (1.2 equiv) **2c** (2.0 mmol) **6ac**: 88%

In an argon-filled glovebox, an oven-dried 10-mL screw-caped vial equipped with a magnetic stirring bar was charged with (dppf)$PdCl_2$ (29 mg, 40 µmol, 2.0 mol%) and Cs_2CO_3 (847 mg, 2.60 mmol, 1.3 equiv). Toluene (2 mL) was added, and the resulting suspension was stirred at room temperature for 5 min. Then, a solution of the (*E*)-1-(*p*-tolyl)-2-(trimethylsilyl)diazene (**1a**, 462 mg, 2.40 mmol, 1.2 equiv) and 1-bromo-3-methylbenzene (**2c**, 342 mg, 2.00 mmol, 1.0 equiv) in toluene (2 mL) was added in one portion, and the parent vial was rinsed with toluene (0.2 mL). The reaction mixture was stirred at 60 °C for 24 h. A color change from dark blue to light orange was observed.

The reaction mixture was quenched by the addition of a saturated aqueous solution of NH_4Cl (20 mL), and further diluted with *tert*-butyl methyl ether (40 mL) and water (20 mL). The organic layer was separated, and the aqueous phase was extracted with *tert*-butyl methyl ether (3 x 30 mL). The combined organic layers were dried over anhydrous $MgSO_4$, filtered, and concentrated under reduced pressure. The resulting crude residue was washed thrice with *n*-pentane. Purification by flash column chromatography on silica gel using *n*-pentane:*tert*-butyl methyl ether (99:1) as eluent afforded **6ac** (369 mg, 1.75 mmol, 88%) as an orange solid.

The NMR spectroscopic and mass spectrometric data were in accordance with those reported on a 0.200 mmol scale.

7 NMR Spectra

Figure S2. ^{1}H NMR spectrum (500 MHz, $CDCl_3$) of 1-(3-chlorophenyl)-2-(trimethylsilyl)hydrazine (**S2**).

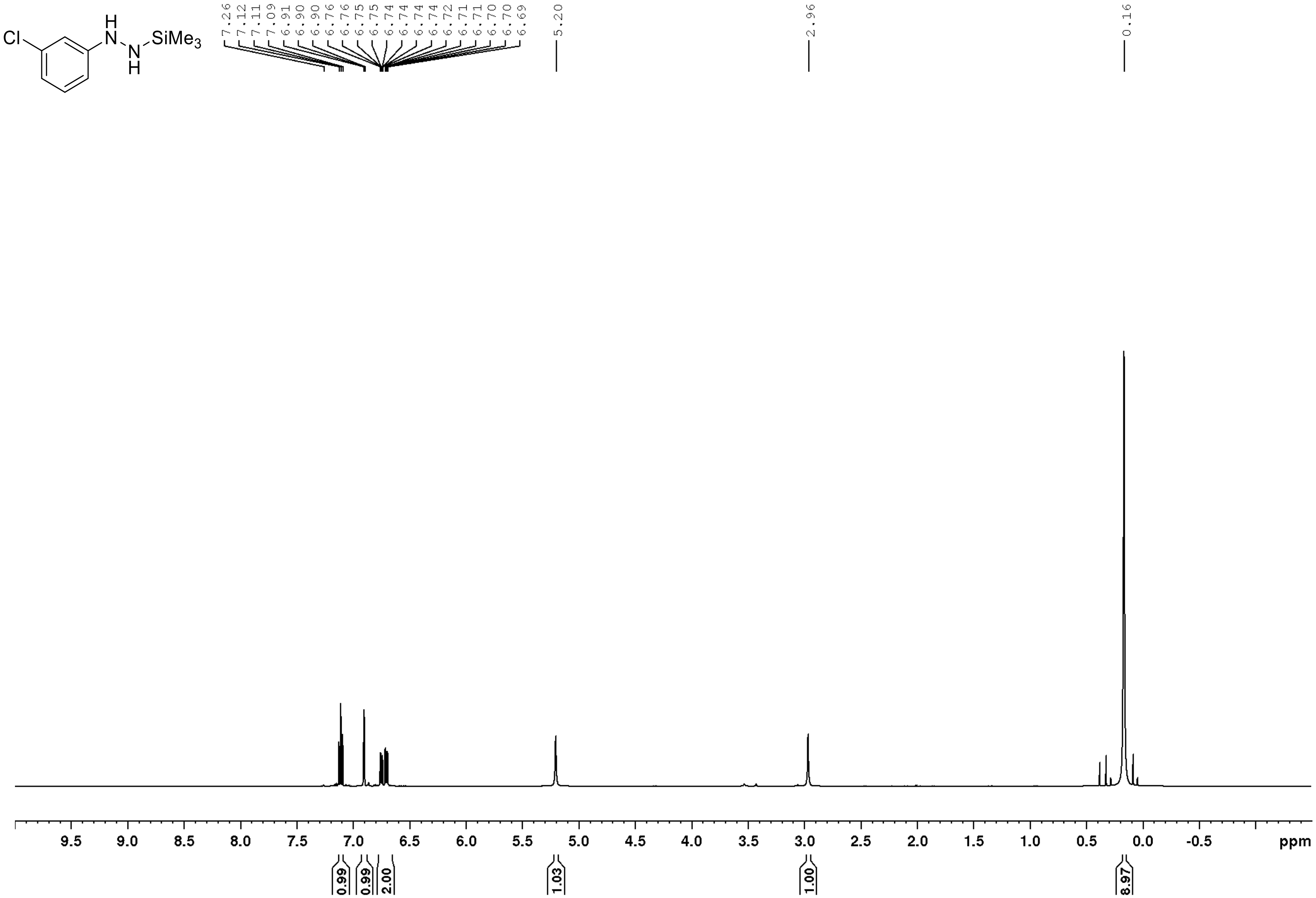

Figure S3. $^{13}C\{^1H\}$ NMR spectrum (126 MHz, $CDCl_3$) of 1-(3-chlorophenyl)-2-(trimethylsilyl)hydrazine (**S2**).

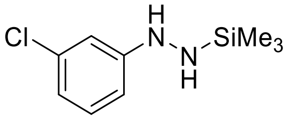

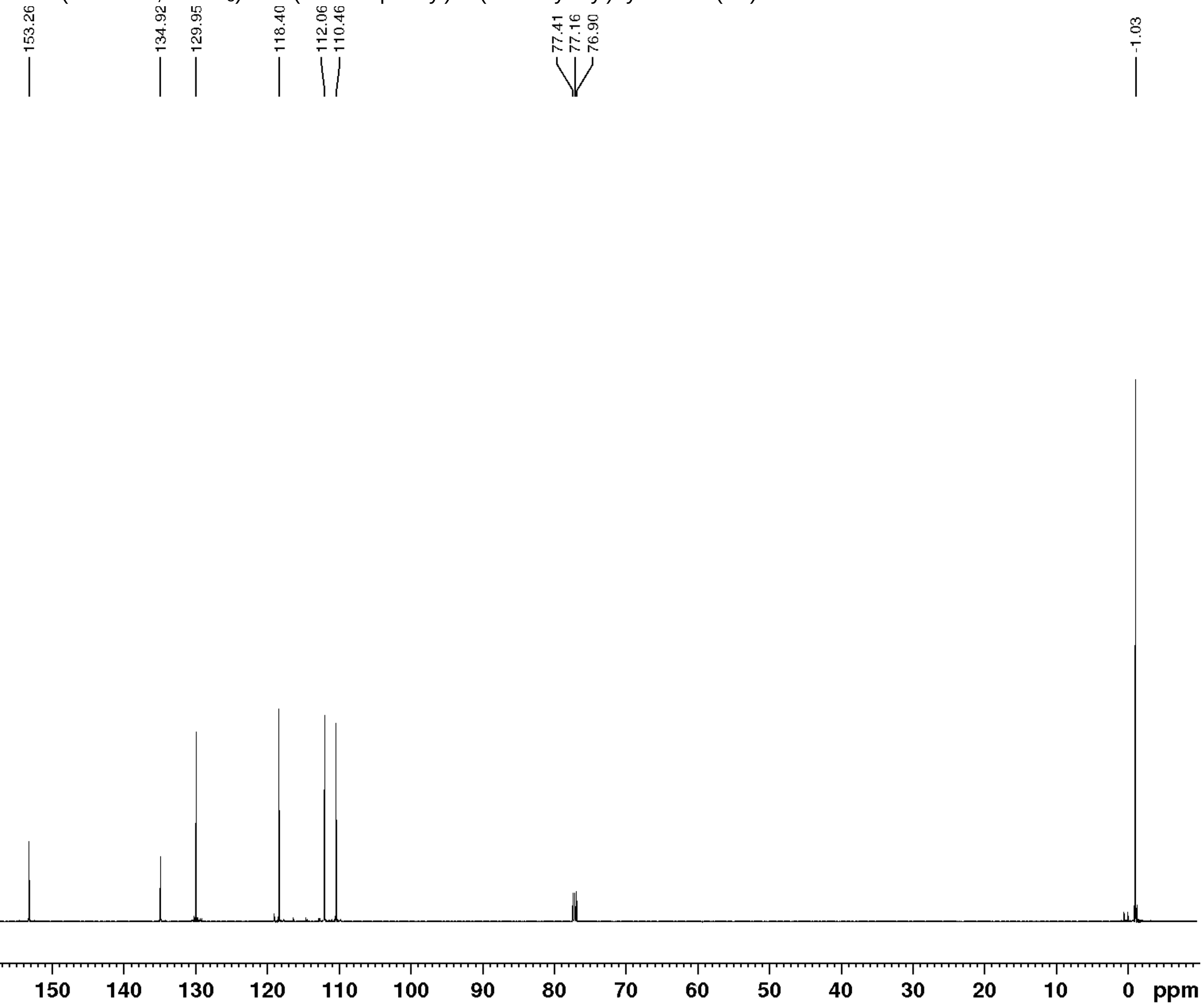

Figure S4. ^{1}H NMR spectrum (500 MHz, $CDCl_3$) of (*E*)-1-(3-chlorophenyl)-2-(trimethylsilyl)diazene (**1i**).

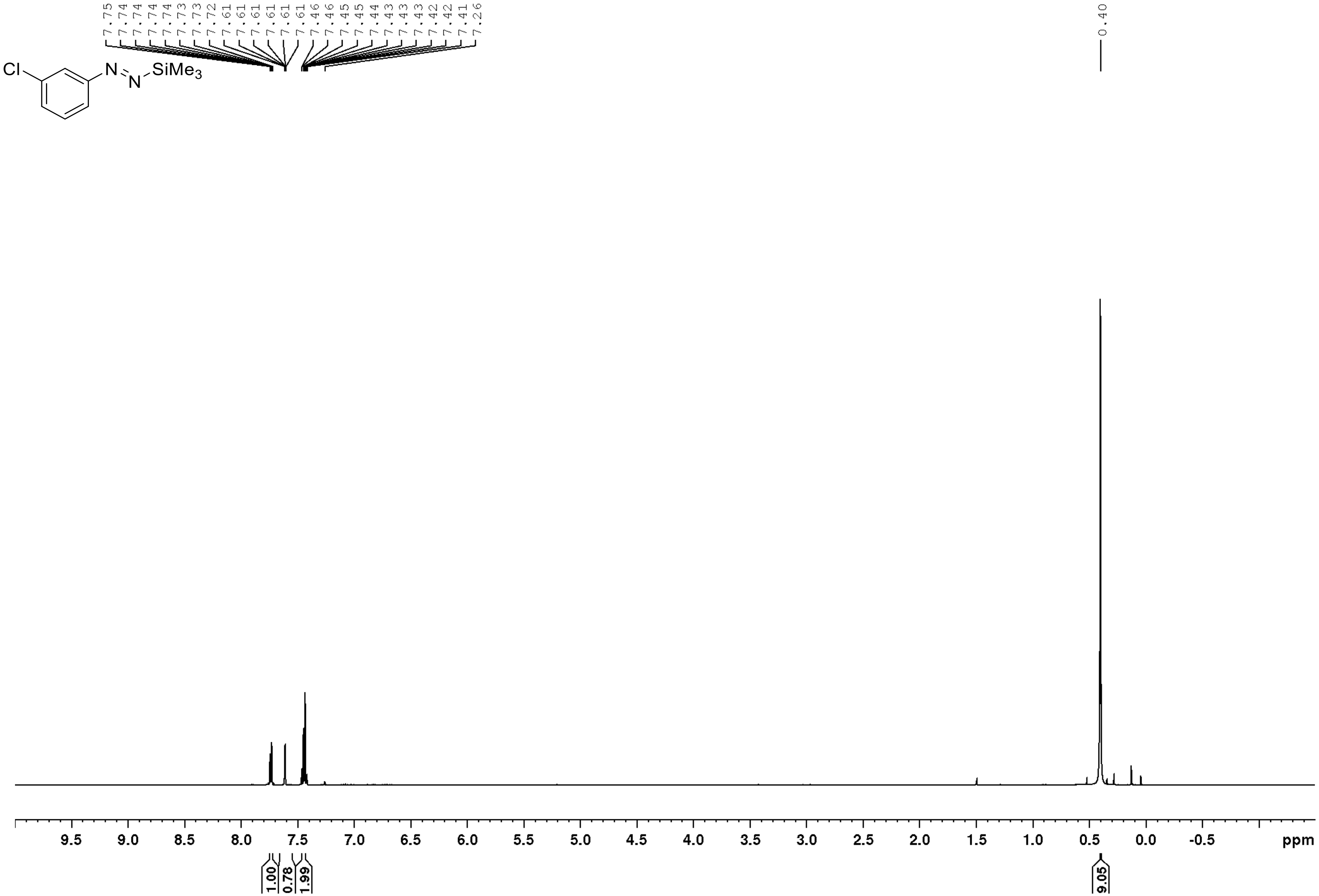

Figure S5. $^{13}C\{^{1}H\}$ NMR spectrum (126 MHz, $CDCl_3$) of (*E*)-1-(3-chlorophenyl)-2-(trimethylsilyl)diazene (**1i**).

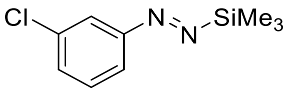

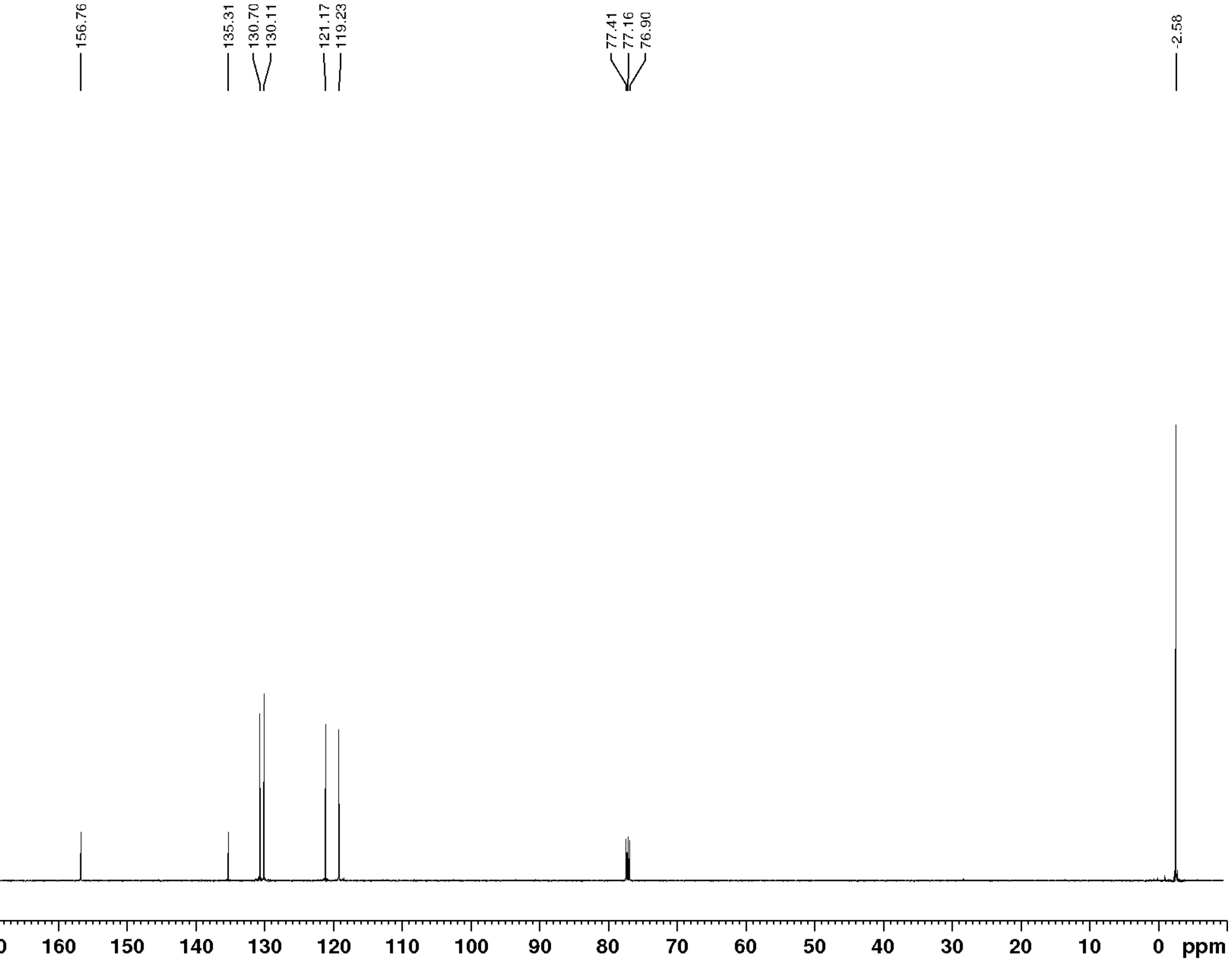

Figure S6. ^{1}H NMR spectrum (500 MHz, $CDCl_3$) of (*E*)-1-(3-fluorophenyl)-2-(*p*-tolyl)diazene (**6ab**).

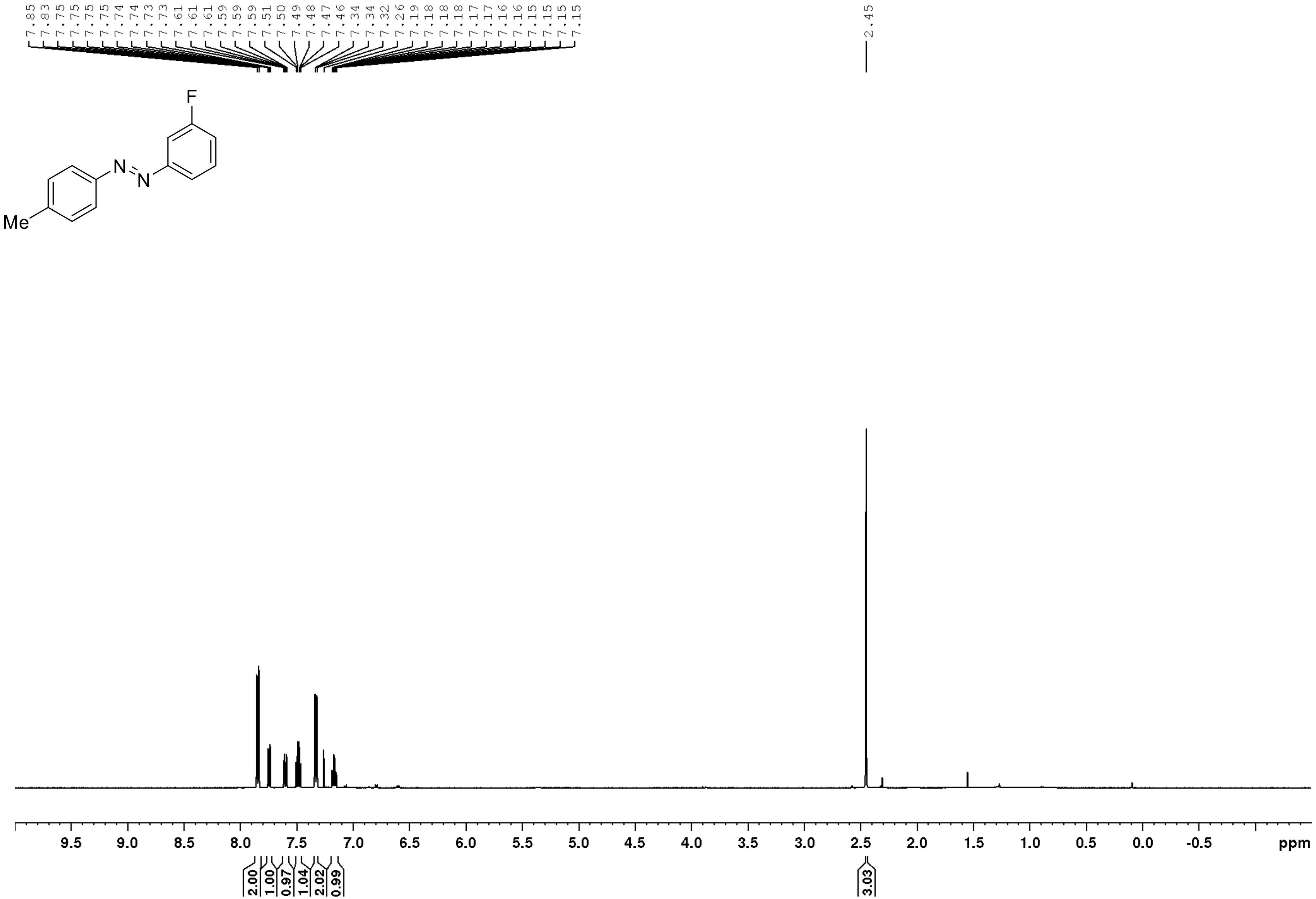

Figure S7. $^{13}C\{^{1}H\}$ NMR spectrum (126 MHz, $CDCl_3$) of (*E*)-1-(3-fluorophenyl)-2-(*p*-tolyl)diazene (**6ab**).

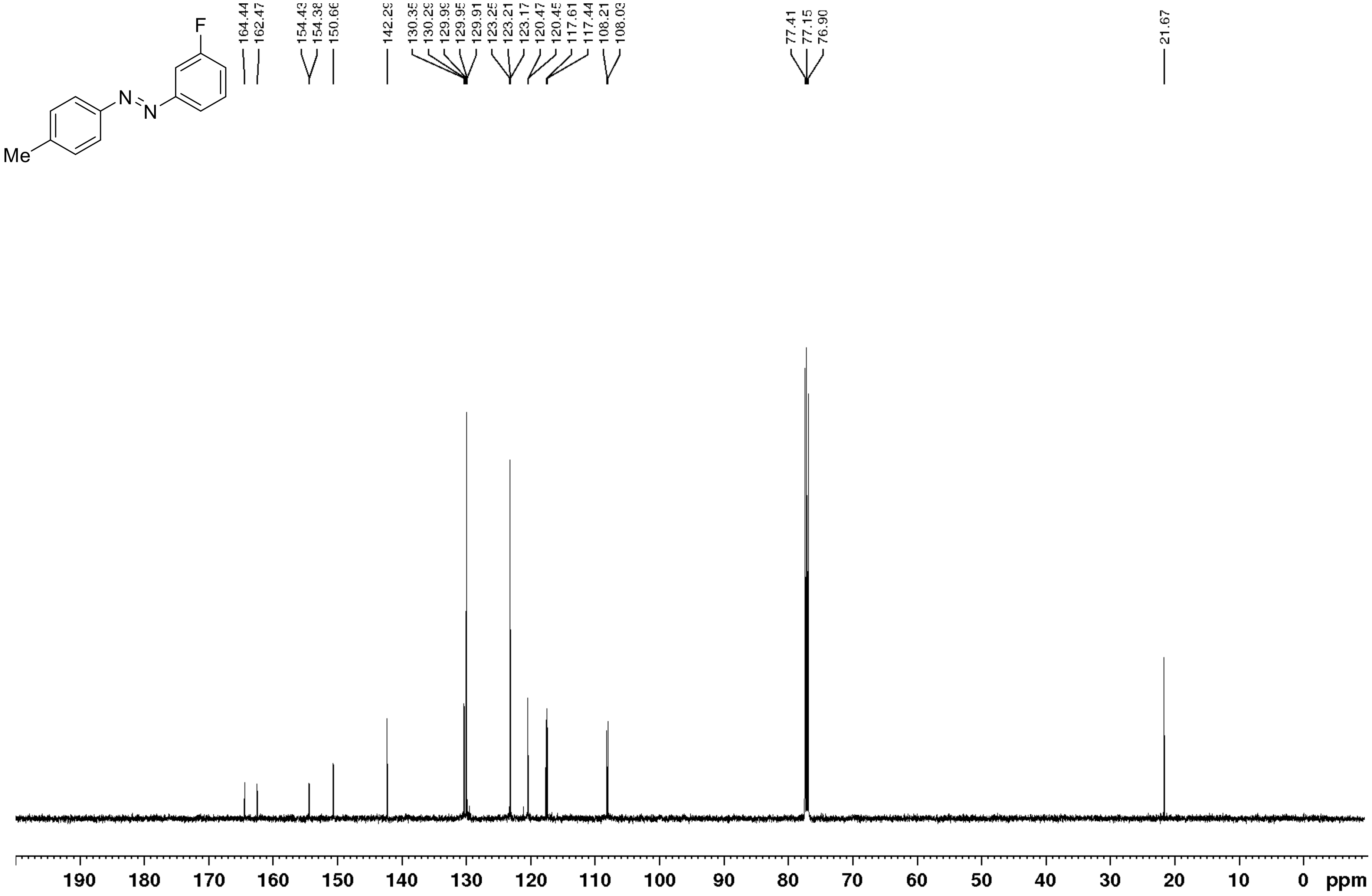

Figure S8. ^{19}F NMR spectrum (471 MHz, $CDCl_3$) of (*E*)-1-(3-fluorophenyl)-2-(*p*-tolyl)diazene (**6ab**).

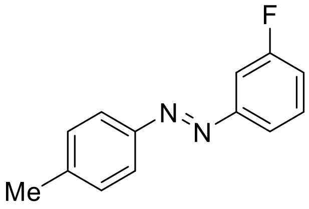

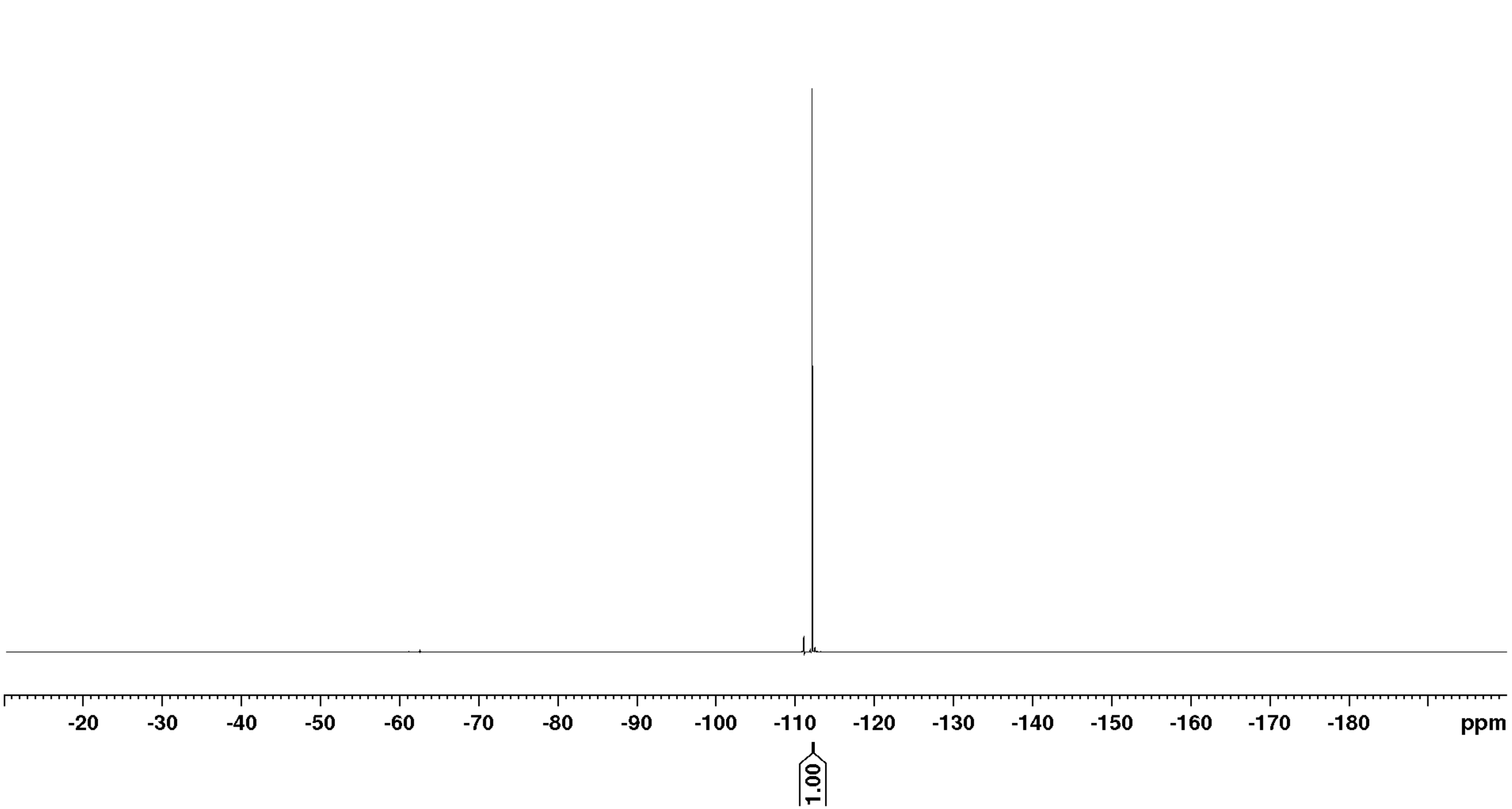

Figure S9. ^{1}H NMR spectrum (500 MHz, $CDCl_3$) of (*E*)-1-(3-fluorophenyl)-2-(4-methoxyphenyl)diazene (**6bb**).

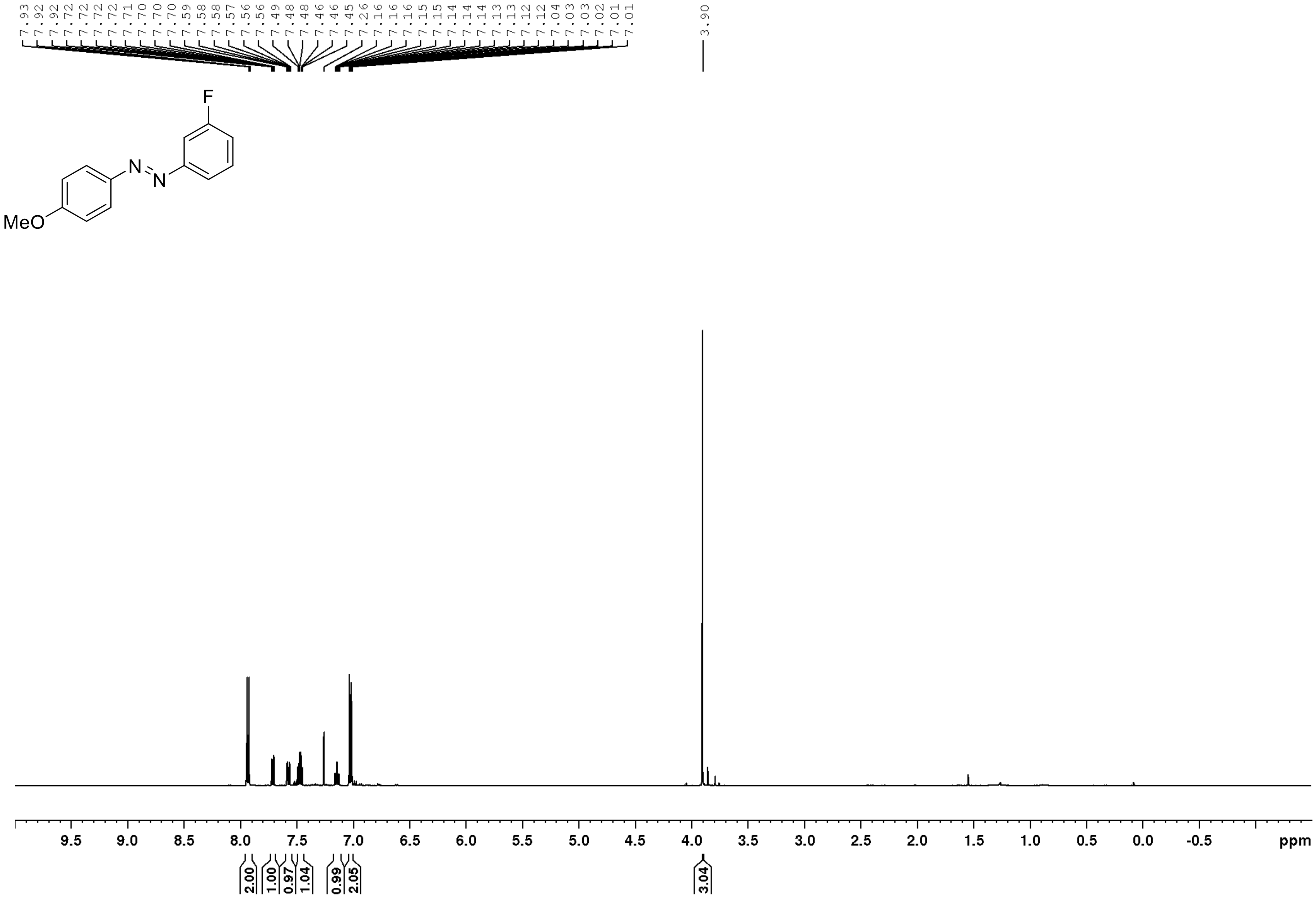

Figure S10. $^{13}C\{^{1}H\}$ NMR spectrum (126 MHz, $CDCl_3$) of (*E*)-1-(3-fluorophenyl)-2-(4-methoxyphenyl)diazene (**6bb**).

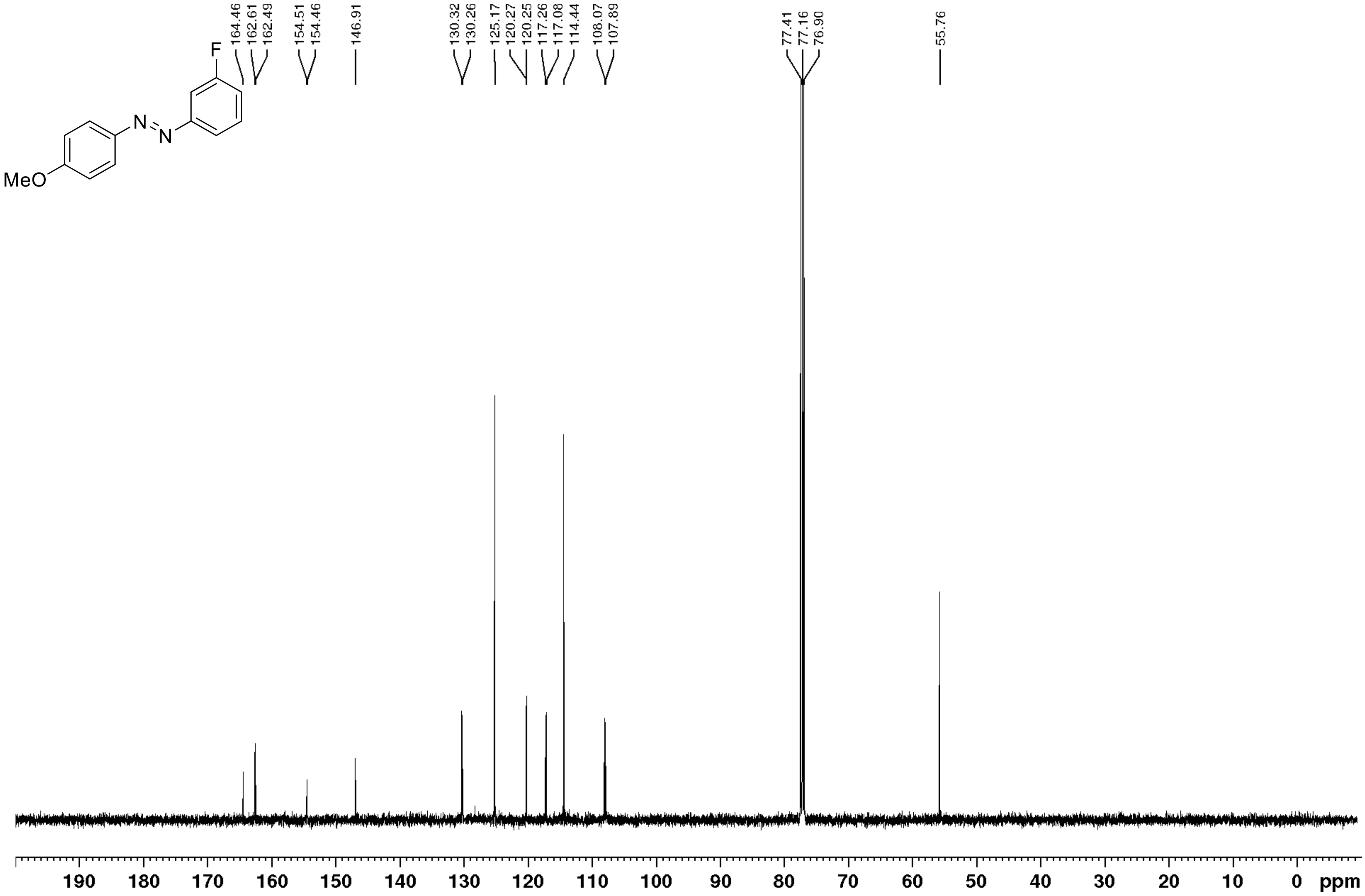

Figure S11. ^{19}F NMR spectrum (471 MHz, $CDCl_3$) of (*E*)-1-(3-fluorophenyl)-2-(4-methoxyphenyl)diazene (**6bb**).

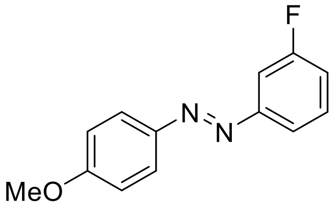

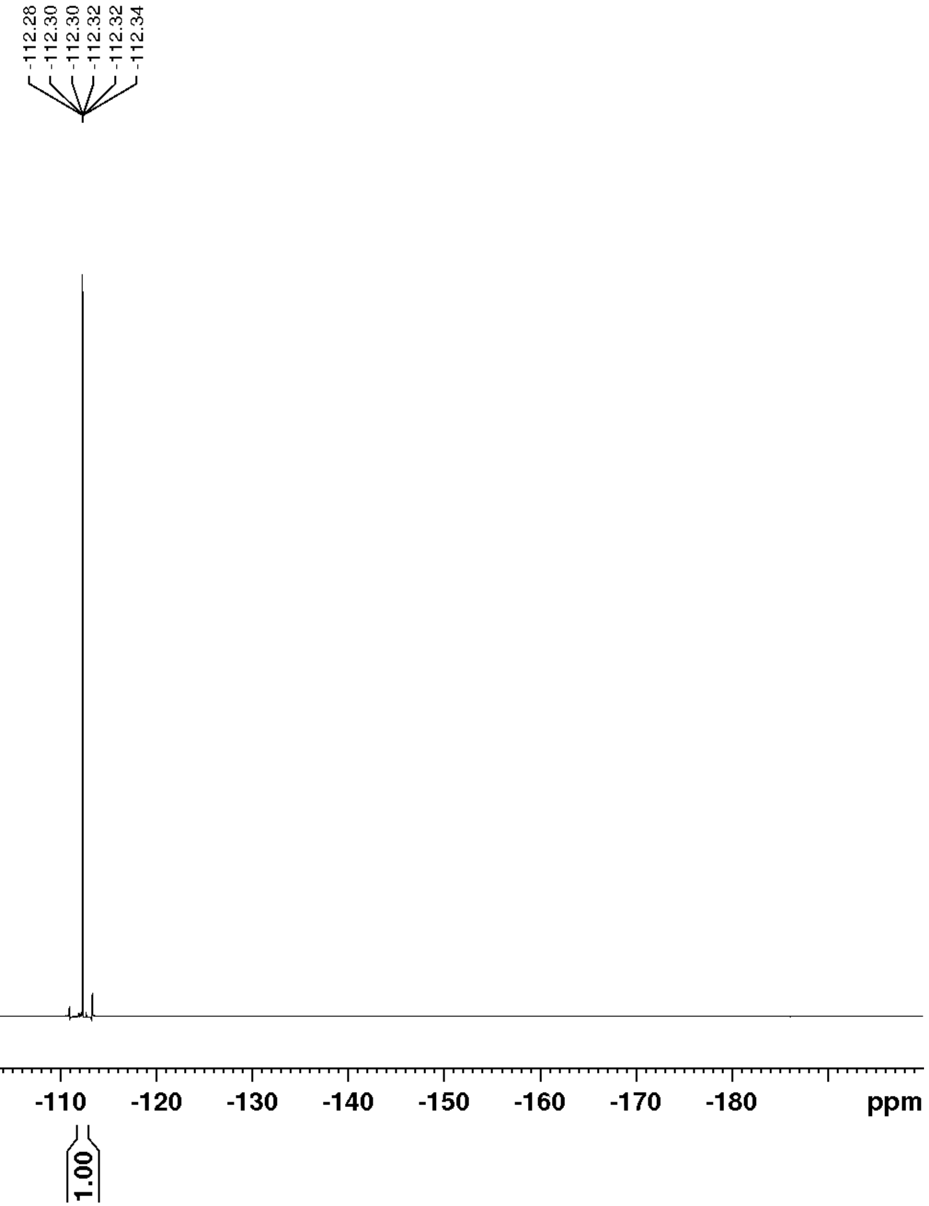

Figure S12. ^{1}H NMR spectrum (500 MHz, $CDCl_3$) of (*E*)-1-(3-fluorophenyl)-2-phenyldiazene (**6cb**).

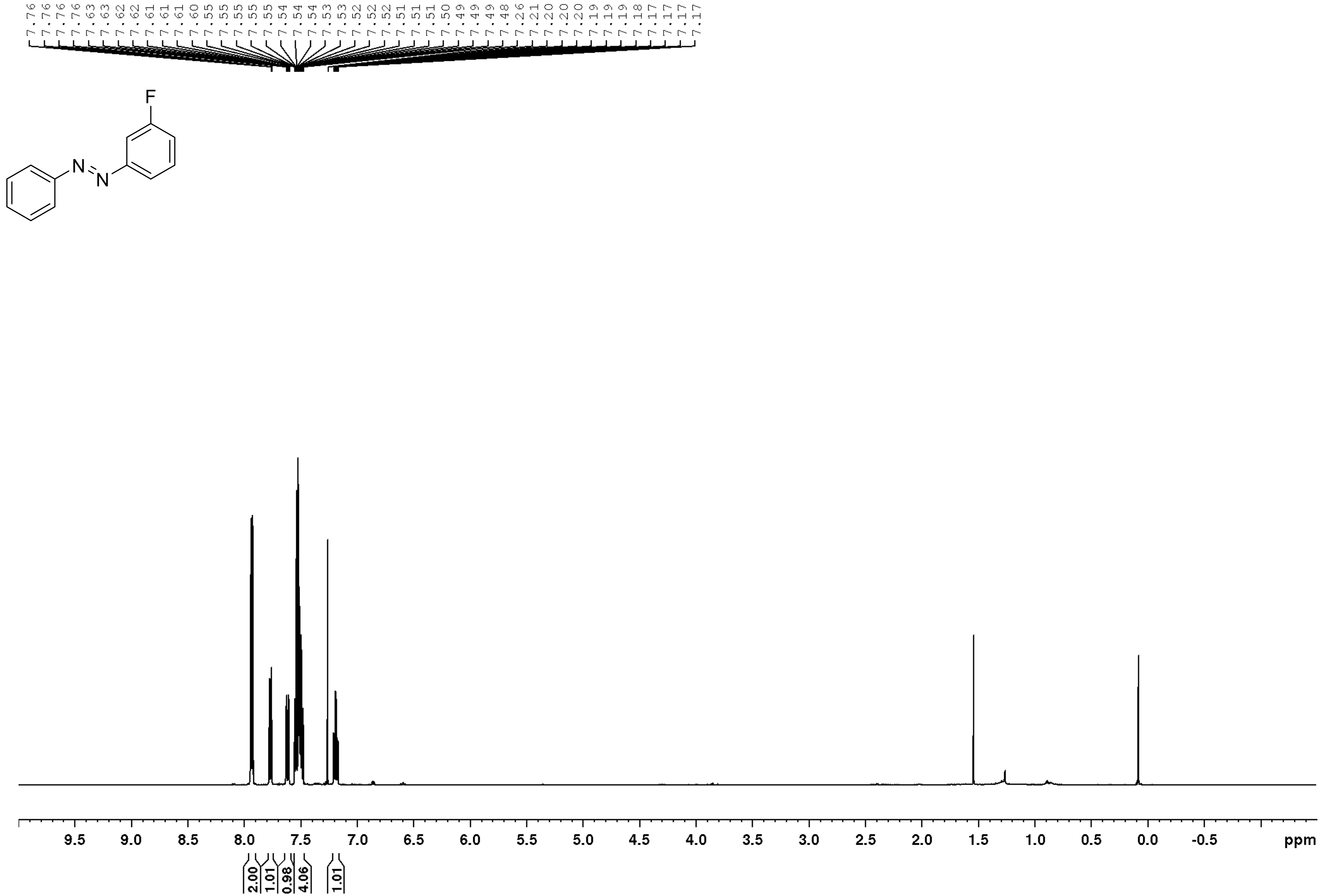

Figure S13. $^{13}C\{^{1}H\}$ NMR spectrum (126 MHz, $CDCl_3$) of (*E*)-1-(3-fluorophenyl)-2-phenyldiazene (**6cb**).

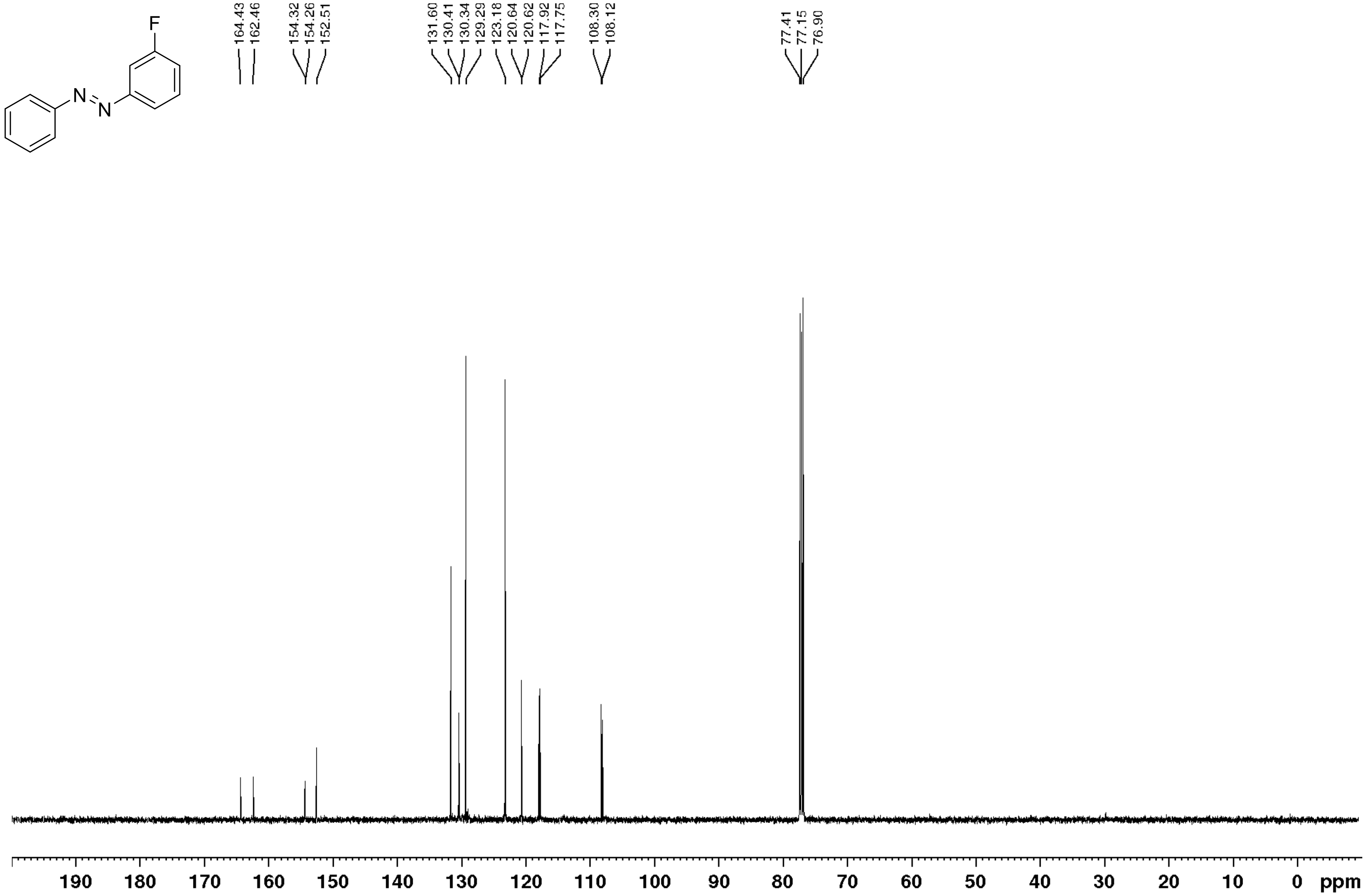

Figure S14. ^{19}F NMR spectrum (471 MHz, $CDCl_3$) of (*E*)-1-(3-fluorophenyl)-2-phenyldiazene (**6cb**).

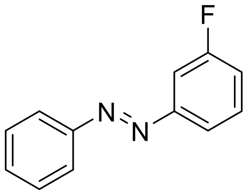

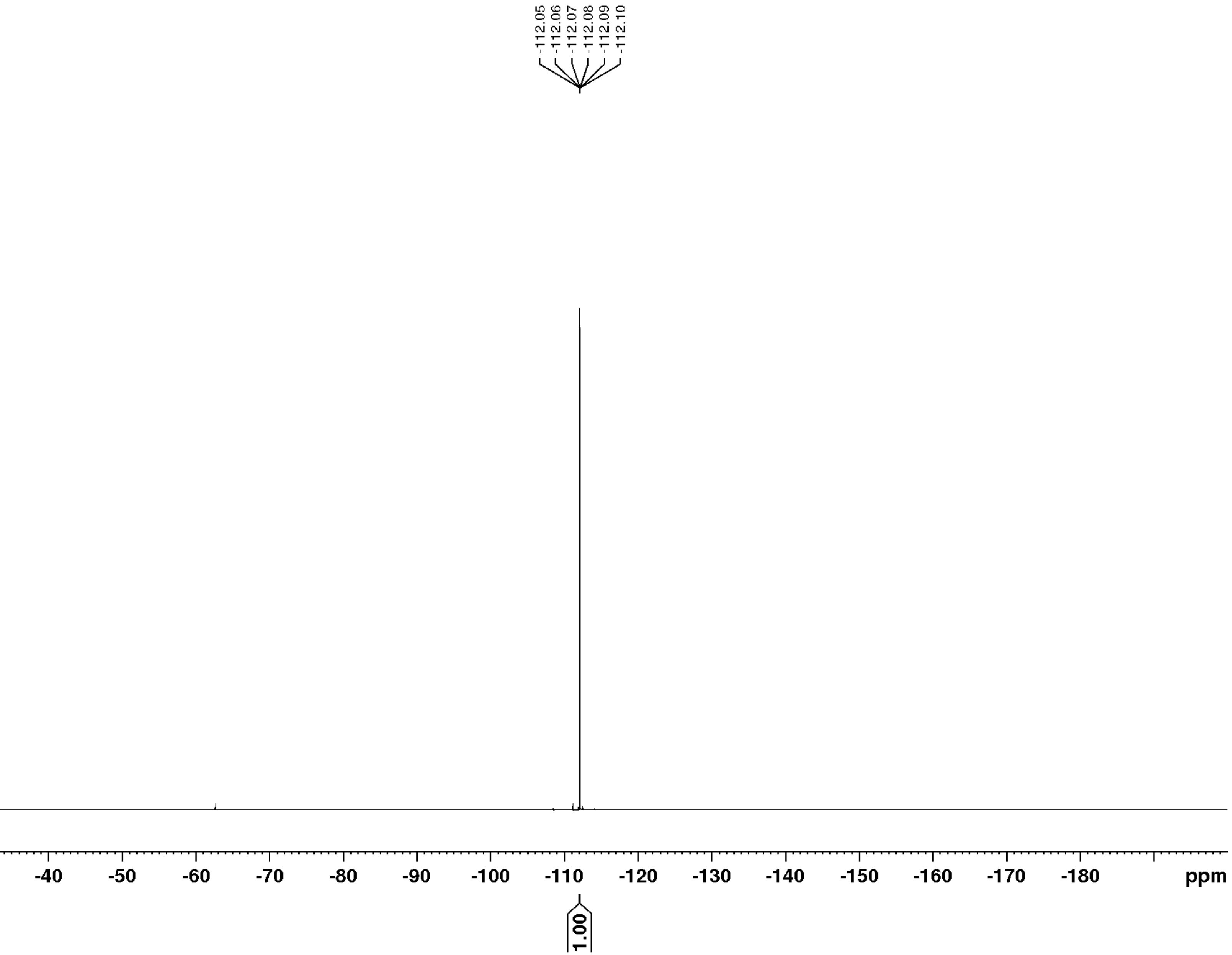

Figure S15. ^{1}H NMR spectrum (500 MHz, $CDCl_3$) of (*E*)-1-(3-fluorophenyl)-2-(4-fluorophenyl)diazene (**6db**).

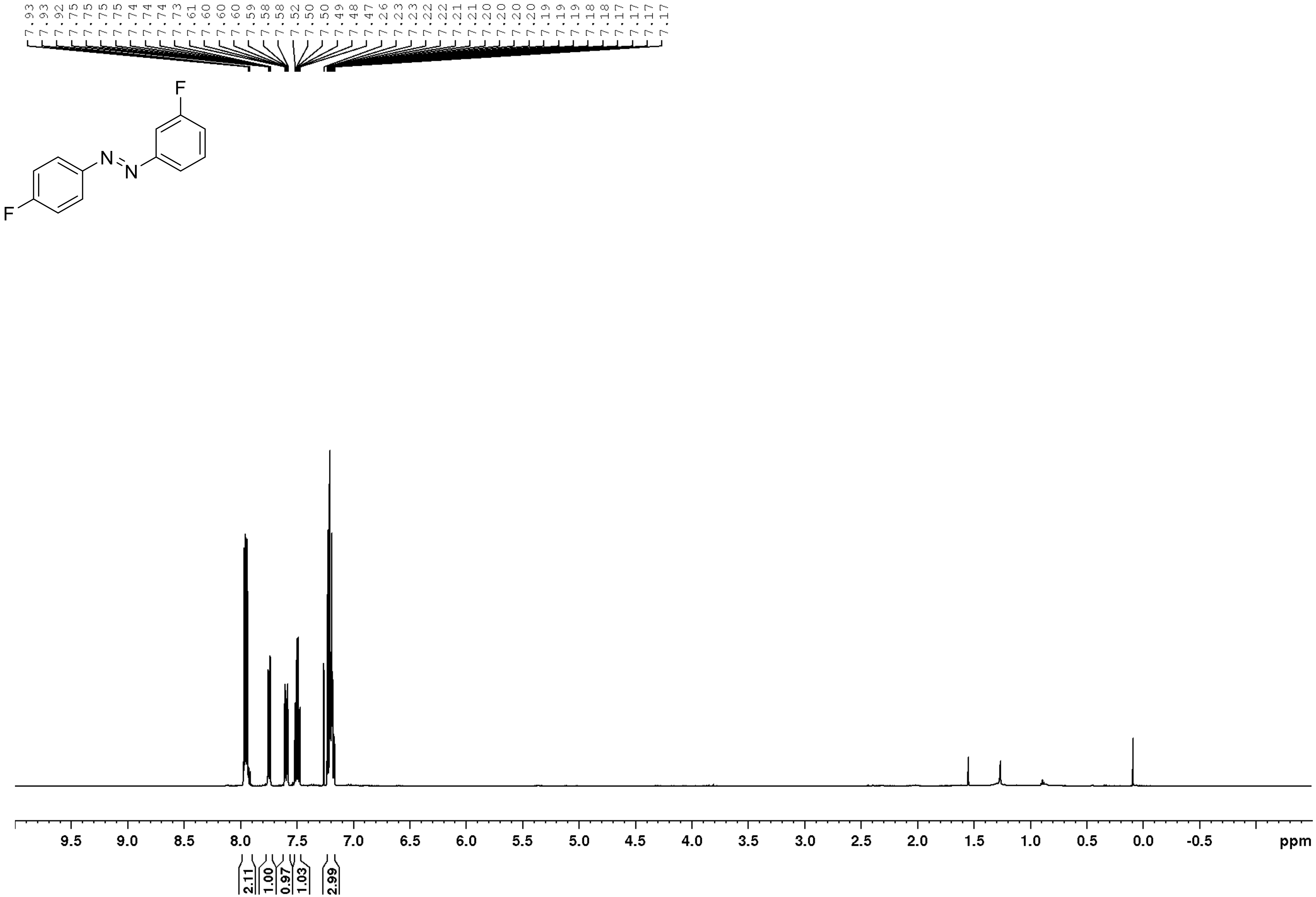

Figure S16. $^{13}C\{^{1}H\}$ NMR spectrum (126 MHz, $CDCl_3$) of (*E*)-1-(3-fluorophenyl)-2-(4-fluorophenyl)diazene (**6db**).

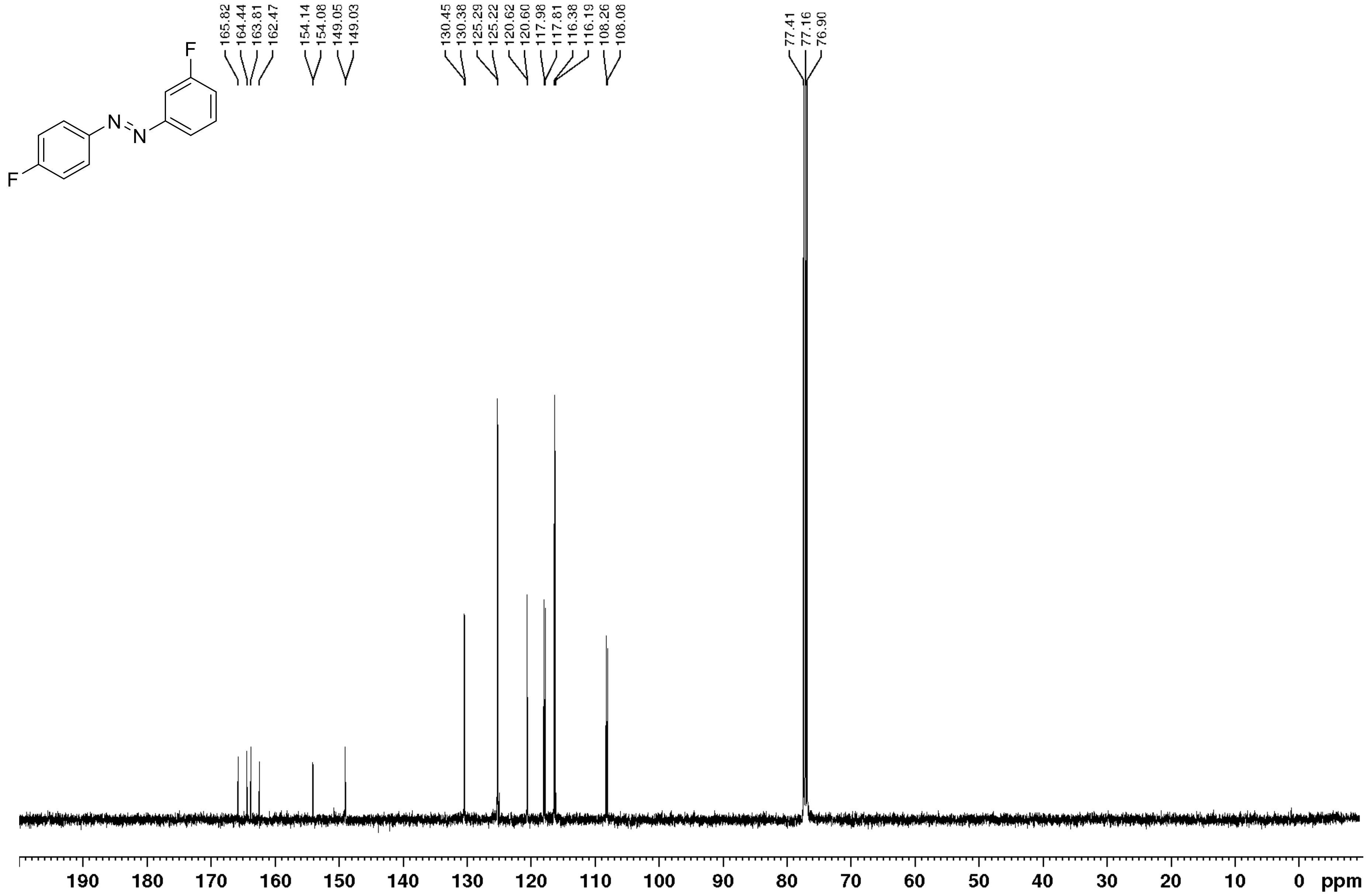

Figure S17. ^{19}F NMR spectrum (471 MHz, $CDCl_3$) of (*E*)-1-(3-fluorophenyl)-2-(4-fluorophenyl)diazene (**6db**).

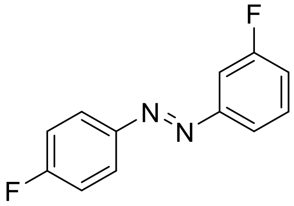

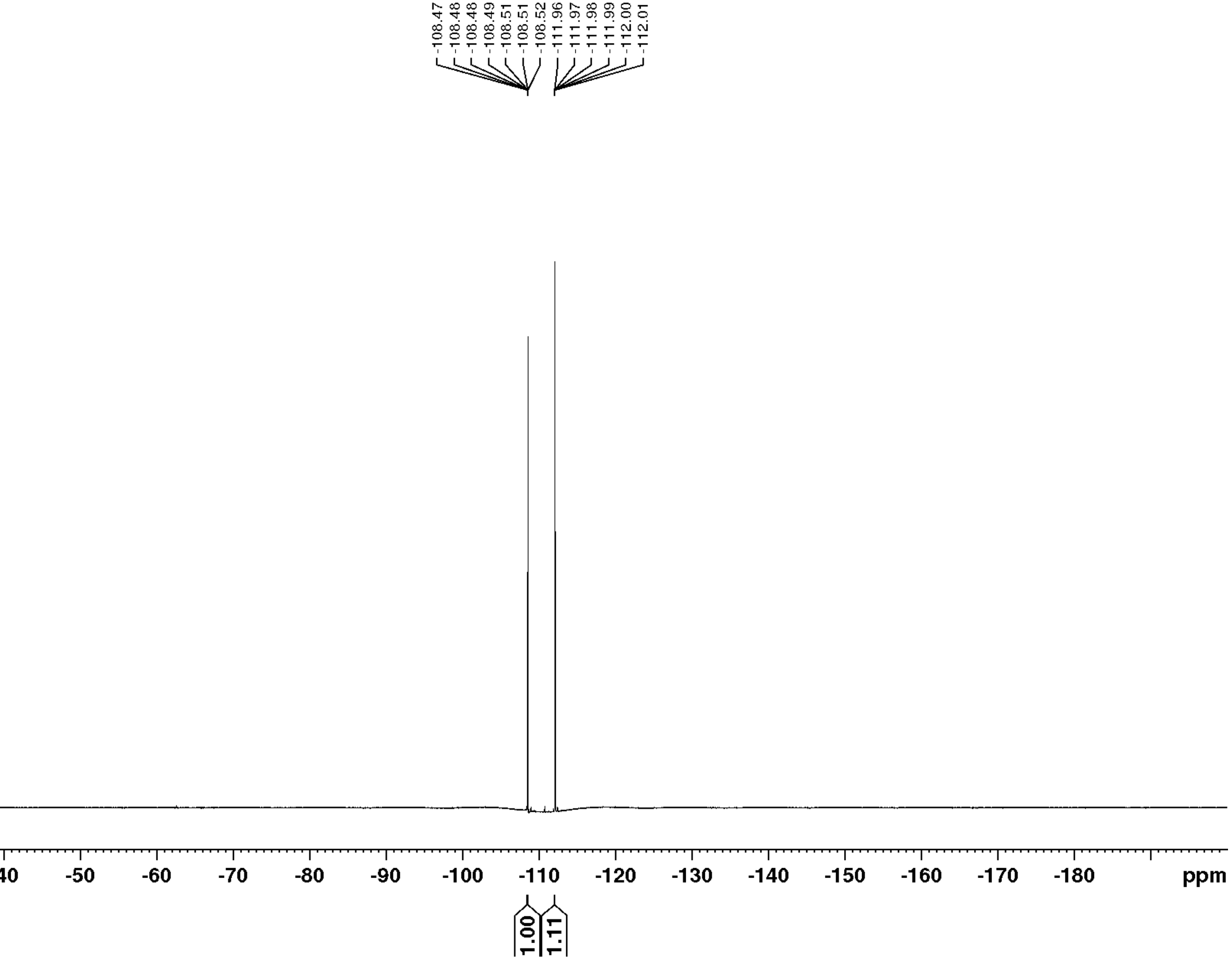

Figure S18. ^{1}H NMR spectrum (500 MHz, $CDCl_3$) of (*E*)-1-(4-chlorophenyl)-2-(3-fluorophenyl)diazene (**6eb**).

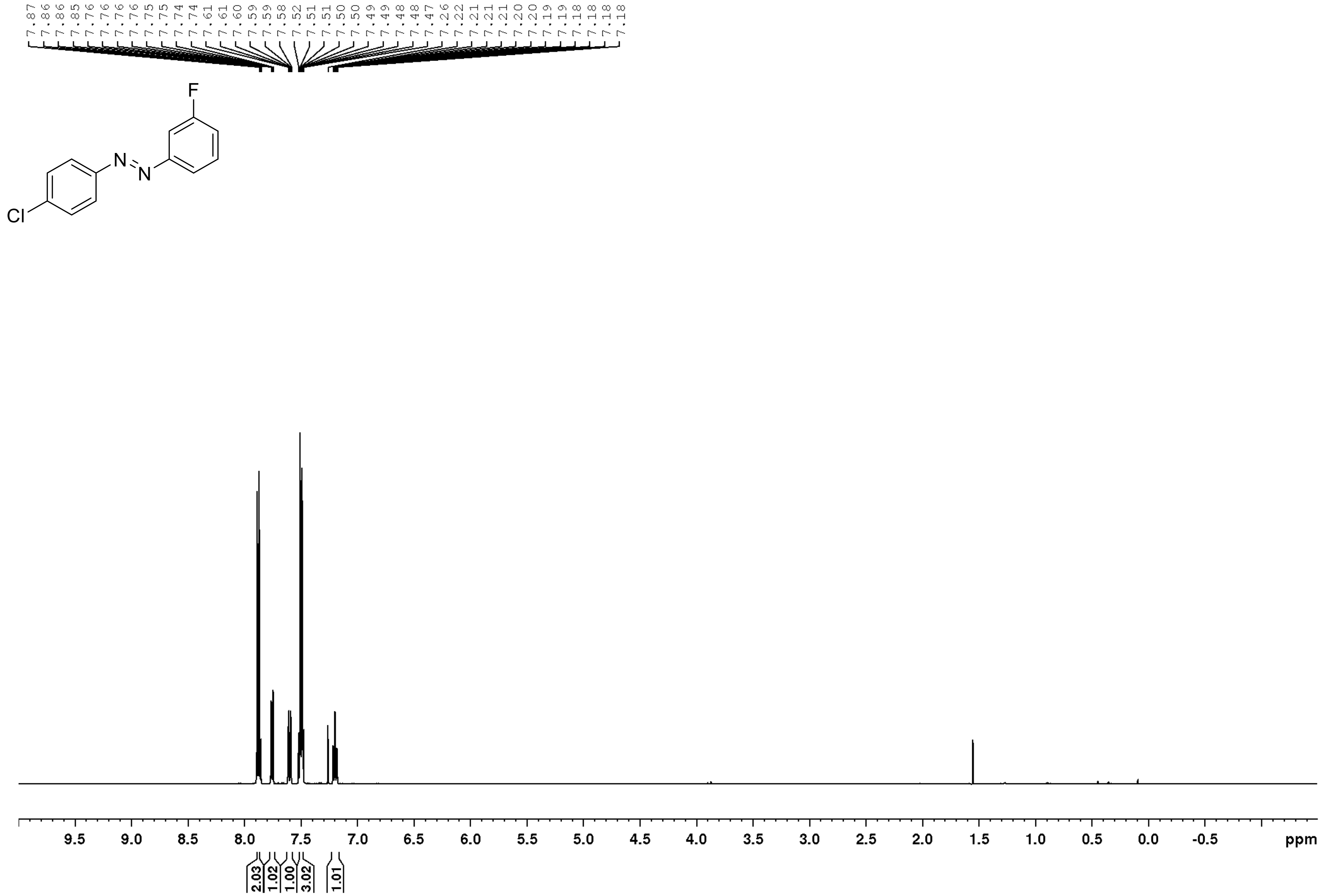

Figure S19. $^{13}C\{^{1}H\}$ NMR spectrum (126 MHz, $CDCl_3$) of (*E*)-1-(4-chlorophenyl)-2-(3-fluorophenyl)diazene (**6eb**).

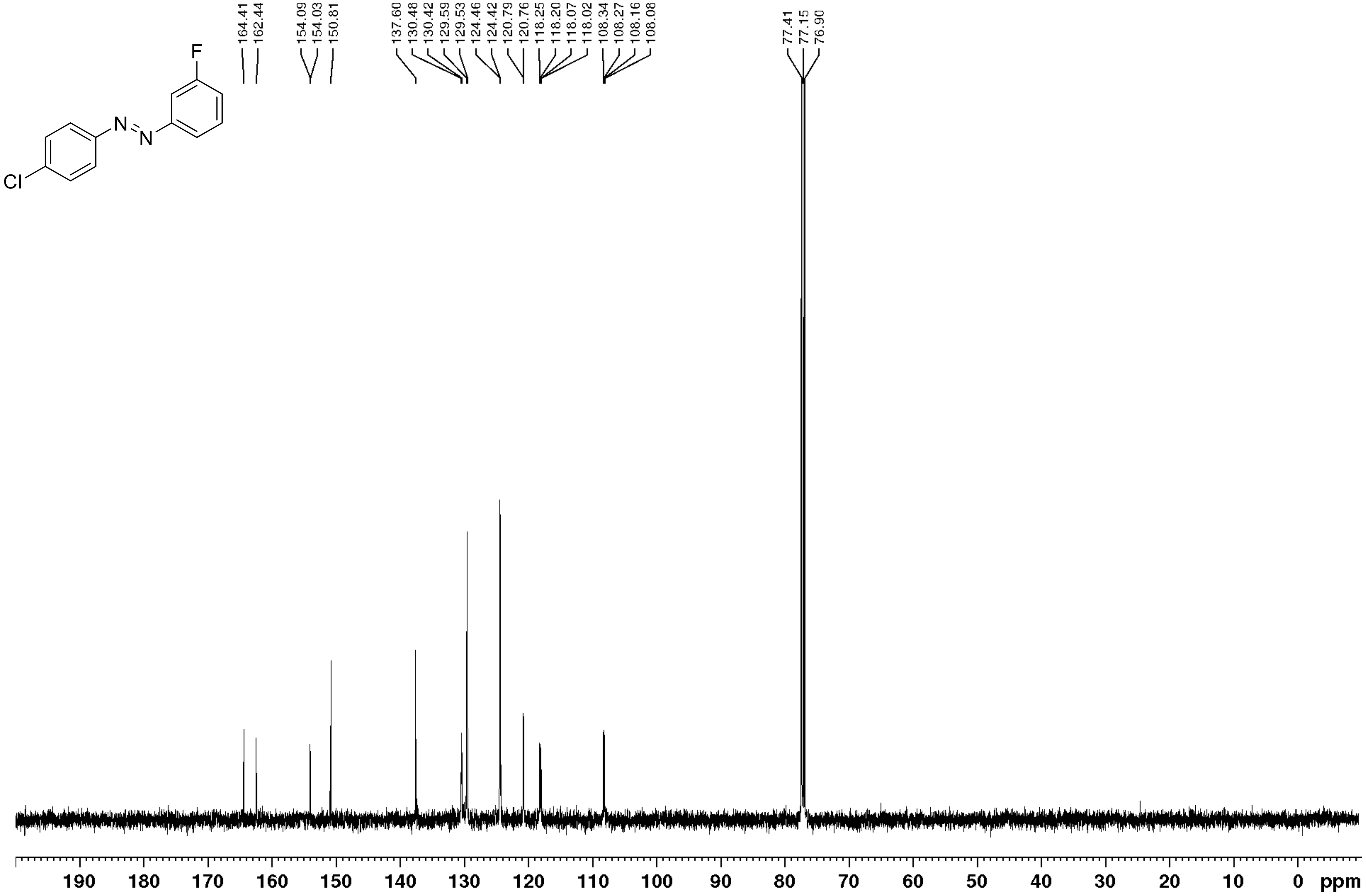

Figure S20. ^{19}F NMR spectrum (471 MHz, $CDCl_3$) of (*E*)-1-(4-chlorophenyl)-2-(3-fluorophenyl)diazene (**6eb**).

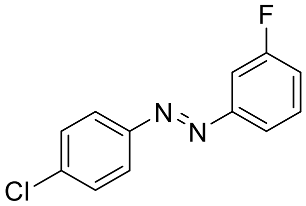

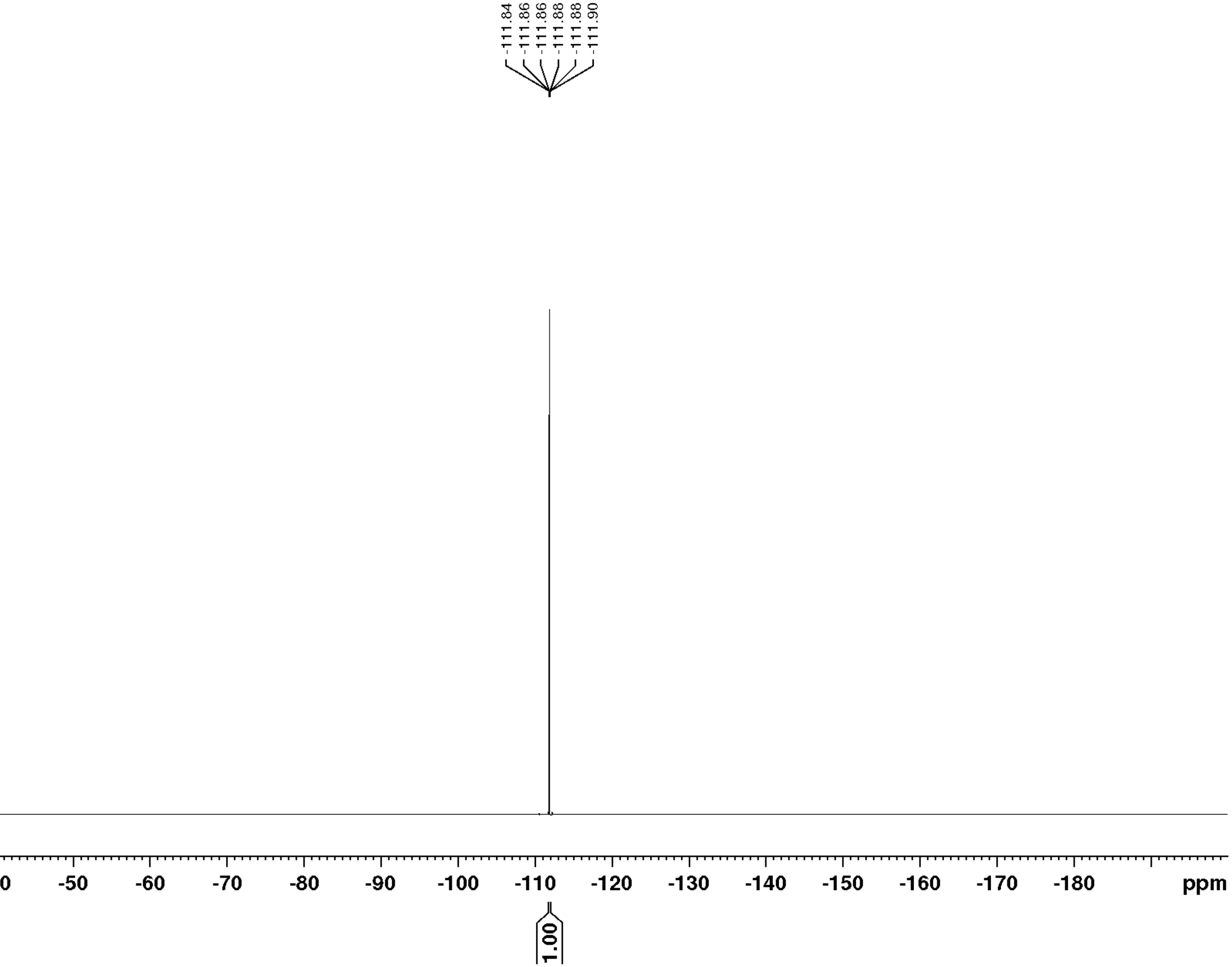

Figure S21. ^{1}H NMR spectrum (500 MHz, $CDCl_3$) of (*E*)-1-(3-fluorophenyl)-2-(4-(trifluoromethyl)phenyl)diazene (**6fb**).

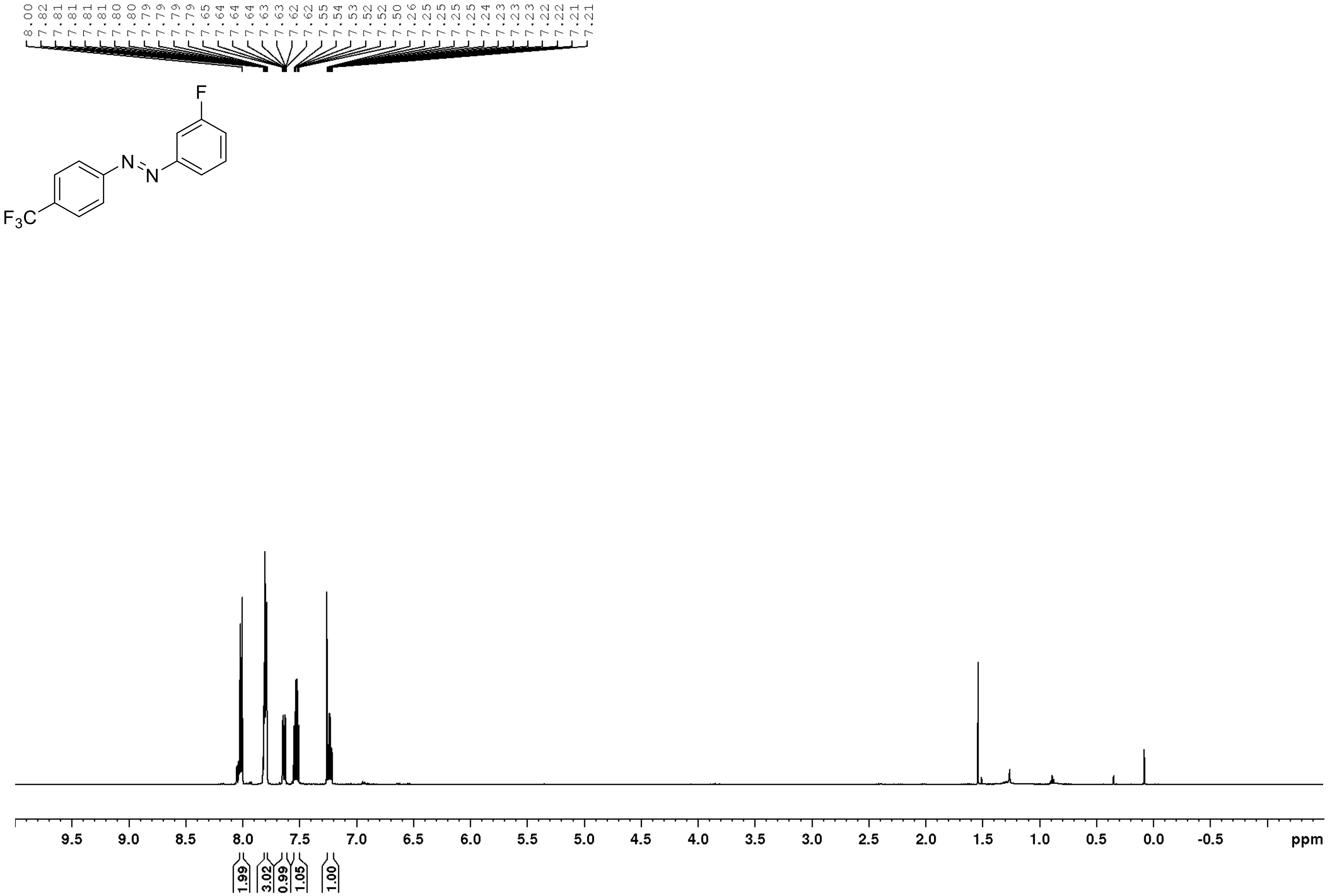

Figure S22. $^{13}C\{^1H\}$ NMR spectrum (126 MHz, $CDCl_3$) of (*E*)-1-(3-fluorophenyl)-2-(4-(trifluoromethyl)phenyl)diazene (**6fb**).

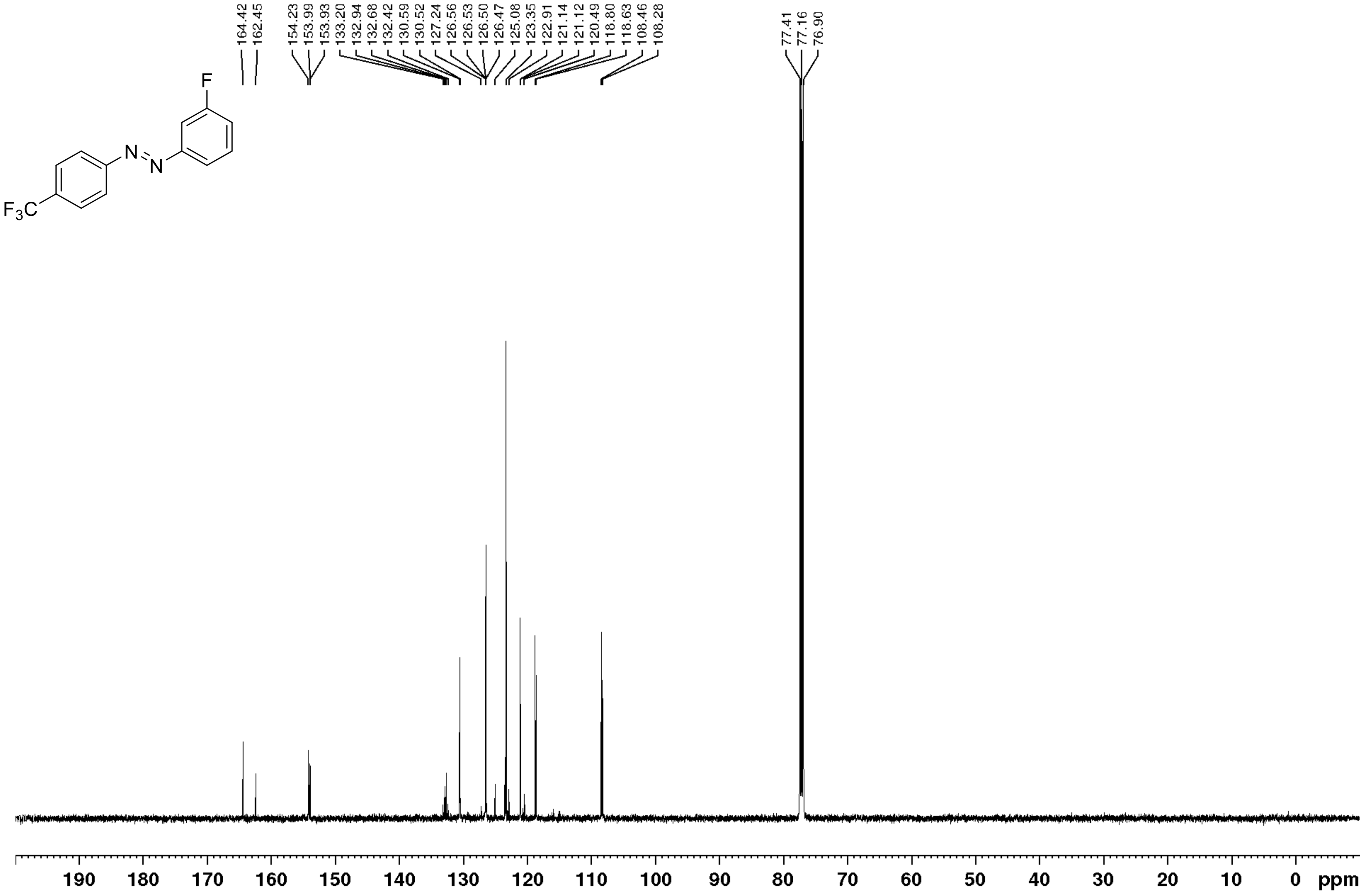

Figure S23. ^{19}F NMR spectrum (471 MHz, $CDCl_3$) of (*E*)-1-(3-fluorophenyl)-2-(4-(trifluoromethyl)phenyl)diazene (**6fb**).

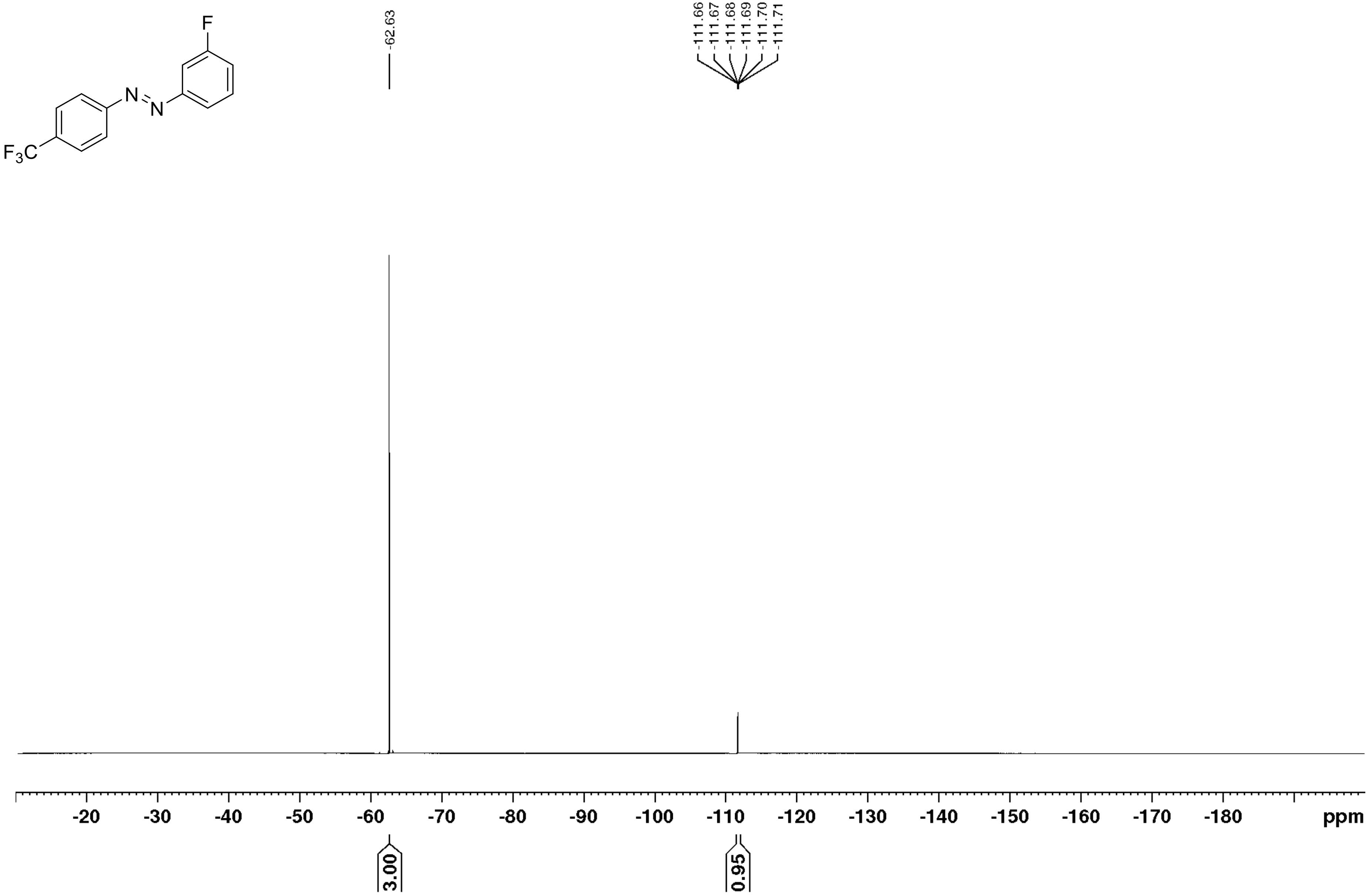

Figure S24. ^{1}H NMR spectrum (500 MHz, $CDCl_3$) of (*E*)-4-((3-fluorophenyl)diazenyl)benzonitrile (**6gb**).

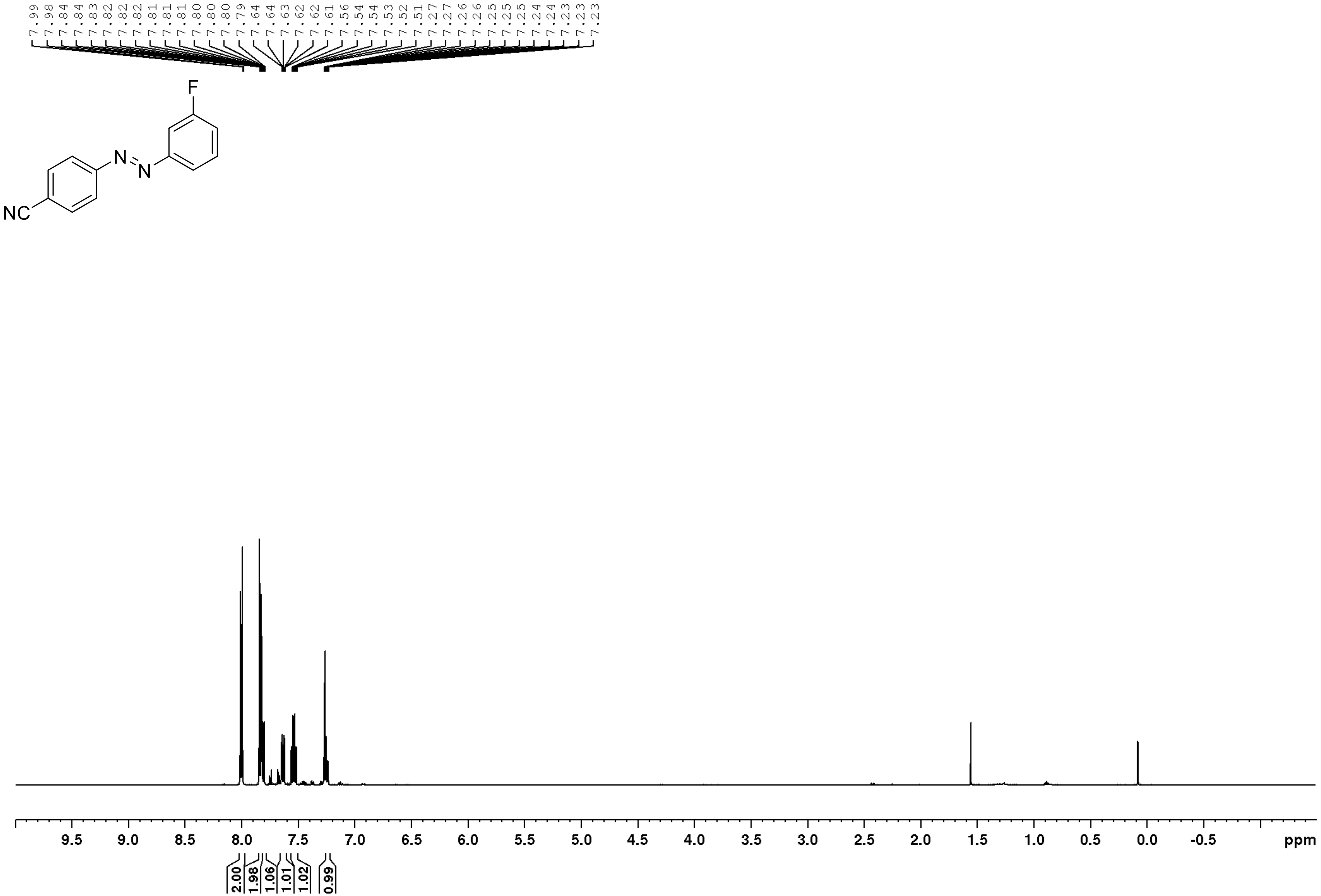

Figure S25. $^{13}C\{^{1}H\}$ NMR spectrum (101 MHz, $CDCl_3$) of (*E*)-4-((3-fluorophenyl)diazenyl)benzonitrile (**6gb**).

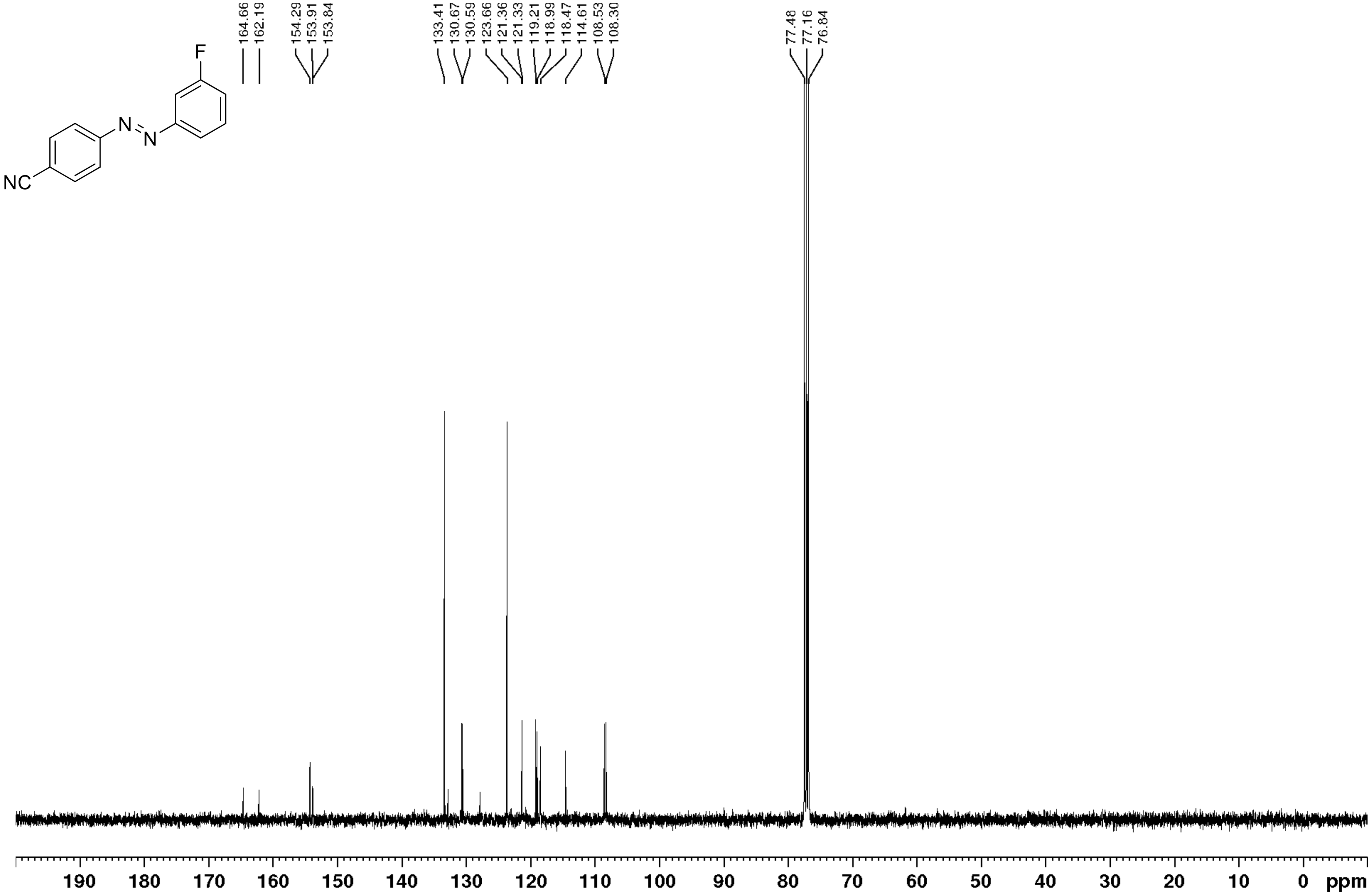

Figure S26. ^{19}F NMR spectrum (471 MHz, $CDCl_3$) of (*E*)-4-((3-fluorophenyl)diazenyl)benzonitrile (**6gb**).

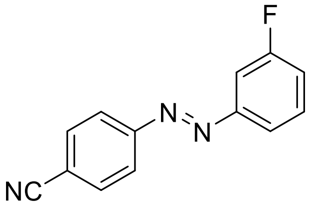

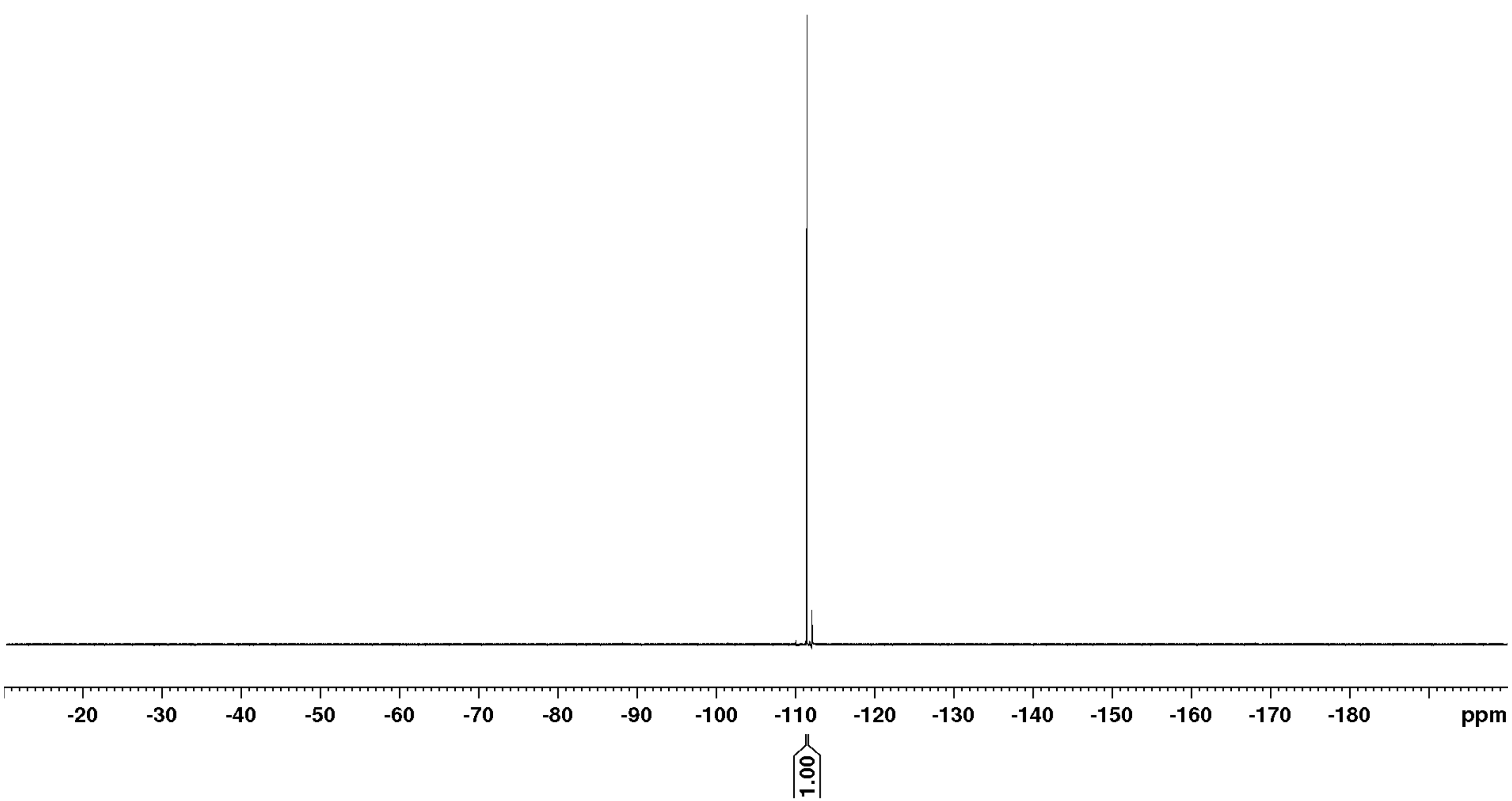

Figure S27. ^{1}H NMR spectrum (500 MHz, $CDCl_3$) of methyl (*E*)-4-((3-fluorophenyl)diazenyl)benzoate (**6hb**).

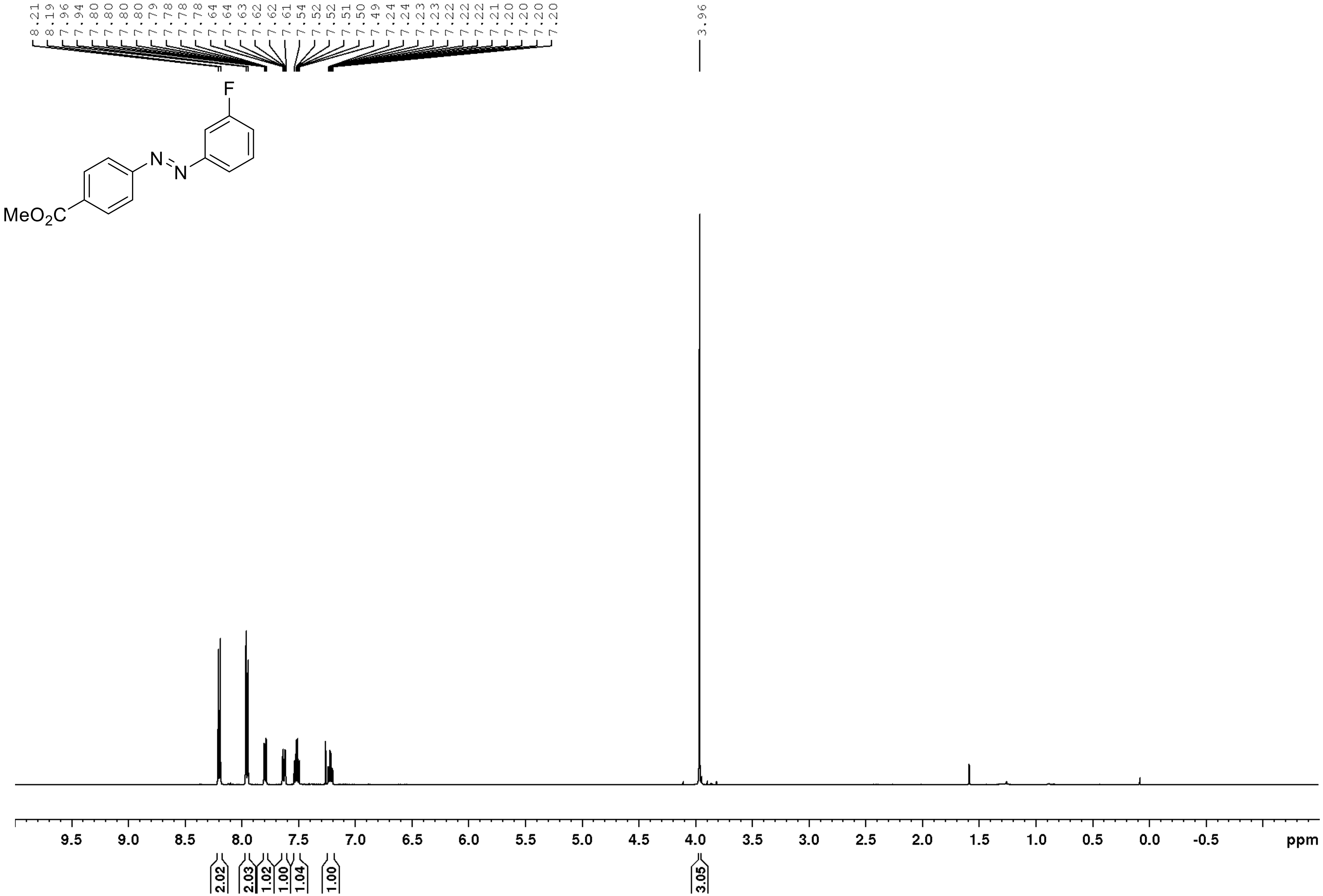

Figure S28. $^{13}C\{^{1}H\}$ NMR spectrum (126 MHz, $CDCl_3$) of methyl (*E*)-4-((3-fluorophenyl)diazenyl)benzoate (**6hb**).

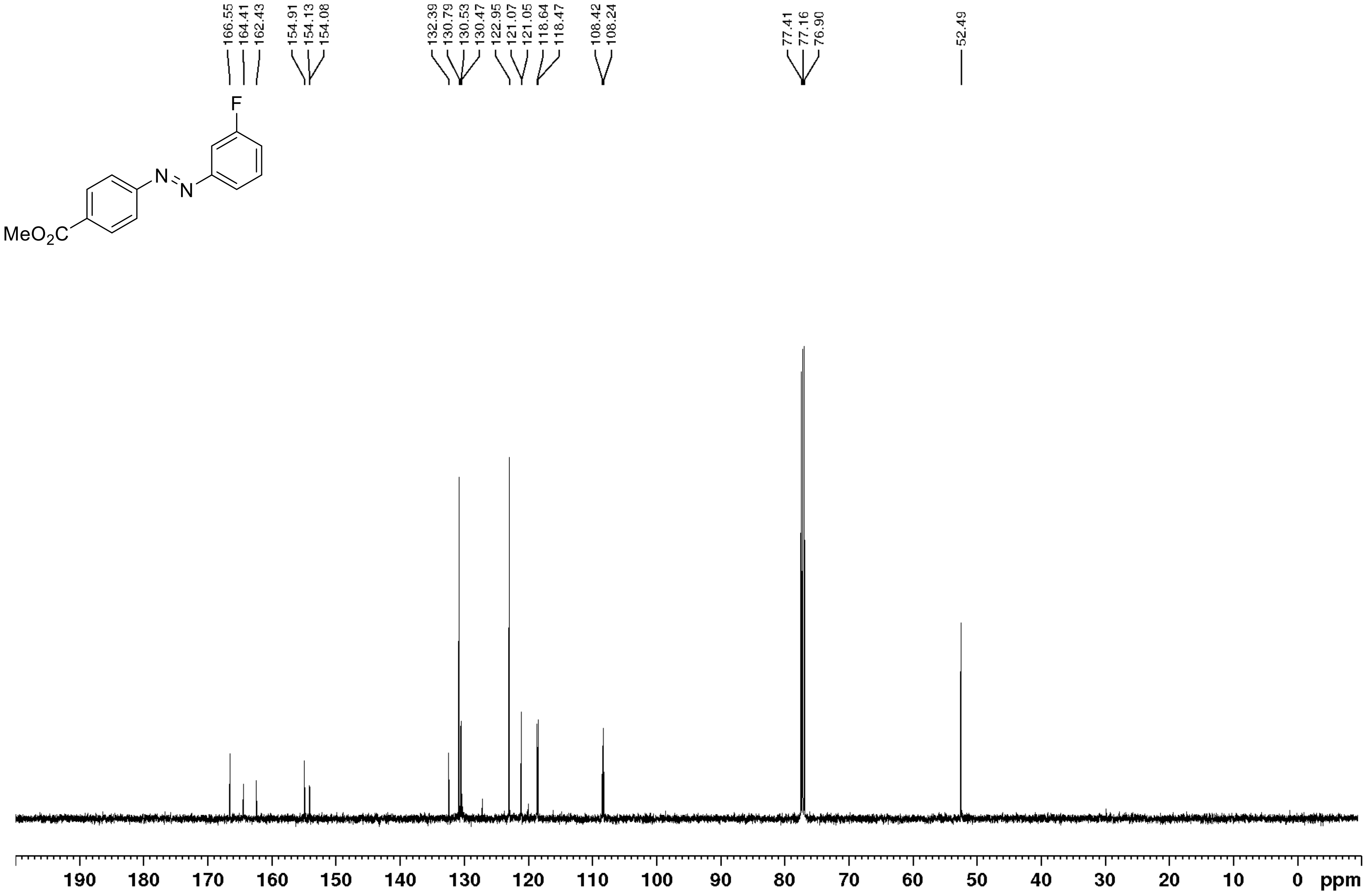

Figure S29. ^{19}F NMR spectrum (471 MHz, $CDCl_3$) of methyl (*E*)-4-((3-fluorophenyl)diazenyl)benzoate (**6hb**).

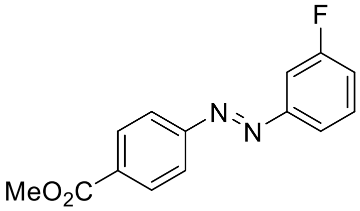

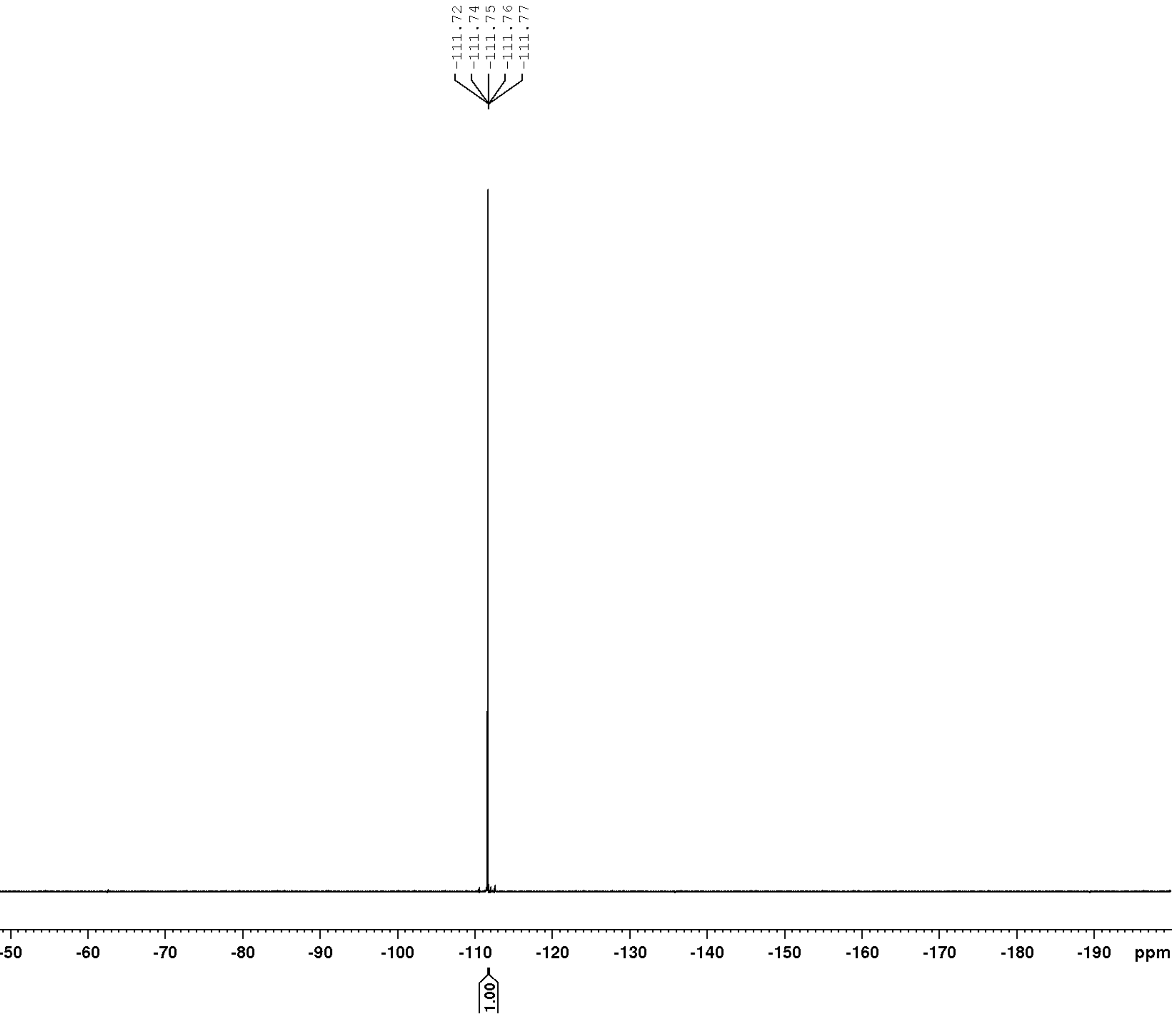

Figure S30. ^{1}H NMR spectrum (500 MHz, $CDCl_3$) of (*E*)-1-(3-chlorophenyl)-2-(3-fluorophenyl)diazene (**6ib**).

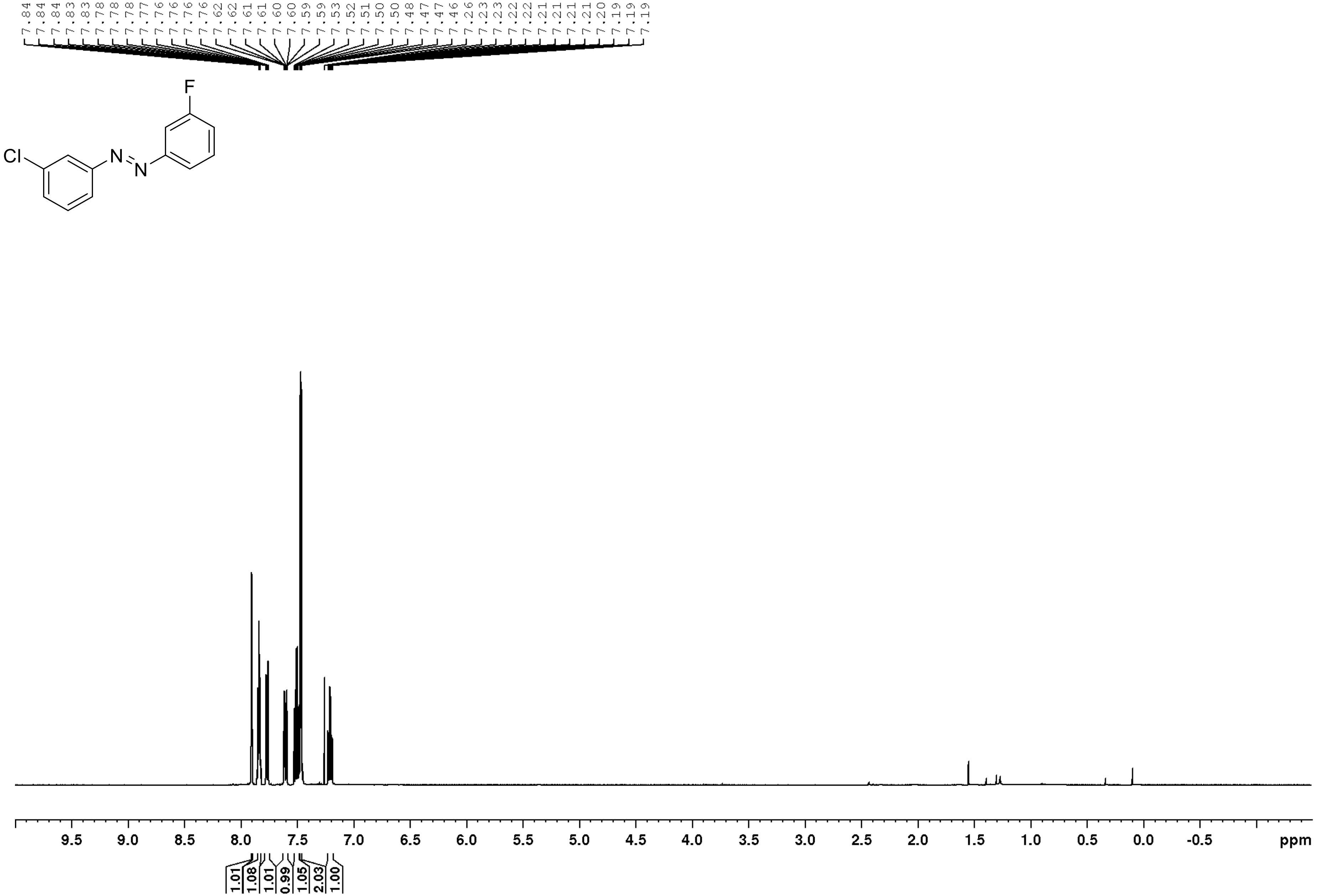

Figure S31. $^{13}C\{^{1}H\}$ NMR spectrum (101 MHz, $CDCl_3$) of (*E*)-1-(3-chlorophenyl)-2-(3-fluorophenyl)diazene (**6ib**).

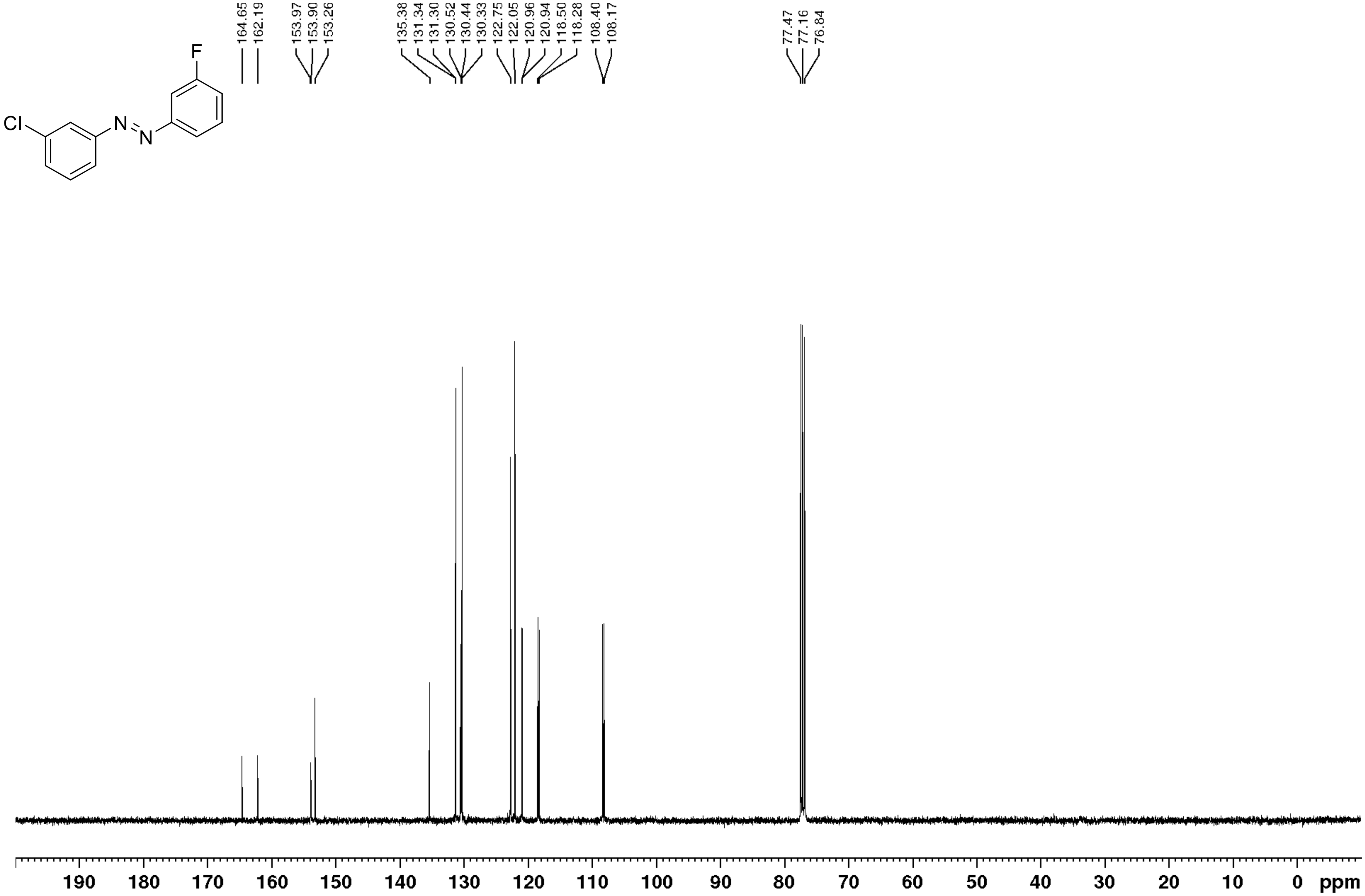

Figure S32. ^{19}F NMR spectrum (471 MHz, $CDCl_3$) of (*E*)-1-(3-chlorophenyl)-2-(3-fluorophenyl)diazene (**6ib**).

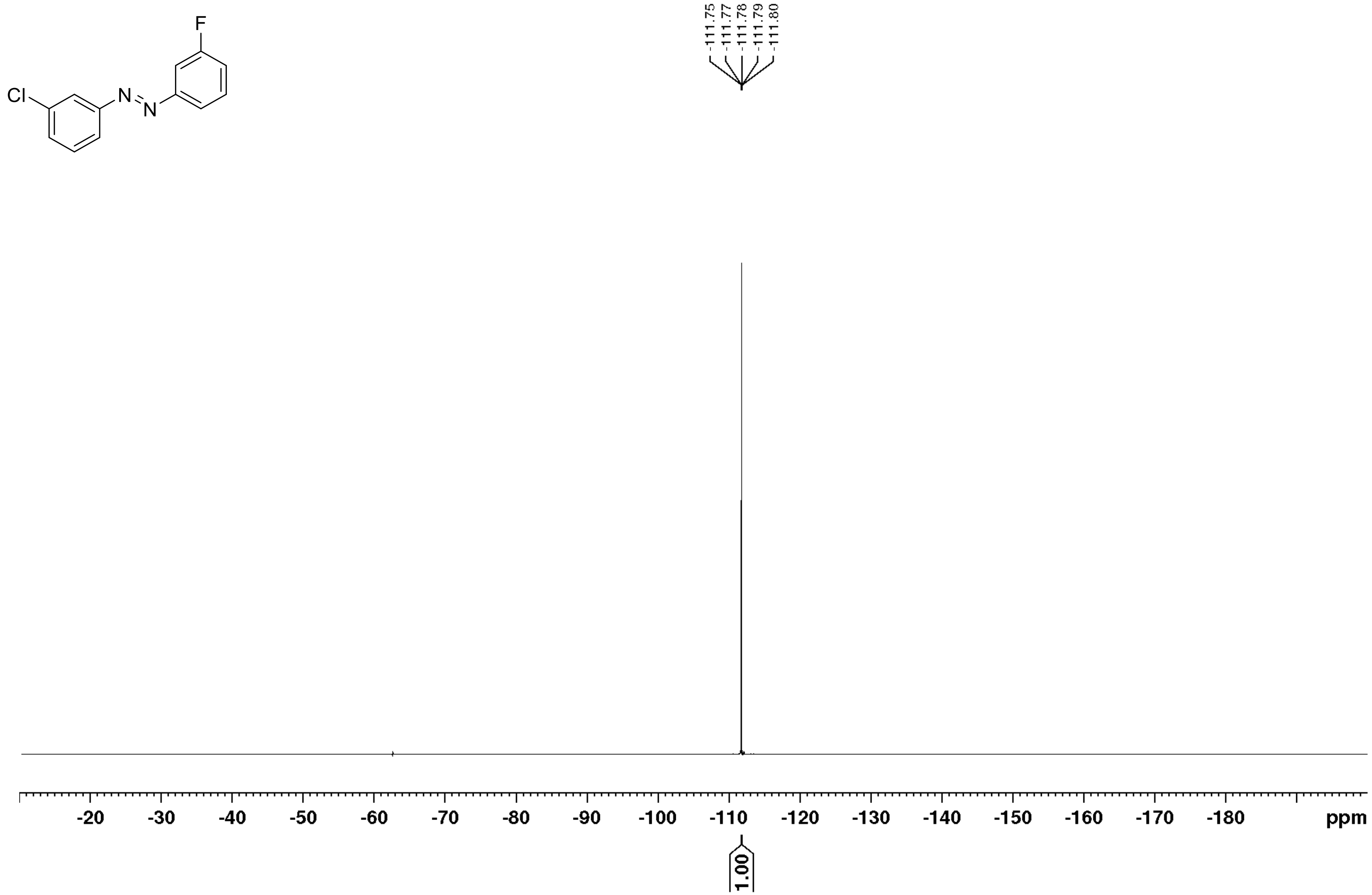

Figure S33. ^{1}H NMR spectrum (500 MHz, $CDCl_3$) of (*E*)-1-(4-fluoro-2,6-dimethylphenyl)-2-(3-fluorophenyl)diazene (**6jb**).

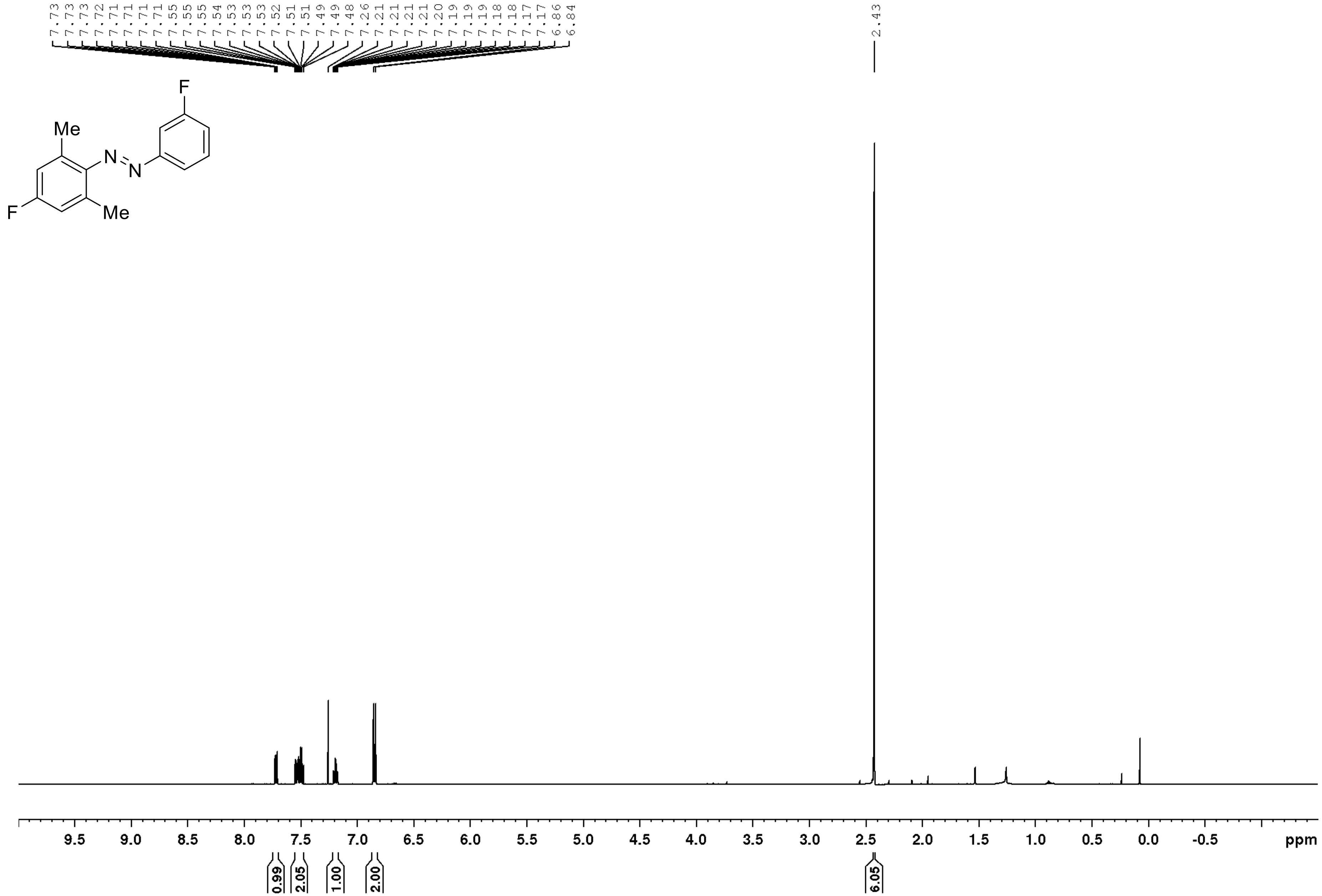

Figure S34. $^{13}C\{^{1}H\}$ NMR spectrum (126 MHz, $CDCl_3$) of (*E*)-1-(4-fluoro-2,6-dimethylphenyl)-2-(3-fluorophenyl)diazene (**6jb**).

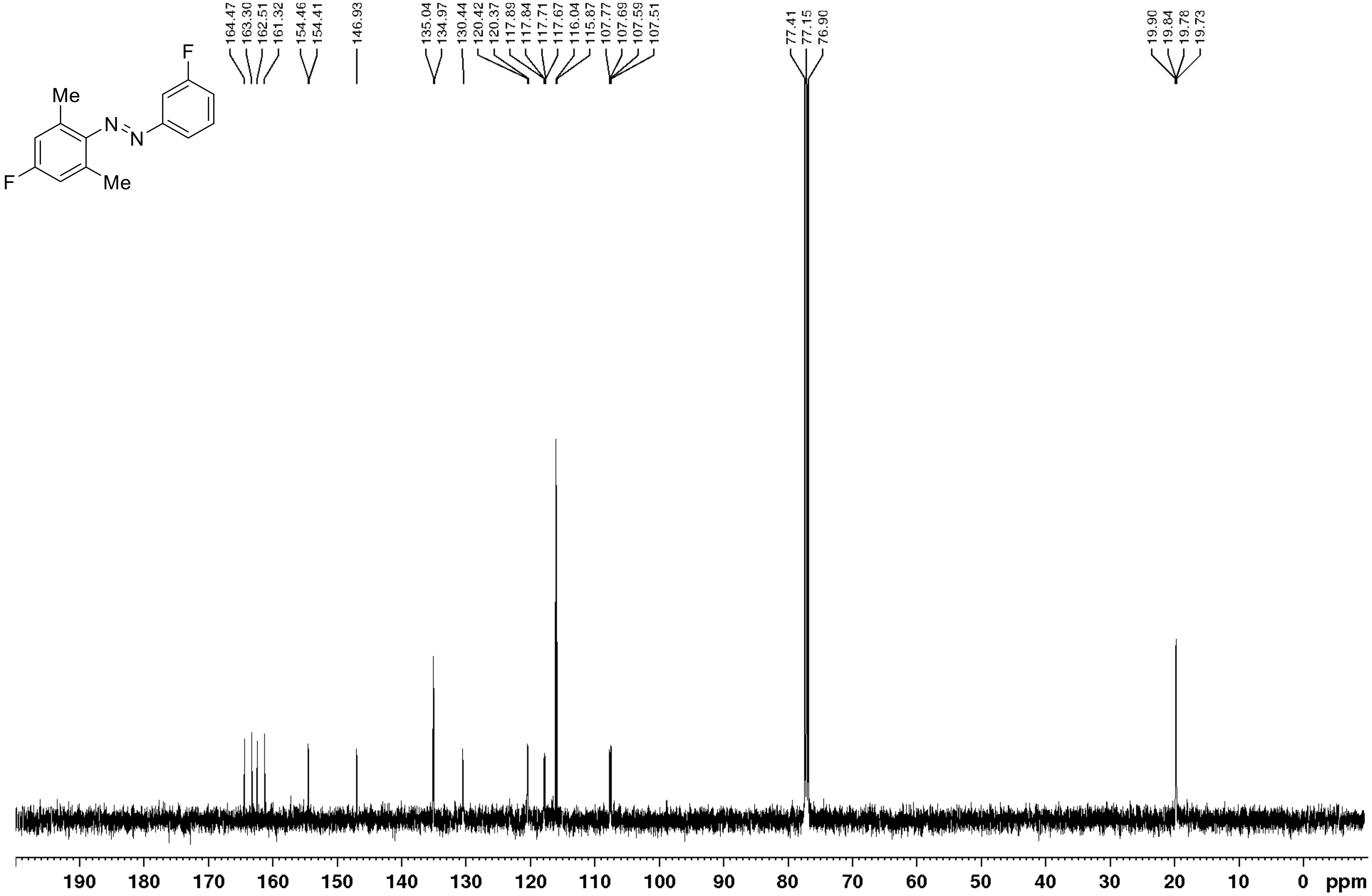

Figure S35. ^{19}F NMR spectrum (471 MHz, $CDCl_3$) of (*E*)-1-(4-fluoro-2,6-dimethylphenyl)-2-(3-fluorophenyl)diazene (**6jb**).

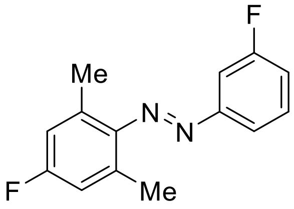

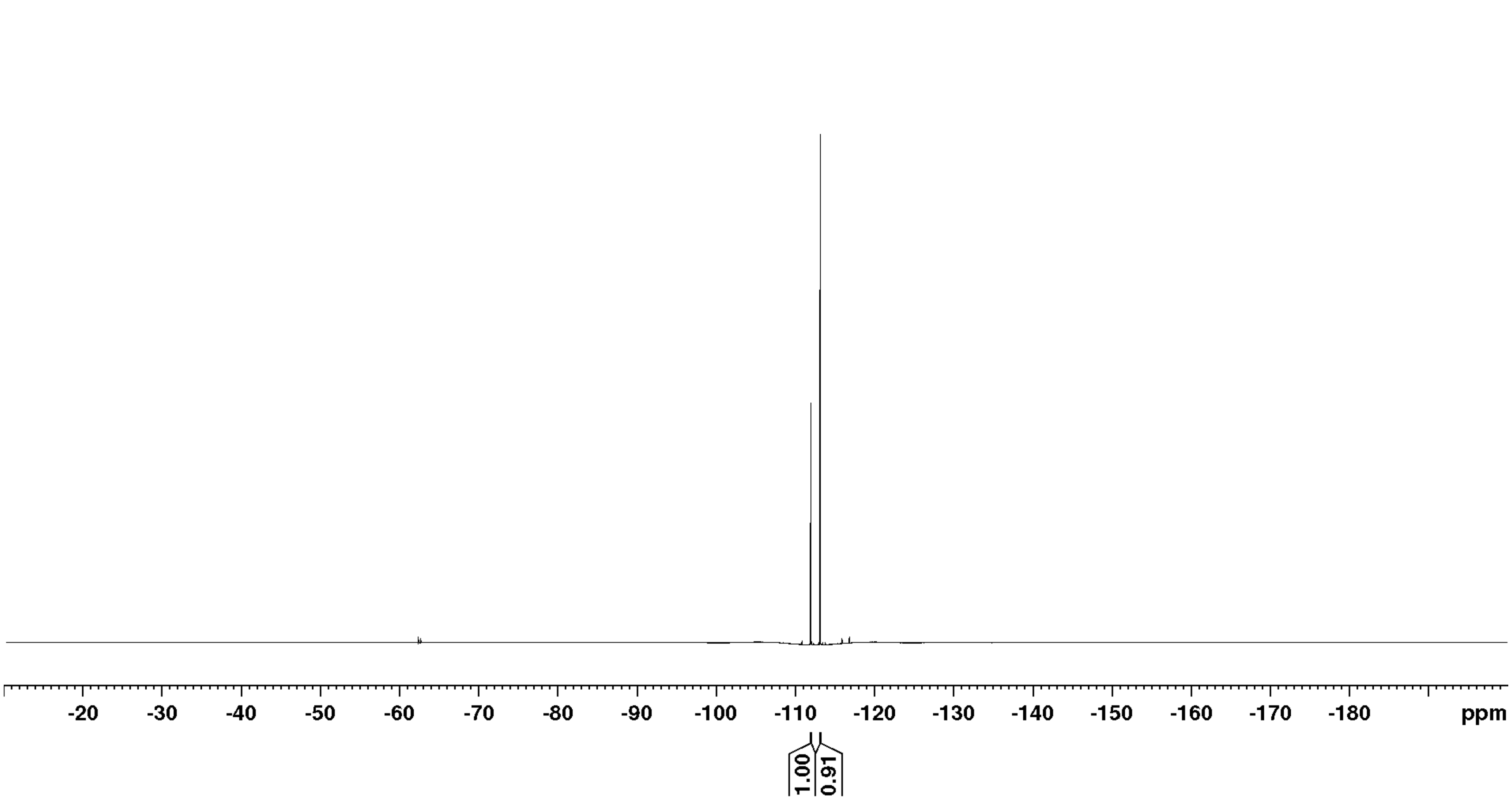

Figure S36. ^{1}H NMR spectrum (500 MHz, $CDCl_3$) of (*E*)-1-(3-fluorophenyl)-2-(naphthalen-2-yl)diazene (**6kb**).

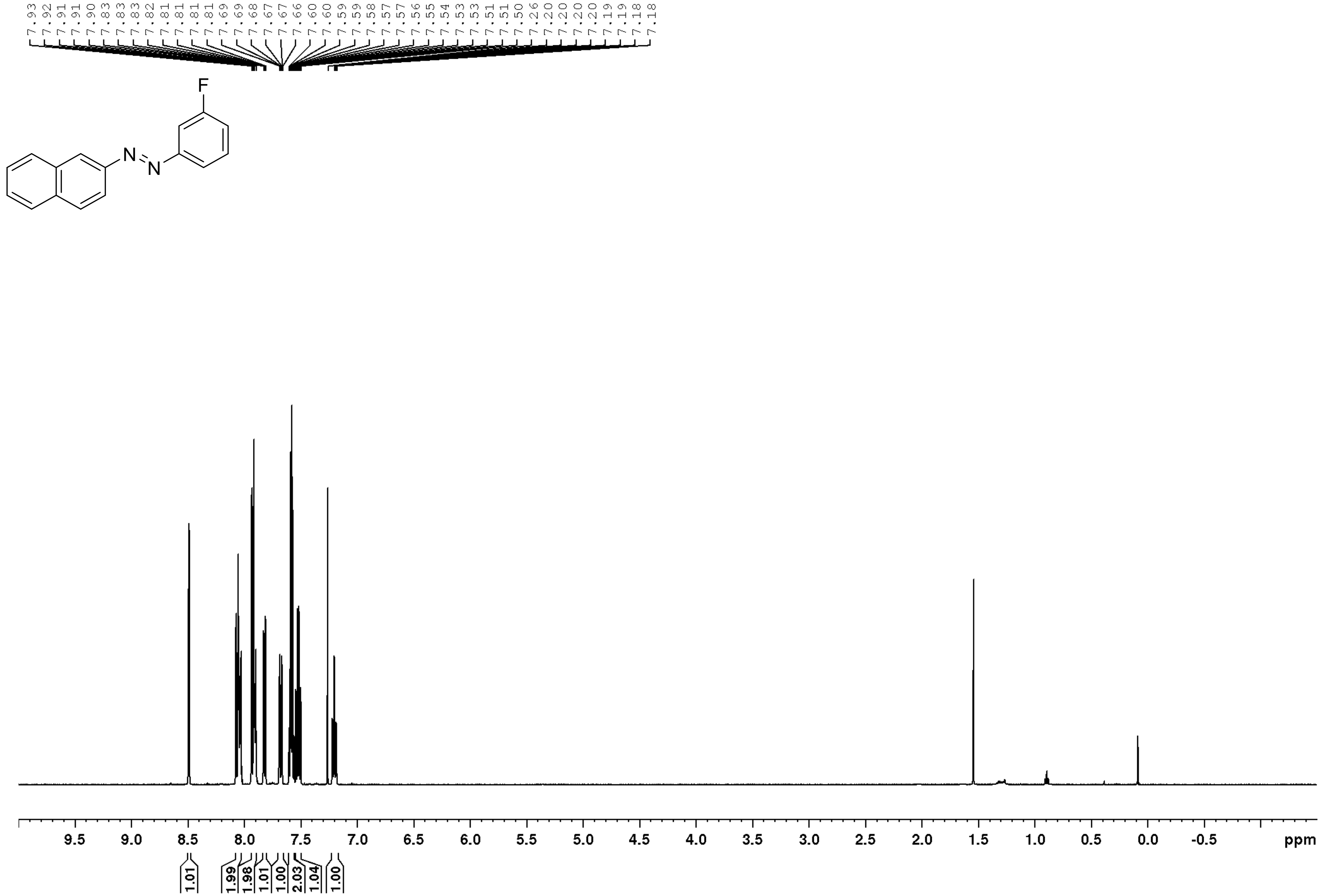

Figure S37. $^{13}C\{^{1}H\}$ NMR spectrum (126 MHz, $CDCl_3$) of (*E*)-1-(3-fluorophenyl)-2-(naphthalen-2-yl)diazene (**6kb**).

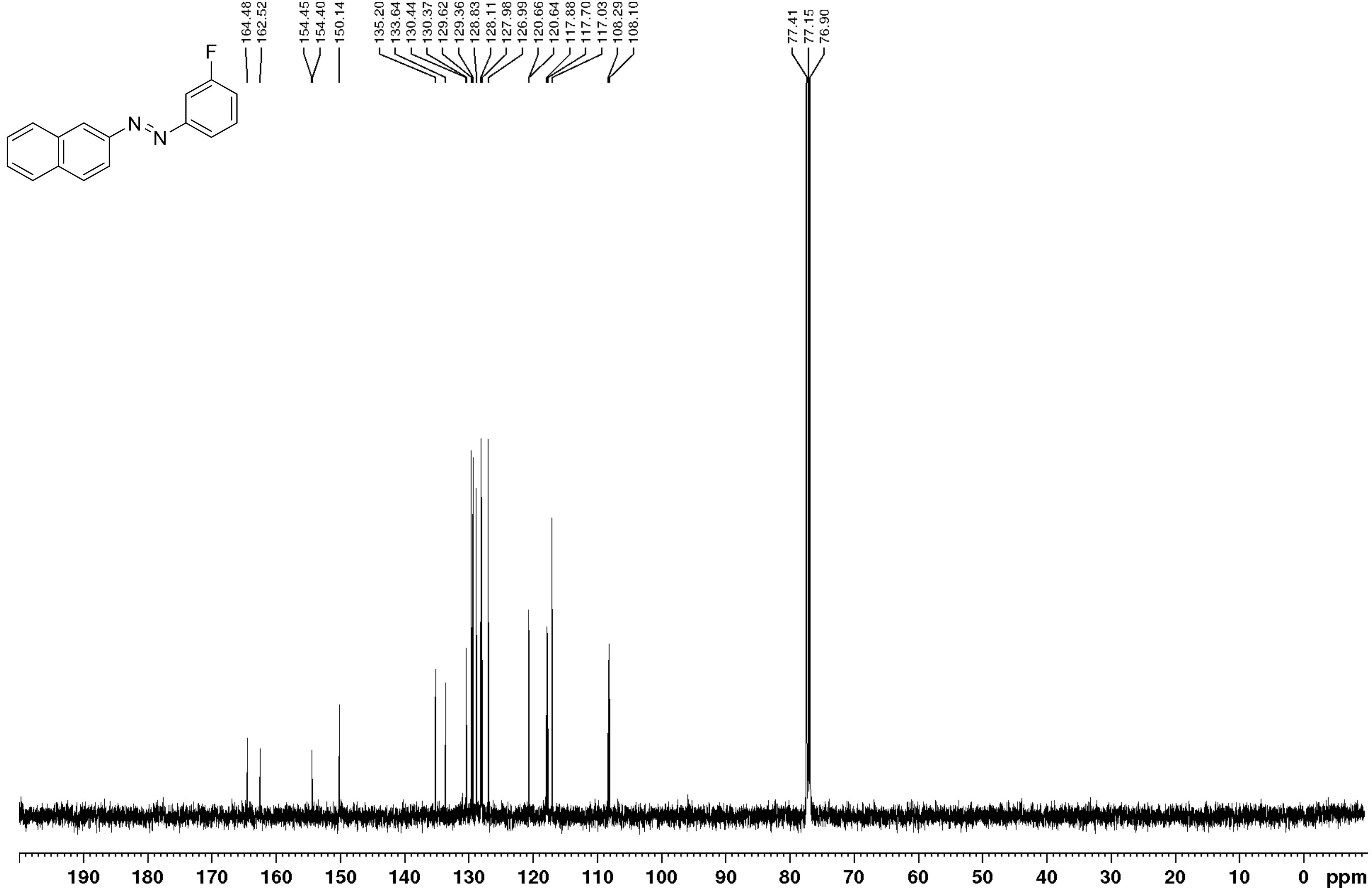

Figure S38. ^{19}F NMR spectrum (471 MHz, $CDCl_3$) of (*E*)-1-(3-fluorophenyl)-2-(naphthalen-2-yl)diazene (**6kb**).

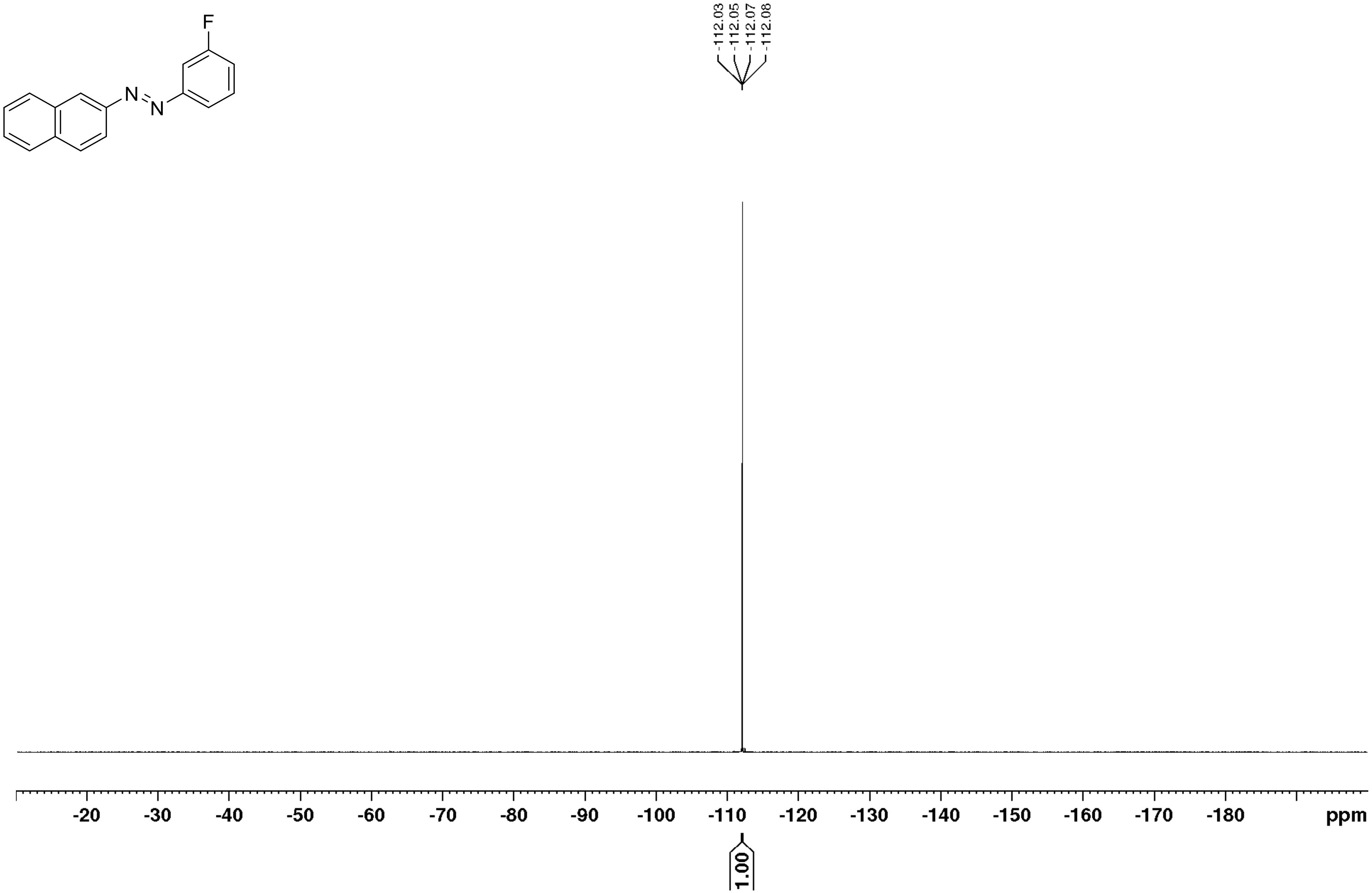

Figure S39. ^{1}H NMR spectrum (500 MHz, $CDCl_3$) of 1,3-bis((*E*)-(3-fluorophenyl)diazenyl)benzene (**6lb**).

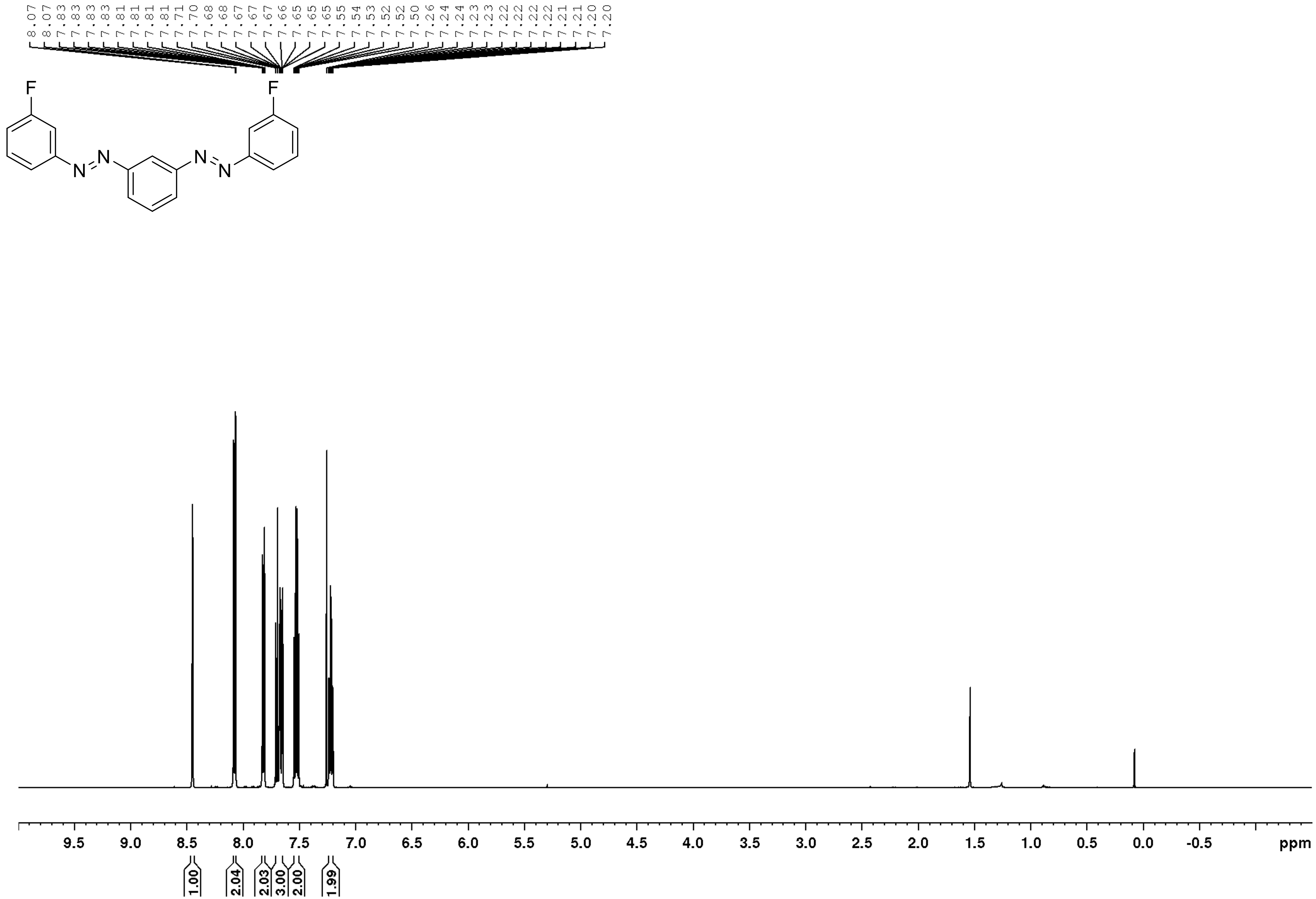

Figure S40. $^{13}C\{^{1}H\}$ NMR spectrum (126 MHz, $CDCl_3$) of 1,3-bis((*E*)-(3-fluorophenyl)diazenyl)benzene (**6lb**).

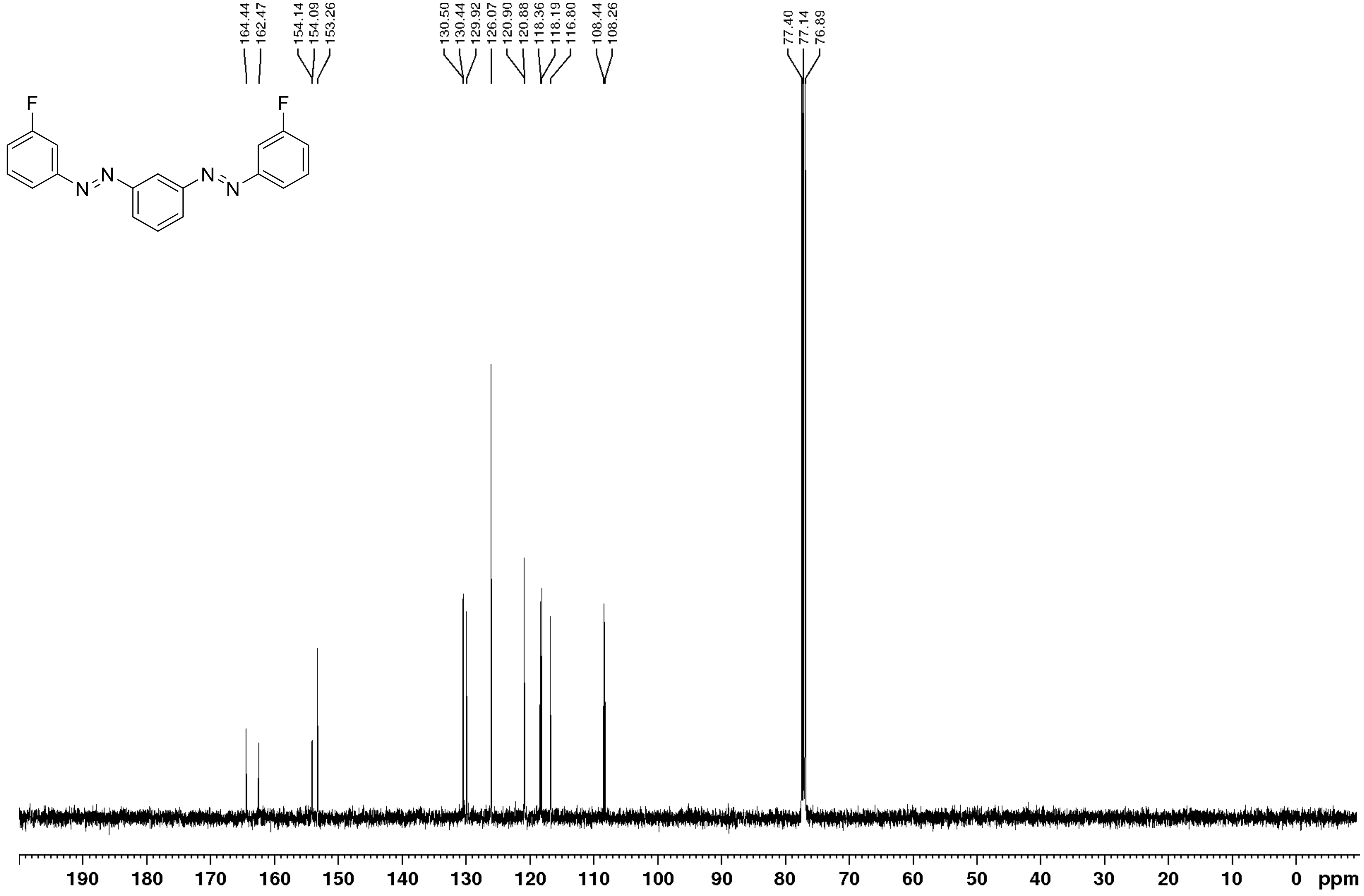

Figure S41. ^{19}F NMR spectrum (471 MHz, $CDCl_3$) of 1,3-bis((*E*)-(3-fluorophenyl)diazenyl)benzene (**6lb**).

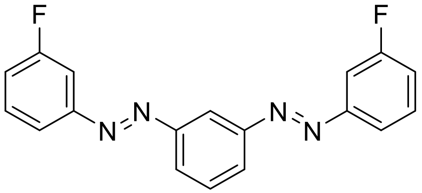

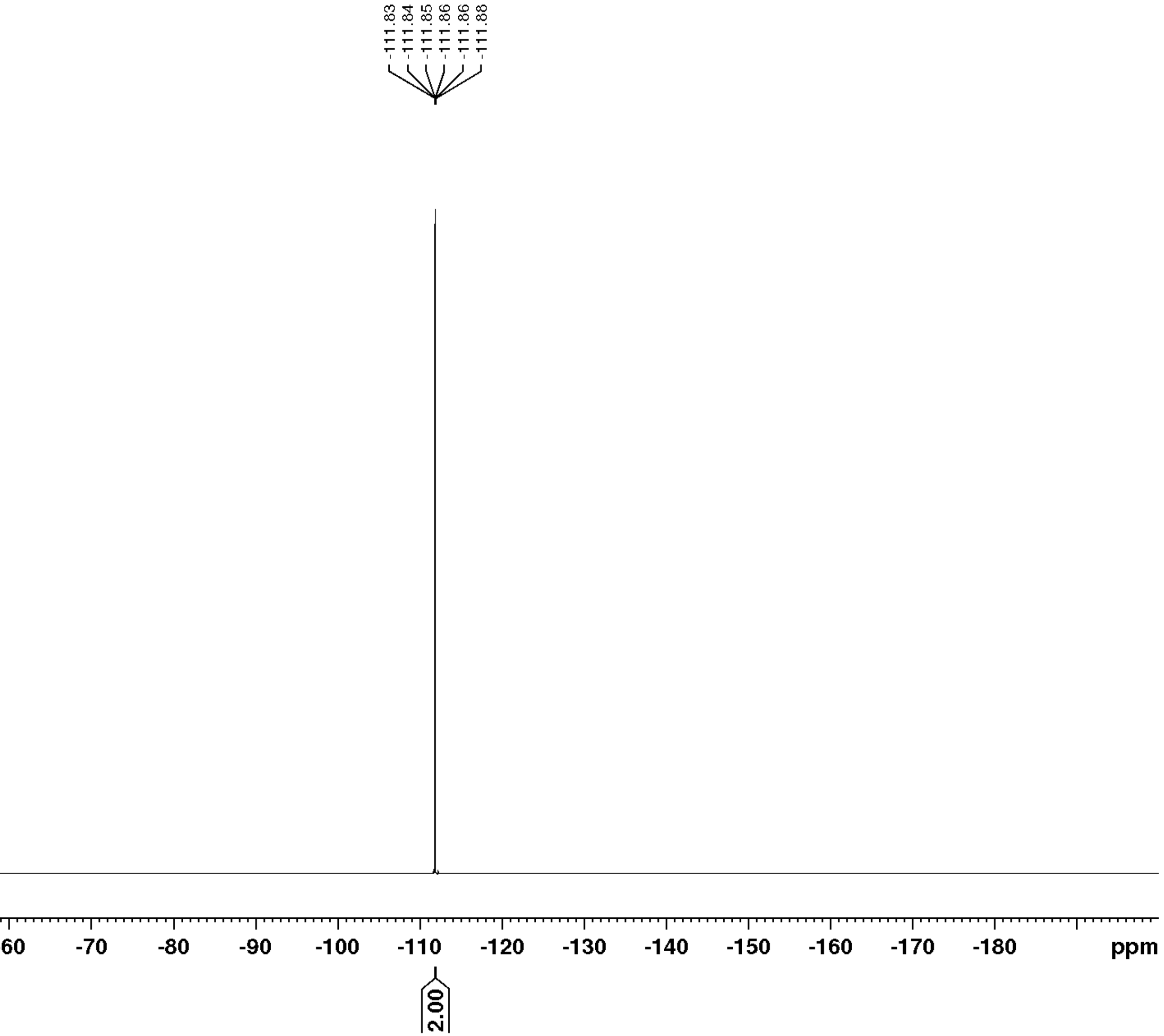

Figure S42. ¹H NMR spectrum (400 MHz, $CDCl_3$) of (*E*)-1-(*m*-tolyl)-2-(*p*-tolyl)diazene (**6ac**).

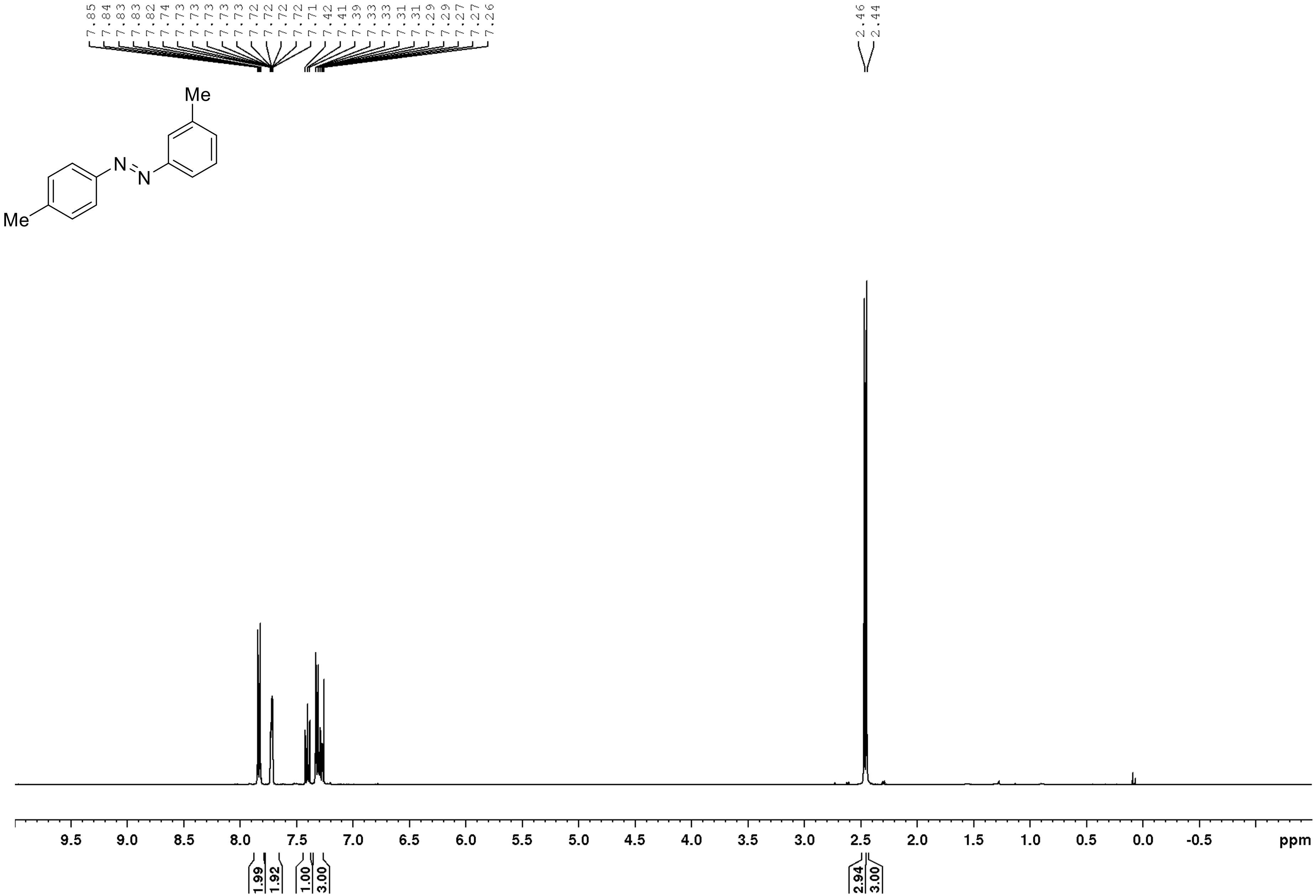

Figure S43. $^{13}C\{^{1}H\}$ NMR spectrum (101 MHz, $CDCl_3$) of (*E*)-1-(*m*-tolyl)-2-(*p*-tolyl)diazene (**6ac**).

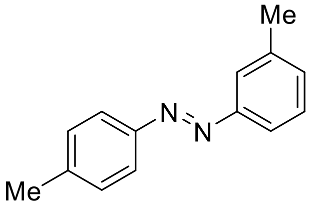

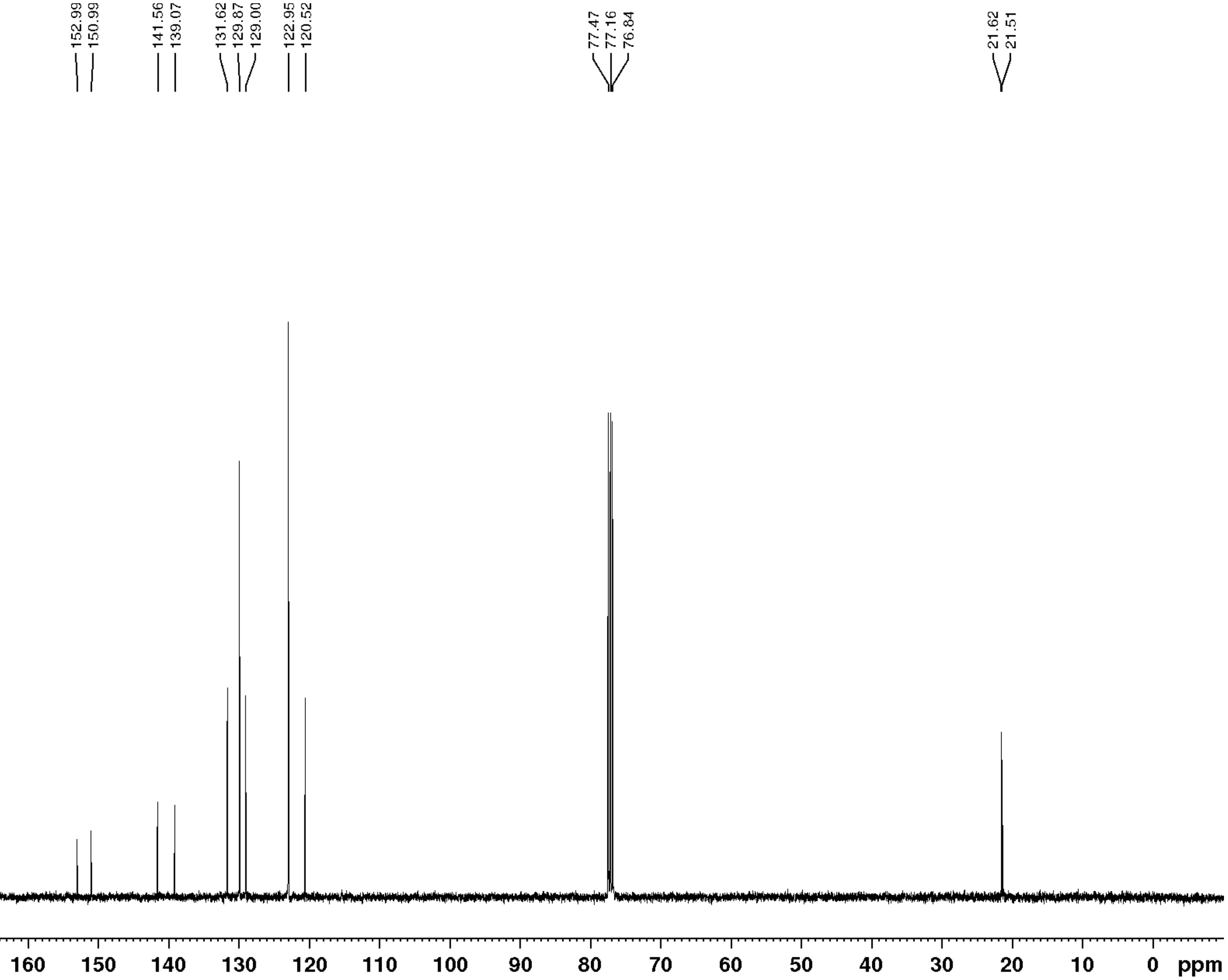

Figure S44. ^{1}H NMR spectrum (500 MHz, $CDCl_3$) of (*E*)-1-(2-methoxyphenyl)-2-(*p*-tolyl)diazene (**6ad**).

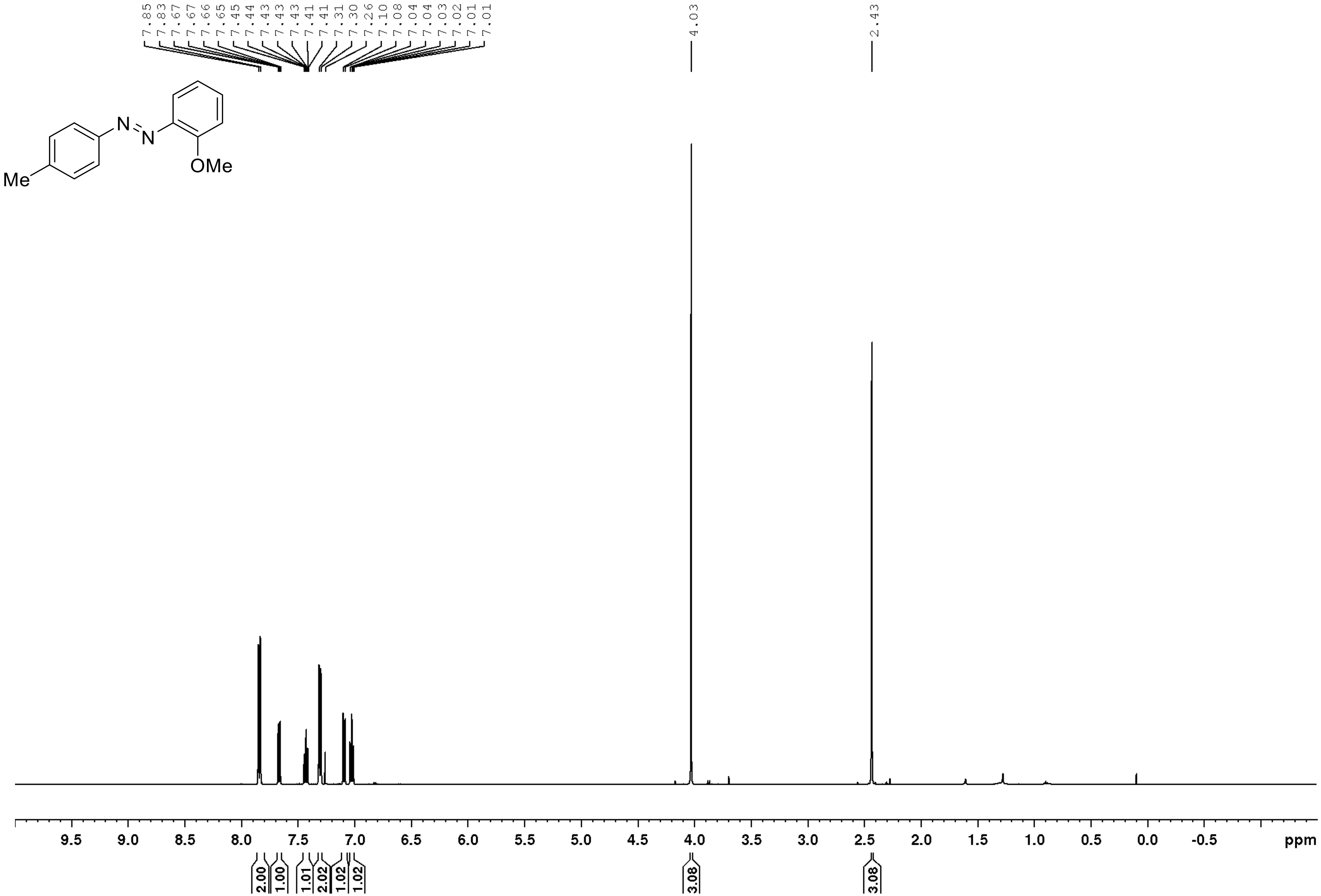

Figure S45. $^{13}C\{^{1}H\}$ NMR spectrum (126 MHz, $CDCl_3$) of (*E*)-1-(2-methoxyphenyl)-2-(*p*-tolyl)diazene (**6ad**).

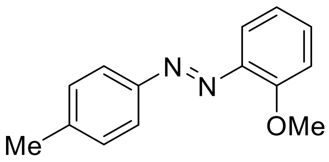

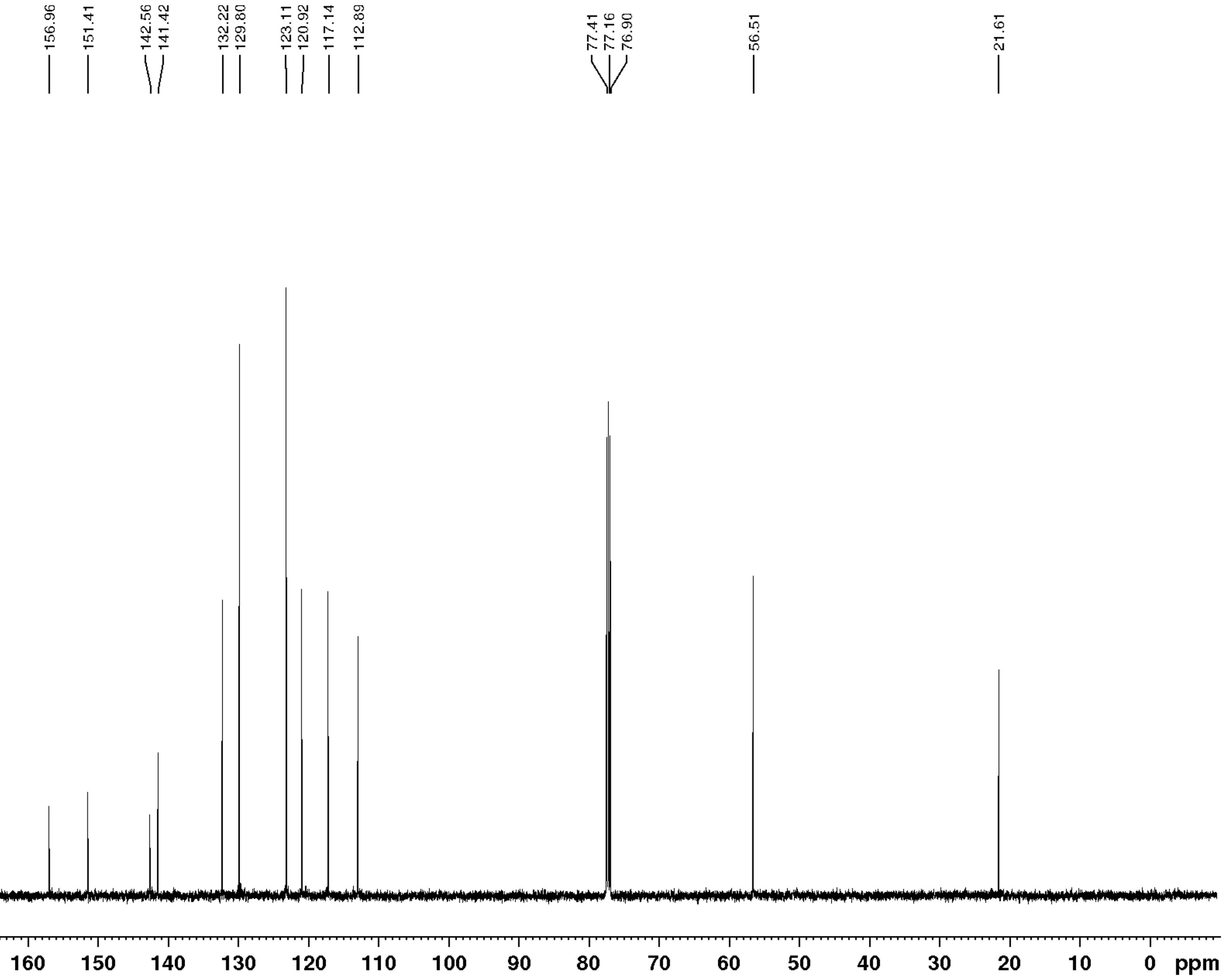

Figure S46. ^{1}H NMR spectrum (500 MHz, $CDCl_3$) of (*E*)-1-(3-methoxyphenyl)-2-(*p*-tolyl)diazene (**6aa**).

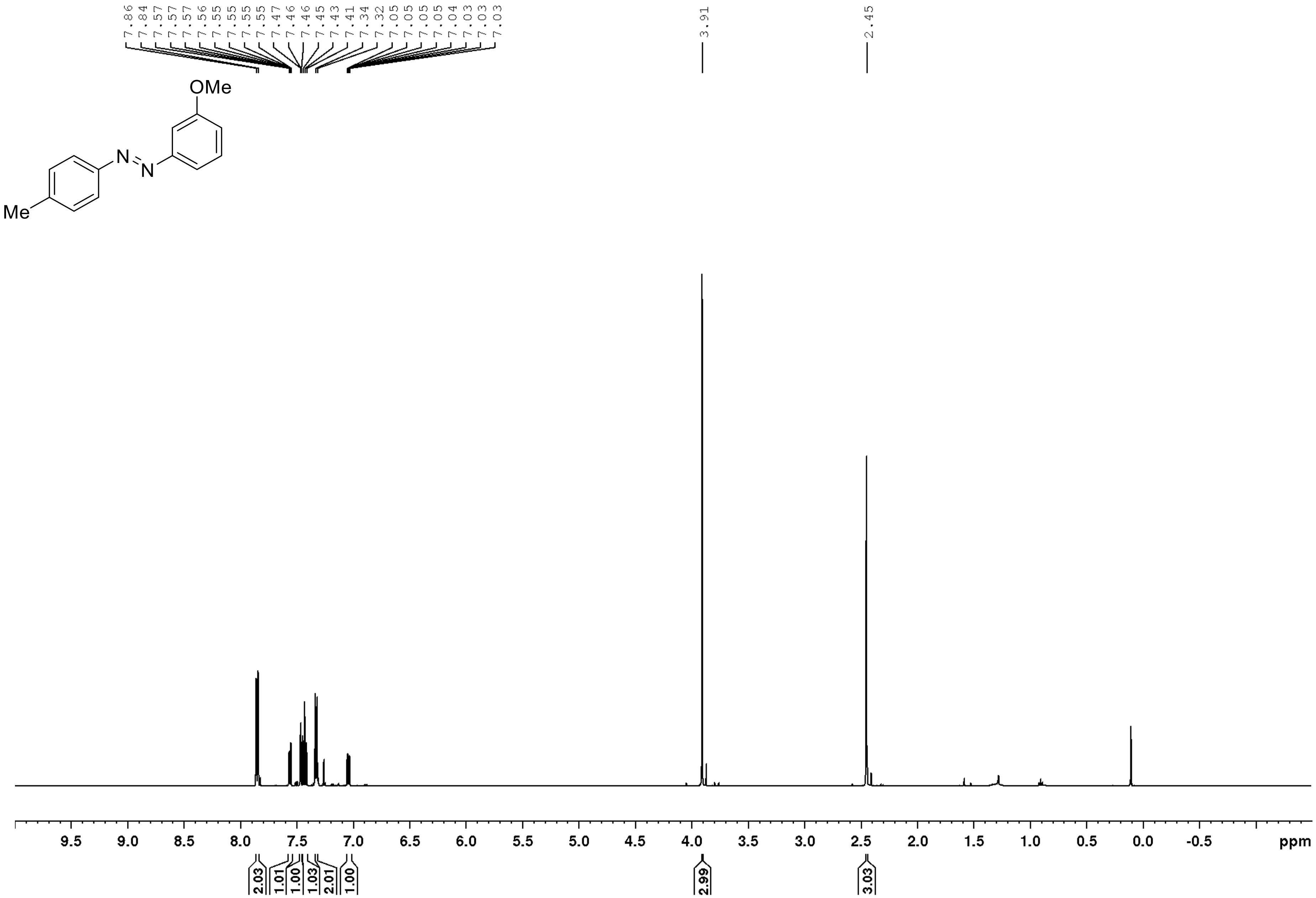

Figure S47. $^{13}C\{^1H\}$ NMR spectrum (126 MHz, $CDCl_3$) of (*E*)-1-(3-methoxyphenyl)-2-(*p*-tolyl)diazene (**6aa**).

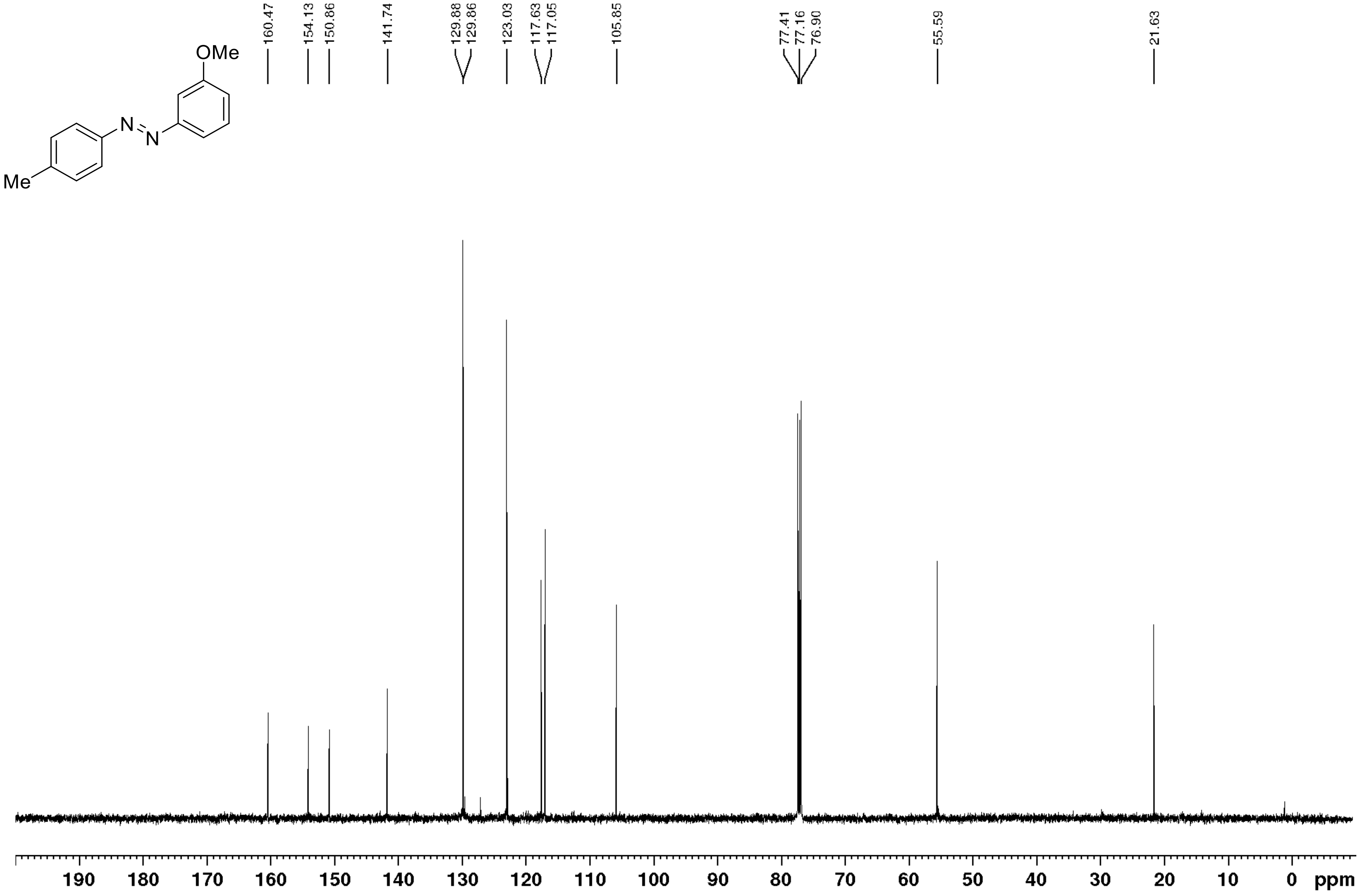

Figure S48. ^{1}H NMR spectrum (400 MHz, $CDCl_3$) of (*E*)-1-(4-methoxyphenyl)-2-(*p*-tolyl)diazene (**6ae**).

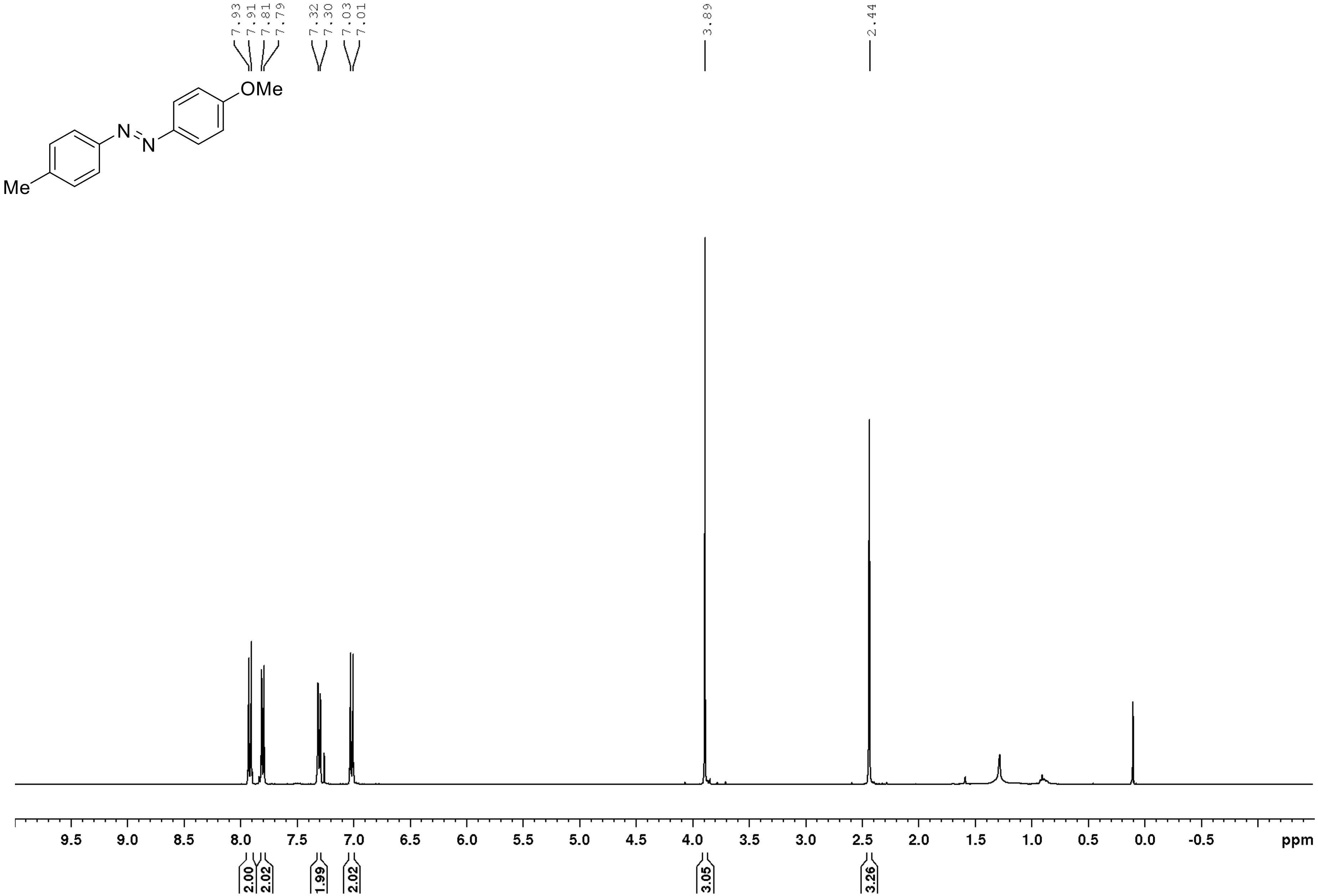

Figure S49. $^{13}C\{^1H\}$ NMR spectrum (101 MHz, $CDCl_3$) of (*E*)-1-(4-methoxyphenyl)-2-(*p*-tolyl)diazene (**6ae**).

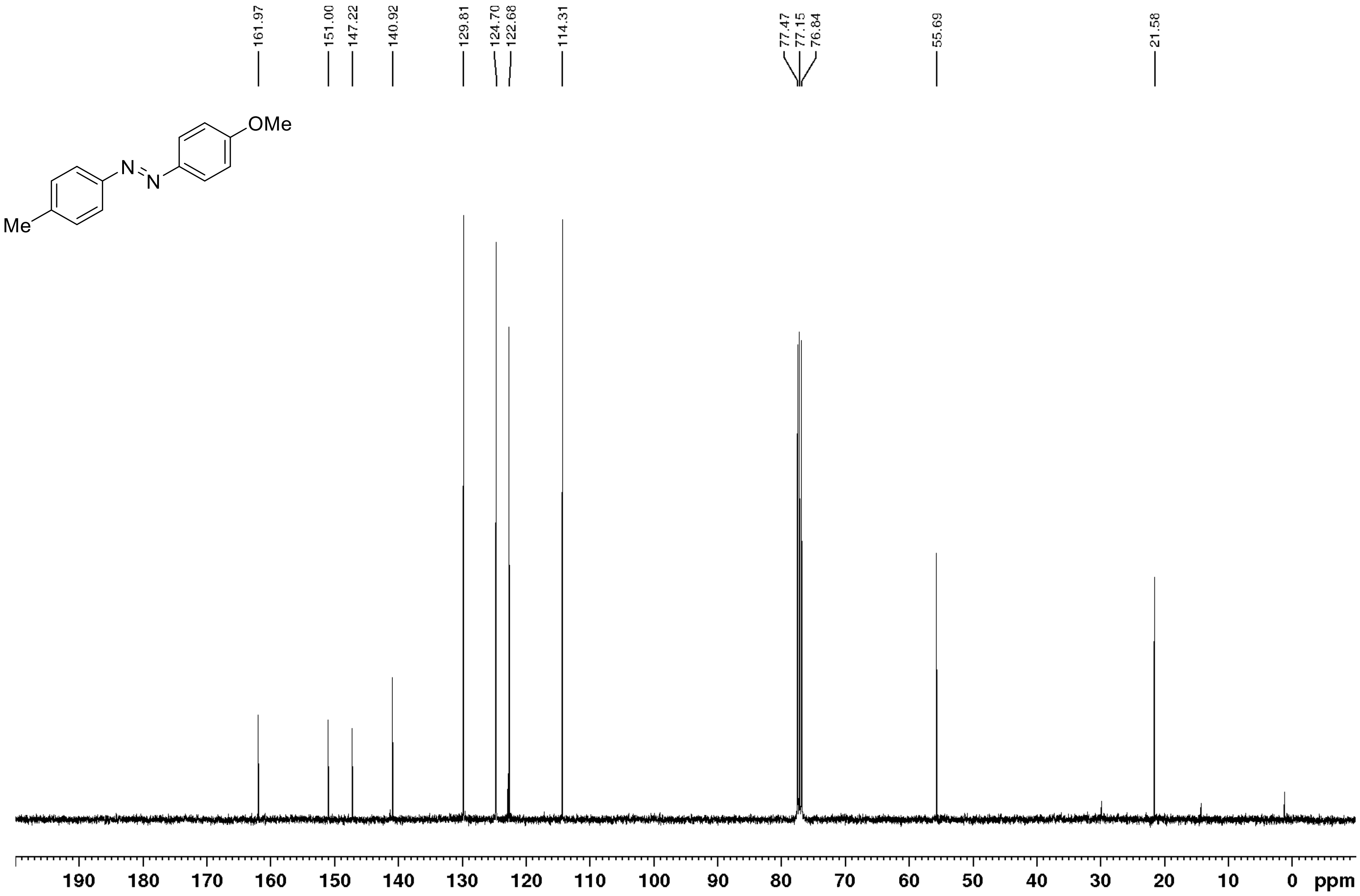

Figure S50. ^{1}H NMR spectrum (500 MHz, $CDCl_3$) of (*E*)-*N*,*N*-dimethyl-4-(*p*-tolyldiazenyl)aniline (**6af**).

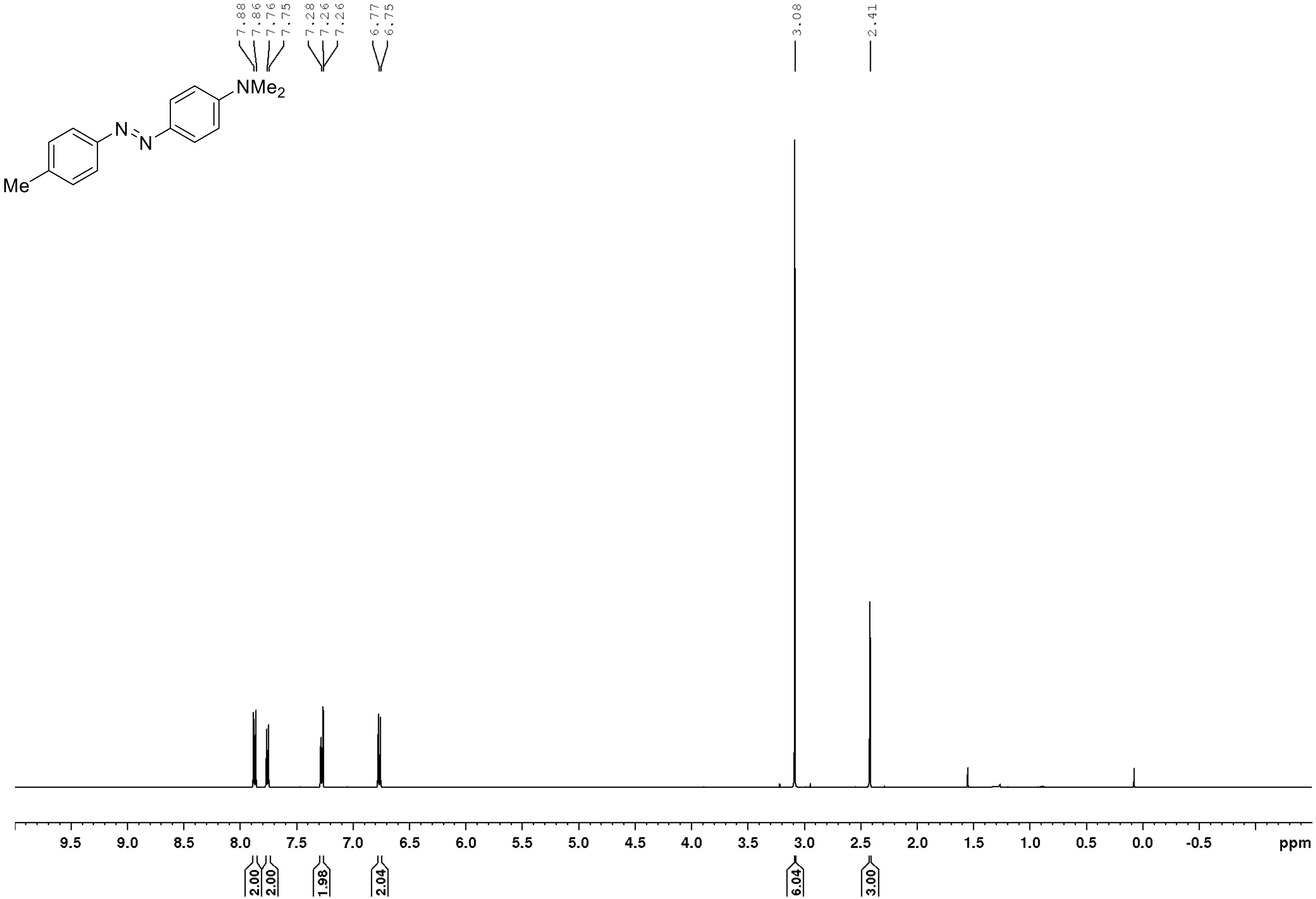

Figure S51. $^{13}C\{^{1}H\}$ NMR spectrum (126 MHz, $CDCl_3$) of (*E*)-*N*,*N*-dimethyl-4-(*p*-tolyldiazenyl)aniline (**6af**).

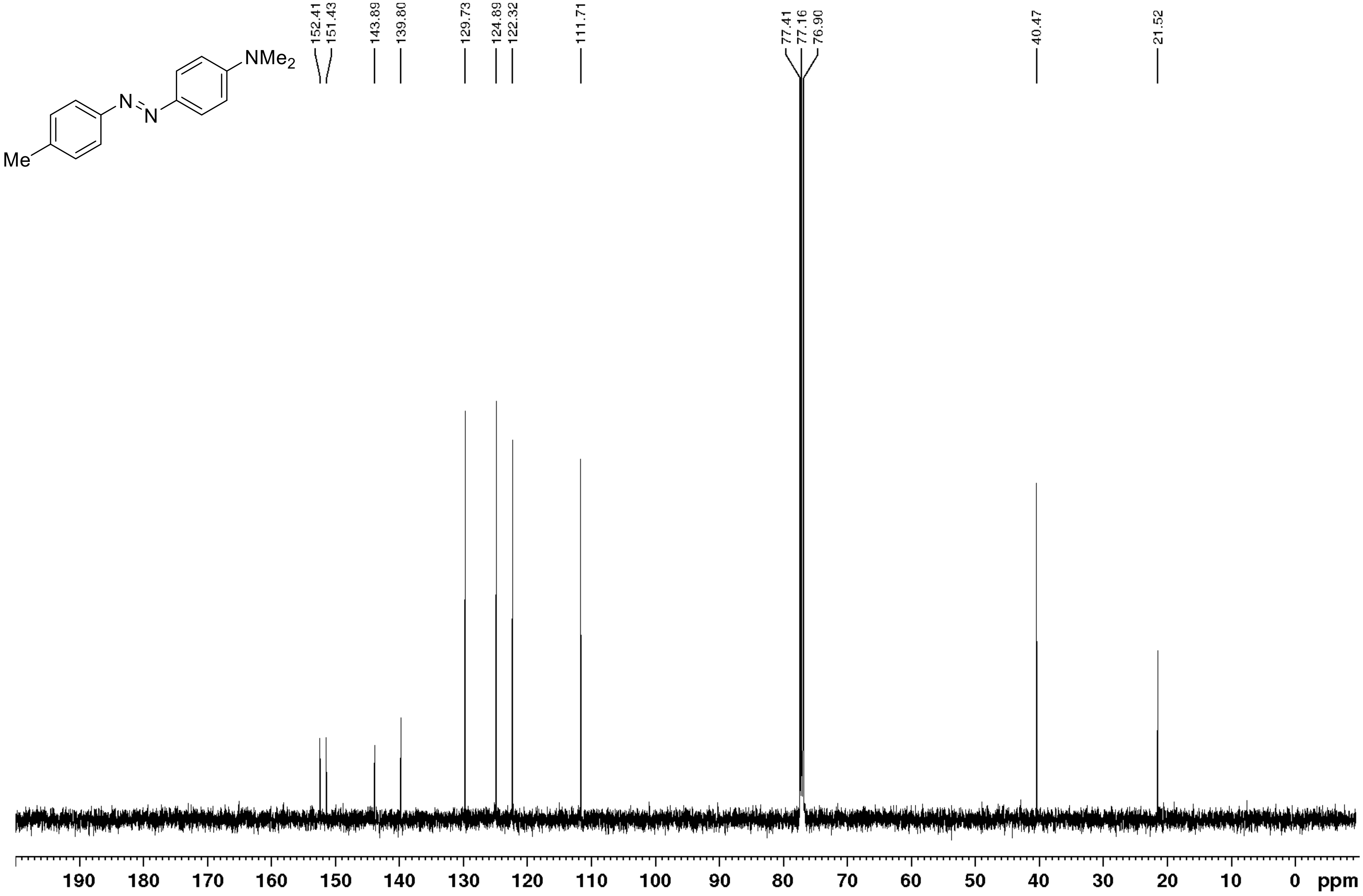

Figure S52. ^{1}H NMR spectrum (400 MHz, $CDCl_3$) of (*E*)-1-phenyl-2-(*p*-tolyl)diazene (**6ag**).

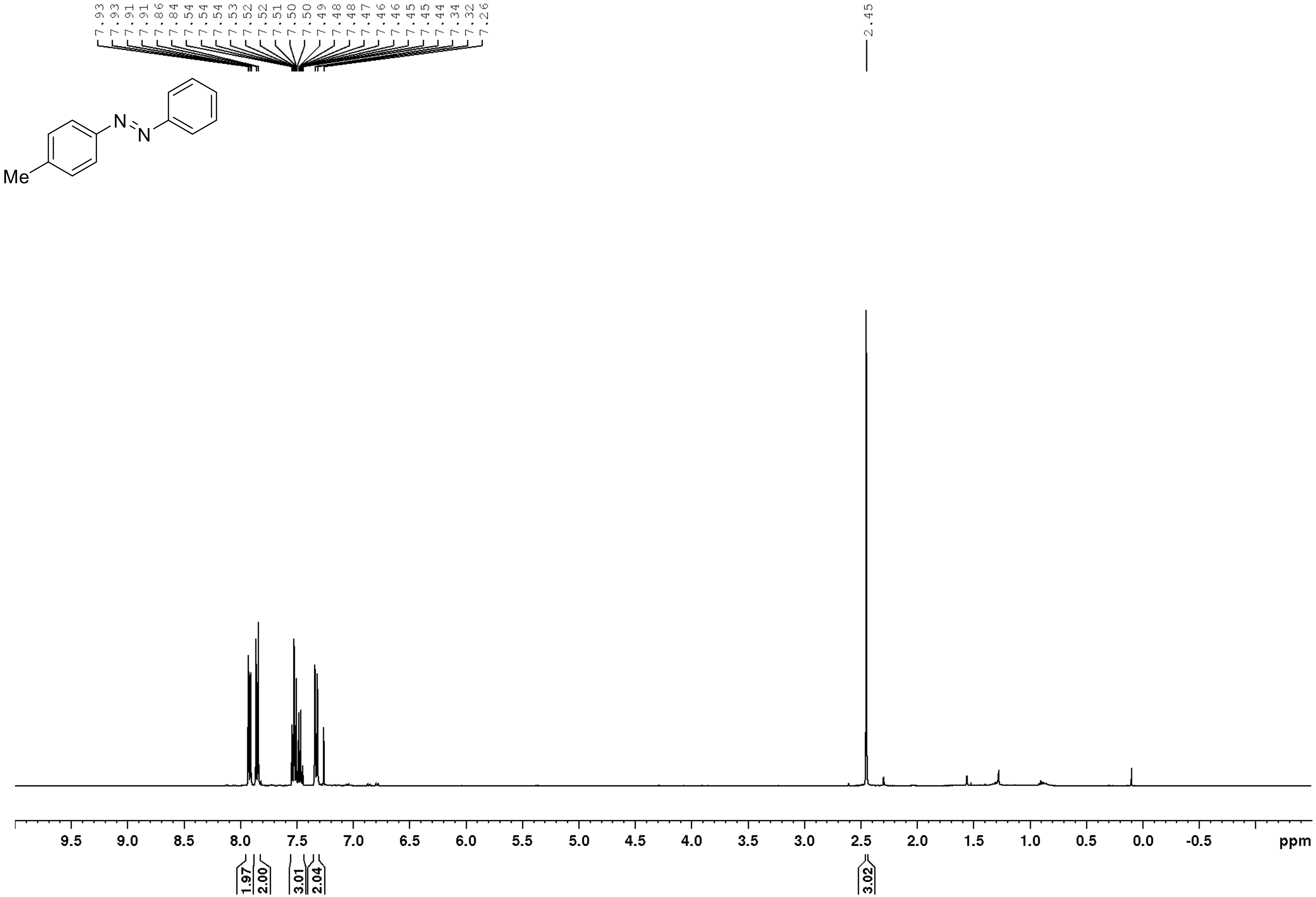

Figure S53. $^{13}C\{^{1}H\}$ NMR spectrum (101 MHz, $CDCl_3$) of (*E*)-1-phenyl-2-(*p*-tolyl)diazene (**6ag**).

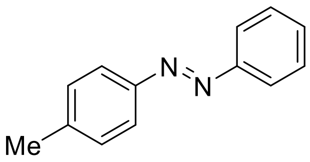

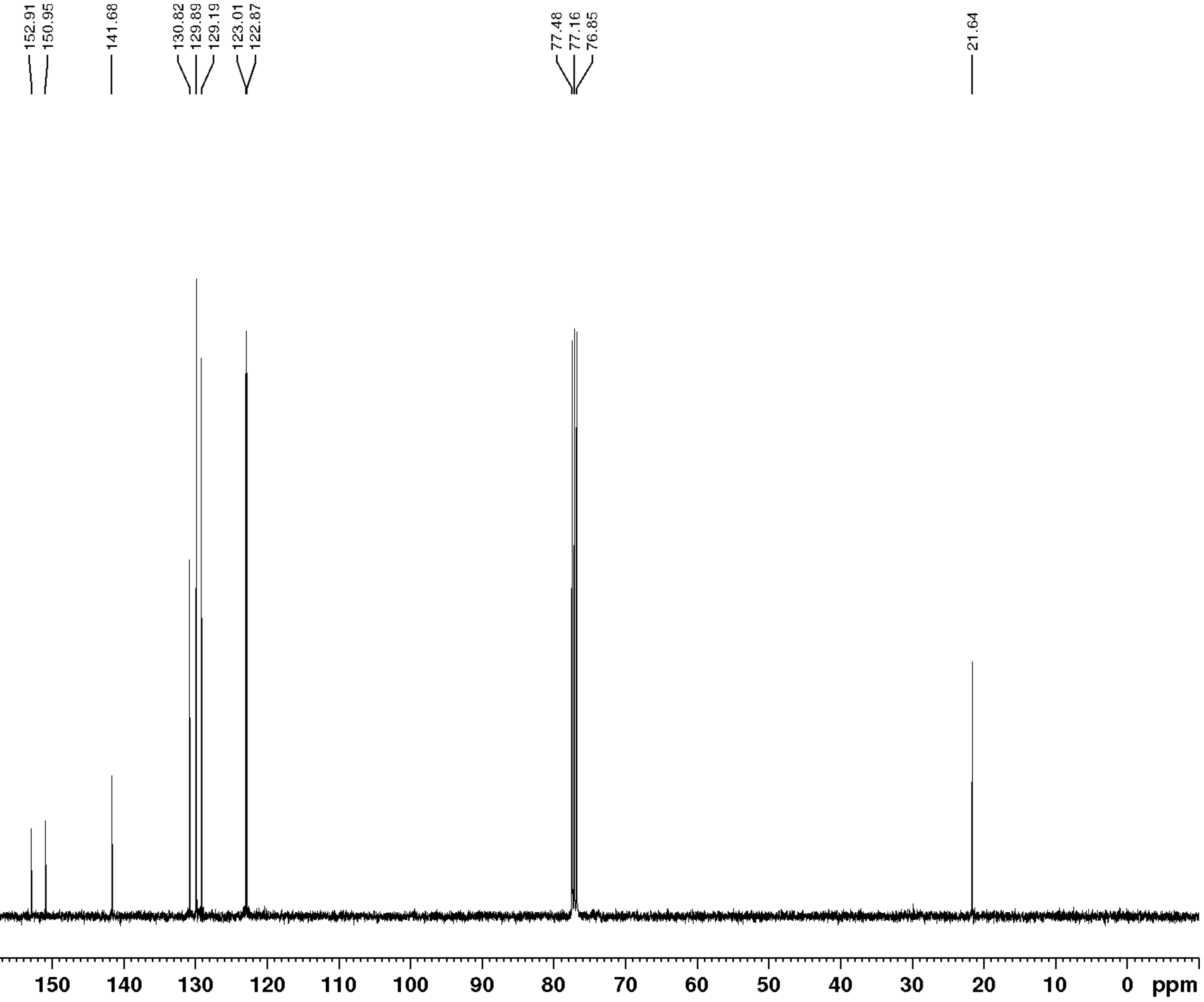

Figure S54. ^{1}H NMR spectrum (500 MHz, $CDCl_3$) of (*E*)-1-(2,6-dimethylphenyl)-2-(*p*-tolyl)diazene (**6ah**).

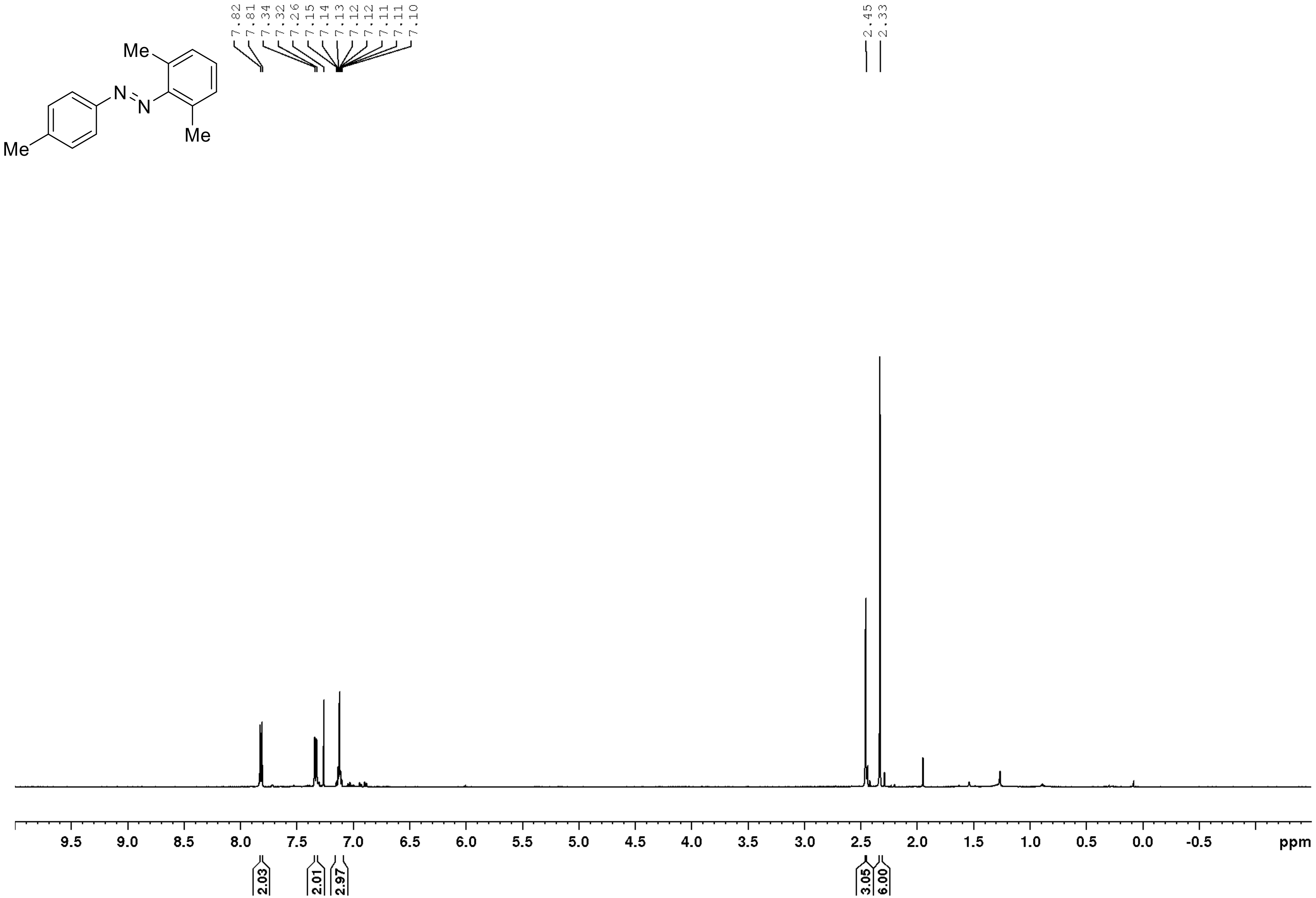

Figure S55. $^{13}C\{^1H\}$ NMR spectrum (101 MHz, $CDCl_3$) of (*E*)-1-(2,6-dimethylphenyl)-2-(*p*-tolyl)diazene (**6ah**).

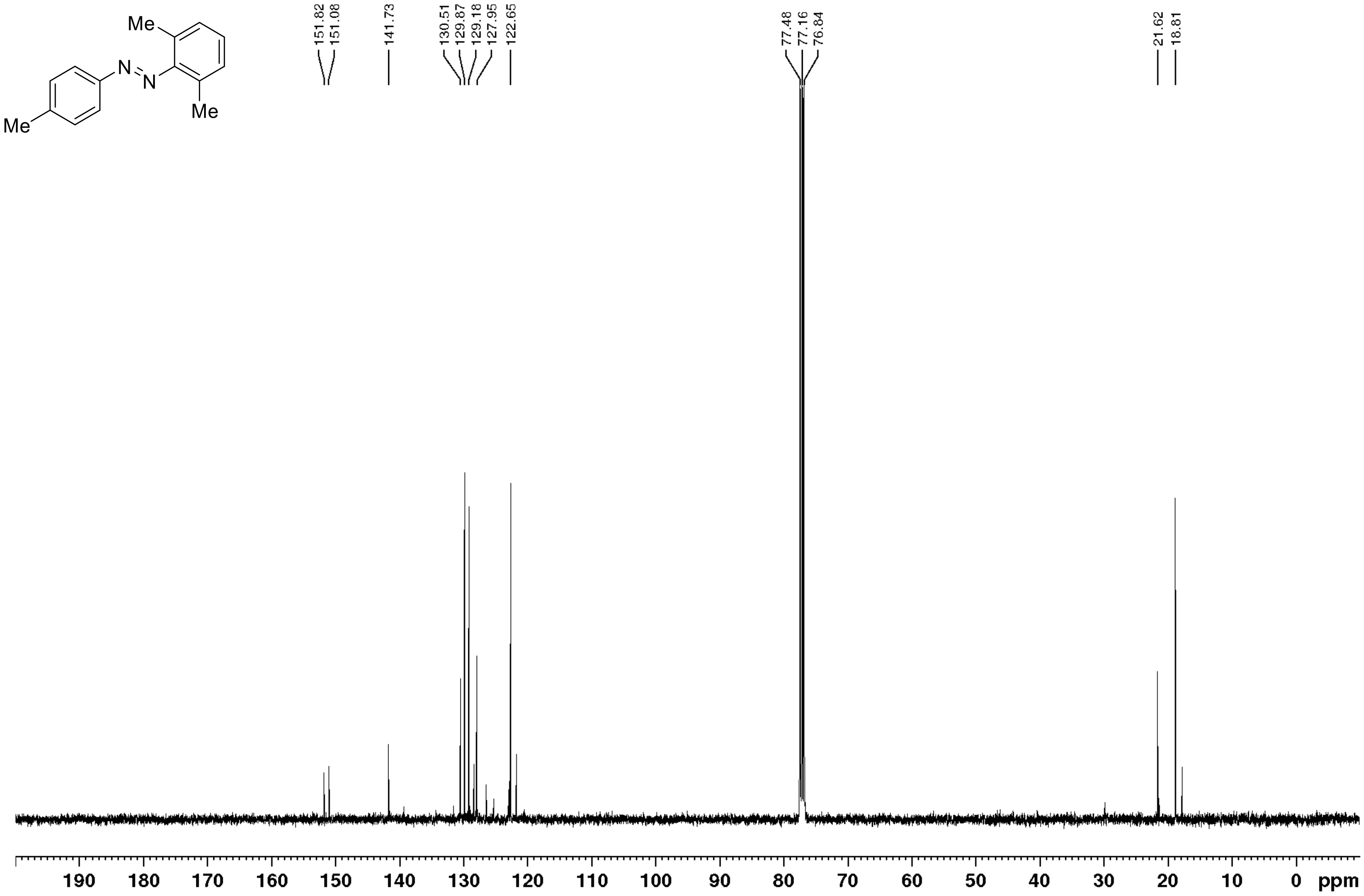

Figure S56. ^{1}H NMR spectrum (500 MHz, $CDCl_3$) of methyl (*E*)-4-((3,5-dimethoxyphenyl)diazenyl)benzoate (**6hi**).

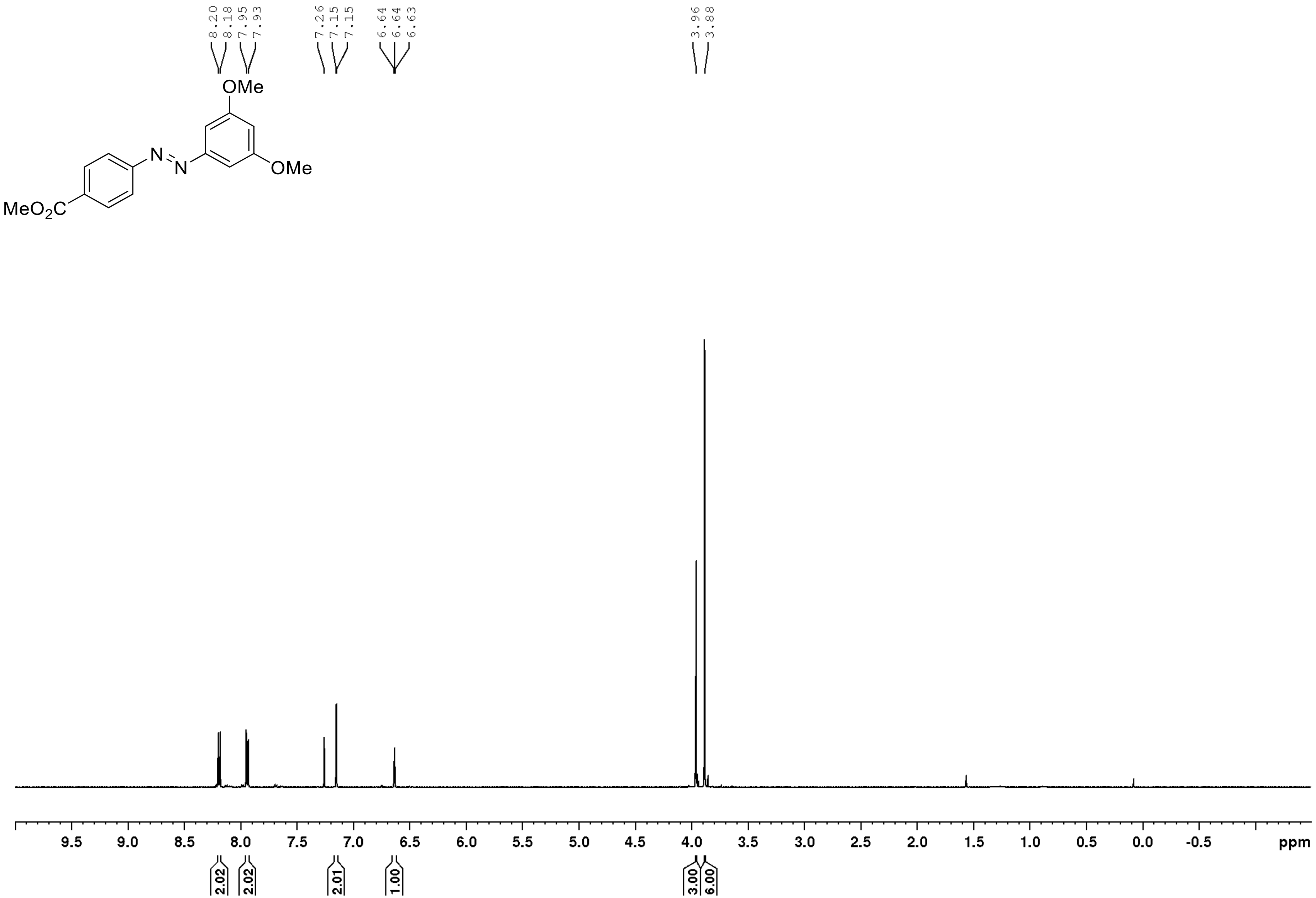

Figure S57. $^{13}C\{^1H\}$ NMR spectrum (126 MHz, $CDCl_3$) of methyl (*E*)-4-((3,5-dimethoxyphenyl)diazenyl)benzoate (**6hi**).

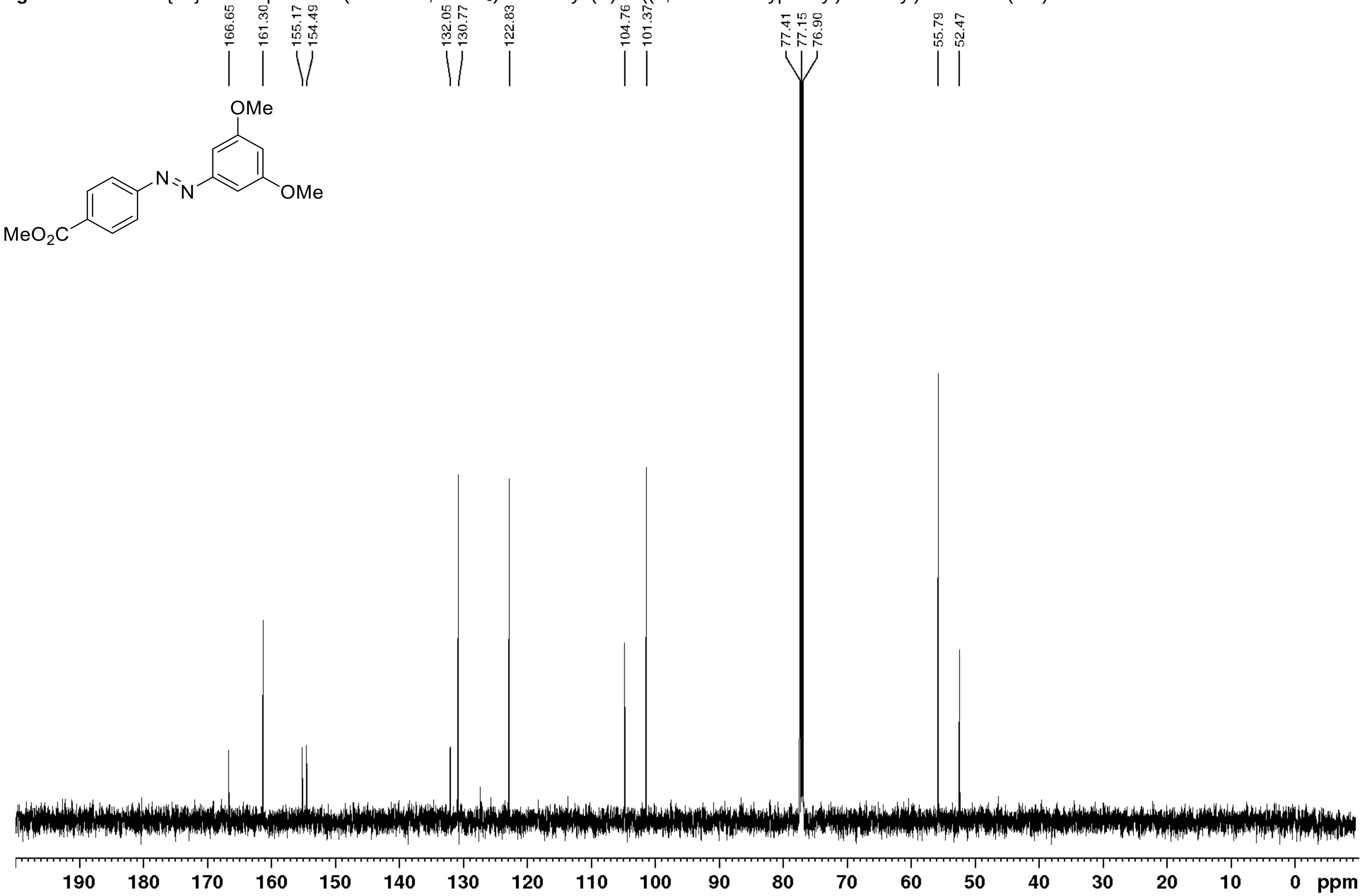

Figure S58. ^{1}H NMR spectrum (400 MHz, $CDCl_3$) of (*E*)-1-(benzo[*d*][1,3]dioxol-5-yl)-2-(*p*-tolyl)diazene (**6aj**).

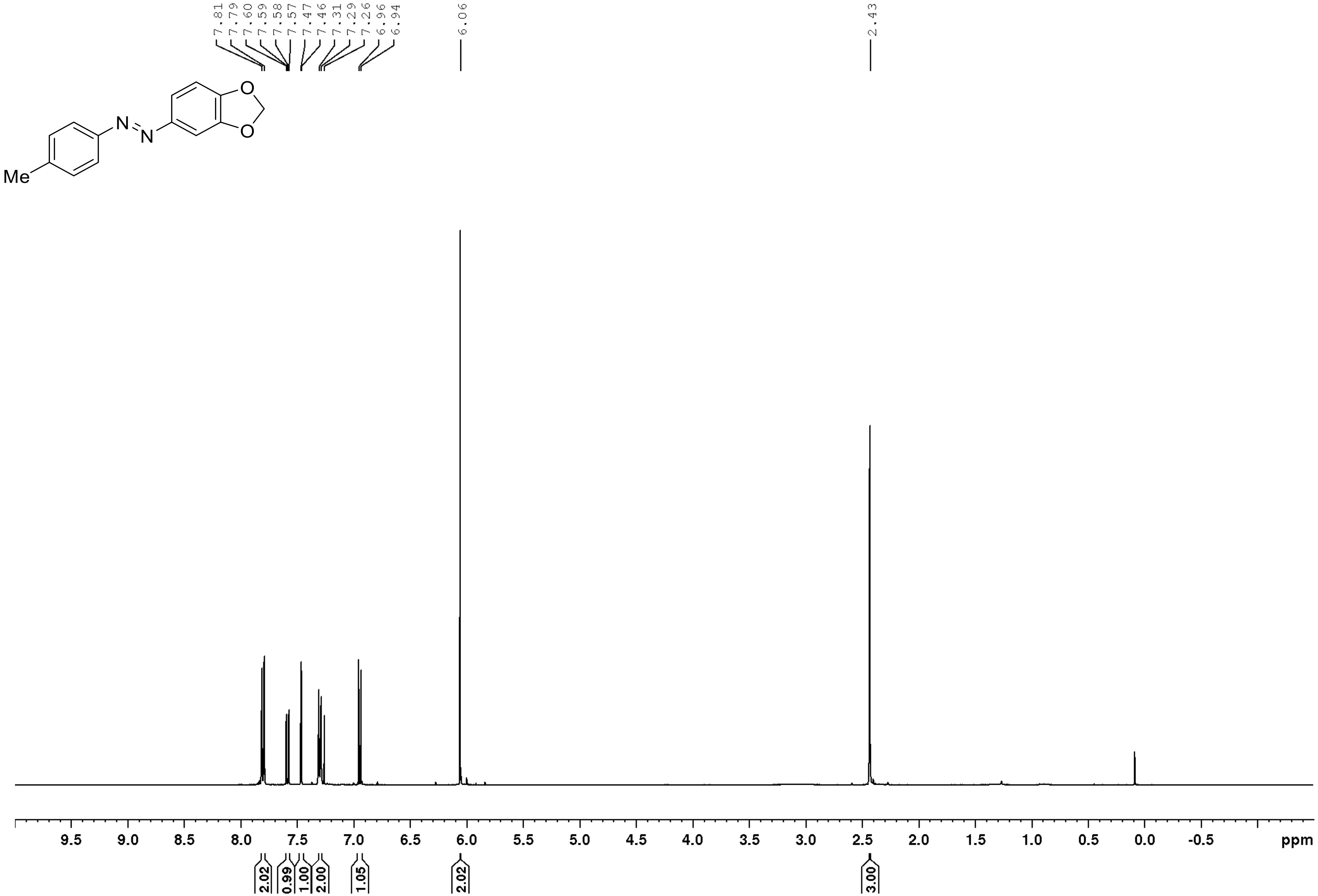

Figure S59. $^{13}C\{^1H\}$ NMR spectrum (101 MHz, $CDCl_3$) of (*E*)-1-(benzo[*d*][1,3]dioxol-5-yl)-2-(*p*-tolyl)diazene (**6aj**).

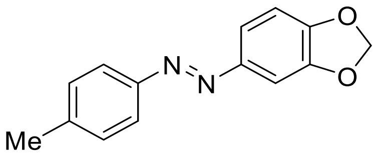

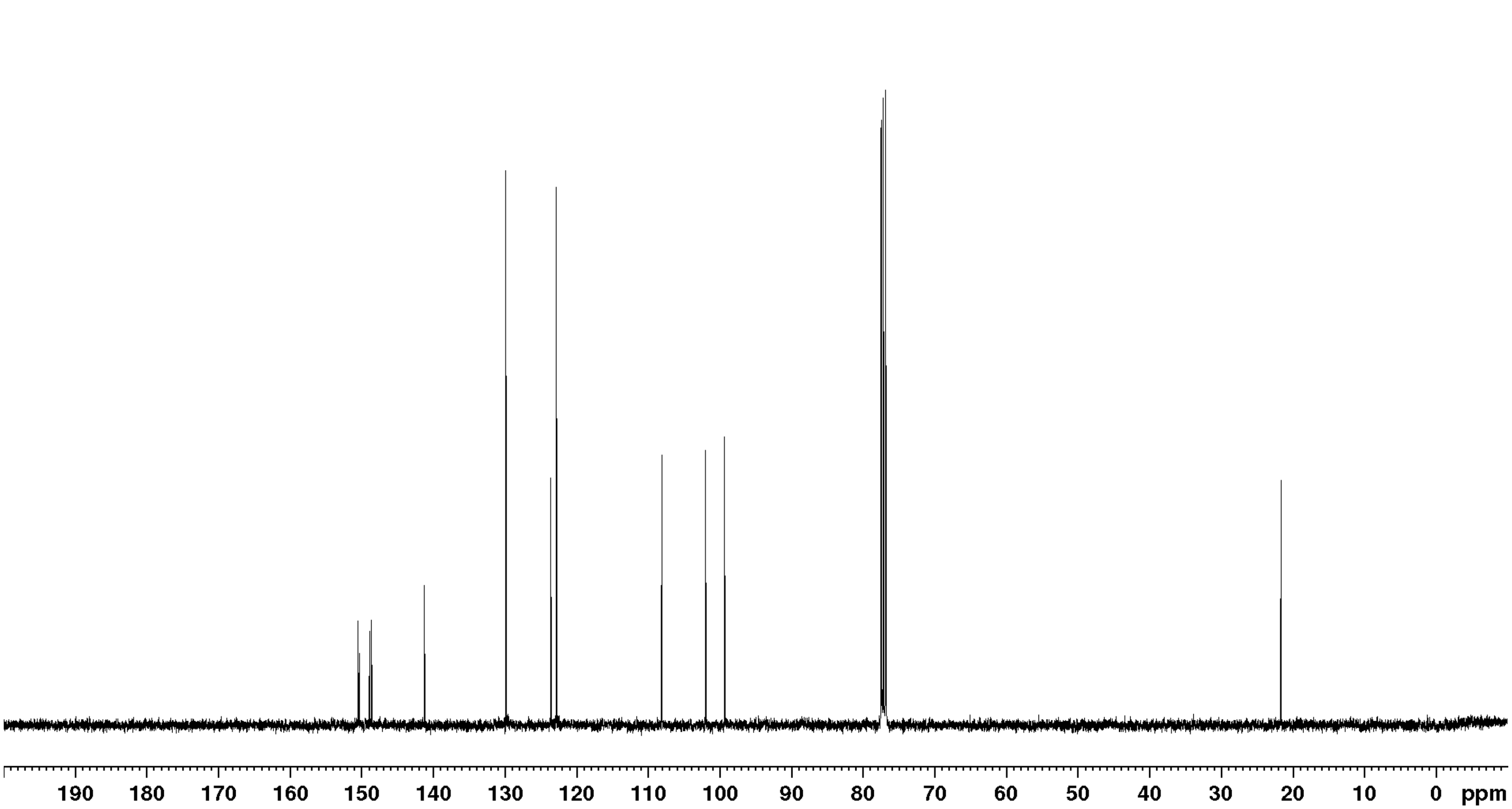

Figure S60. ^1H NMR spectrum (500 MHz, $CDCl_3$) of (*E*)-1-(benzo[*d*][1,3]dioxol-5-yl)-2-(4-chlorophenyl)diazene (**6ej**).

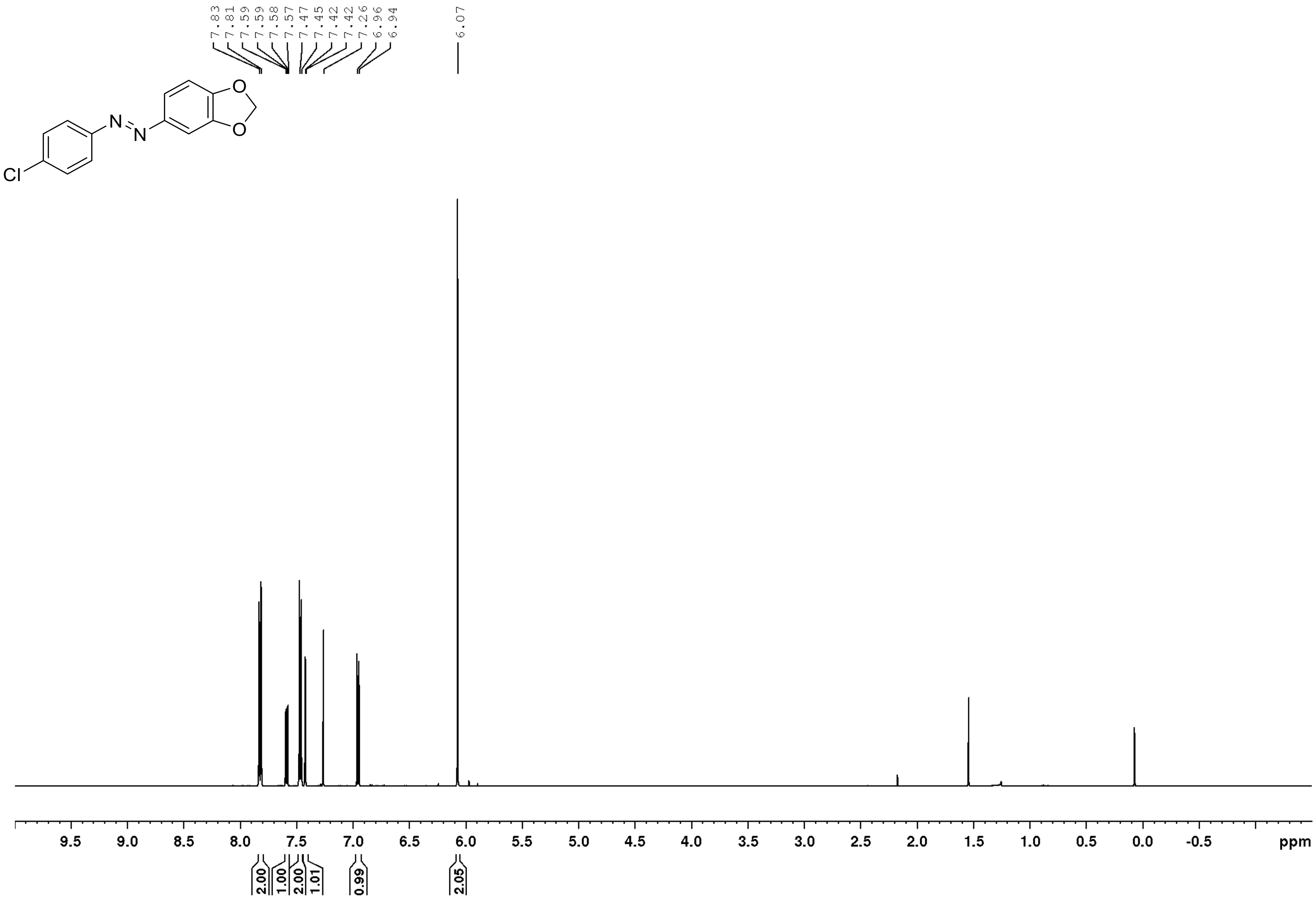

Figure S61. $^{13}C\{^{1}H\}$ NMR spectrum (126 MHz, $CDCl_3$) of (*E*)-1-(benzo[*d*][1,3]dioxol-5-yl)-2-(4-chlorophenyl)diazene (**6ej**).

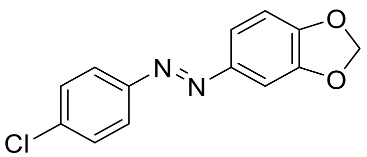

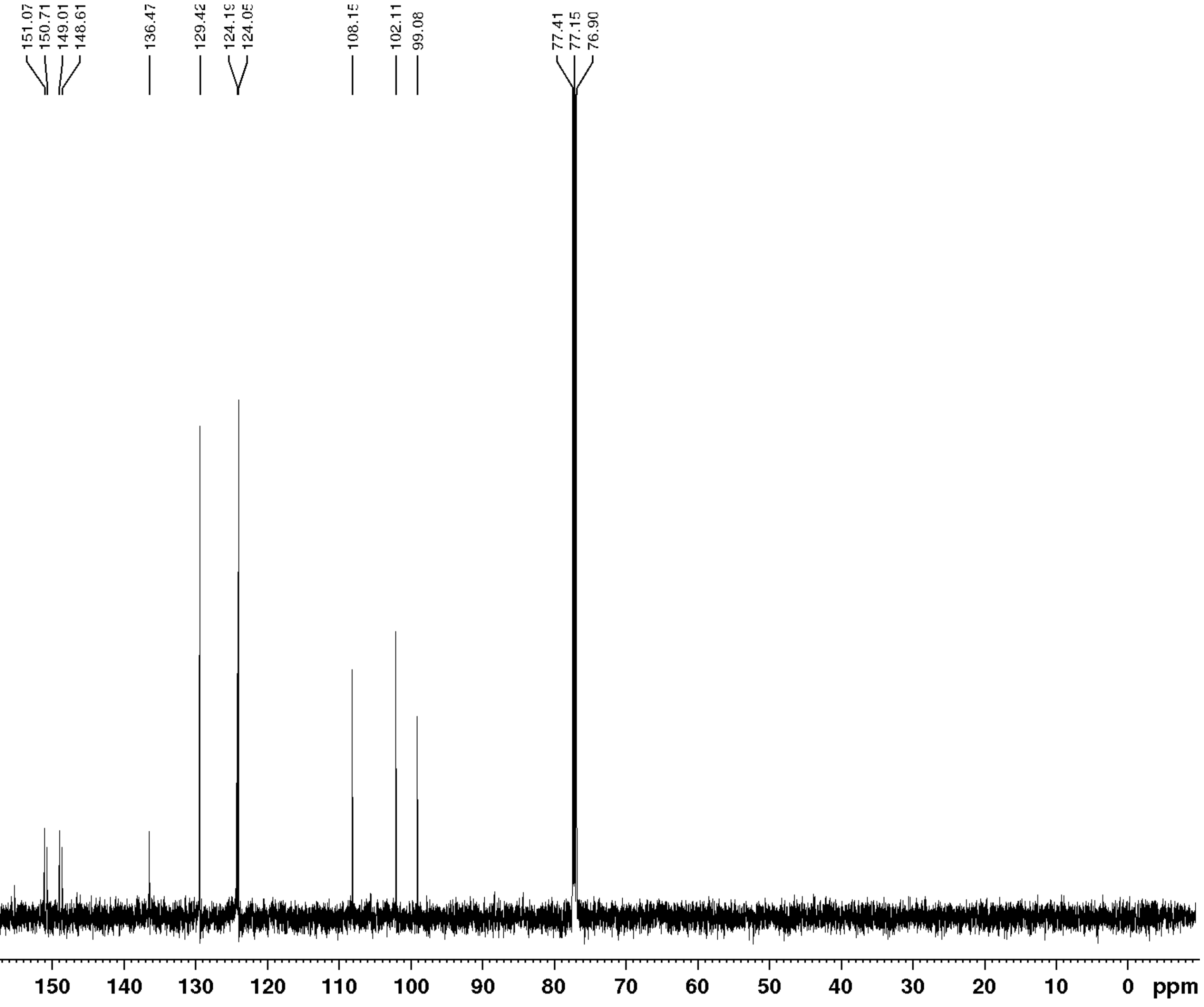

Figure S62. ^{1}H NMR spectrum (500 MHz, $CDCl_3$) of (*E*)-1-(4-fluorophenyl)-2-(4-methoxyphenyl)diazene (**6bk**).

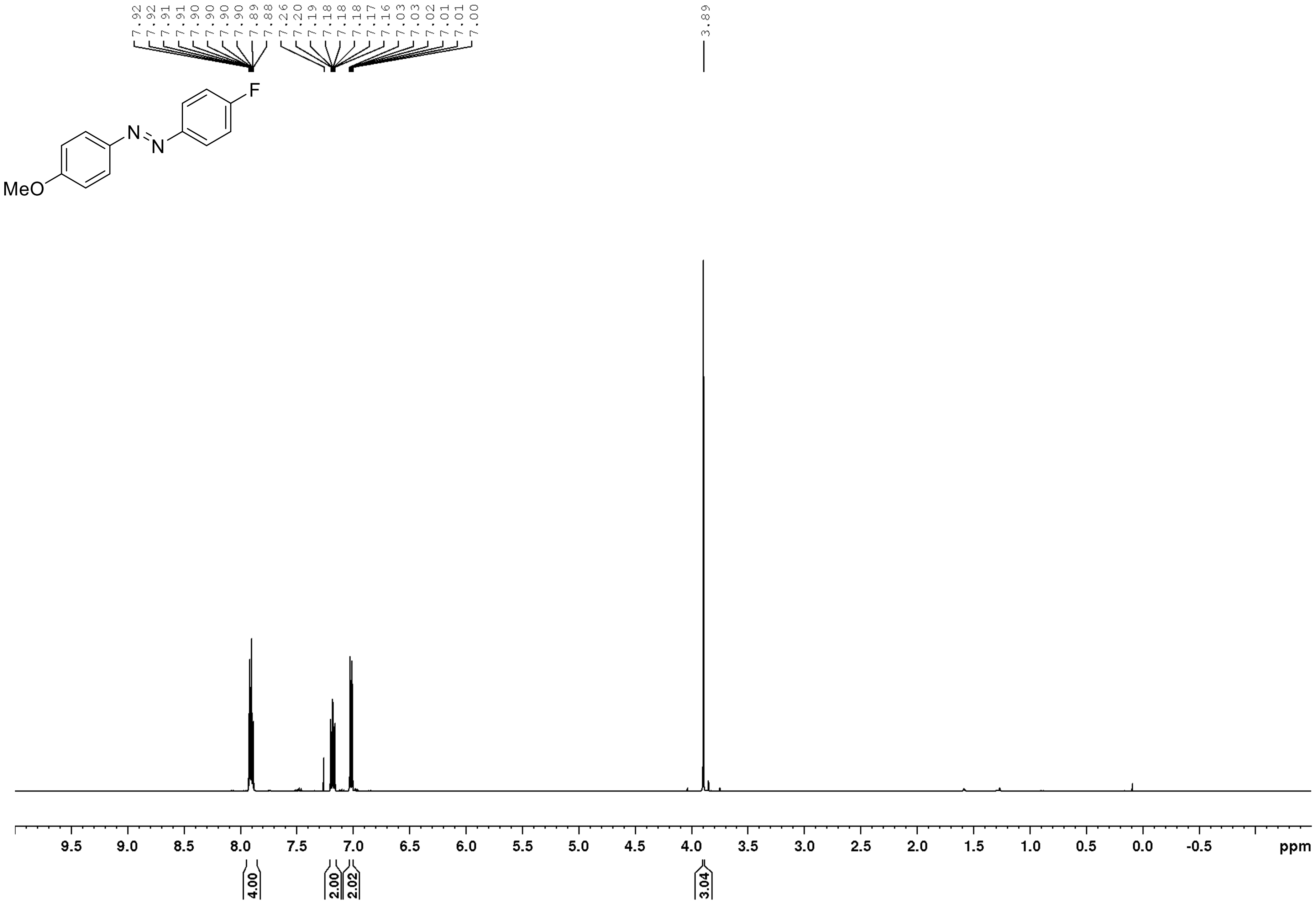

Figure S63. $^{13}C\{^{1}H\}$ NMR spectrum (126 MHz, $CDCl_3$) of (*E*)-1-(4-fluorophenyl)-2-(4-methoxyphenyl)diazene (**6bk**).

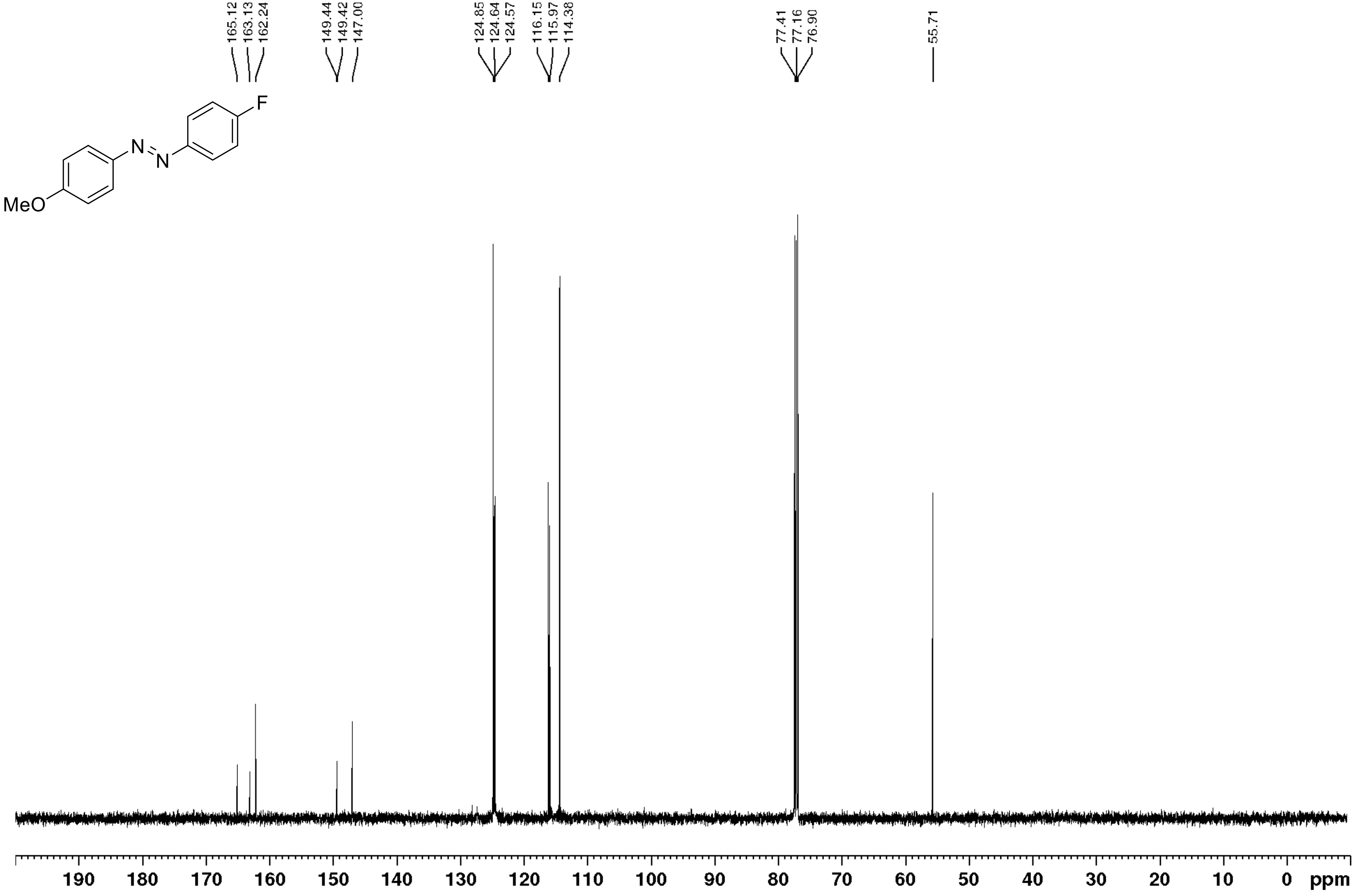

Figure S64. ^{19}F NMR spectrum (471 MHz, $CDCl_3$) of (*E*)-1-(4-fluorophenyl)-2-(4-methoxyphenyl)diazene (**6bk**).

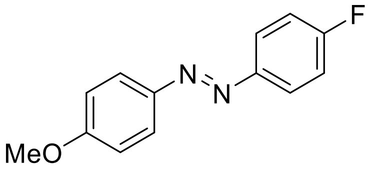

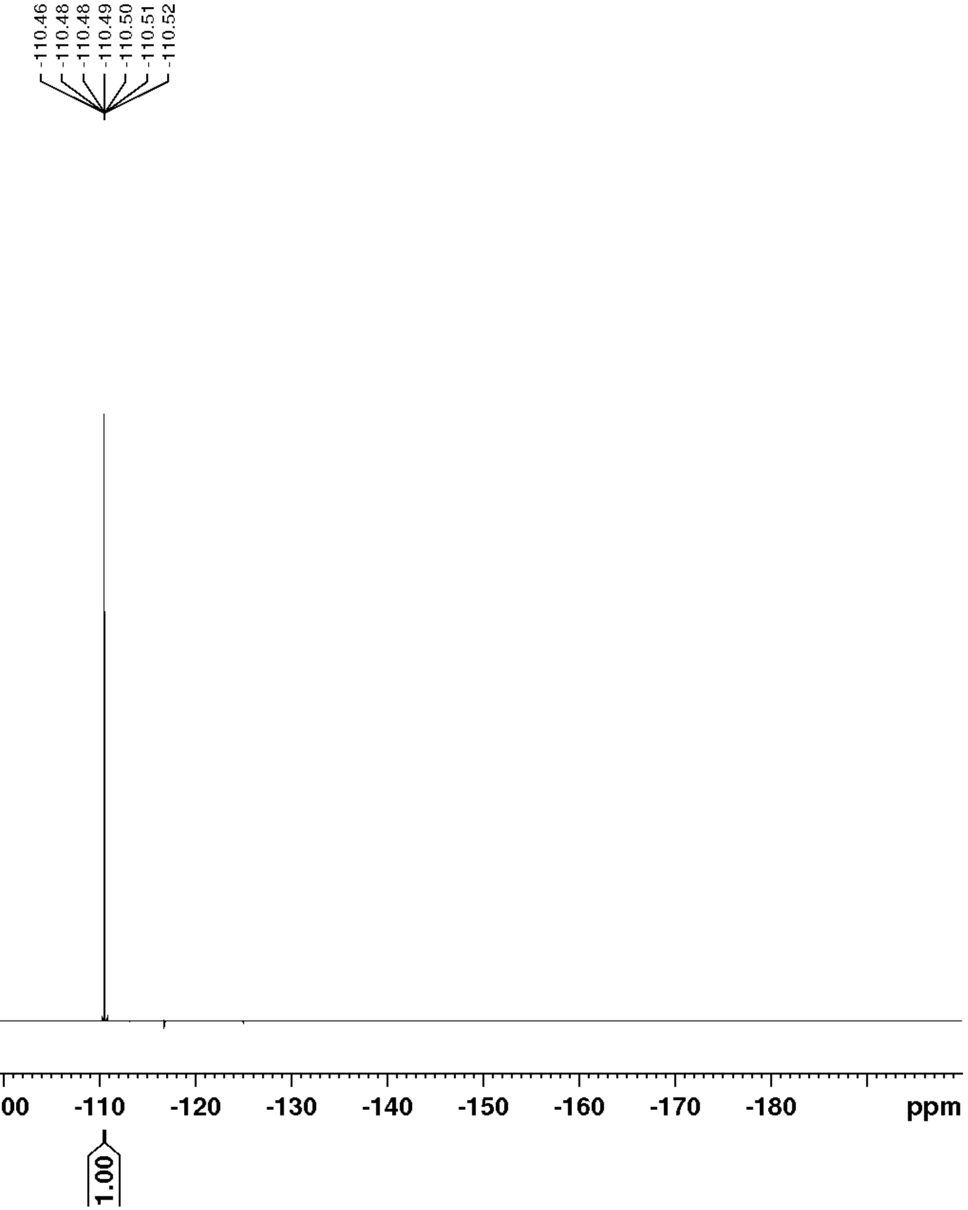

Figure S65. ^{1}H NMR spectrum (400 MHz, $CDCl_3$) of (*E*)-1-(4-chlorophenyl)-2-(4-methoxyphenyl)diazene (**6bl**).

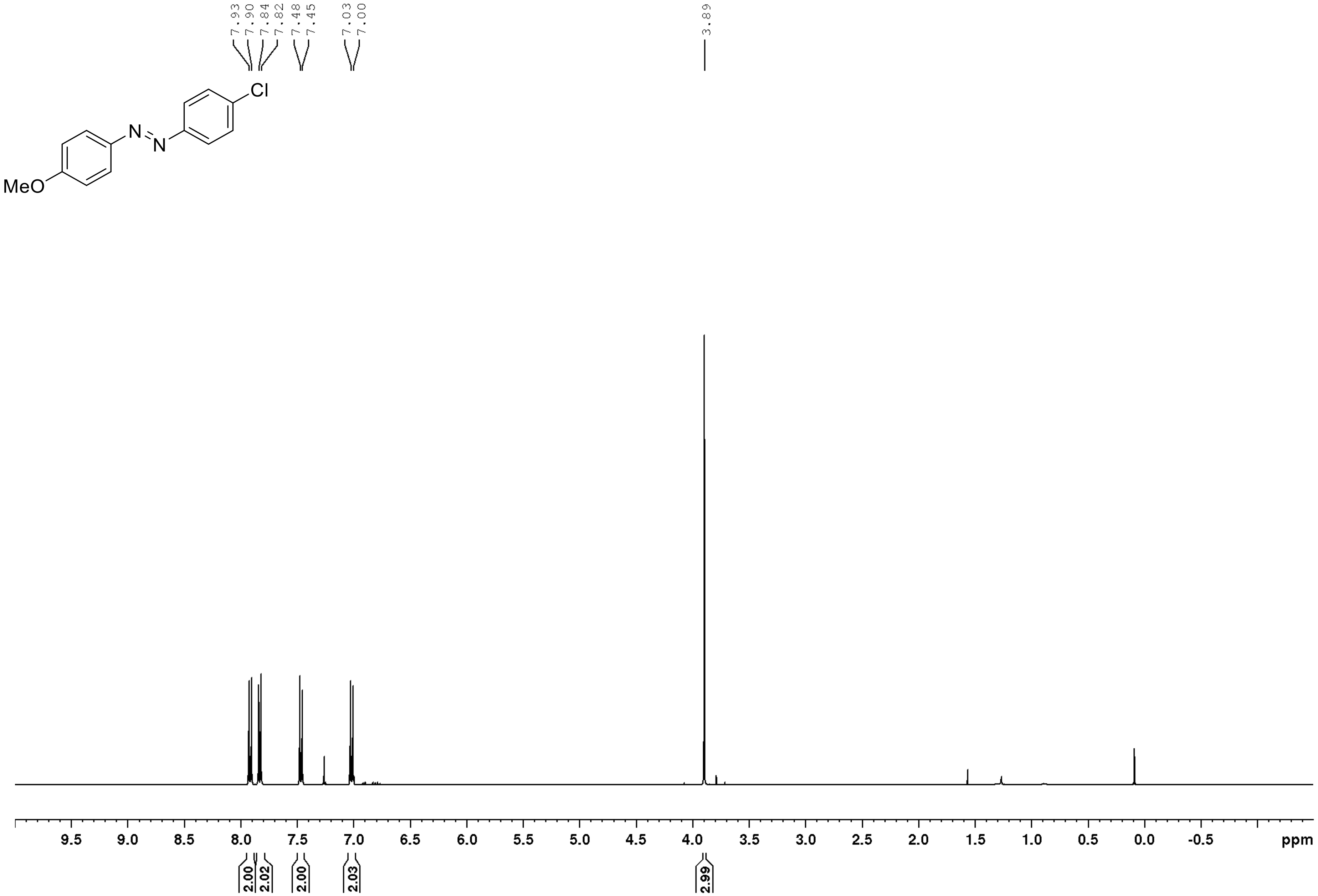

Figure S66. $^{13}C\{^1H\}$ NMR spectrum (101 MHz, $CDCl_3$) of (*E*)-1-(4-chlorophenyl)-2-(4-methoxyphenyl)diazene (**6bl**).

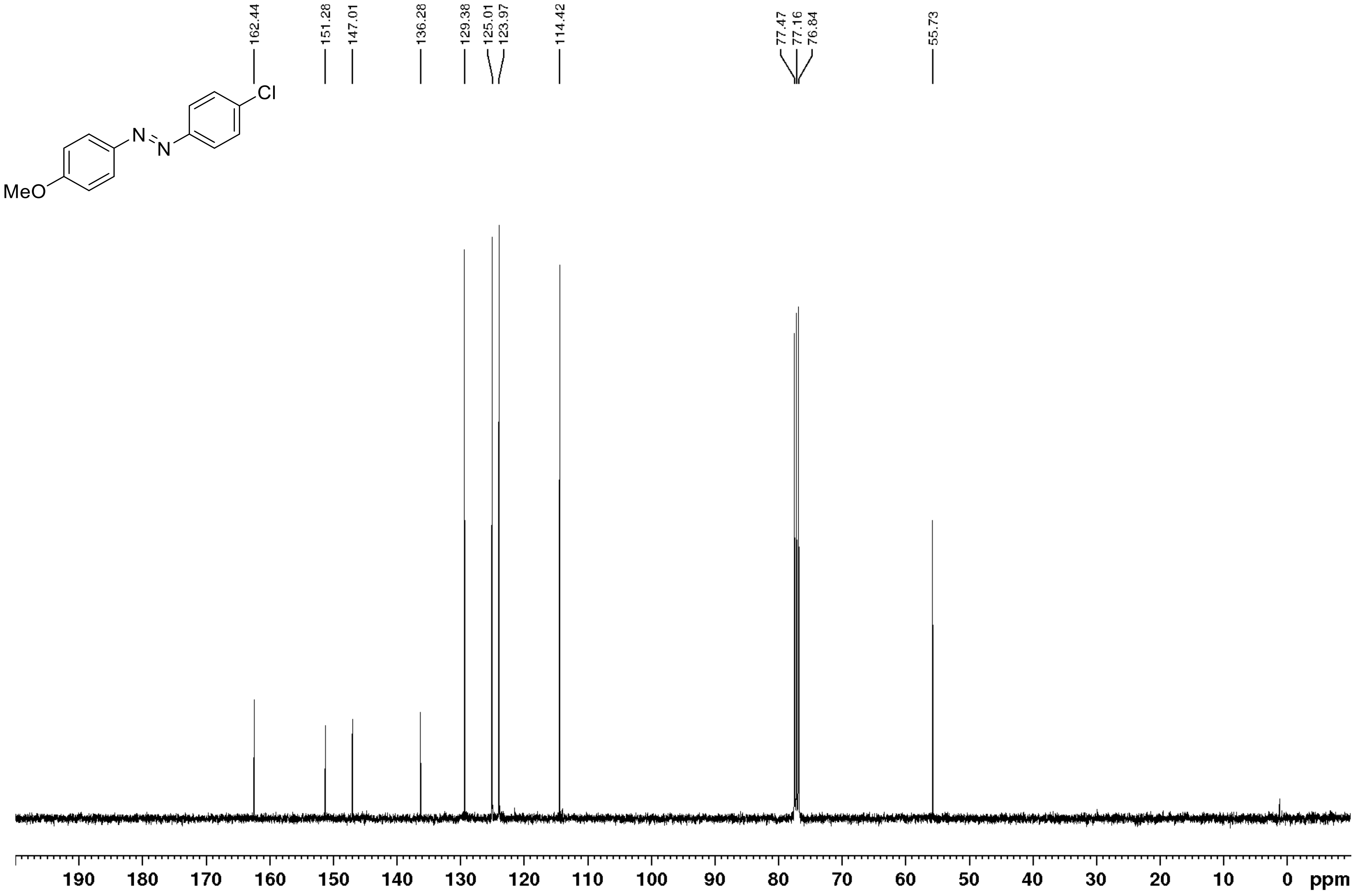

Figure S67. ^{1}H NMR spectrum (500 MHz, $CDCl_3$) of (*E*)-1-(4-bromophenyl)-2-(4-methoxyphenyl)diazene (**6bm**).

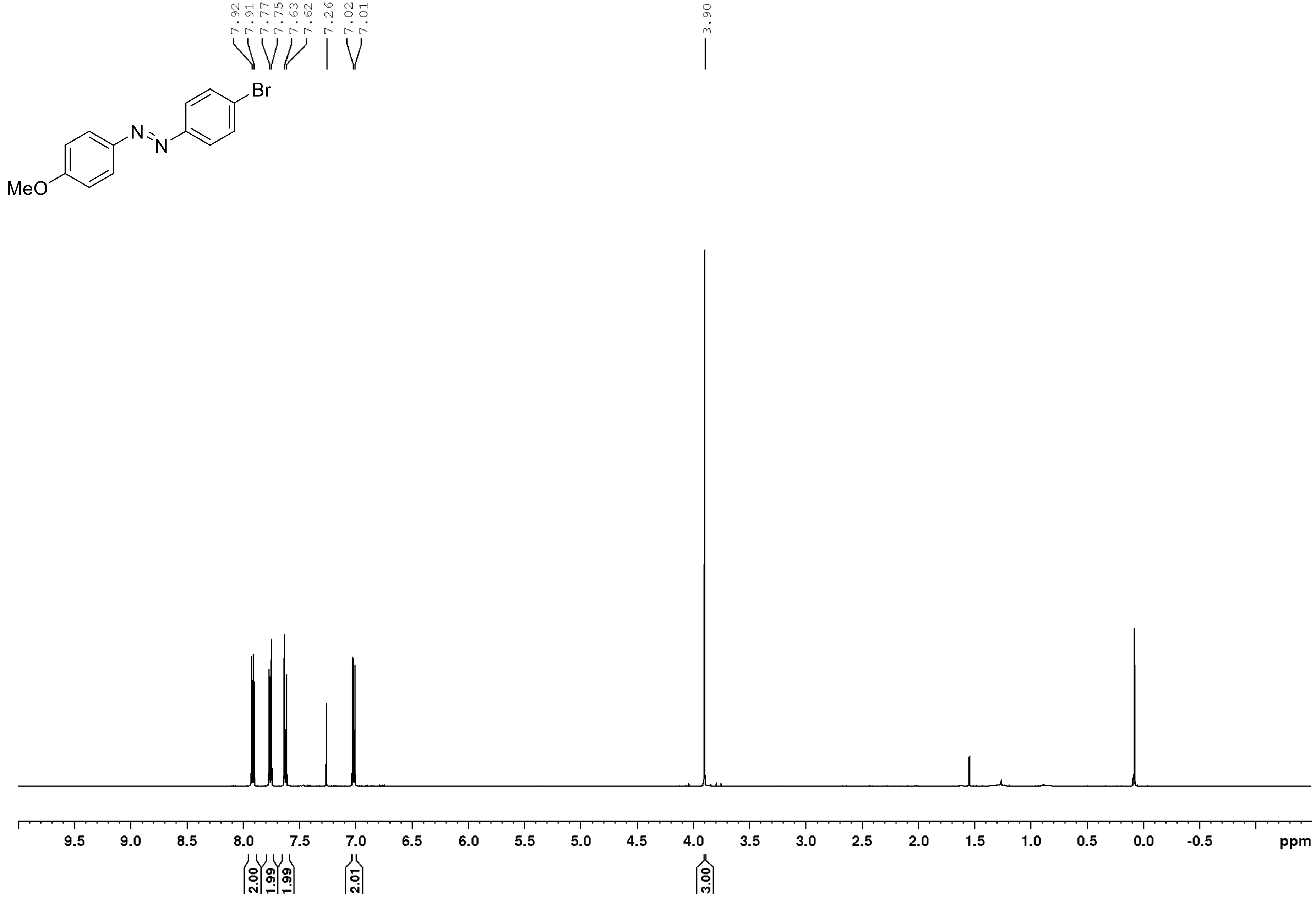

Figure S68. $^{13}C\{^1H\}$ NMR spectrum (126 MHz, $CDCl_3$) of (*E*)-1-(4-bromophenyl)-2-(4-methoxyphenyl)diazene (**6bm**).

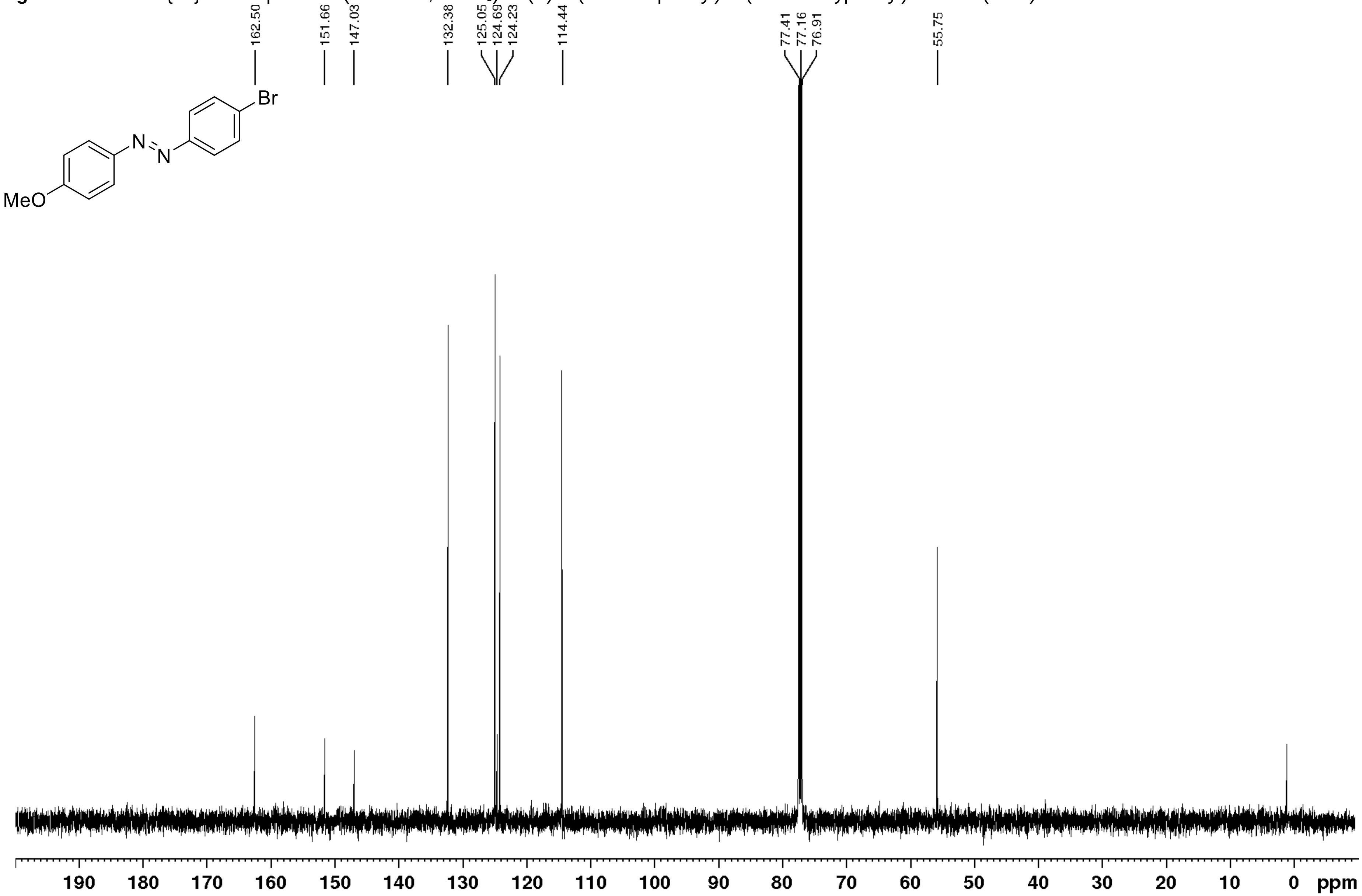

Figure S69. ^{1}H NMR spectrum (400 MHz, $CDCl_3$) of (*E*)-1-(4-methoxyphenyl)-2-(4-(trifluoromethyl)phenyl)diazene (**6bn**).

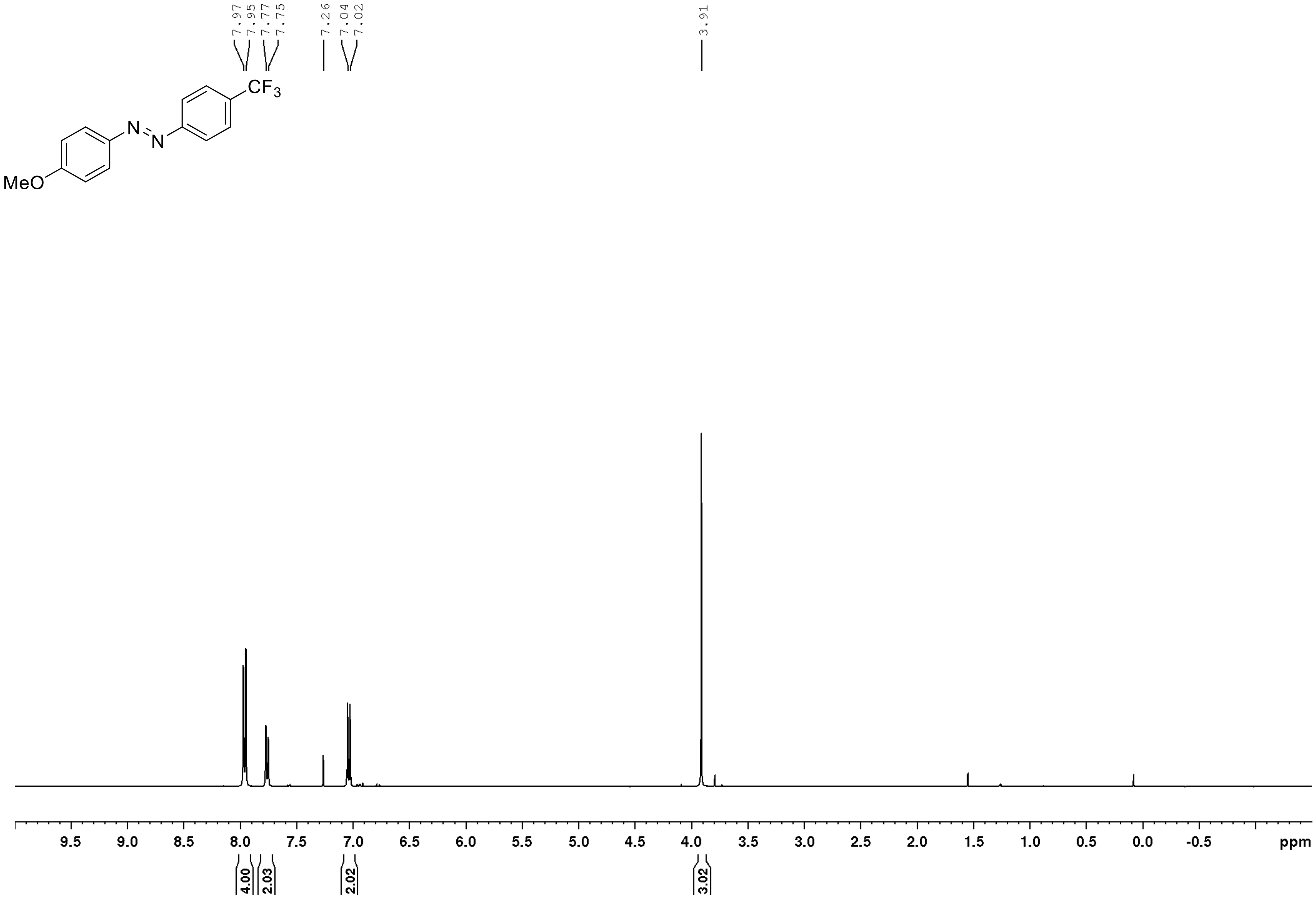

Figure S70. $^{13}C\{^{1}H\}$ NMR spectrum (101 MHz, $CDCl_3$) of (*E*)-1-(4-methoxyphenyl)-2-(4-(trifluoromethyl)phenyl)diazene (**6bn**).

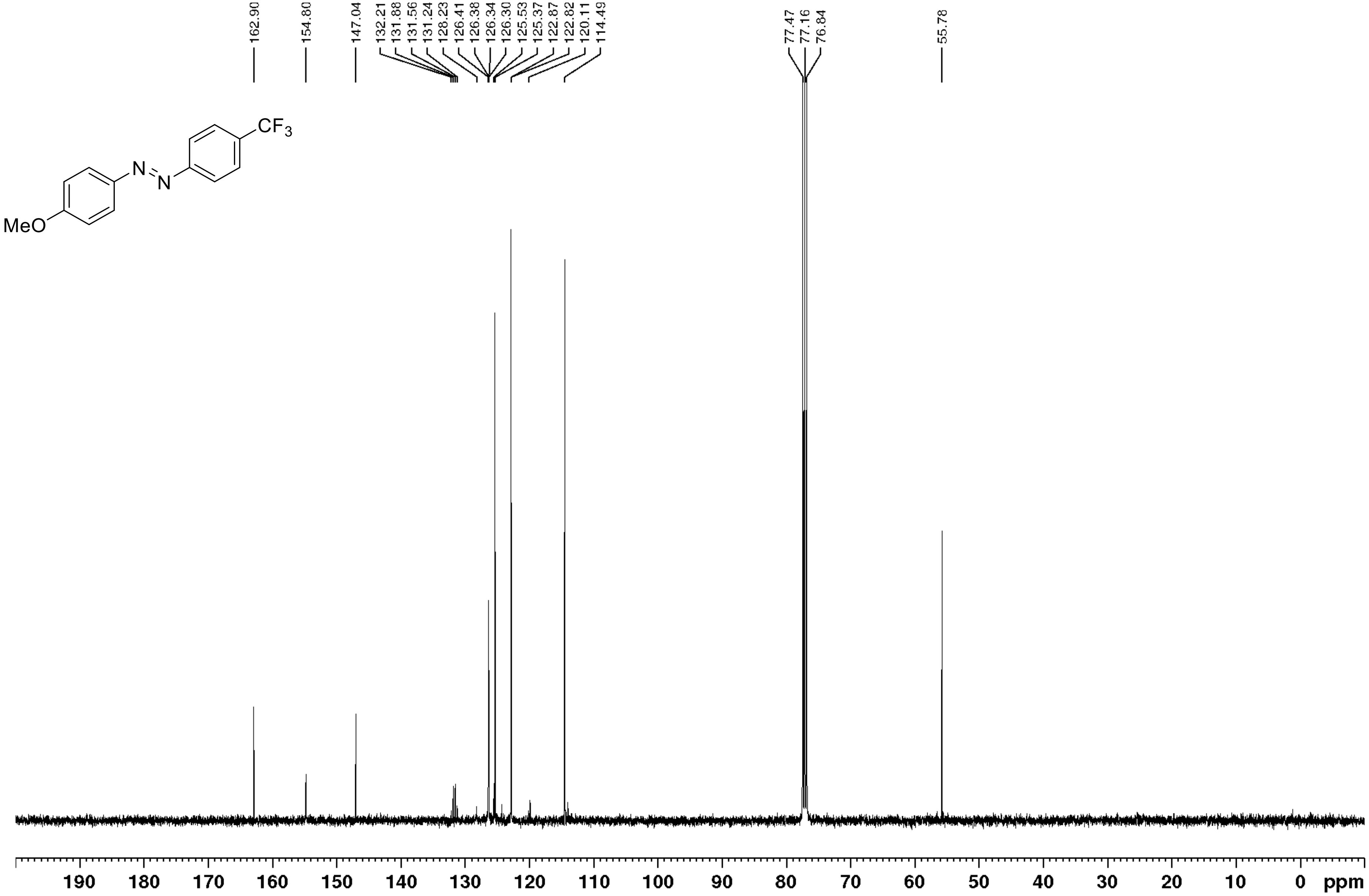

Figure S71. ^{19}F NMR spectrum (471 MHz, $CDCl_3$) of (*E*)-1-(4-methoxyphenyl)-2-(4-(trifluoromethyl)phenyl)diazene (**6bn**).

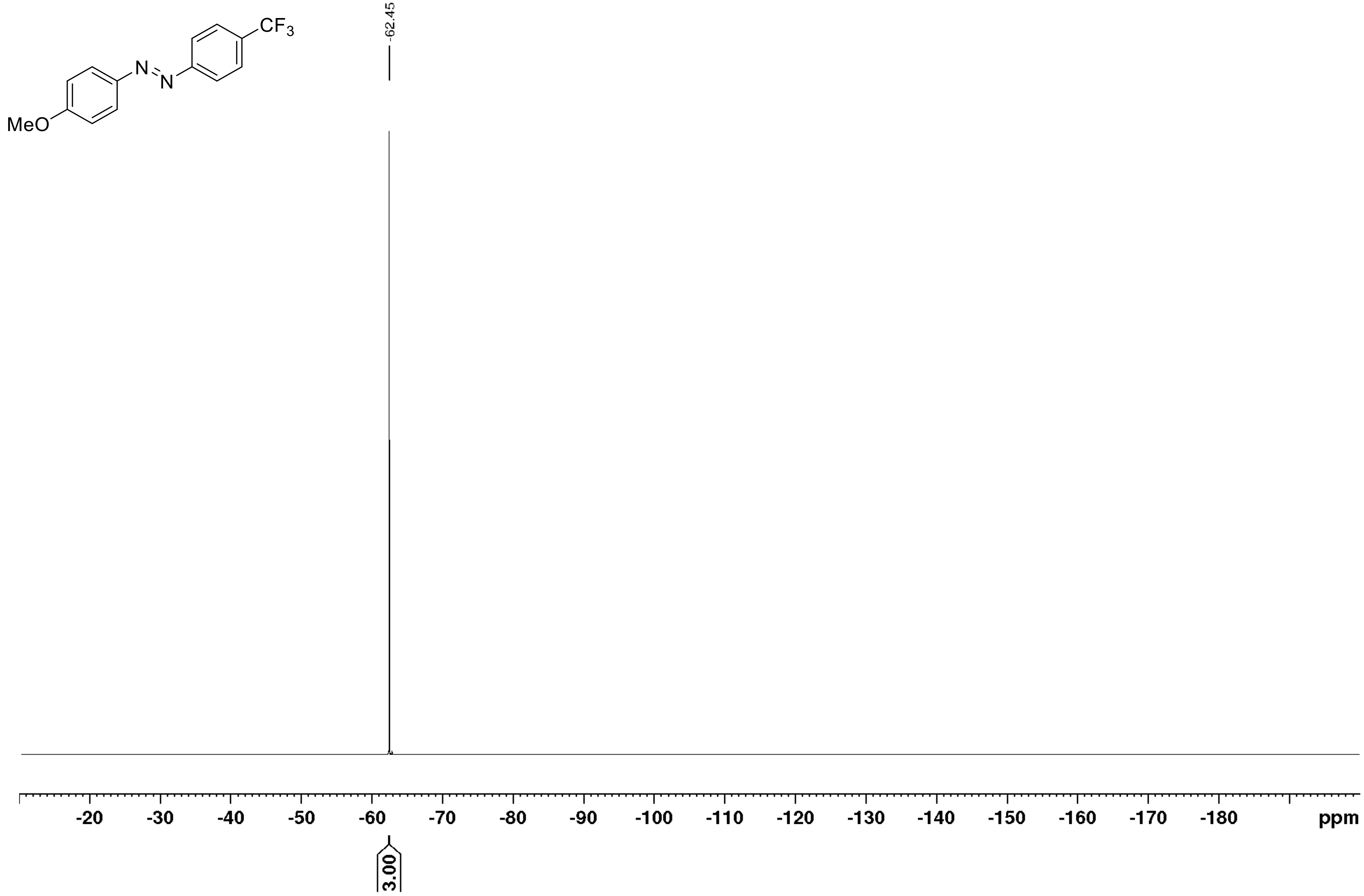

Figure S72. ^{1}H NMR spectrum (400 MHz, $CDCl_3$) of (*E*)-4-((4-methoxyphenyl)diazenyl)benzonitrile (**6bo**).

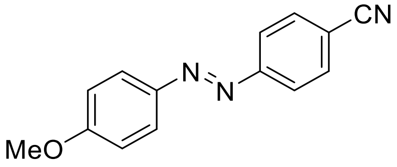

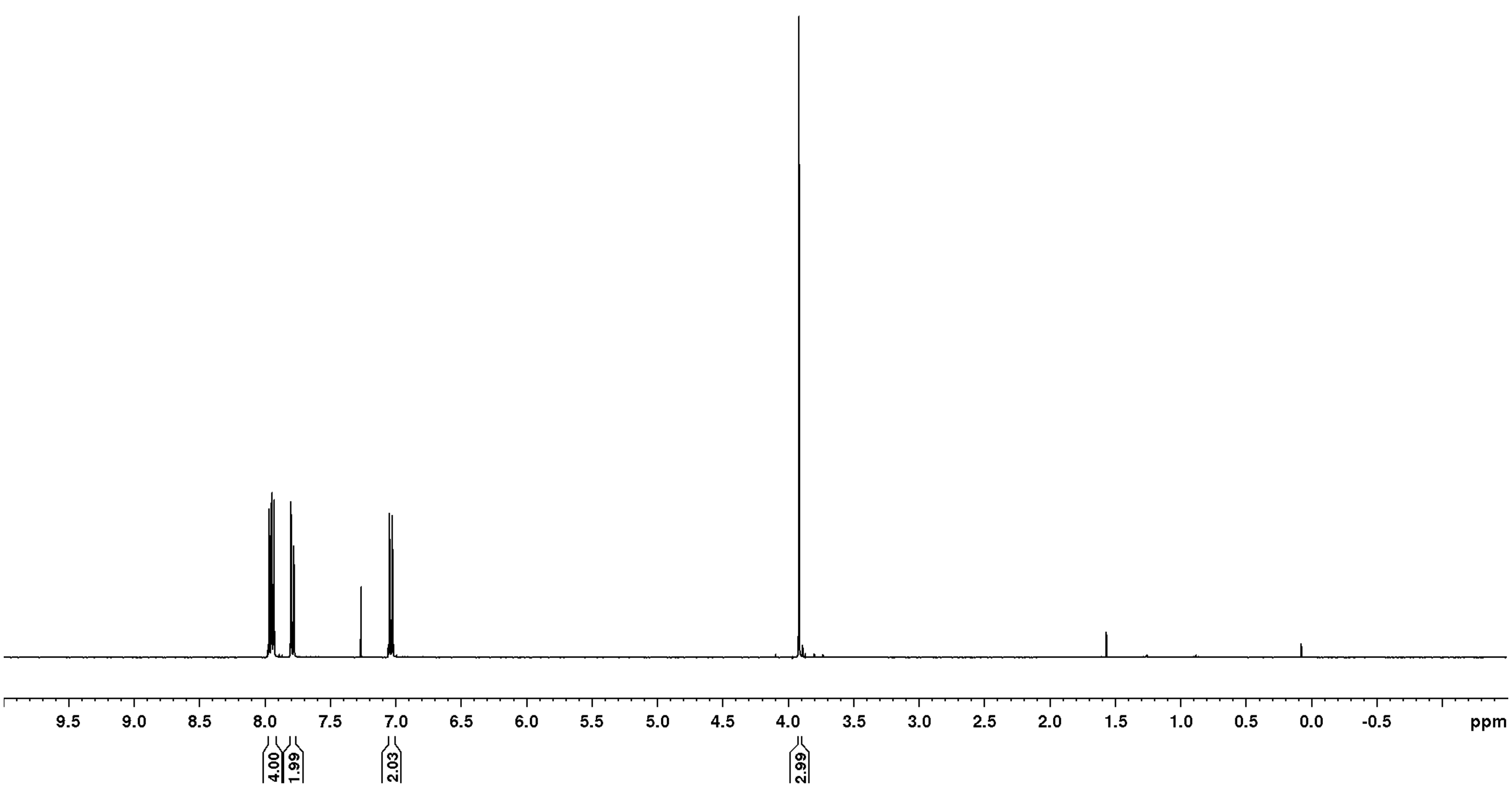

Figure S73. $^{13}C\{^{1}H\}$ NMR spectrum (101 MHz, $CDCl_3$) of (*E*)-4-((4-methoxyphenyl)diazenyl)benzonitrile (**6bo**).

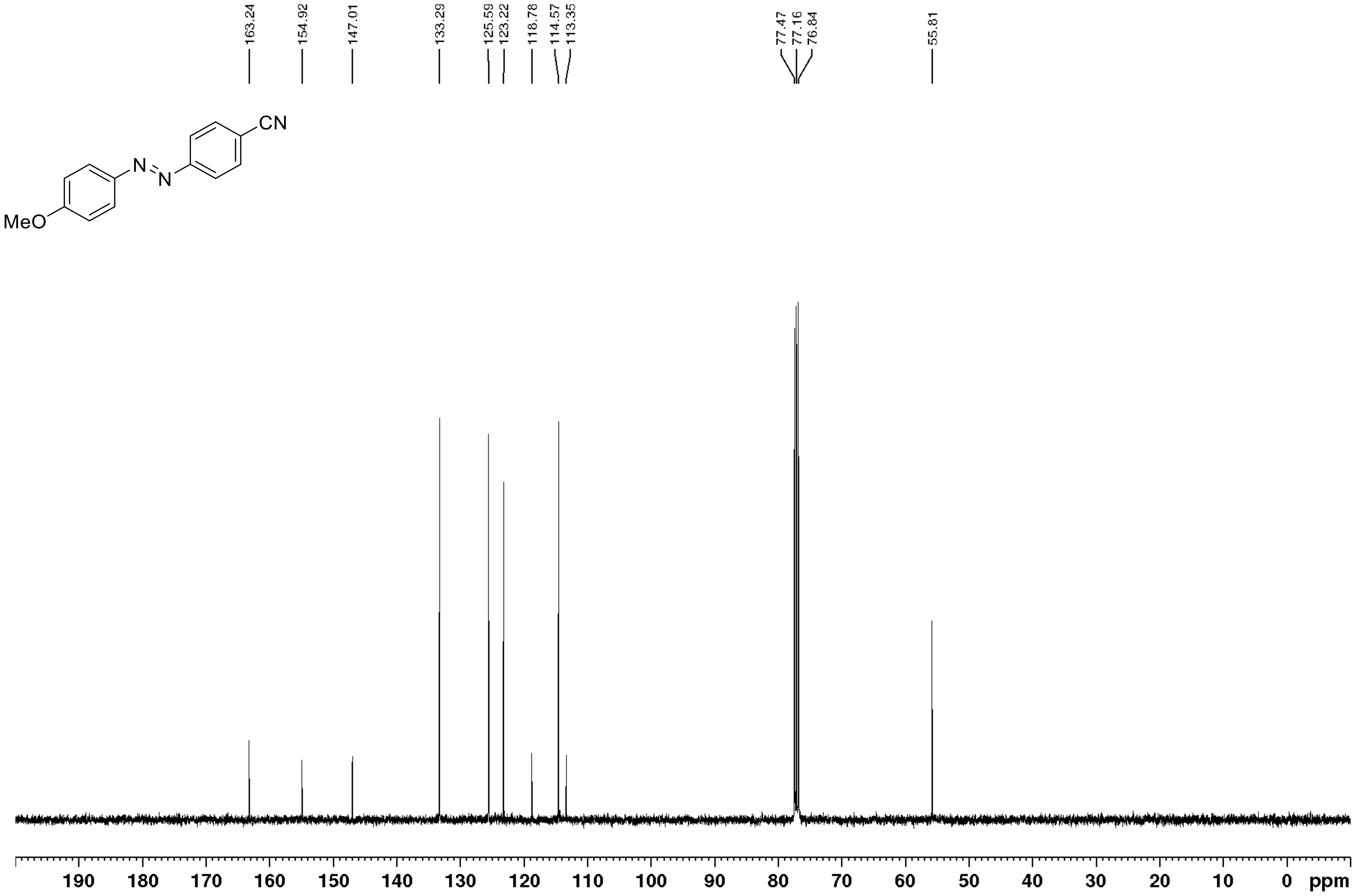

Figure S74. ^{1}H NMR spectrum (400 MHz, $CDCl_3$) of (*E*)-1-(4-methoxyphenyl)-2-(4-nitrophenyl)diazene (**6bp**).

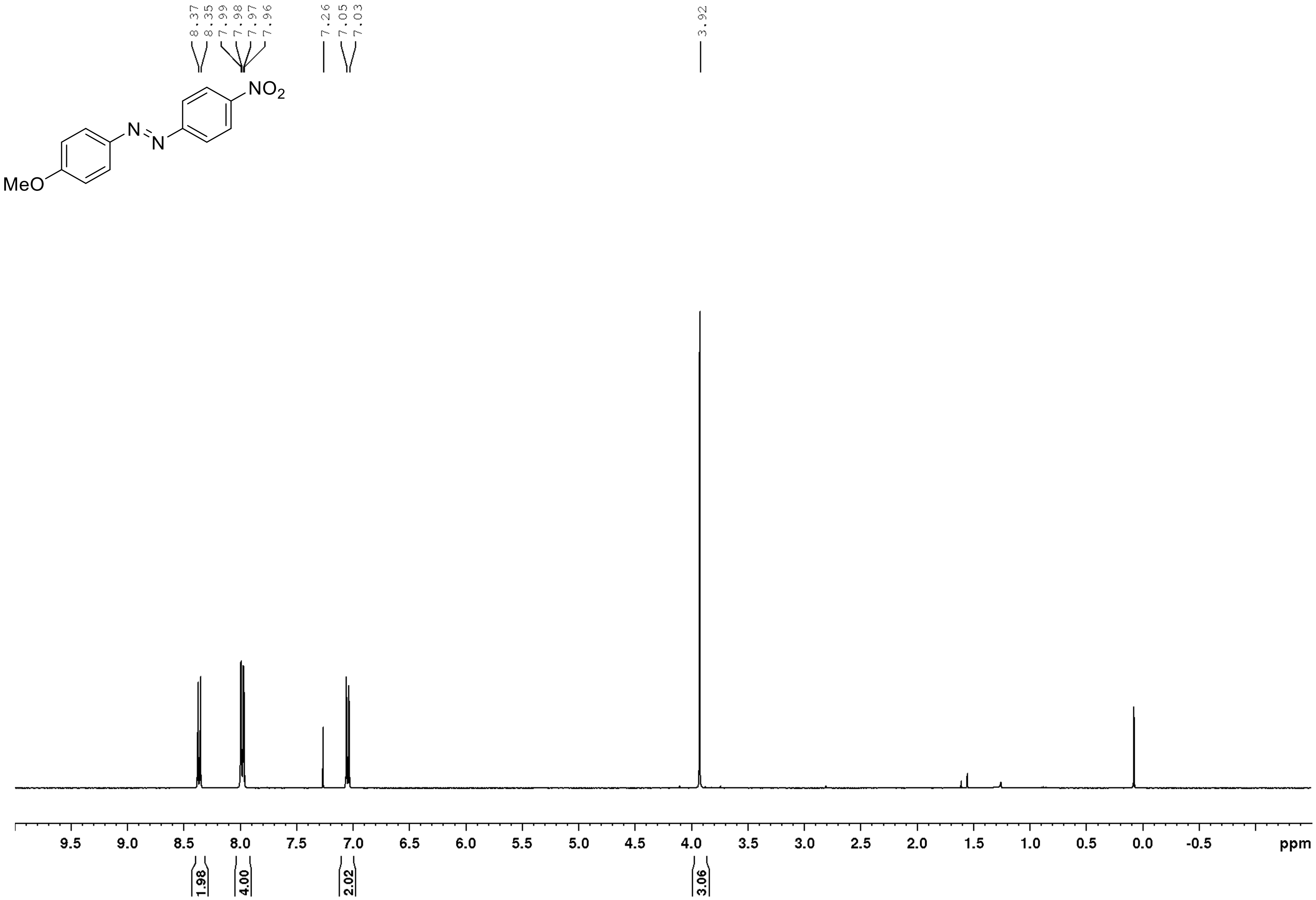

Figure S75. $^{13}C\{^{1}H\}$ NMR spectrum (101 MHz, $CDCl_3$) of (*E*)-1-(4-methoxyphenyl)-2-(4-nitrophenyl)diazene (**6bp**).

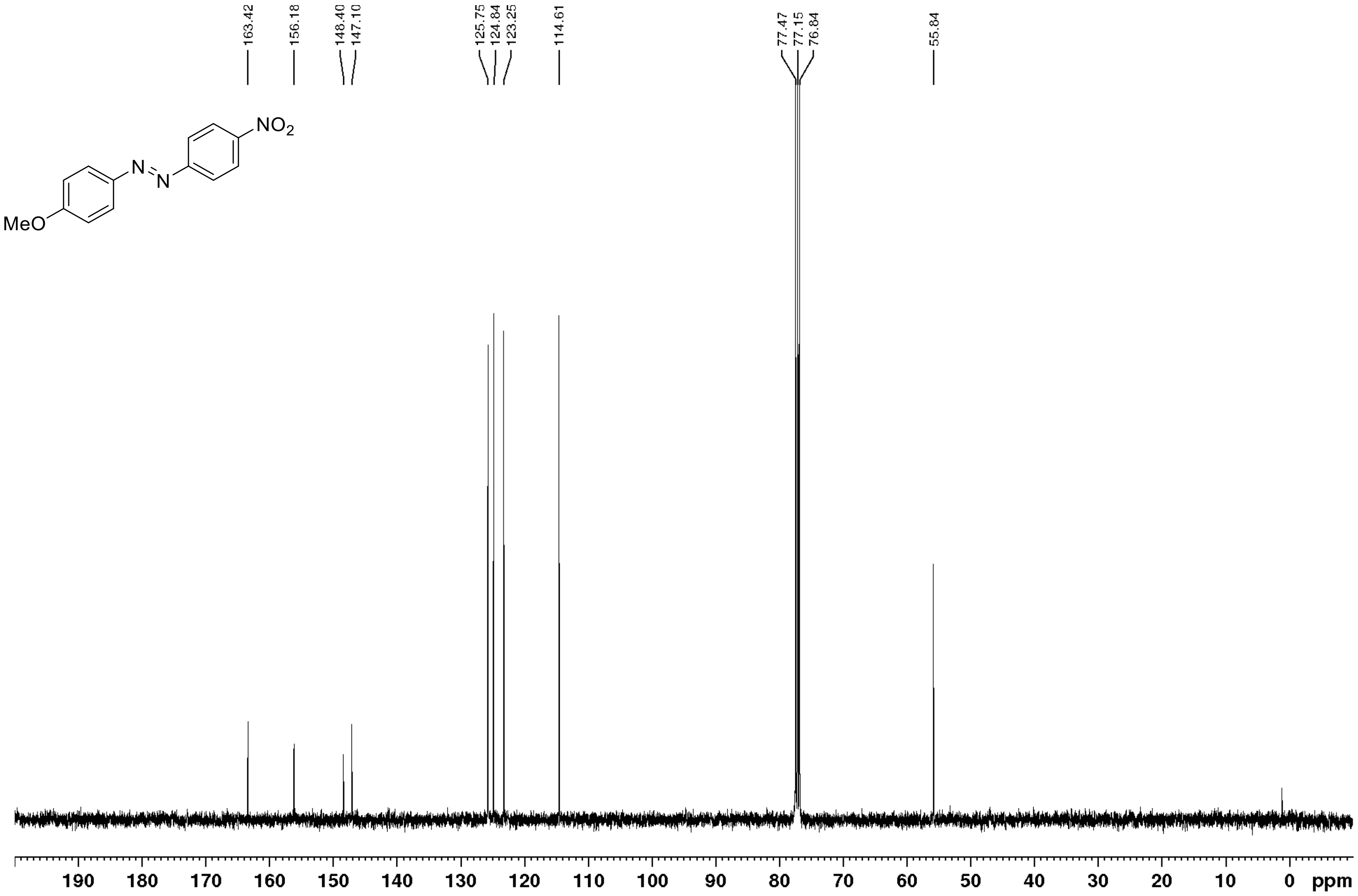

Figure S76. ^1H NMR spectrum (400 MHz, $CDCl_3$) of methyl (*E*)-4-((4-methoxyphenyl)diazenyl)benzoate (**6bq**).

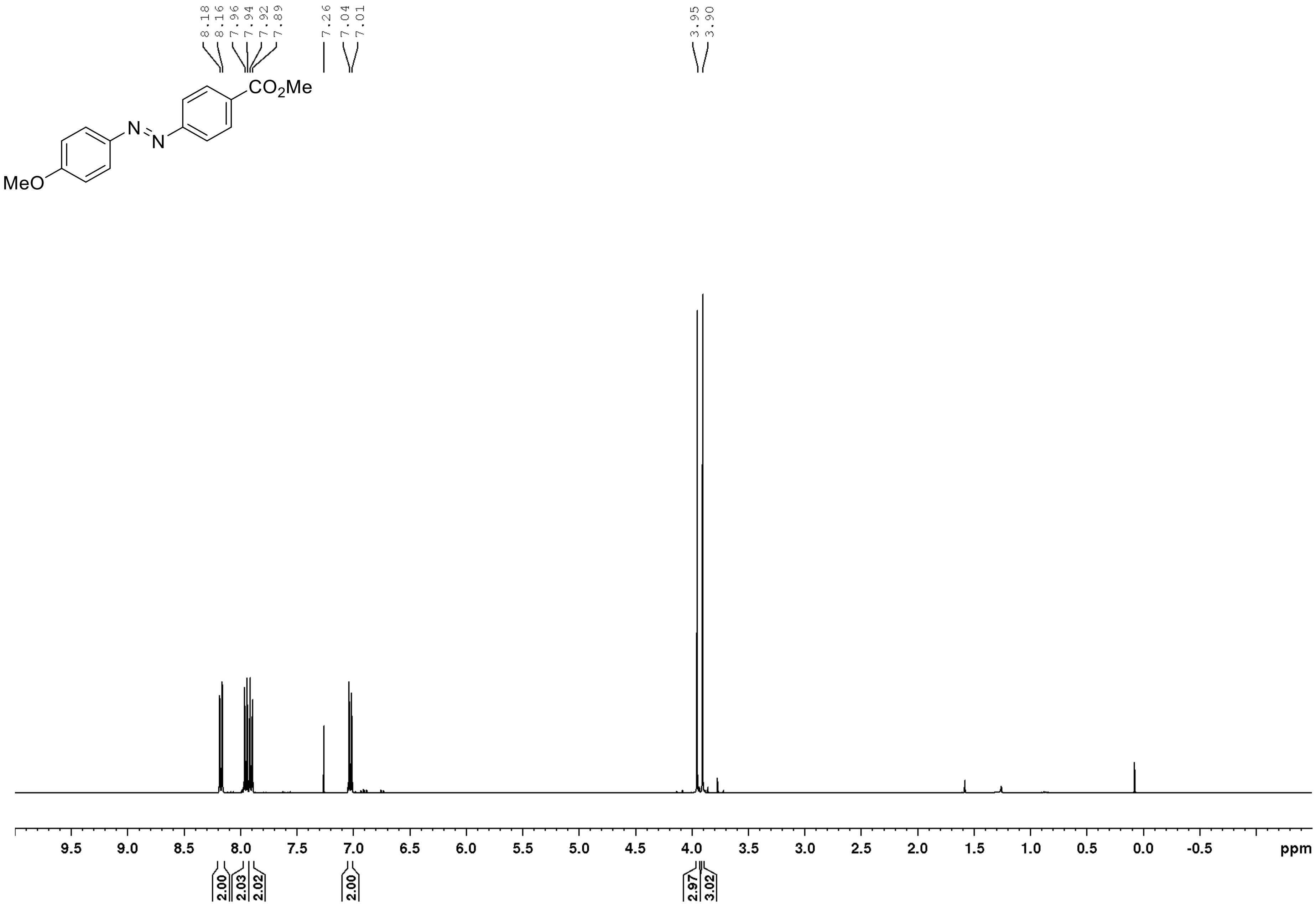

Figure S77. $^{13}C\{^1H\}$ NMR spectrum (101 MHz, $CDCl_3$) of methyl (*E*)-4-((4-methoxyphenyl)diazenyl)benzoate (**6bq**).

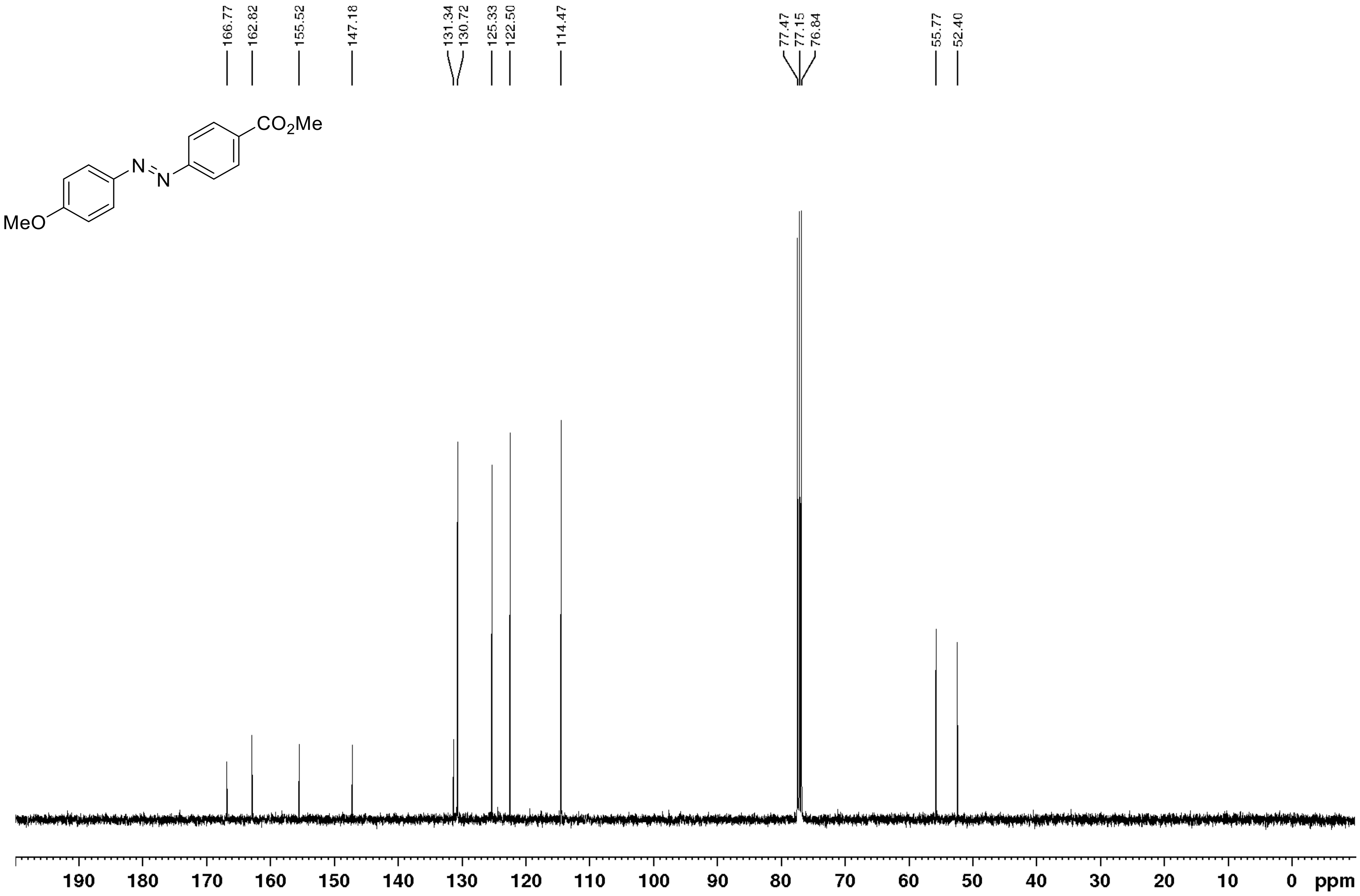

Figure S78. ^{1}H NMR spectrum (500 MHz, $CDCl_3$) of ethyl (*E*)-2-((4-methoxyphenyl)diazenyl)benzoate (**6br**).

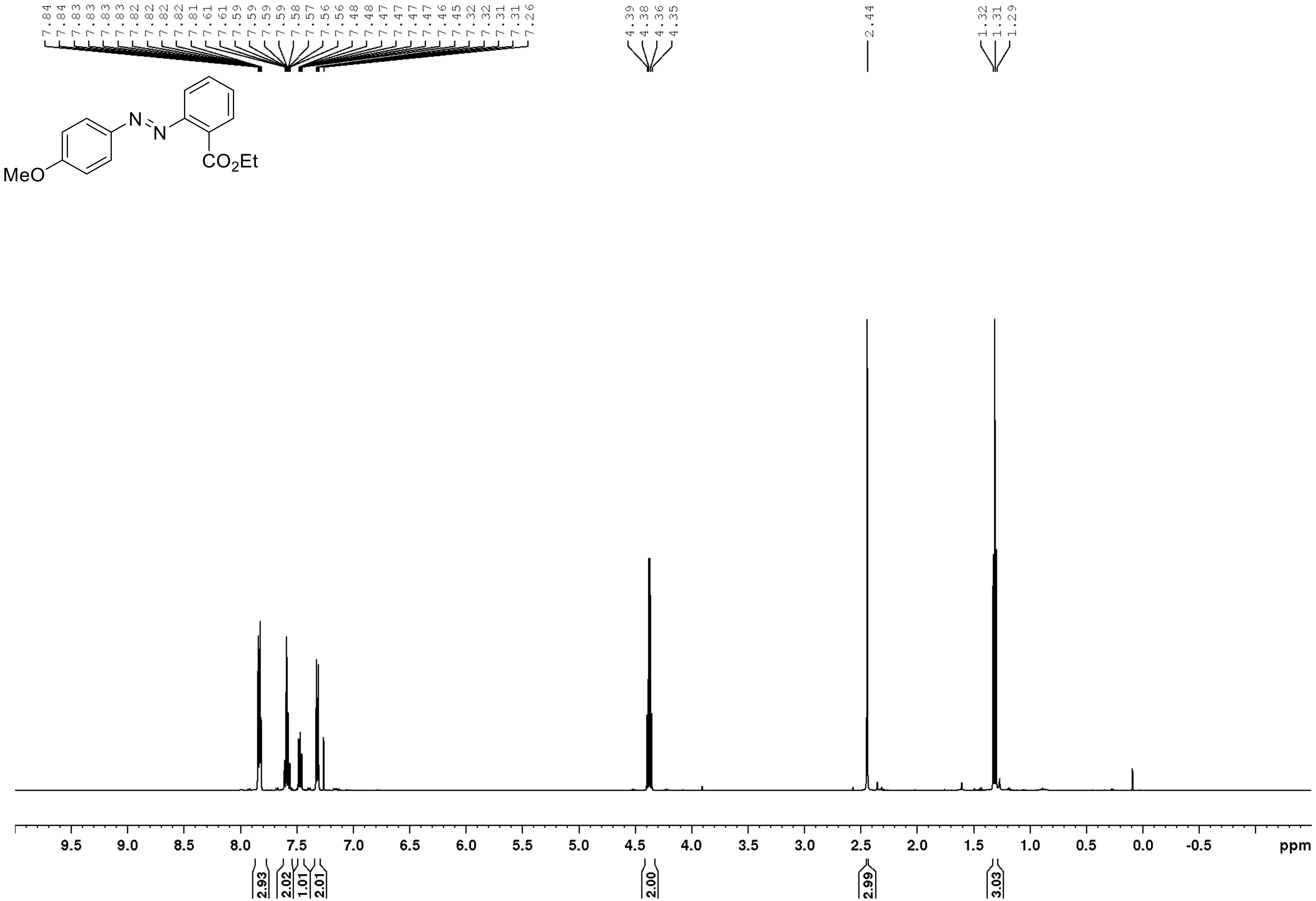

Figure S79. $^{13}C\{^1H\}$ NMR spectrum (126 MHz, $CDCl_3$) of ethyl (*E*)-2-((4-methoxyphenyl)diazenyl)benzoate (**6br**).

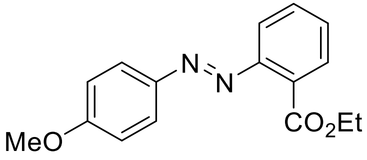

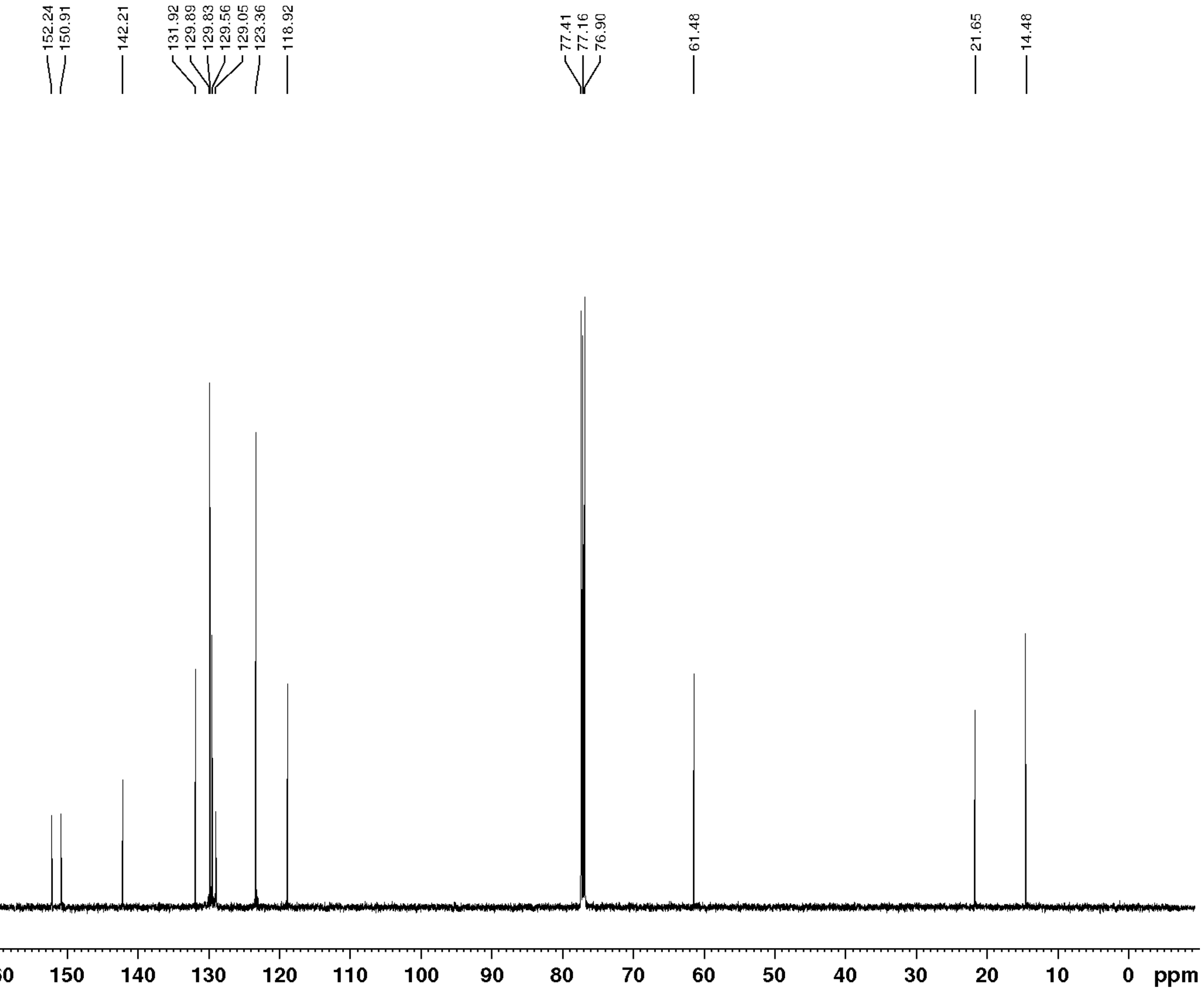

Figure S80. ^{1}H NMR spectrum (400 MHz, $CDCl_3$) of (*E*)-1-(4-((4-methoxyphenyl)diazenyl)phenyl)ethan-1-one (**6bs**).

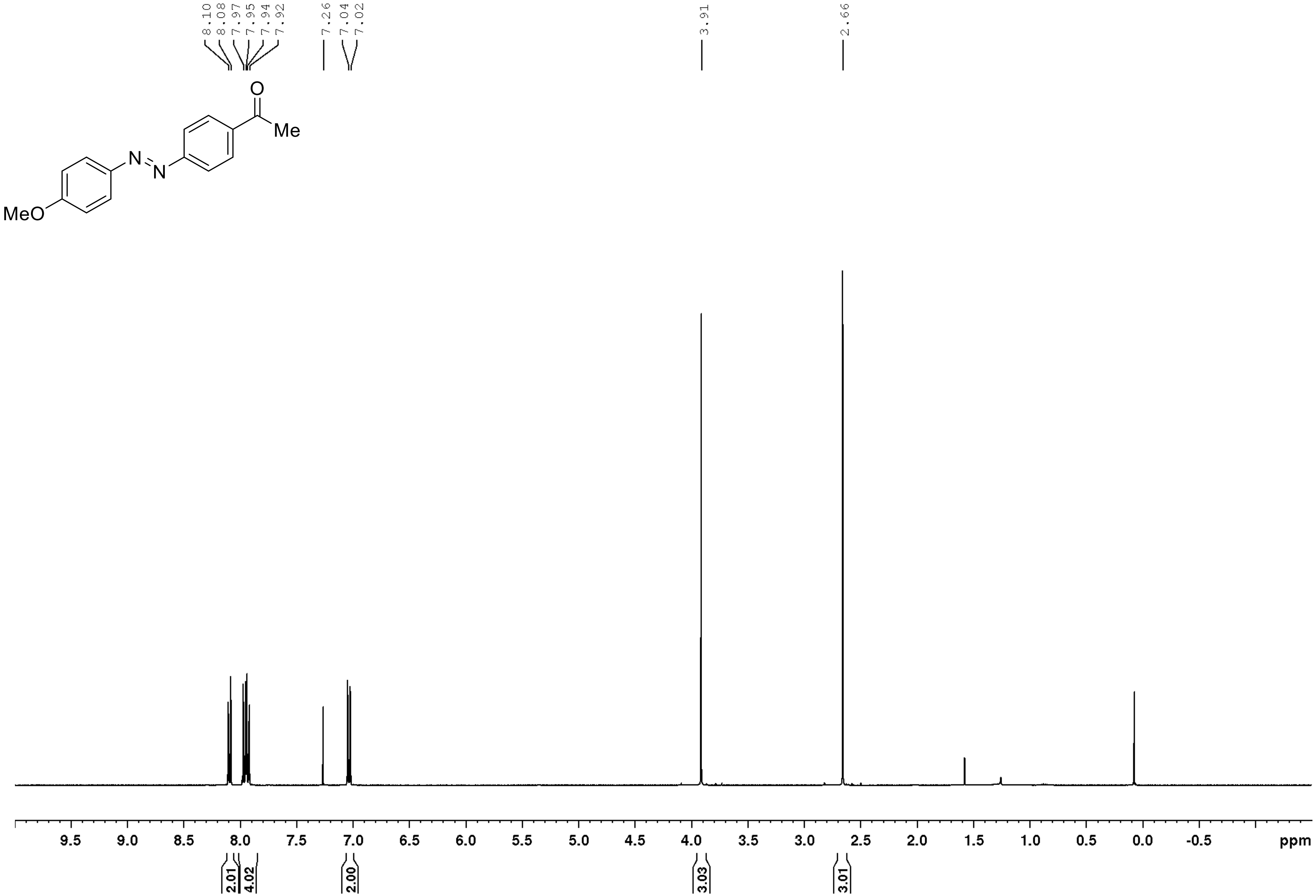

Figure S81. $^{13}C\{^{1}H\}$ NMR spectrum (101 MHz, $CDCl_3$) of (*E*)-1-(4-((4-methoxyphenyl)diazenyl)phenyl)ethan-1-one (**6bs**).

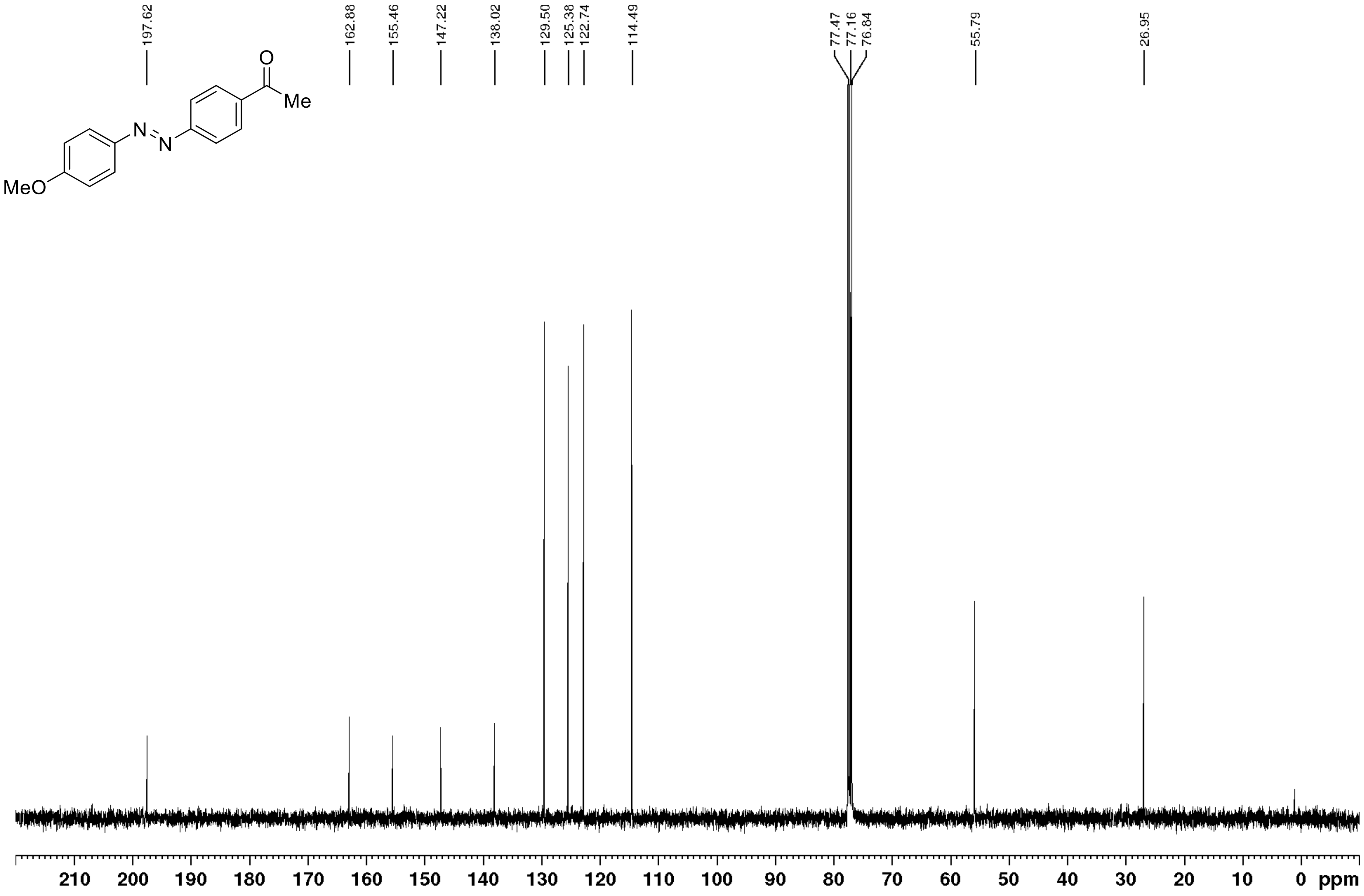

Figure S82. ^{1}H NMR spectrum (400 MHz, $CDCl_3$) of (*E*)-(4-((4-methoxyphenyl)diazenyl)phenyl)(phenyl)methanone (**6bt**).

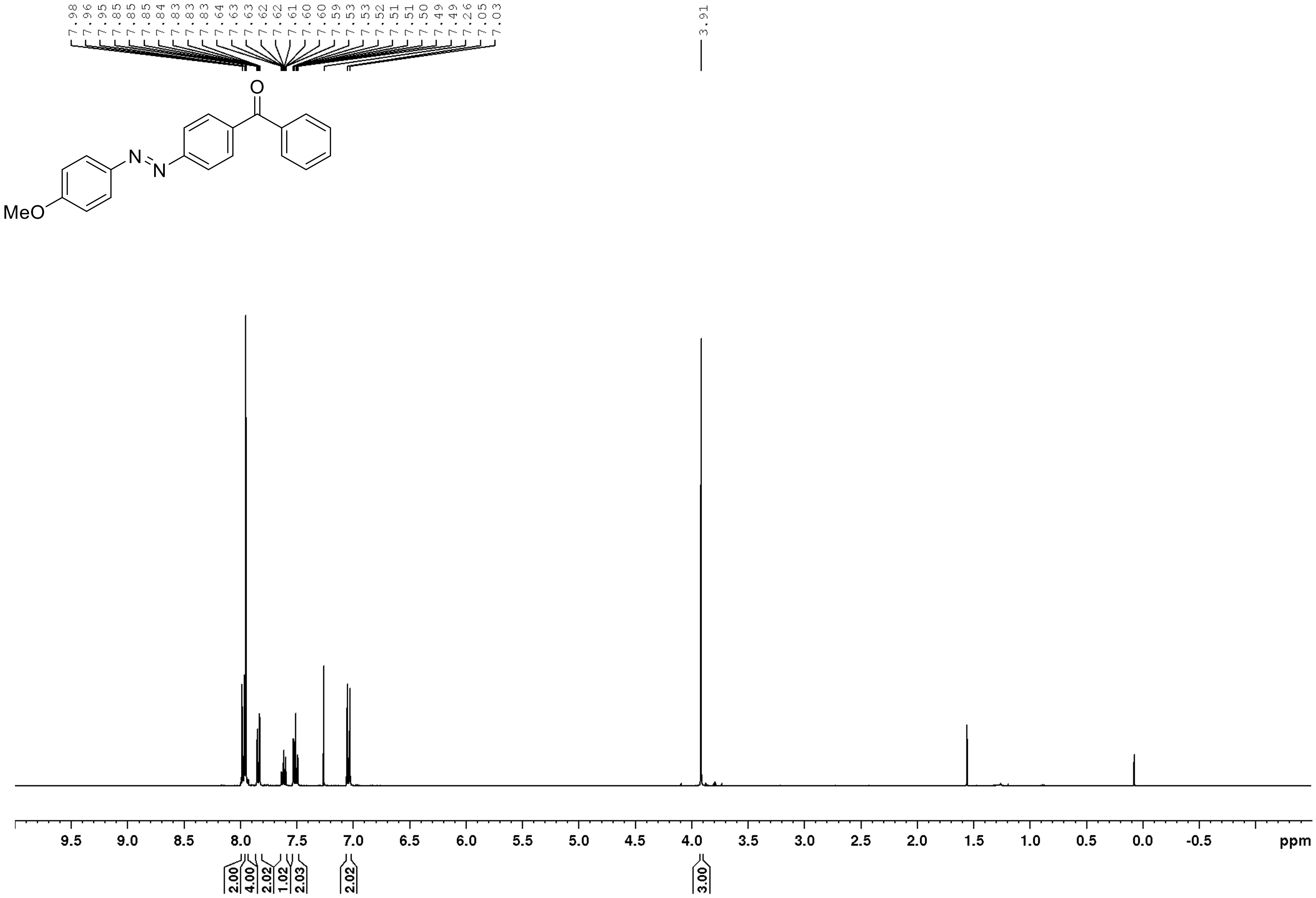

Figure S83. $^{13}C\{^1H\}$ NMR spectrum (101 MHz, $CDCl_3$) of (*E*)-(4-((4-methoxyphenyl)diazenyl)phenyl)(phenyl)methanone (**6bt**).

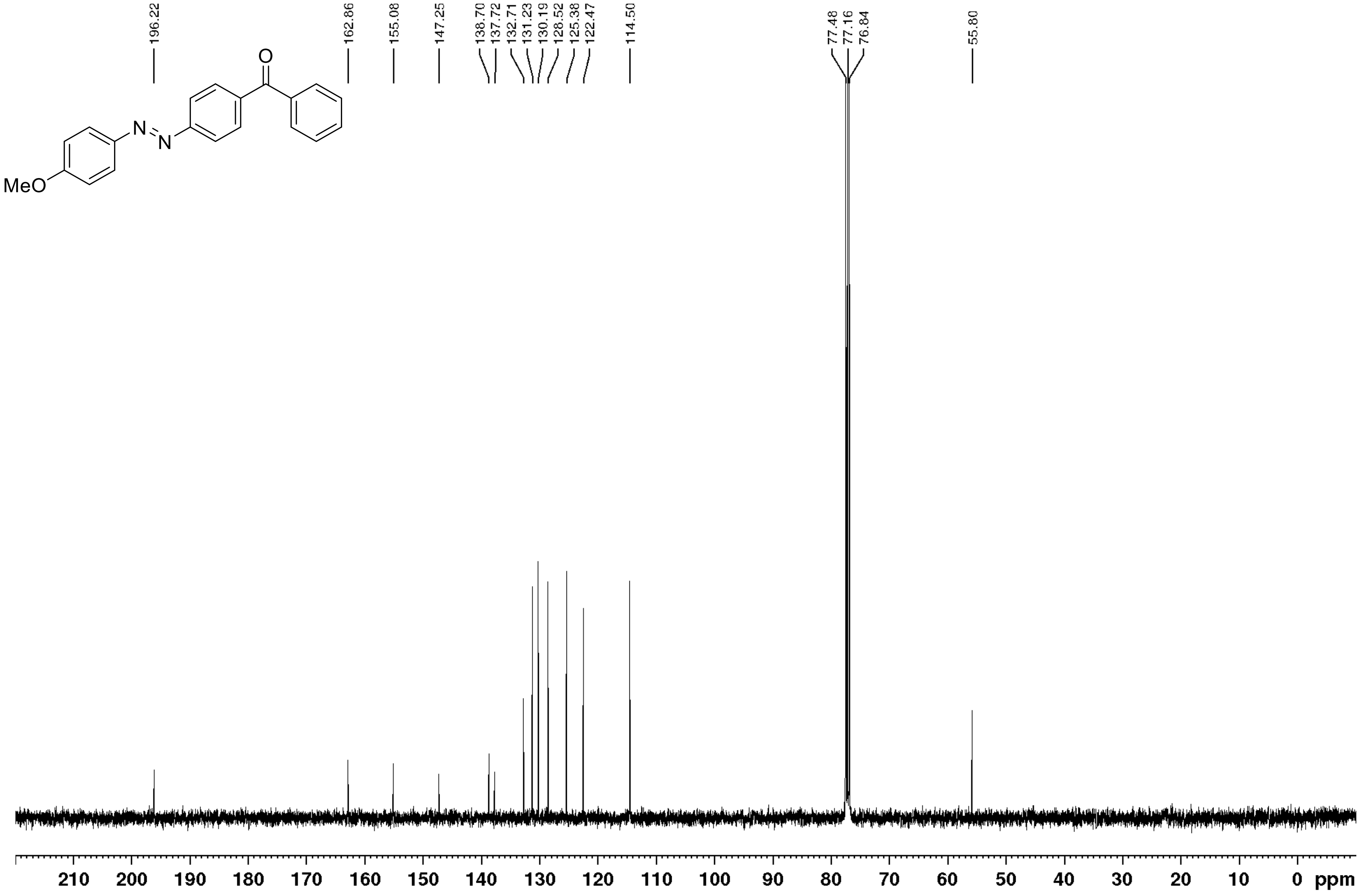

Figure S84. ^{1}H NMR spectrum (700 MHz, $CDCl_3$) of (*E*)-1-(3,5-bis(trifluoromethyl)phenyl)-2-(4-fluoro-2,6-dimethylphenyl)diazene (**6ju**).

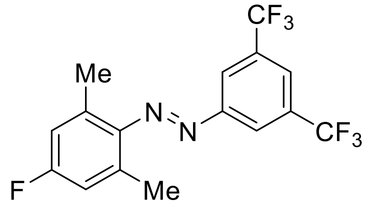

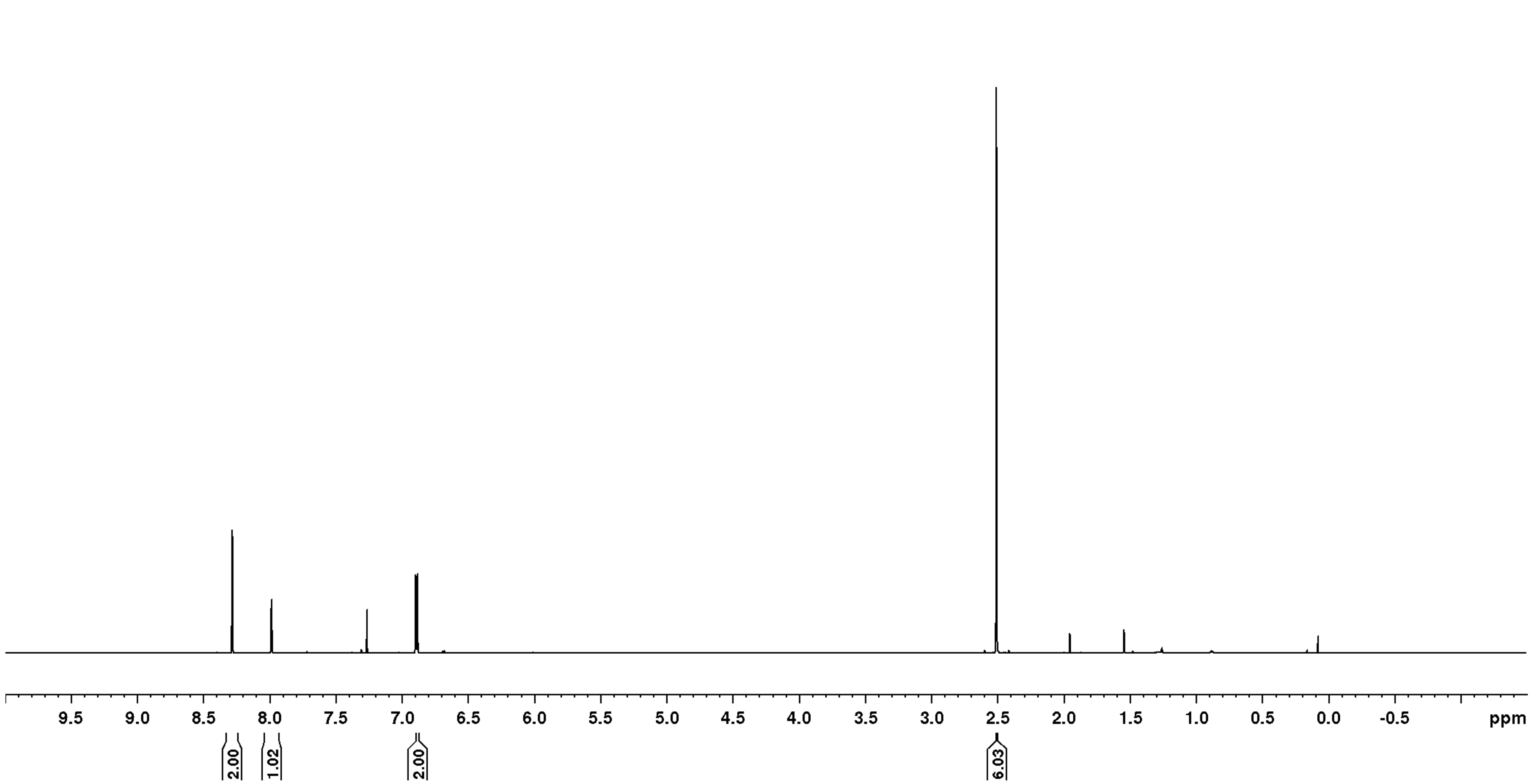

Figure S85. $^{13}C\{^1H\}$ NMR spectrum (176 MHz, $CDCl_3$) of (*E*)-1-(3,5-bis(trifluoromethyl)phenyl)-2-(4-fluoro-2,6-dimethylphenyl)diazene (**6ju**).

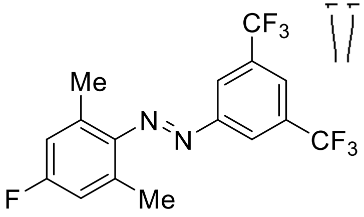

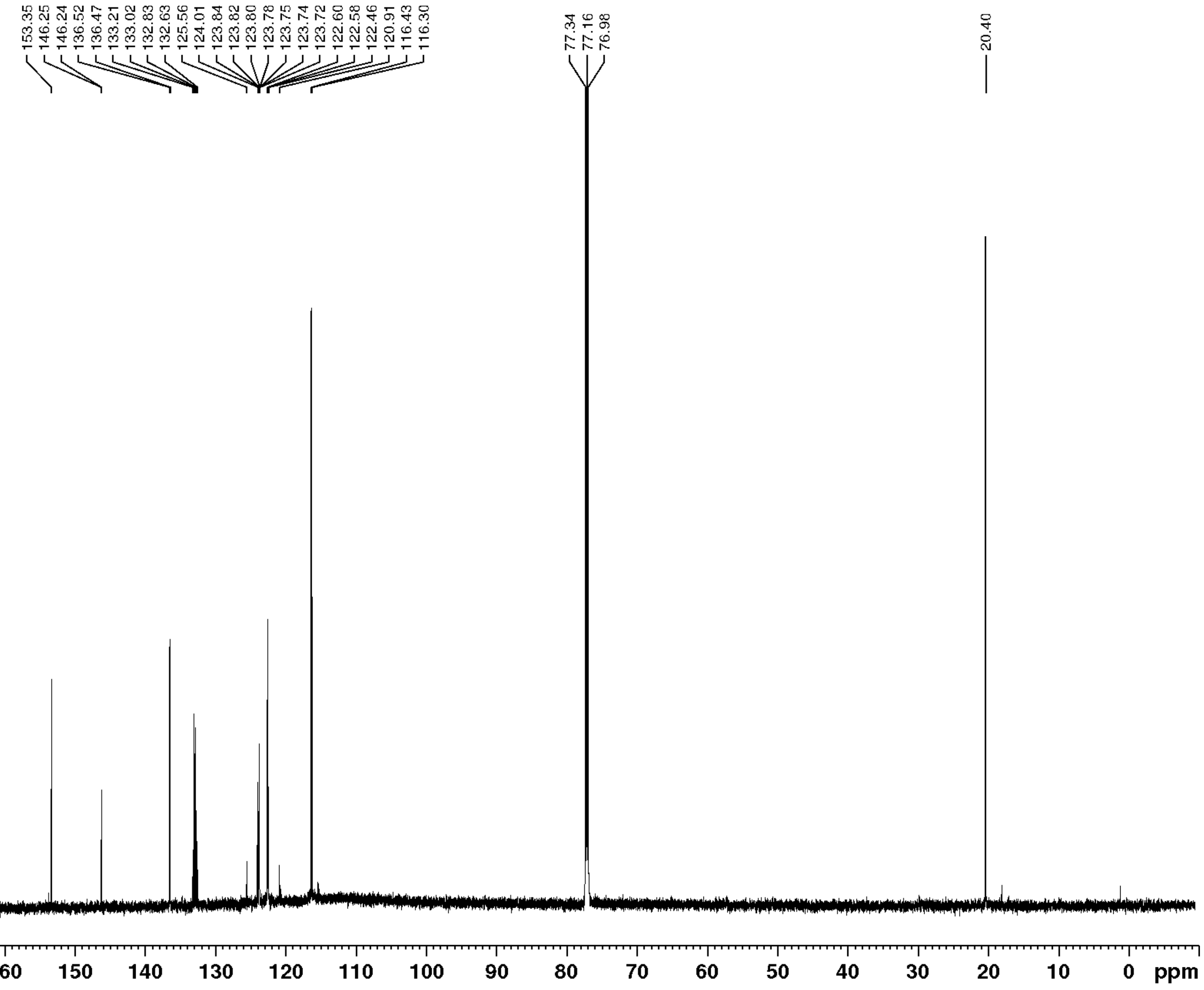

Figure S86. ^{19}F NMR spectrum (659 MHz, $CDCl_3$) of (*E*)-1-(3,5-bis(trifluoromethyl)phenyl)-2-(4-fluoro-2,6-dimethylphenyl)diazene (**6ju**).

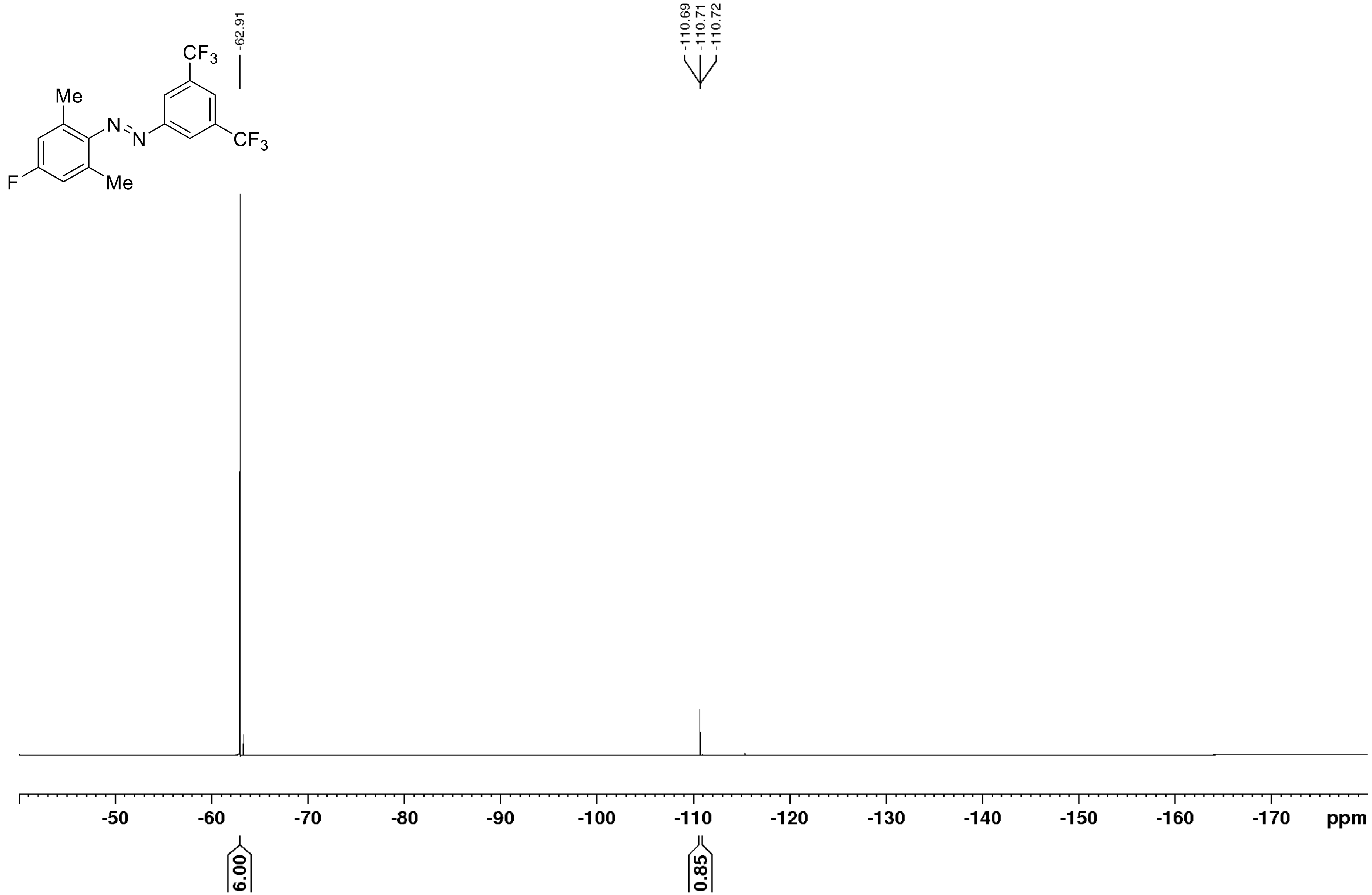

Figure S87. ^{1}H NMR spectrum (500 MHz, $CDCl_3$) of (*E*)-1-(4-(*p*-tolyldiazenyl)phenyl)cyclopropane-1-carbonitrile (**6av**).

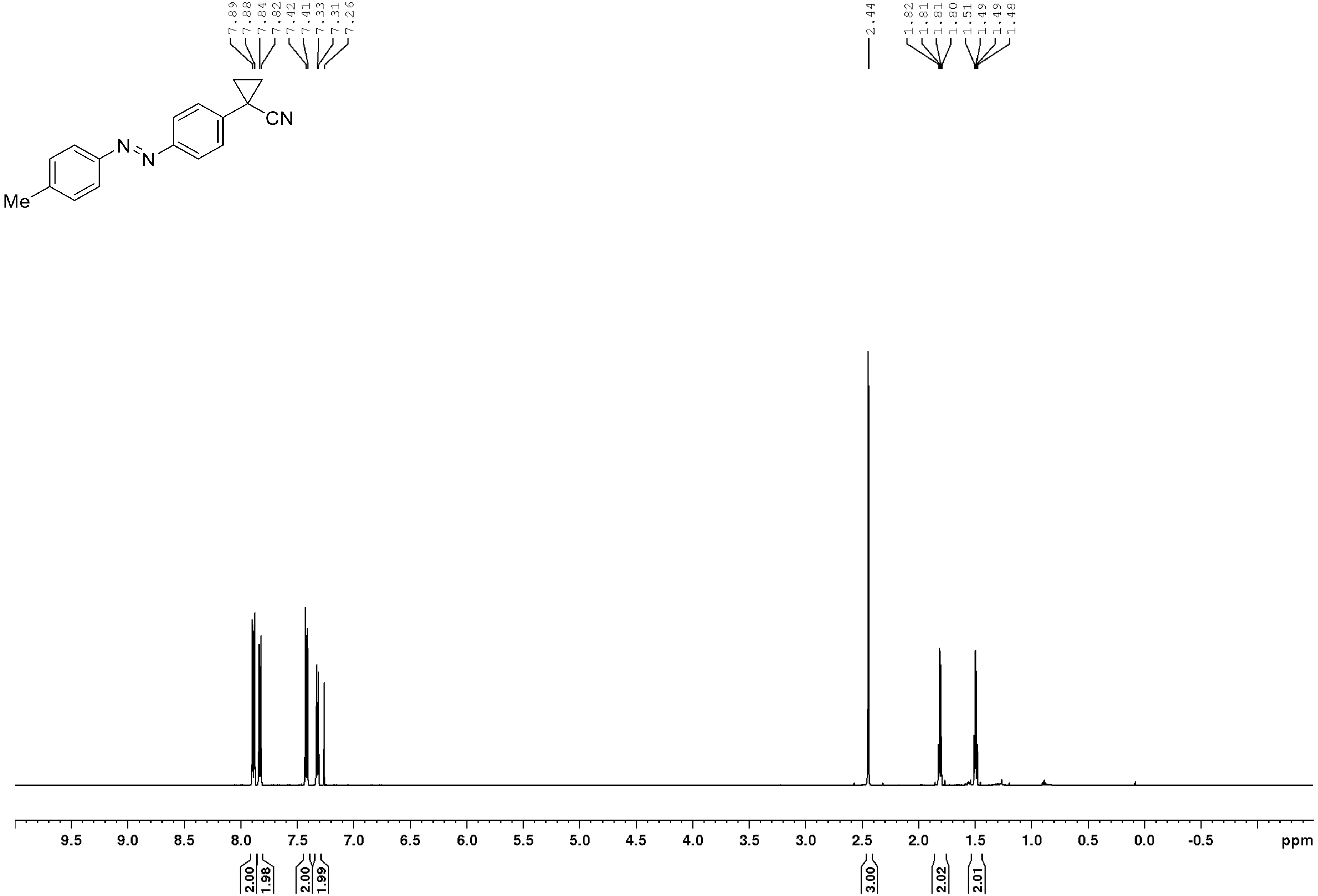

Figure S88. $^{13}C\{^1H\}$ NMR spectrum (126 MHz, $CDCl_3$) of (*E*)-1-(4-(*p*-tolyldiazenyl)phenyl)cyclopropane-1-carbonitrile (**6av**).

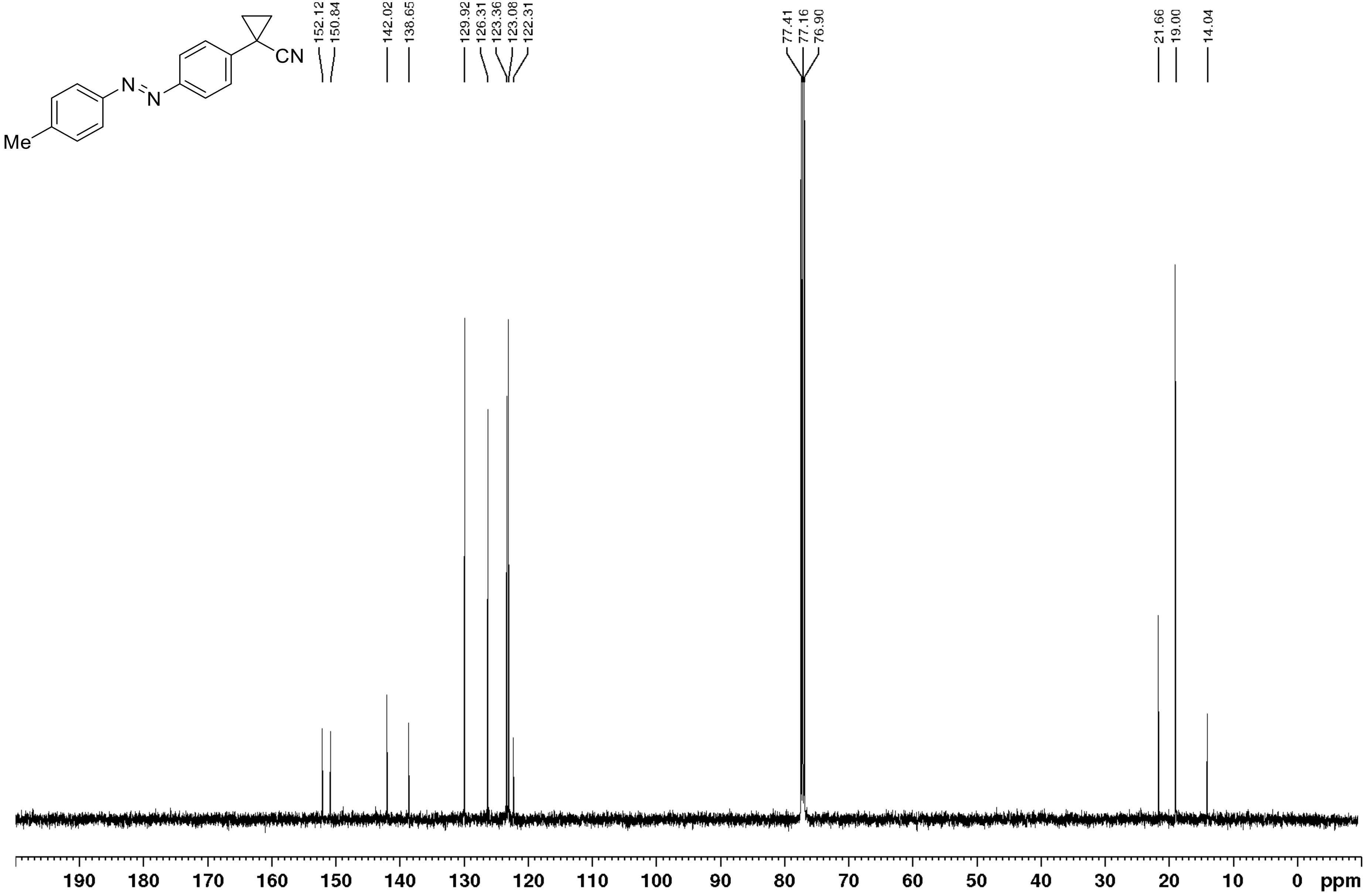

Figure S89. ^{1}H NMR spectrum (400 MHz, $CDCl_3$) of ethyl (*S*,*E*)-2-acetamido-3-(4-(*p*-tolyldiazenyl)phenyl)propanoate (**6aw**).

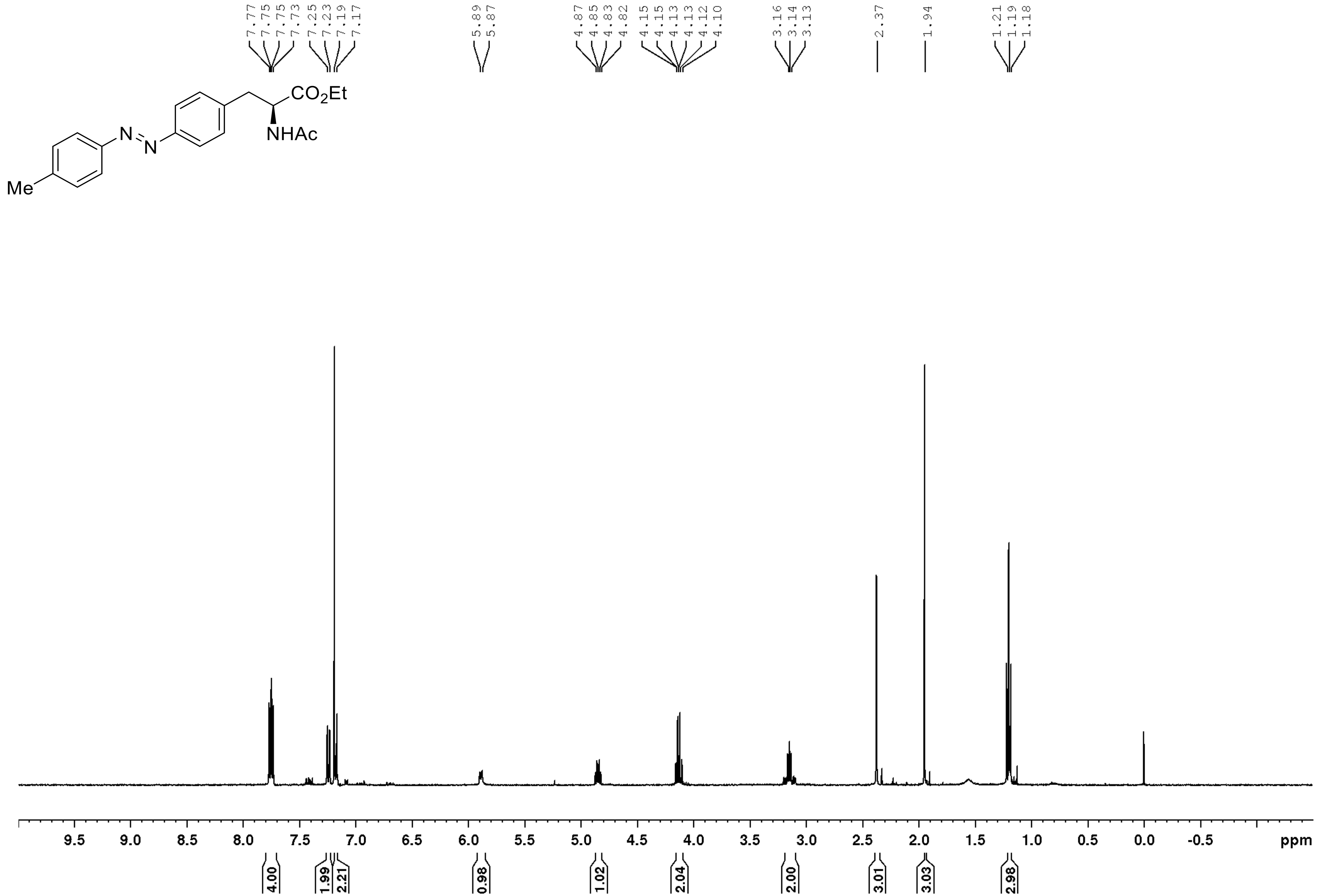

Figure S90. $^{13}C\{^{1}H\}$ NMR spectrum (101 MHz, $CDCl_3$) of ethyl (*S*,*E*)-2-acetamido-3-(4-(*p*-tolyldiazenyl)phenyl)propanoate (**6aw**).

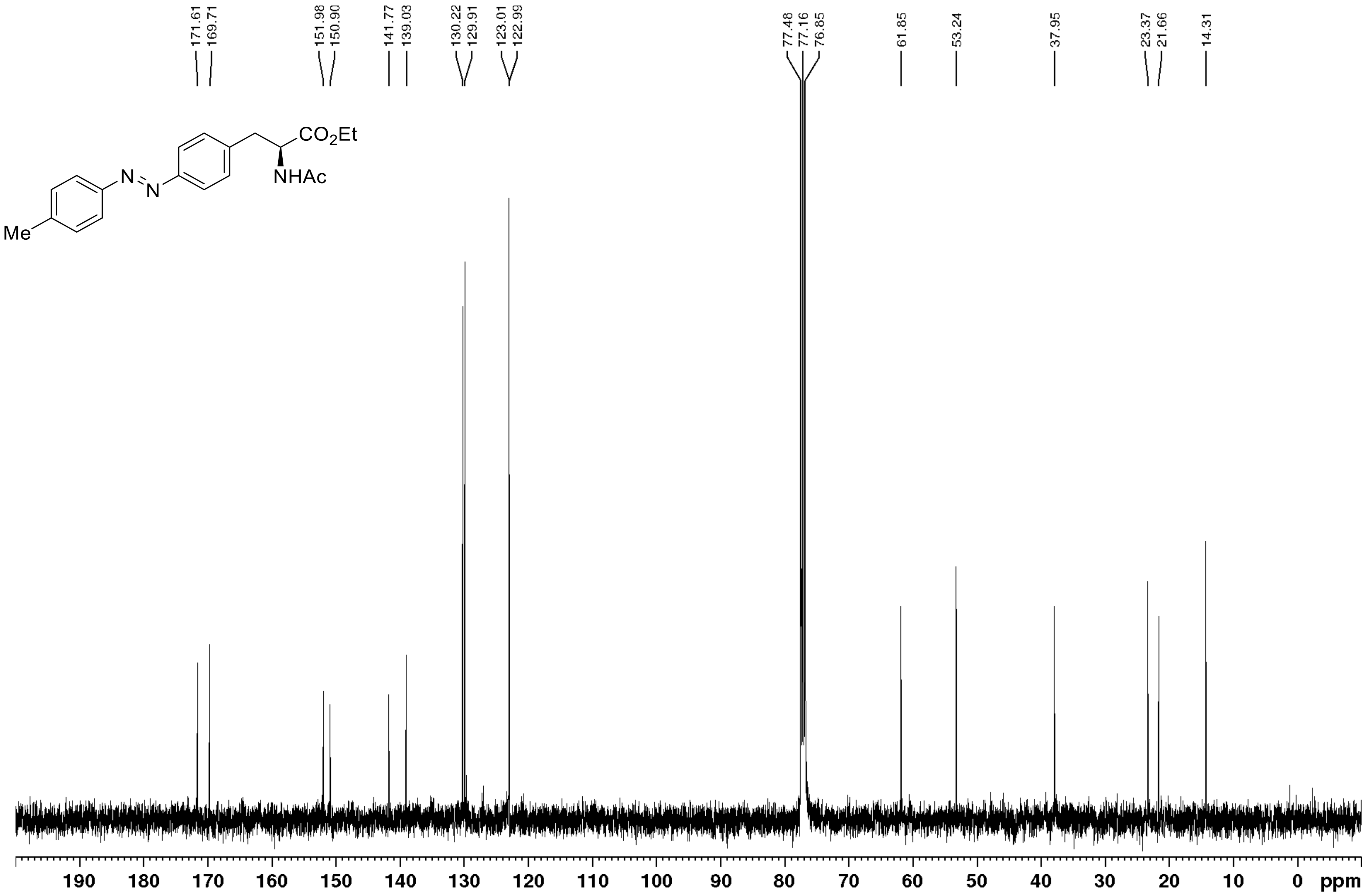

Figure S91. ^{1}H NMR spectrum (400 MHz, $CDCl_3$) of (*E*)-2-((4-chlorophenyl)diazenyl)-5-methylpyridine (**9ea**).

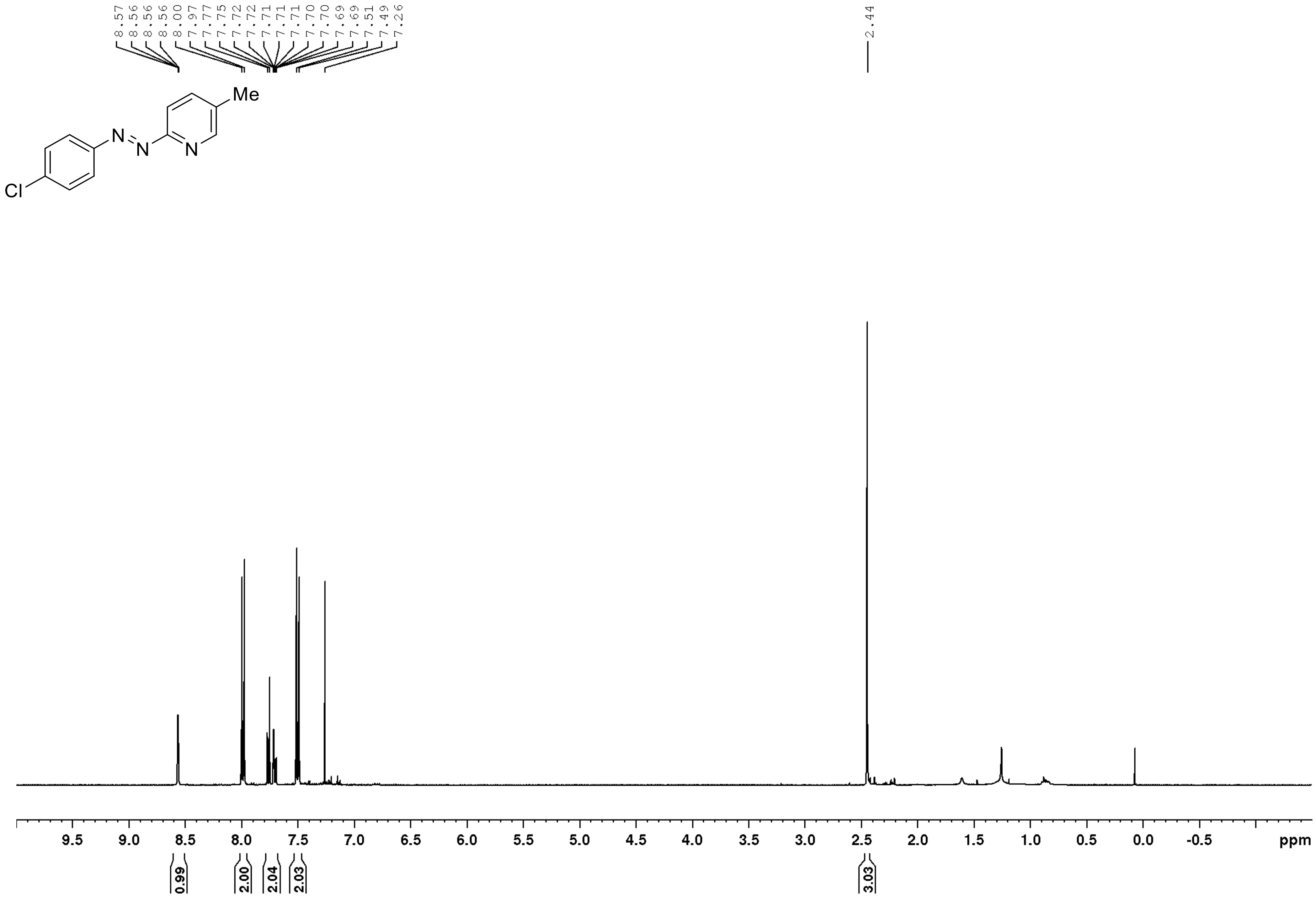

Figure S92. $^{13}C\{^1H\}$ NMR spectrum (101 MHz, $CDCl_3$) of (*E*)-2-((4-chlorophenyl)diazenyl)-5-methylpyridine (**9ea**).

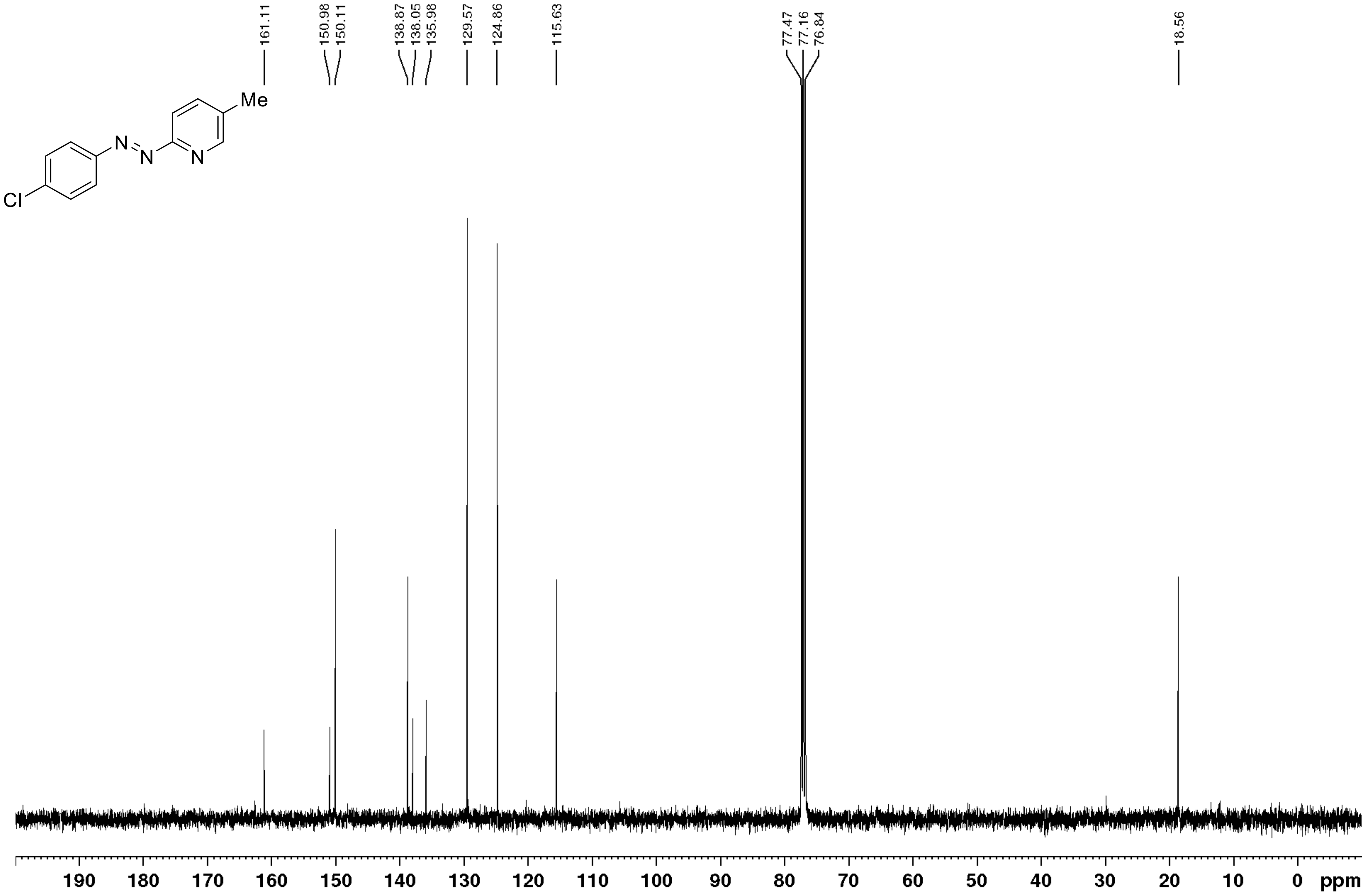

Figure S93. ^{1}H NMR spectrum (400 MHz, $CDCl_3$) of (*E*)-1-(4-chlorophenyl)-2-(thiophen-3-yl)diazene (**9eb**).

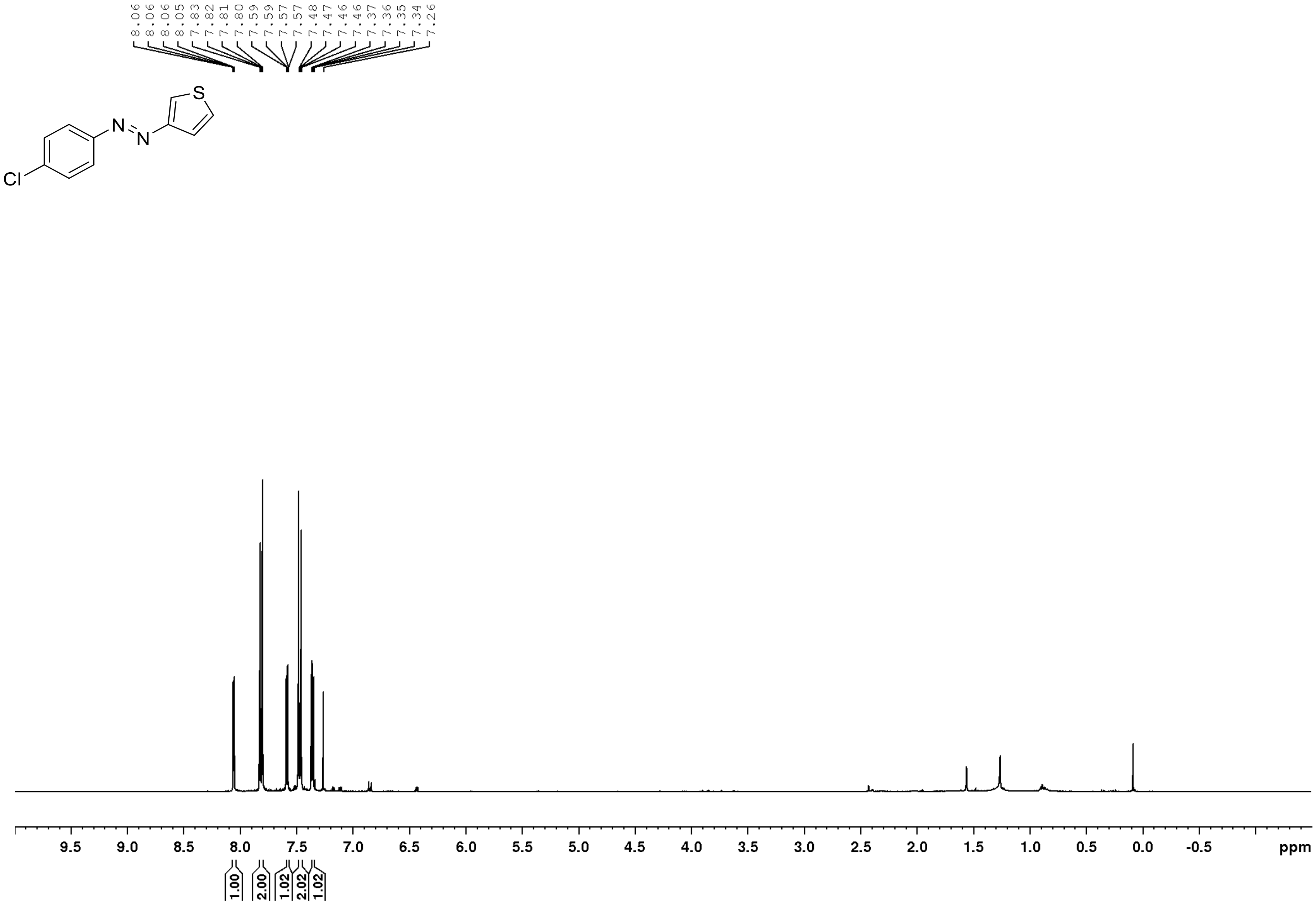

Figure S94. $^{13}C\{^{1}H\}$ NMR spectrum (101 MHz, $CDCl_3$) of (*E*)-1-(4-chlorophenyl)-2-(thiophen-3-yl)diazene (**9eb**).

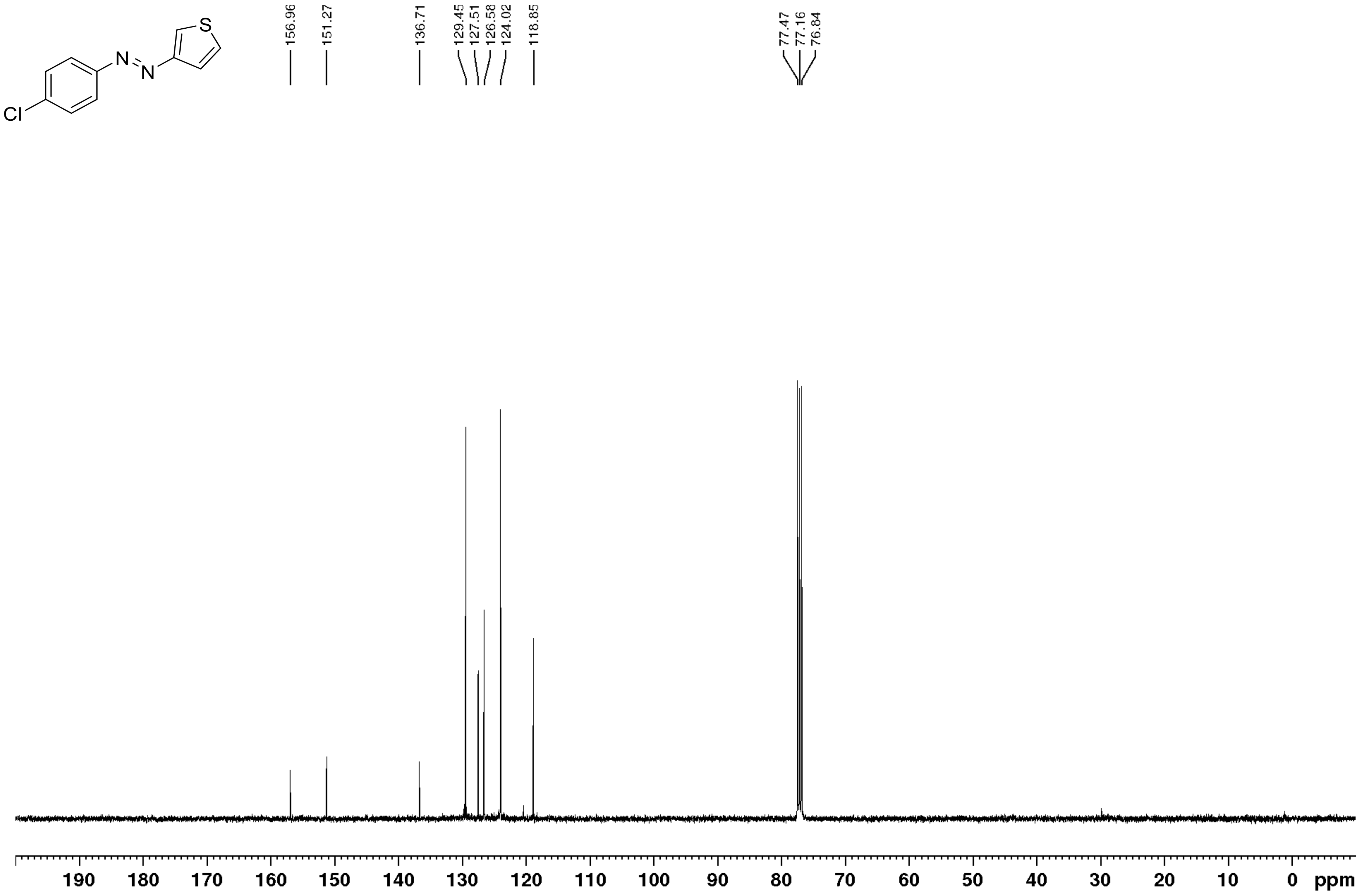

Figure S95. ^{1}H NMR spectrum (500 MHz, $CDCl_3$) of (*E*)-1-(naphthalen-2-yl)-2-(thiophen-2-yl)diazene (**9kc**).

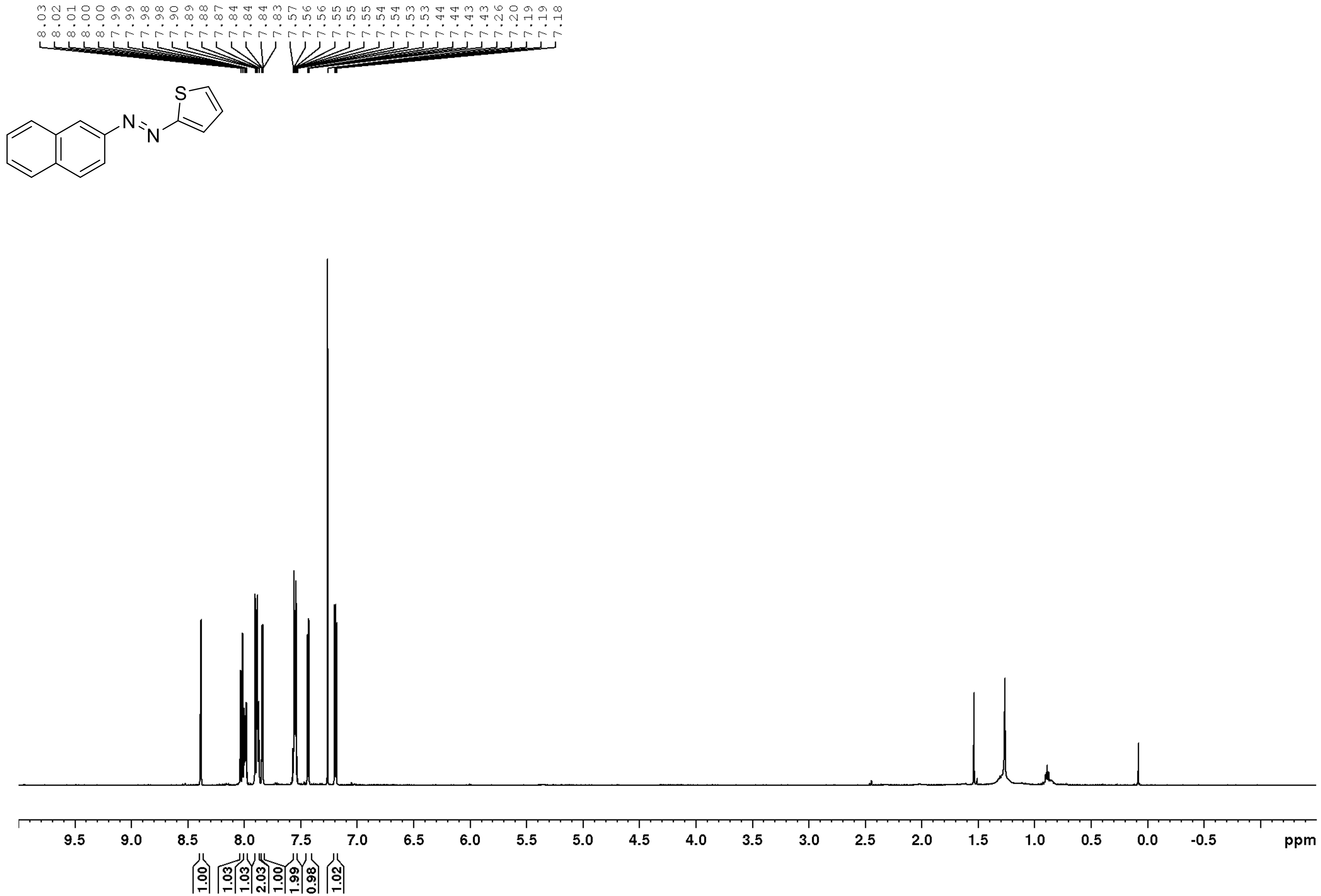

Figure S96. $^{13}C\{^{1}H\}$ NMR spectrum (126 MHz, $CDCl_3$) of (*E*)-1-(naphthalen-2-yl)-2-(thiophen-2-yl)diazene (**9kc**).

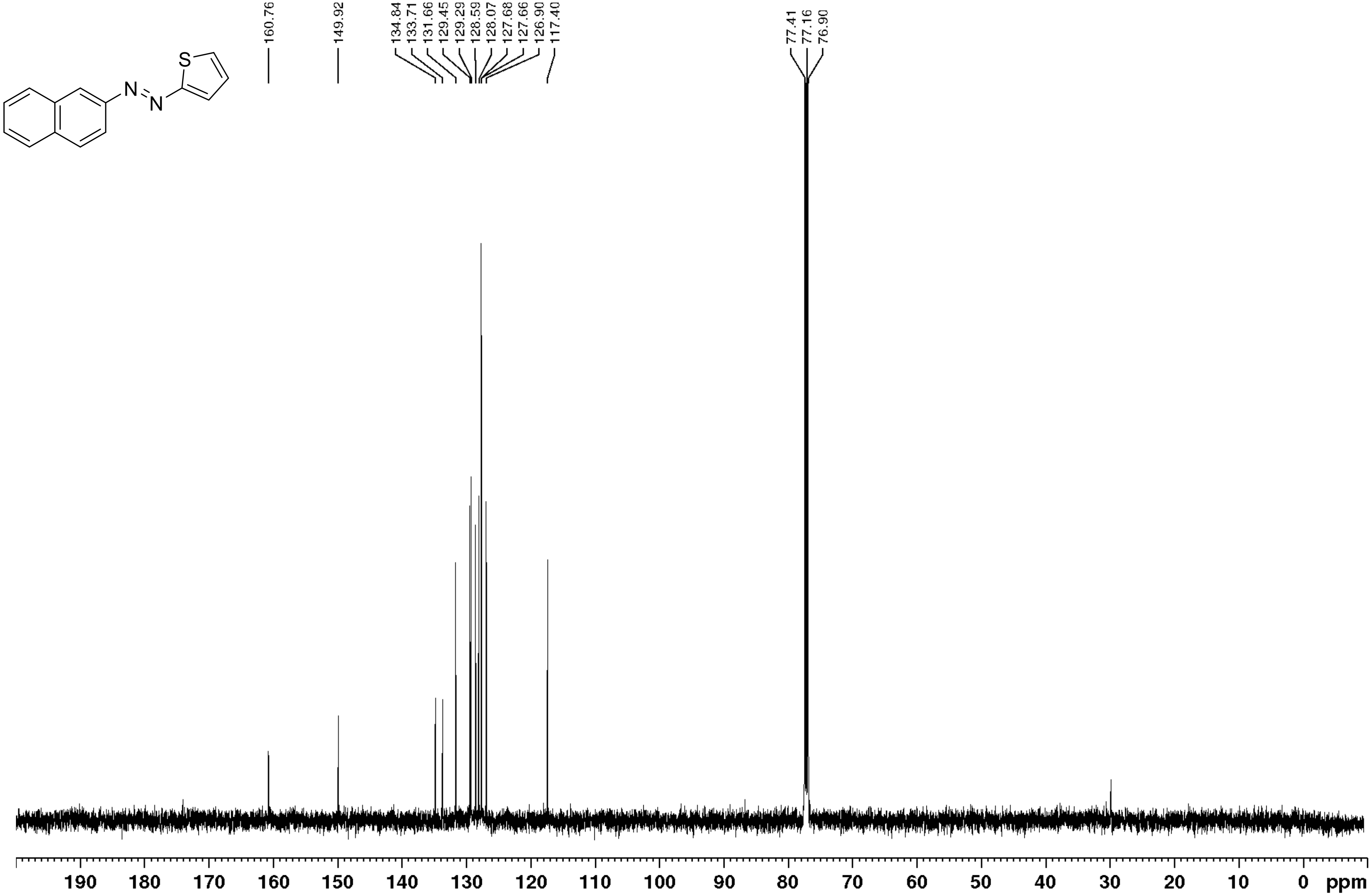

Figure S97. ^{1}H NMR spectrum (500 MHz, $CDCl_3$) of *tert*-butyl (*E*)-5-((4-methoxyphenyl)diazenyl)-1*H*-indole-1-carboxylate (**9bd**).

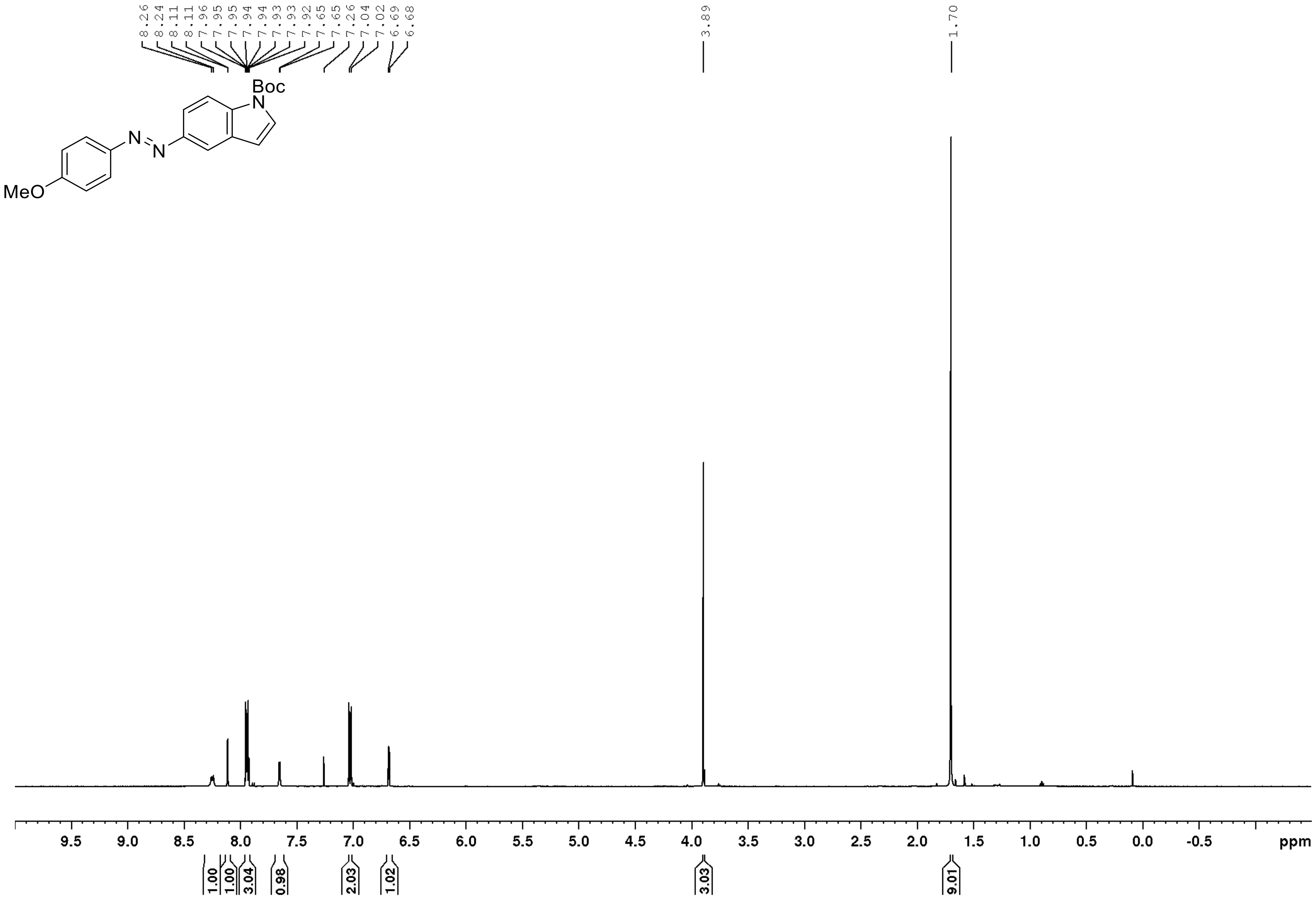

Figure S98. $^{13}C\{^{1}H\}$ NMR spectrum (126 MHz, $CDCl_3$) of *tert*-butyl (*E*)-5-((4-methoxyphenyl)diazenyl)-1*H*-indole-1-carboxylate (**9bd**).

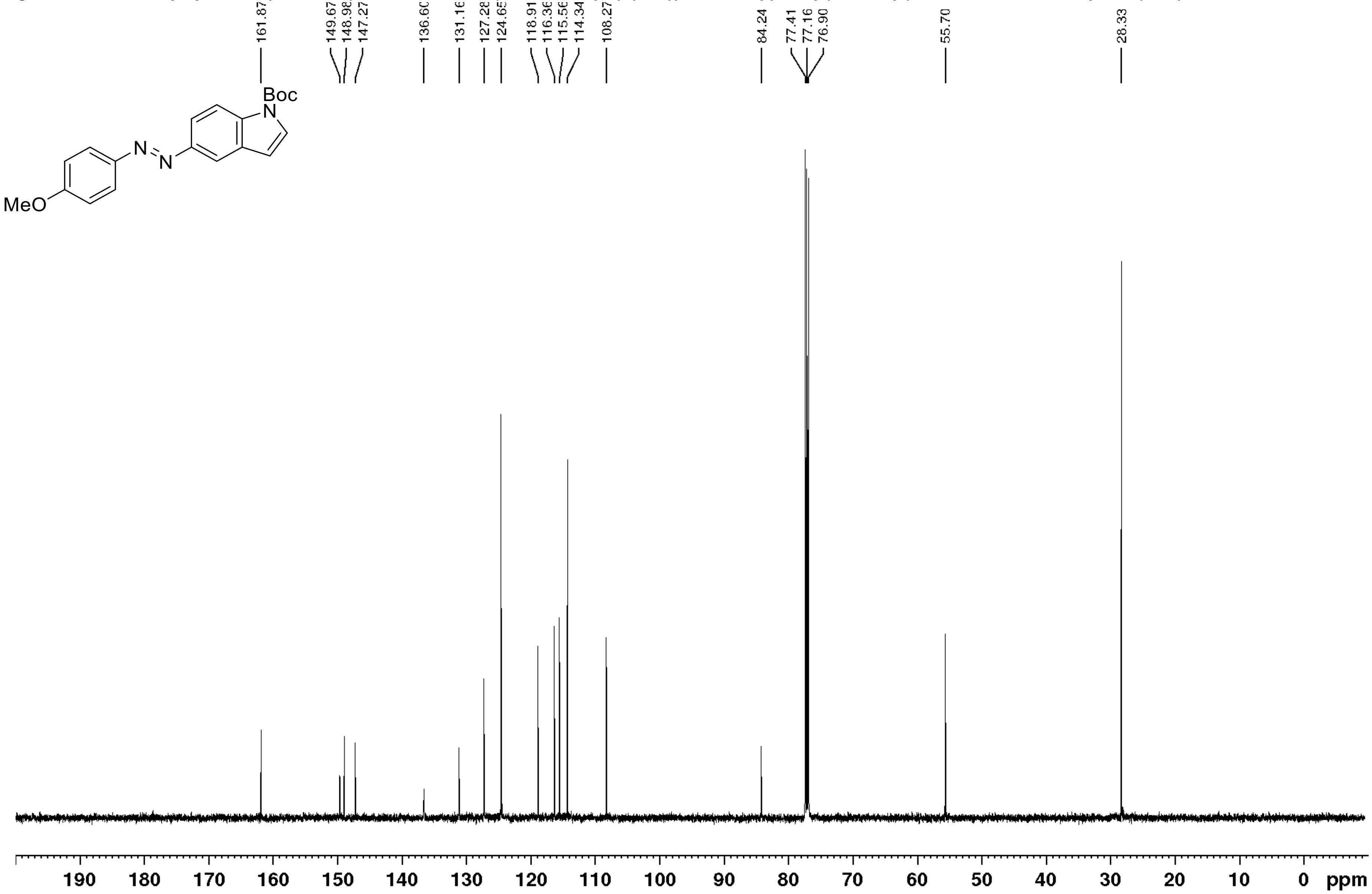

Figure S99. ^{1}H NMR spectrum (400 MHz, $CDCl_3$) of (*E*)-1-(benzofuran-5-yl)-2-(4-methoxyphenyl)diazene (**9be**).

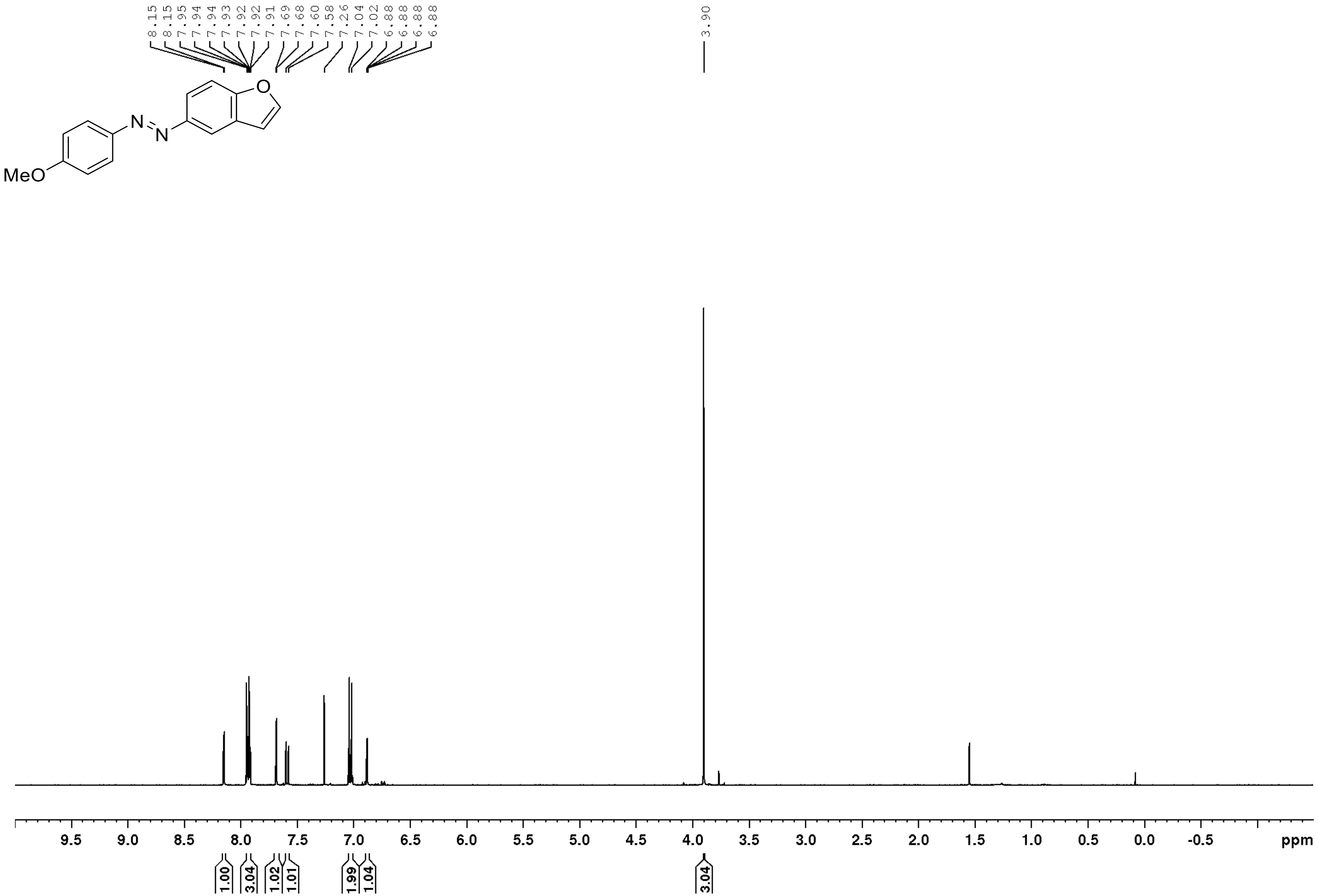

Figure S100. $^{13}C\{^{1}H\}$ NMR spectrum (101 MHz, $CDCl_3$) of (*E*)-1-(benzofuran-5-yl)-2-(4-methoxyphenyl)diazene (**9be**).

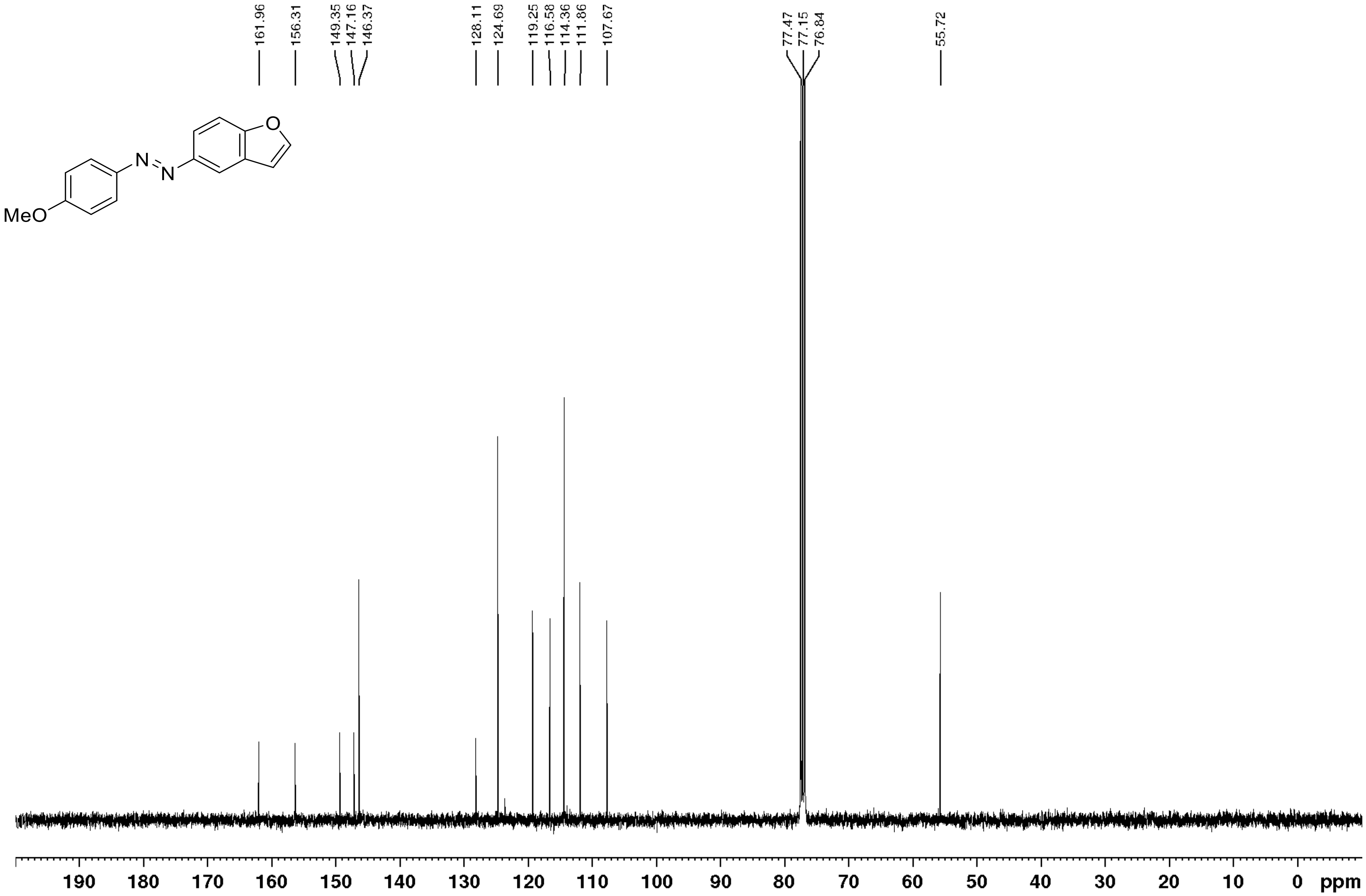

Figure S101. ^{1}H NMR spectrum (400 MHz, $CDCl_3$) of (*E*)-1-(benzo[*b*]thiophen-5-yl)-2-(4-methoxyphenyl)diazene (**9bf**).

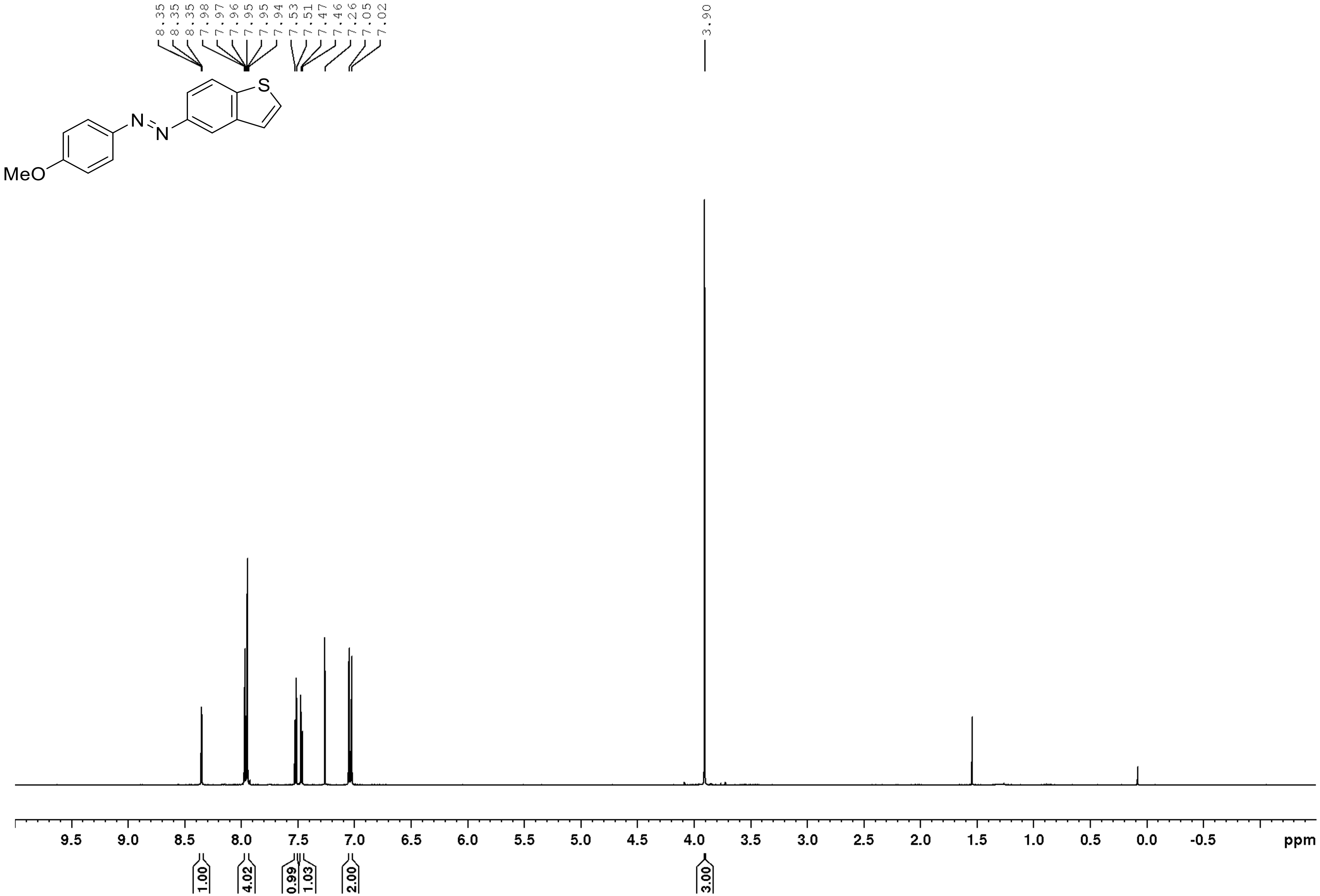

Figure S102. $^{13}C\{^1H\}$ NMR spectrum (101 MHz, $CDCl_3$) of (*E*)-1-(benzo[*b*]thiophen-5-yl)-2-(4-methoxyphenyl)diazene (**9bf**).

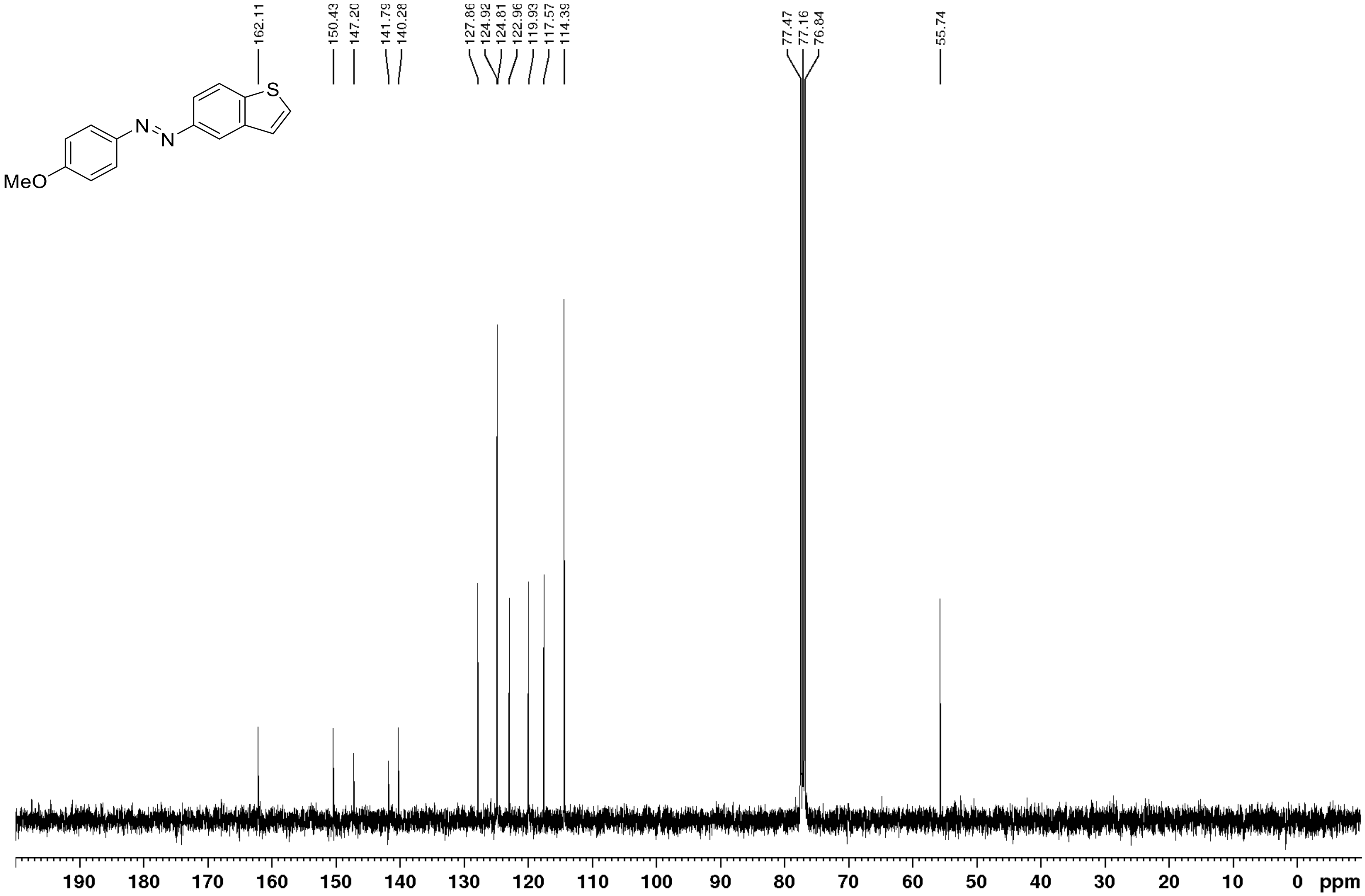

Figure S103. ^{1}H NMR spectrum (400 MHz, $CDCl_3$) of (*E*)-3-((4-methoxyphenyl)diazenyl)quinoline (**9bg**).

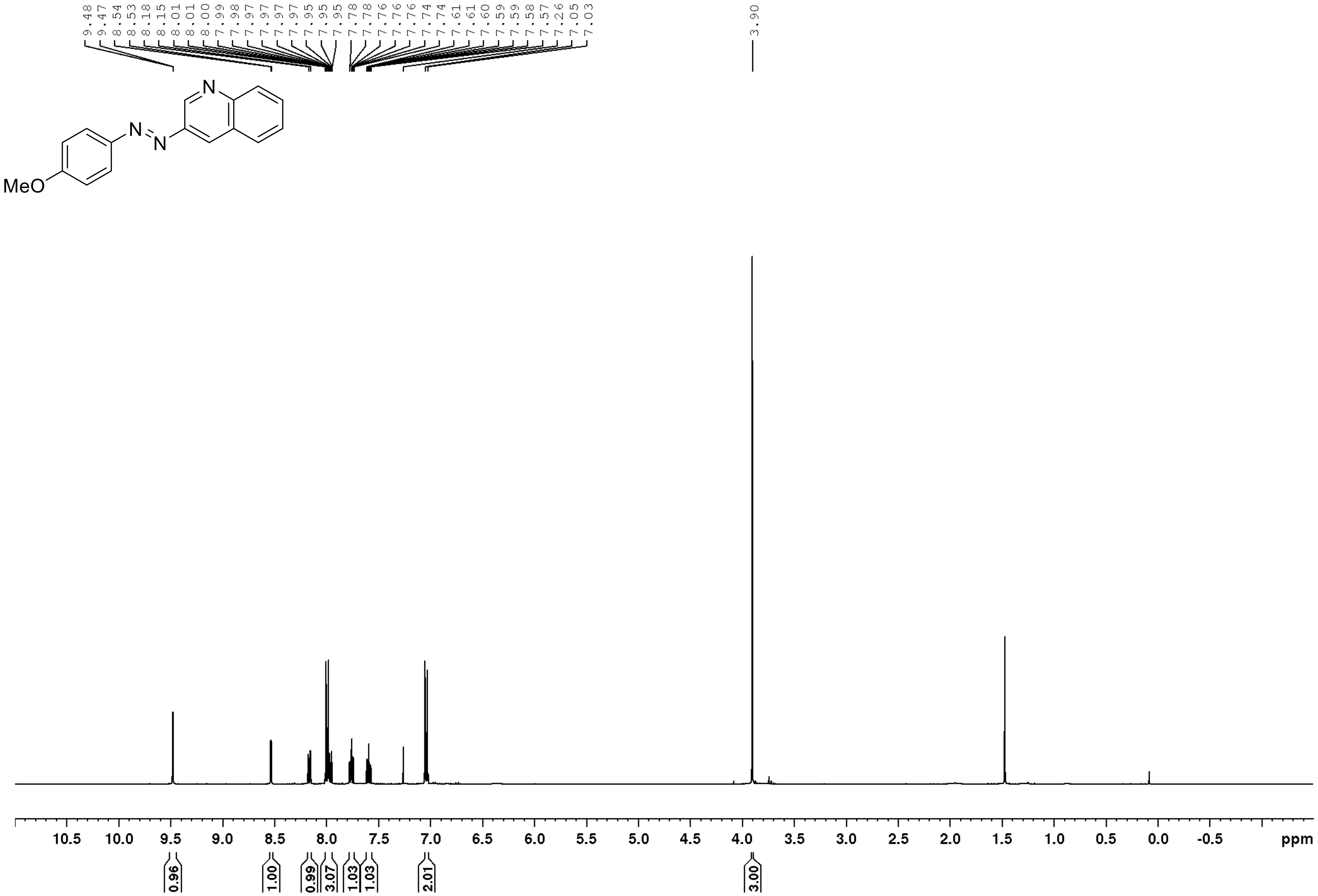

Figure S104. $^{13}C\{^1H\}$ NMR spectrum (101 MHz, $CDCl_3$) of (*E*)-3-((4-methoxyphenyl)diazenyl)quinoline (**9bg**).

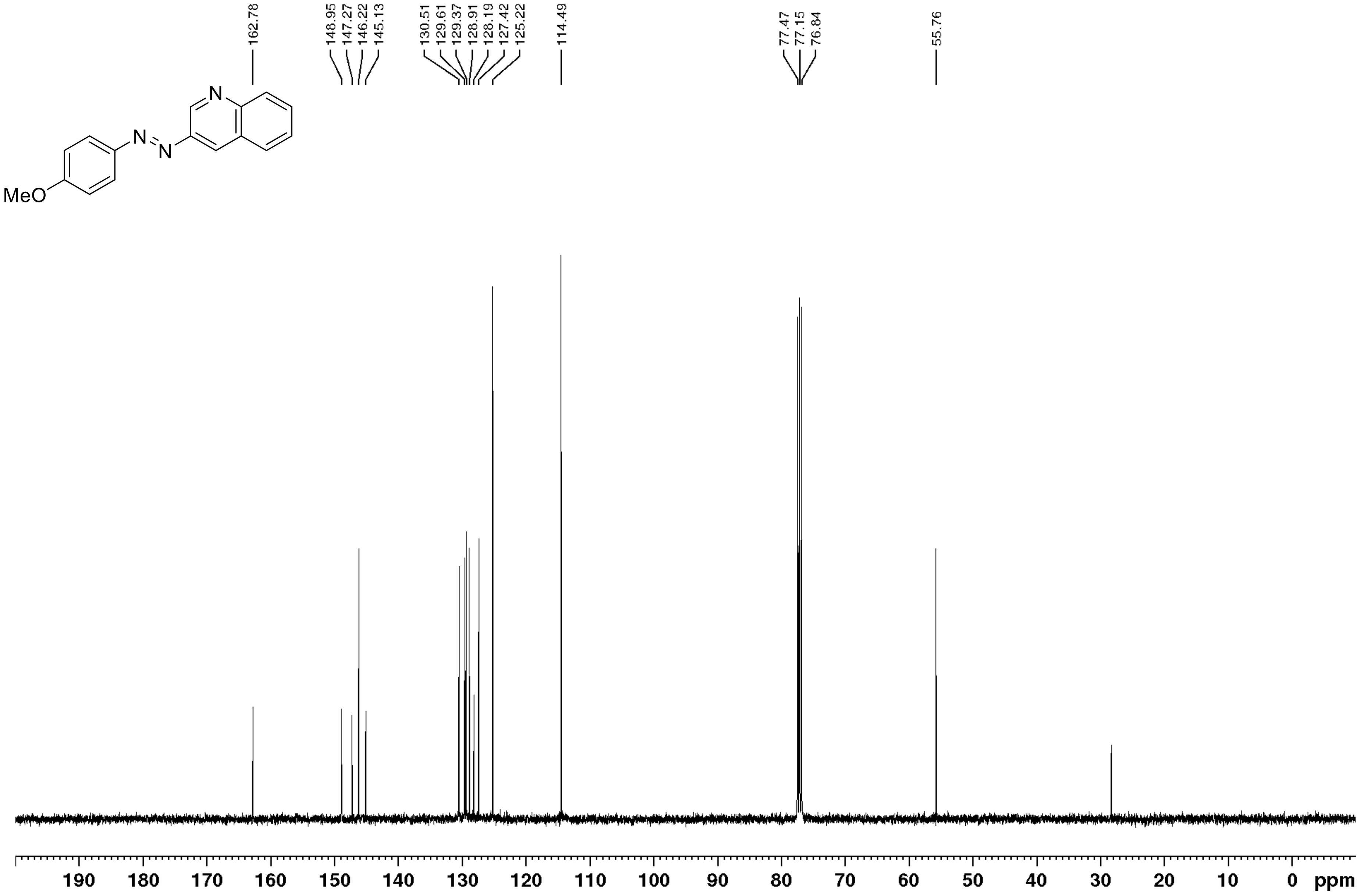

Figure S105. ^{1}H NMR spectrum (400 MHz, $CDCl_3$) of (*E*)-1-(benzo[*b*]thiophen-3-yl)-2-(4-(trifluoromethyl)phenyl)diazene (**9fh**).

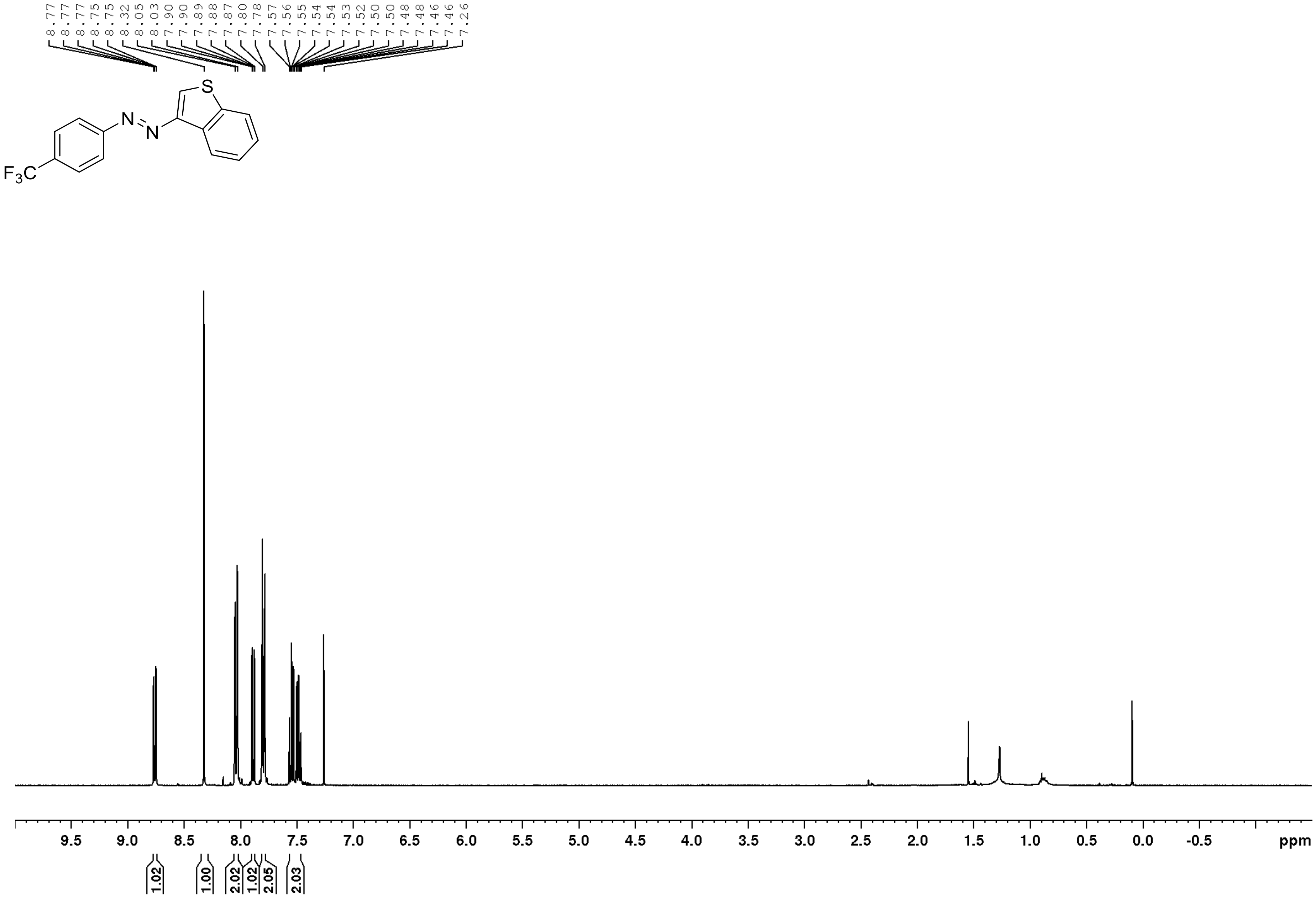

Figure S106. $^{13}C\{^{1}H\}$ NMR spectrum (101 MHz, $CDCl_3$) of (*E*)-1-(benzo[*b*]thiophen-3-yl)-2-(4-(trifluoromethyl)phenyl)diazene (**9fh**).

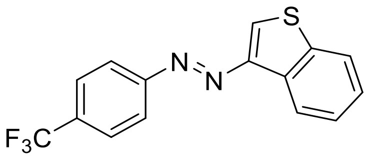

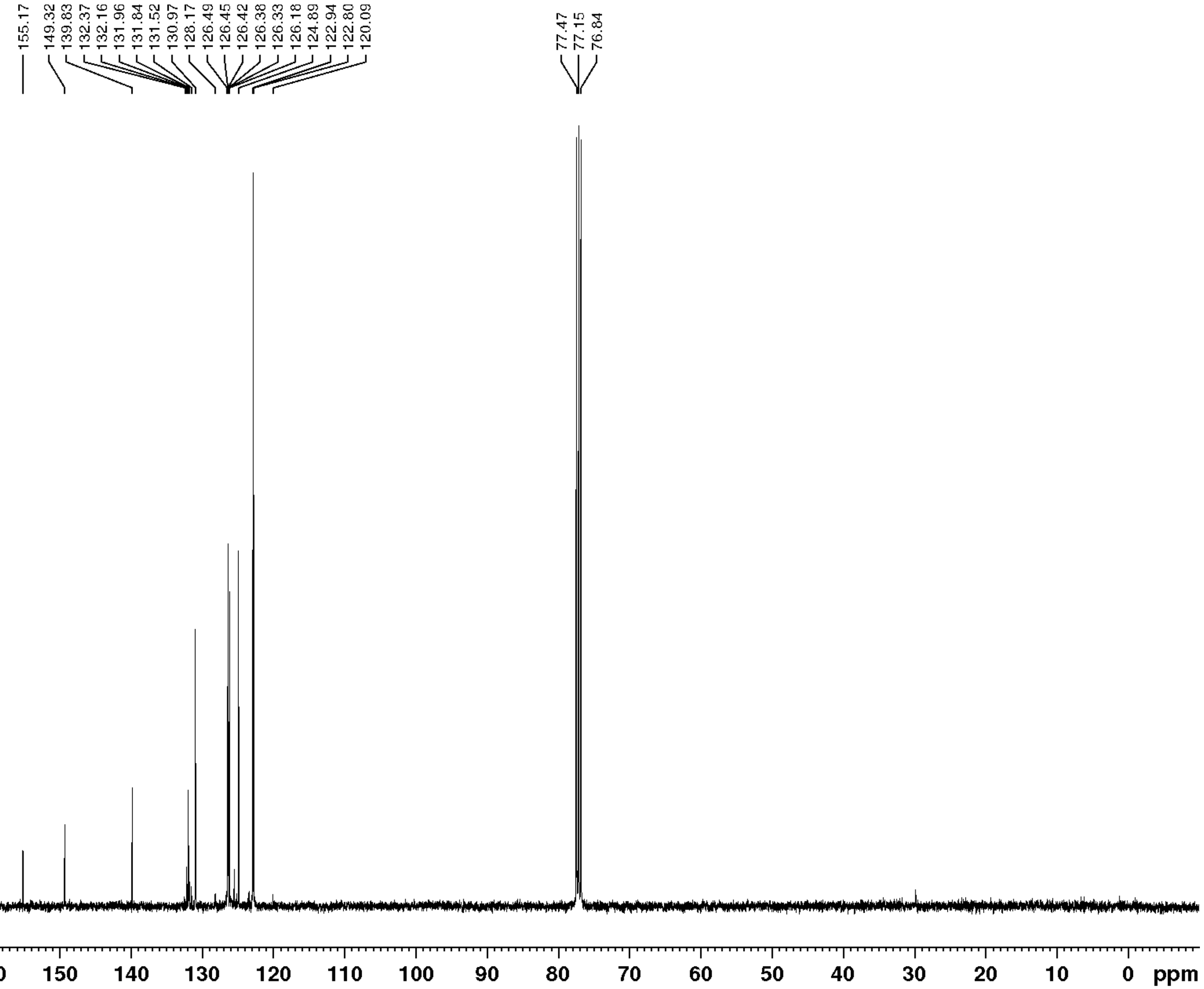

Figure S107. ^{19}F NMR spectrum (471 MHz, $CDCl_3$) of (*E*)-1-(benzo[*b*]thiophen-3-yl)-2-(4-(trifluoromethyl)phenyl)diazene (**9fh**).

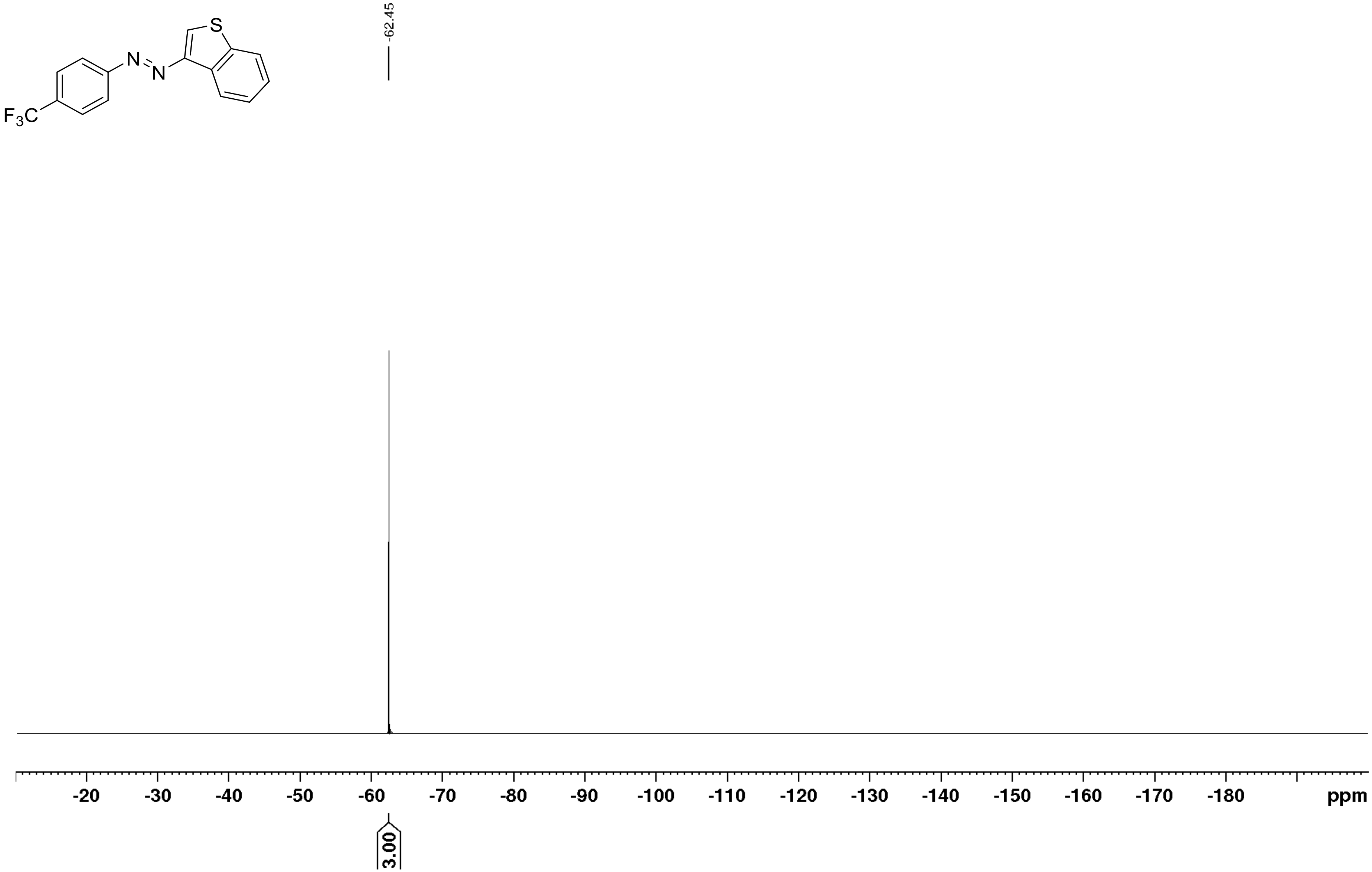

8 References

[S1] a) C. Chauvier, L. Finck, S. Hecht, M. Oestreich, *Organometallics* **2019**, *38*, 4679–4686; b) C. Chauvier, L. Finck, E. Irran, M. Oestreich, *Angew. Chem. Int. Ed.* **2020**, *59*, 12337–12341; *Angew. Chem.* **2020**, *132*, 12436–12440; c) L. Finck, M. Oestreich, *Chem. Eur. J.* **2021**, *27*, 11061–11064.

[S2] a) J.-C. Hsieh, Y.-H. Chu, K. Muralirajan, C.-H. Cheng, *Chem. Commun.* **2017**, *53*, 11584–11587; b) W. Liu, J. Li, P. Querard, C.-J. Li, *J. Am. Chem. Soc.* **2019**, *141*, 6755–6764.

[S3] W. C. Still, M. Kahn, A. Mitra, *J. Org. Chem.* **1978**, *43*, 2923–2925.

[S4] a) H. E. Gottlieb, V. Kotlyar, A. Nudelman, *J. Org. Chem.* **1997**, *62*, 7512–7515; b) G. R. Fulmer, A. J. M. Miller, N. H. Sherden, H. E. Gottlieb, A. Nudelman, B. M. Stoltz, J. E. Bercaw, K. I. Goldberg, *Organometallics* **2010**, *29*, 2176–2179.

[S5] R. K. Harris, E. D. Becker, S. M. Cabral de Menezes, R. Goodfellow, P. Granger, *Pure Appl. Chem.* **2001**, *73*, 1795–1818.
